# Exploring the genetic basis of natural resistance to microcins

**DOI:** 10.1099/mgen.0.001156

**Published:** 2024-02-26

**Authors:** Soufiane Telhig, Nguyen Phuong Pham, Laila Ben Said, Sylvie Rebuffat, Marc Ouellette, Séverine Zirah, Ismaïl Fliss

**Affiliations:** ^1^​ Food Science Department, Food and Agriculture Faculty, Laval University, Quebec, Canada; ^2^​ Laboratoire Molécules de Communication et Adaptation des Microorganismes, Muséum national d’Histoire naturelle, Centre national de la Recherche scientifique, Paris, France; ^3^​ Centre de Recherche en Infectiologie du Centre de Recherche du CHU de Québec and Département de Microbiologie, Infectiologie et Immunologie, Faculté de Médecine, Université Laval, Québec City, Québec, Canada; ^4^​ Institute of Nutrition and Functional Foods, Laval University, Quebec, Canada

**Keywords:** antibiotics, Gram-negative bacteria, microcin, resistance

## Abstract

*Enterobacteriaceae* produce an arsenal of antimicrobial compounds including microcins, ribosomally produced antimicrobial peptides showing diverse structures and mechanisms of action. Microcins target close relatives of the producing strain to promote its survival. Their narrow spectrum of antibacterial activity makes them a promising alternative to conventional antibiotics, as it should decrease the probability of resistance dissemination and collateral damage to the host’s microbiota. To assess the therapeutic potential of microcins, there is a need to understand the mechanisms of resistance to these molecules. In this study, we performed genomic analyses of the resistance to four microcins [microcin C, a nucleotide peptide; microcin J25, a lasso peptide; microcin B17, a linear azol(in)e-containing peptide; and microcin E492, a siderophore peptide] on a collection of 54 *Enterobacteriaceae* from three species: *Escherichia coli*, *Salmonella enterica* and *Klebsiella pneumoniae*. A gene-targeted analysis revealed that about half of the microcin-resistant strains presented mutations of genes involved in the microcin mechanism of action, especially those involved in their uptake (*fhuA*, *fepA*, *cirA* and *ompF*). A genome-wide association study did not reveal any significant correlations, yet relevant genetic elements were associated with microcin resistance. These were involved in stress responses, biofilm formation, transport systems and acquisition of immunity genes. Additionally, microcin-resistant strains exhibited several mutations within genes involved in specific metabolic pathways, especially for *S. enterica* and *K. pneumoniae*.

## Data Summary

The genome sequence data generated in this study have been submitted to the NCBI BioProject database (https://www.ncbi.nlm.nih.gov/bioproject/) under accession number PRJNA915079. The genome-wide association study data obtained in the study are provided in the supplementary data.

**Impact Statement**

Presently, we are not able to produce new antibiotics faster than bacteria can develop resistance. In particular, infections by antibiotic-resistant bacteria are becoming harder to treat and are poised to overtake cancer as the biggest cause of human mortality. In this context, it is no longer enough to simply evaluate the antimicrobial potential of new therapies, and it is paramount to study their resistance emergence in nature. Microcins are a diverse group of antimicrobial peptides produced by enterobacteria, which target other enterobacteria. Previously we showed that microcins are able to inhibit certain multidrug-resistant enterobacteria. Little is known about the genetic relationship between antibiotic resistance and microcin resistance on the one hand, and the development of microcin resistance in nature on the other. Here, we have investigated the genetic elements behind microcin resistance. According to our results, they seem to be independent of antibiotic resistance genes, yet they could be related to other mechanisms employed by enterobacteria to deal with different environmental stresses. We believe such an approach is necessary for the evaluation of any new potential antimicrobial in order to avoid exacerbating the antibiotic resistance problem. Additionally, it can offer relevant data on bacterial adaptations to stressful environments.

## Introduction

*Enterobacteriaceae* form a large family of Gram-negative bacteria that include various harmless commensals together with critical pathogens involved in disease and outbreaks, impacting veterinary and human health [1]. The increasing prevalence of multidrug resistance in *Enterobacteriaceae* constitutes a serious threat to global health [2, 3]. The outer membrane (OM) of Gram-negative bacteria constitutes their first line of defence and any novel antimicrobial needs to overcome it if it is to succeed [4]. The OM serves as a filtering barrier against potential noxious elements. It is dotted with multiple passive porins such as OmpA, OmpC and OmpF. These porins filter incoming compounds based on their chemico-physical properties [5] and can be used as entry gates by antibiotics such as β-lactams. However, mutations in the encoding genes or modulation of their expression level can trigger resistance to antibiotics [6, 7]. In addition, Gram-negative bacteria possess a plethora of efflux systems capable of reducing the intracellular concentrations of any harmful compound [8]. Furthermore, dissemination of antimicrobial resistance (AMR) genes has been increasing in recent years in *Enterobacteriaceae* species, due to their ability to exchange genetic material through horizontal gene transfer [9]. Given the capacity of *Enterobacteriaceae* to acquire and disseminate antibiotic resistance, new therapies are needed.

The family *Enterobacteriaceae* could provide such new therapies, due to their antimicrobial metabolites such as microcins, a family of bacteriocins showing antibacterial activity against strains phylogenetically related to their producer [10–12]. Their narrow spectrum of activity compared to that of conventional antibiotics should limit collateral damage to the host microbiome, the homeostasis of which is crucial for health [13]. Also, it should reduce the risk of resistance dissemination, should it emerge [14]. Microcins make up a structurally diverse family of peptides, translating to a diversity of mechanisms of action. They are ribosomally synthesized peptides below 10 kDa that parasitize different nutrient uptake receptors of the target cell, effectively using a ‘Trojan Horse’ mechanism to penetrate susceptible bacteria. The potential of these compounds and by extension bacteriocins has already been highlighted multiple times in previous reviews [11, 15, 16], yet the question of resistance remains. It should be noted that there is a finite number of entry points into Gram-negative bacteria and it is possible that both antibiotics and microcins could present commonalities leading to cross-resistance.

Furthermore, Gram-negative bacteria are notorious for their capacity to develop resistance due to their intrinsic resistance. Thus, naturally occurring genes such as RND superfamily genes [8] or *mcr* [17] can increase the fitness of a strain as well as its resistance to antimicrobials. To shed light on the potential of microcins as new therapies and subsequently any potential cross-resistance, a previous study was conducted [18]. The antibacterial activity of four microcins was tested against a collection of *Enterobacteriaceae* along with different consortia of microcins and antibiotics. The tested collection contained pathogens and multi-drug-resistant (MDR) strains from three different species, namely *Escherichia coli*, *Klebsiella pneumoniae* and *Salmonella enterica*. The four tested microcins were: microcin C (McC), a nucleotide peptide acting as an aspartyl tRNA synthetase inhibitor once processed inside the target cell [19]; microcin J25 (MccJ25), a lasso peptide that inhibits RNA polymerase [20]; microcin B17 (MccB17), a peptide with thiazole and oxazole rings that inhibits DNA-gyrase [21–23]; and microcin E492 (MccE492), a siderophore peptide causing inner membrane destabilization and alteration of mannose transport through an association with the mannose permease [24]. The proteins involved in the mechanism of action of each microcin are listed in Table S1, available in the online version of this article. It was observed that no strain was resistant to all tested microcins, despite many of them being MDR strains. The tested microcins exhibited varying activities and range of action. Globally, McC was found to possess the widest range of inhibition, i.e. was able to inhibit the largest number of strains. In contrast, MccJ25 exhibited the narrowest range of inhibition, but the most efficient activities, i.e. the lowest minimal inhibitory concentration (MIC). Furthermore, *K. pneumoniae* strains were found to be ‘naturally’ resistant to MccJ25 and MccB17, with the exception of a single strain, C4750. In addition, despite multiple shared entry points between the tested microcins and antibiotics, no antagonism was observed. However, all of these observations were phenotypical, and it remained unclear which genetic elements might explain the microcin resistance phenotypes.

Microcin resistance has not yet been studied using a holistic approach and natural resistance emergence is poorly understood. In previous studies, spontaneous mutants resistant to microcins were generated to elucidate their mechanism of action [25–28]. The corresponding mutations were located on genes encoding the proteins involved in microcin uptake into the target cell or, more rarely, the intracellular target. Immunity of the producing strains relies generally on efflux by dedicated ABC transporters [27, 29], or for McC inactivation by the acetyl transferase MccE and/or by the peptidase MccF [30–32]. For MccB17, an immunity protein, McbG, which belongs to the pentapeptide repeat protein (PRP) family, is produced in addition to the efflux transporter McbEF and protects DNA gyrase from MccB17 [23]. Resistance to microcins can result from efflux pumps such as YojI or its regulatory protein Lrp [26] for MccJ25, or acetylation by the transacetylase RimL for the processed form of McC [28]. Moreover, a survey of the sensitivity of several *S. enterica* serovars showed that MccJ25 was highly active against some serovars, while *S*. Typhimurium, *S*. Derby and some *S*. Enteritidis strains were resistant [33]. It was shown that the difference in the susceptibility to MccJ25 of *Salmonella* strains was mainly attributed to differences in the sequence of FhuA, the OM hydroxamate siderophore receptor ensuring MccJ25 uptake [34]. Where our previous study had a phenotypical approach to the potential of microcins, the current study aimed to explore the genetic elements capable of explaining the previously observed microcin resistance phenotypes [18].

For this study, the AMR and virulence genes from the previous *Enterobacteriaceae* strains were analysed to determine whether they are associated with microcin resistance. In addition, the sequence of all genes implicated in microcin mechanisms of action were compared to determine their influence on the susceptibility to microcins. Finally, a genome-wide association study (GWAS) was conducted to help identify any potential novel gene associations with either microcin or antibiotic resistance.

## Methods

### Bacterial strains and microcins

**Fig. 1.**
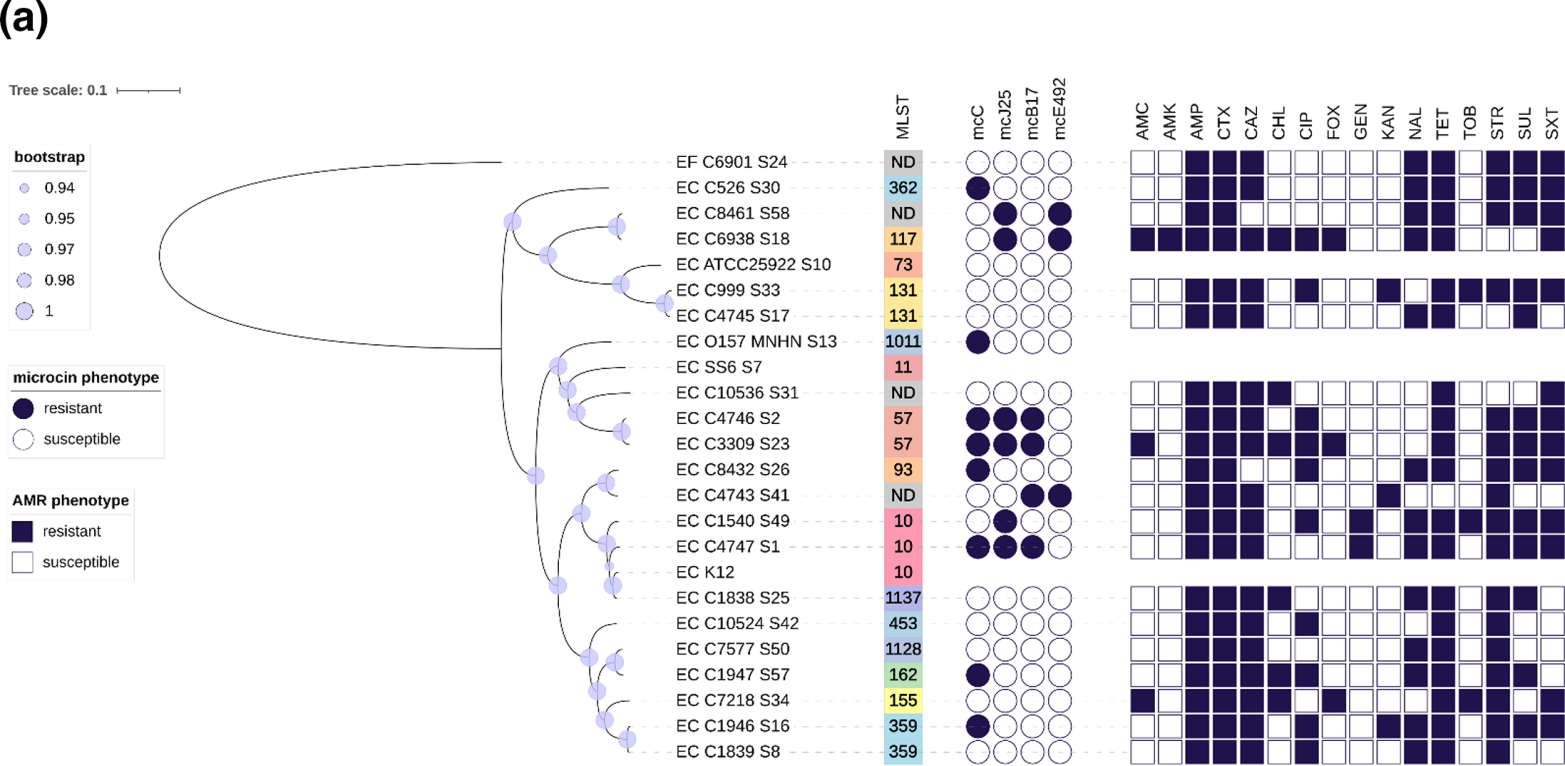

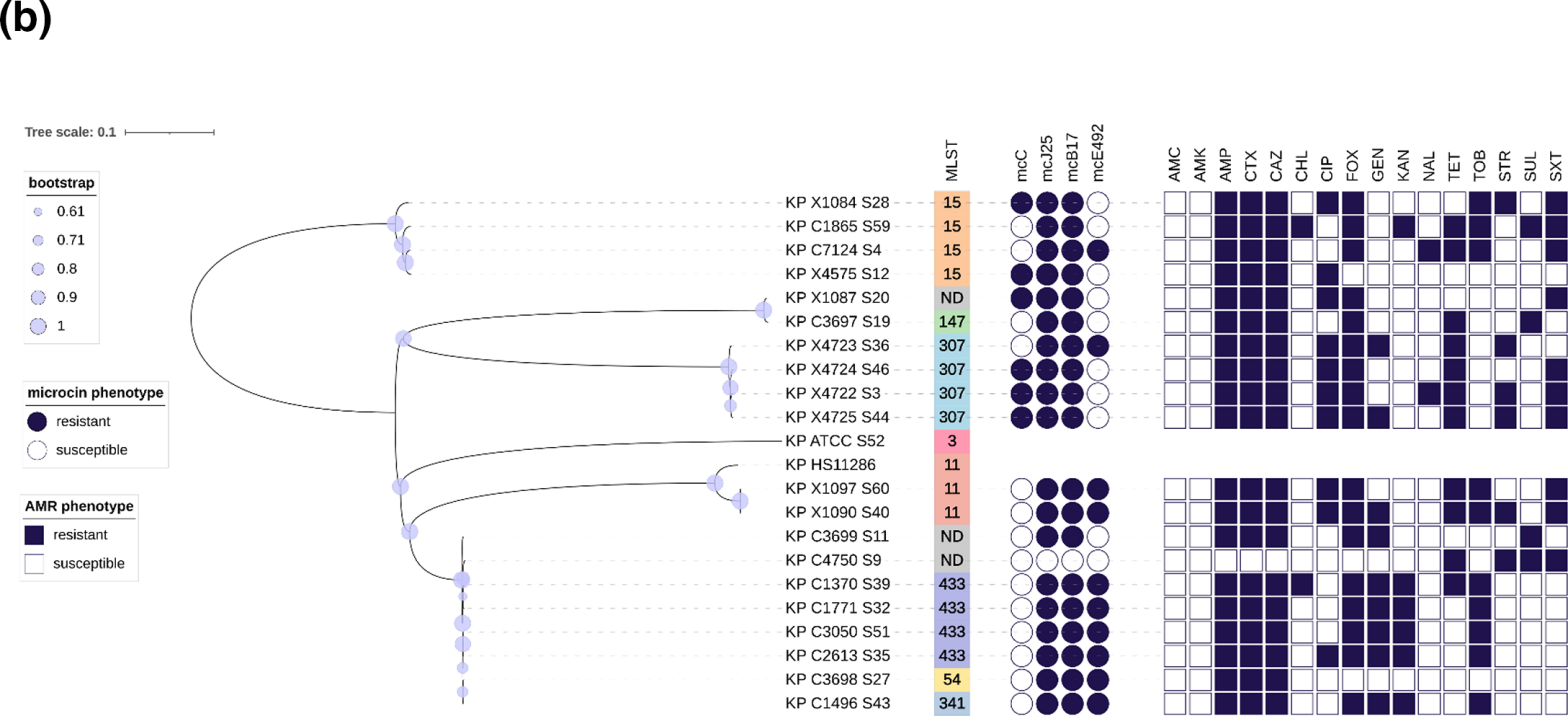

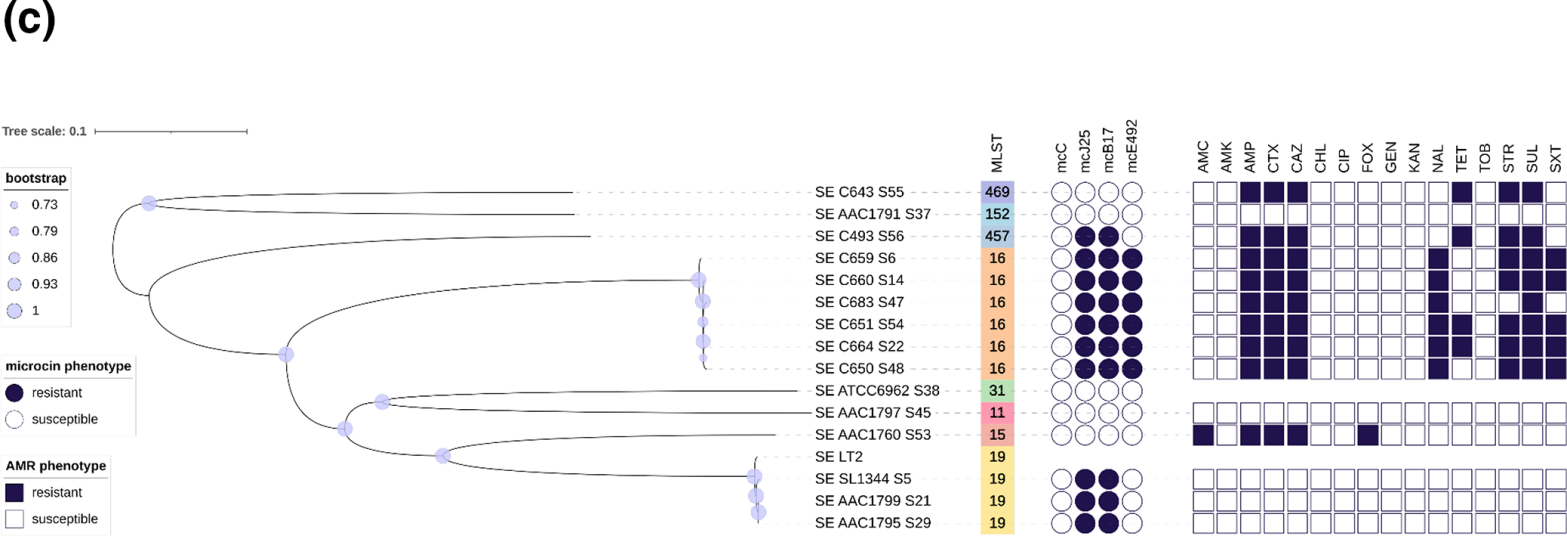
Phylogenetic and resistance data of the *Enterobacteriaceae* collection tested against candidate microcins. (**a**) Phylogenetic data of the *E. coli* strains with the accompanying phylogenetic tree, multi-locus sequence typing (MLST) group, microcin activity, sensitivity and antibiotic resistance profile. (**b**) Phylogenetic data of the *K. pneumoniae* strains with the accompanying phylogenetic tree, MLST group, microcin activity, sensitivity and antibiotic resistance profile. (**c**) Phylogenetic data of the *S. enterica* strains with the accompanying phylogenetic tree, MLST group, microcin activity, sensitivity and antibiotic resistance profile.

The *E. coli*, *K. pneumoniae* and *S. enterica* natural isolates along with their serotypes, antibiotic resistance phenotypes and sequence type (ST) groups are listed in Tables S2, S3 and S4, respectively. These strains were obtained from the collection of the University of La Rioja (Logroño, Spain) and from Agriculture Canada’s pathogen collection and their phenotypes and genotypes of resistance were known from previous studies. Their susceptibility to McC, MccJ25, MccB17 and MccE492 is reported in our previous study [35] (Fig. 1).

### DNA extraction and whole-genome sequencing

DNA extraction was performed with the Qiagen DNeasy blood and tissue kit, following the manufacturer’s instructions for planktonic bacterial cultures. DNA libraries were constructed using the NEBNext Ultra II kit (with Covaris M220 mechanical fragmentation). Sequencing was performed with an Illumina MiSeq 600 v3 cycle kit. The resulting reads were quality-checked and assembled into contigs using the A5 pipeline [36]. Only contigs with length ≥1000 bp were considered for further study.

### Genomic analyses and genome-wide association studies

The genome assemblies from this study, along with the reference genomes of *E. coli* strain K12 substrain MG1655 (Project accession number PRJNA57779), *S. enterica* subsp. *enterica* serovar Typhimurium strain LT2 (Project accession number PRJNA57799) and *K. pneumoniae* subsp. *pneumoniae* HS11286 (Project accession number PRJNA84387) were annotated using Prokka [37]. For each species, a pangenome was generated using the OrthoMCL procedure [38] implemented in the GET-HOMOLOGUES package [39]. Orthologous genes were defined with the following parameters: 75 % as minimum coverage, 75 % as minimum identity in BlastP pairwise alignments and 1e-05 as maximum E-value. Core genome (SNP) analysis was performed using Snippy [40], with *E. coli* strain K12 substrain MG1655, *S. enterica* subsp. *enterica* serovar Typhimurium strain LT2 and *K. pneumoniae* subsp. *pneumoniae* HS11286 as reference genomes. Core SNP alignment was used to build the phylogenetic tree using FastTree [41]. Multilocus sequence typing (MLST) analysis was performed using mlst v2.19.0 [42] and the PubMLST database [43]. AMR genes and virulence factors were screened using the Comprehensive Antibiotic Resistance Database – Resistance Gene Identifier (CARD-RGI) [44] and the Virulence Factors database (VFDB) [45] via ABRicate software [46], respectively. GWAS was conducted on the SNP and pangenome matrices using Scoary [47].

Due to a limited number of samples (*n*=57: *E. coli n*=22; *K. pneumoniae n*=20; *S. enterica n*=15), false discovery rate (FDR) multiple correction methods were applied and no SNP/gene was associated above the Bonferroni-corrected threshold (*P*<0.05). Associations in the pangenome or ‘accessory genome’ were referred to as gene-content associations in contrast to core genome (SNP) associations.

GWAS data are available in the supplementary material, with the naive *P*-values. The heatmaps used to describe the genomic analysis data were made ccording to Gu *et al*. [48]. If not mentioned otherwise, the bioinformatic tools were used with default settings.

## Results

The susceptibility to microcins and antibiotics of the collection of 54 strains of *Enterobacteriaceae*, reported in our previous study [18], were based on the observed phenotypes; that is, any strain capable of growing at the maximum tested concentration of a given microcin (50 µg ml^−1^) or an antibiotic was considered to be resistant. These observations are summarized in Fig. 1. Given the purely phenotypical nature of the study, a more holistic investigation of their genomes was needed. Genotypic analysis of the strains by MLST based on their variable number of tandem repeats (Tables S2–S4) was performed (Fig. 1). *E. coli* was the most diverse group with 14 unique STs, whereas *S. enterica* and *K. pneumoniae* strains each exhibited seven unique STs. *E. coli* MLST analysis showed two novel alleles for the genes *recA* and *fumC* in strains C8461 and C10536, respectively. *K. pneumoniae* also presented multiple novel alleles: for the gene *gapA* in strain X1087 and for the genes *mdh*, *pgi*, *phoE*, *rpoB* and *tonB* in strain C4750. The latter strain was susceptible to all four microcins and the only *K. pneumoniae* strain susceptible to MccJ25 and MccB17, in contrast to all other *Klebsiella* strains which were resistant to both MccJ25 and MccB17. A few correlations between MLST profiles and susceptibility to microcins were observed. As an example, the six strains from the ST16 group of *S. enterica* were resistant to MccB17, MccJ25 and MccE492 (Fig. 1).

Genomic analysis permitted us to detect the virulence and AMR genes for each strain (Fig. S1), showing three distinct profiles based on the three species studied. *E. coli* genomes possessed the highest arsenal in terms of the number of AMR genes, representing 59.53 % of all AMR genes detected in this study. In general, all strains presented a diverse range of AMR genes with the most common implicated in resistance to tetracycline, aminoglycoside and fluoroquinolone antibiotics. Other important AMR genes such as *oxa-48* were detected to a lesser degree, mainly in *K. pneumoniae* strains. Concerning virulence, of all the detected virulence genes, *E. coli* and *S. enterica* presented 44.83 and 39.35 %, respectively. In contrast, *K. pneumoniae* presented 8 % of the detected virulence genes. The gene *ompA* was identified as both an AMR and virulence gene and was highly represented in *E. coli* and *S. enterica* strains.

No clear correlation was observed between AMR or virulence genes and susceptibility to microcins. Only one correlation was noted between the presence of the AMR *mcr-9* gene, assigned to a colistin resistance gene, and MccE492 resistance phenotypes in *S. enterica* strains. However, the hierarchical clustering and distribution of both virulence and resistance genes appeared to be largely species-specific and independent of microcin susceptibility phenotypes (Figs 1 and S1), which is in line with the phenotype data obtained from our previous study.

### Gene-targeted approach

**Fig. 2.**
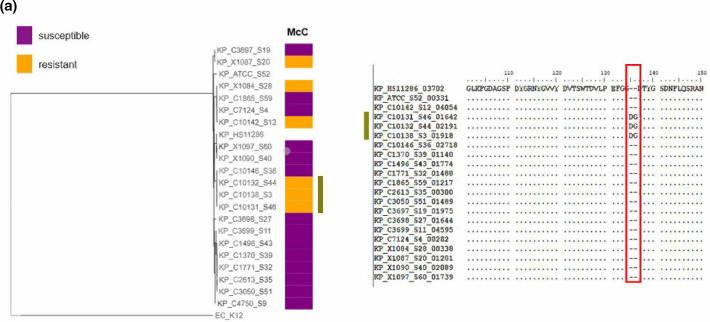

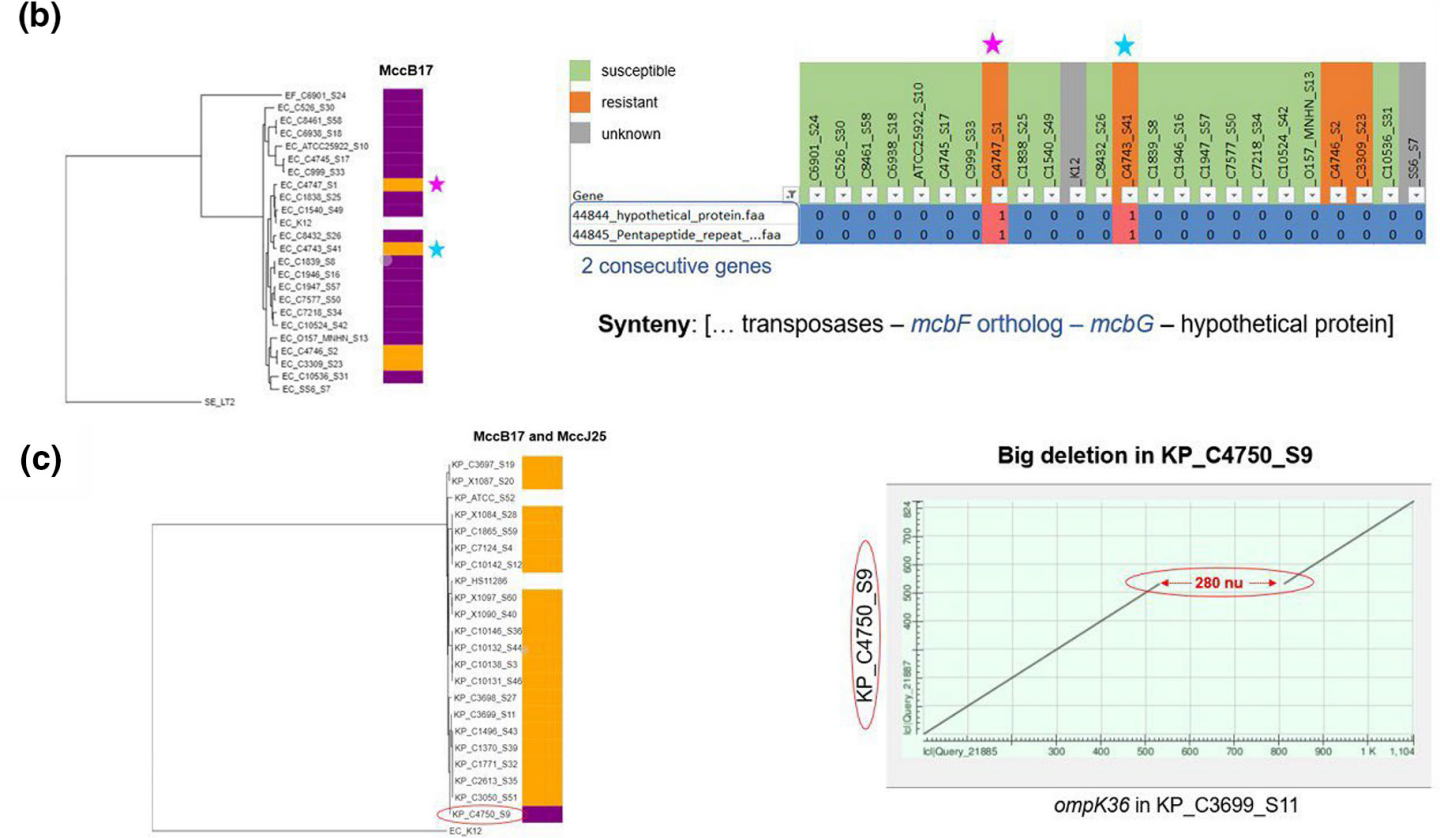
Genetic deviations associated with microcin resistance. (**a**) The insertion mutation in the *ompK36* gene associated with McC resistance in the studied *K. pneumoniae* strains. (**b**) The presence of the immunity gene *mcbG* and an orthologue to the immunity gene *mcbF* associated with MccB17 resistance in the tested *E. coli* strains. (**c**) The only *K. pneumoniae* strain susceptible to MccB17 and MccJ25 harbours a 280 nt deletion on its *ompK36* gene.

According to our previous study, a total of 101 microcin resistance phenotypes were identified (Fig. 1). Given the lack of correlation between the AMR and virulence genes with the observed microcin phenotypes, a comparison of all genes implicated in the mechanisms of action of microcins was conducted (Table S1). The aim of this approach was to first determine the genetic differences directly linked to the antimicrobial activity of microcins as opposed to other potentially non-specific associations. Essentially, all these genes were aligned to detect any biologically relevant correlations (Table 1). Among the 101 microcin resistance phenotypes, 49 (48.5 %) exhibited mutations in genes implicated in the mechanisms of action of the corresponding microcin. The majority of these mutations were detected on the uptake mechanisms of these microcins, such as the OM receptor genes *fepA* and *fhuA* involved in the uptake of MccE492 and MccJ25, respectively. Furthermore, in two *E. coli* strains resistant to MccB17, the *mcbG* gene and an orthologue of the *mcbF* gene were detected (Fig. 2b). These genes, known to be MccB17 immunity genes, are usually harboured by the MccB17 biosynthesis gene cluster, making the production of MccB17 non-lethal. In these strains, only these two genes were detected without the other genes involved in the biosynthesis of MccB17. Of the 49 correlations detected with this analysis, only seven affected genes coding for the final target of the corresponding microcins. Six *Salmonella* strains presented mutations in their *gyrA* gene coding for the final target of MccB17. All mutations affected the Ser^83^ amino acid. Similarly, a single mutation on the *aspS* gene coding for the final target of McC was detected in an McC-resistant *K. pneumoniae* strain. In other *K. pneumoniae* strains, mutations in the *ompK36* gene coding for an OM porin homologous to OmpC in *E. coli* were associated with either McC resistance or MccB17 susceptibility (Fig. 2a–c). In the case of *K. pneumoniae* strains resistant to McC, OmpK36 presented an insertion of two extra amino acids. In contrast, the only *K. pneumoniae* strain susceptible to MccB17 (and to all four microcins) presented a deletion of 280 nt on its *ompK36* gene (Fig. 2c). In summary, mutations in genes implicated in microcin mechanisms of action are correlated with almost half of all observed microcin resistance phenotypes.

**Table 1.** List of mutations on the genes implicated in microcin mechanisms of action All the genetic sequences of microcin partners were analysed compared to the reference strains to find correlations with the microcin phenotype. Presented are the genetic sequences of the genes implicated in microcin mechanisms of action and associated with a resistance phenotype. na, Not applicable.

Microcin	Species	Strains	Genetic mutations	Functions
McC	*E. coli*	C526 and C4747	*ompF*: three-point mutations at position Glu^51^Val, Gly^48^Asp and Thr^99^Lys	Passive outer membrane porin and entry point of McC in the target cell
		C4747 and C3309	*yejB*: frameshift mutation at position 812/813	Inner membrane active transporter and used by McC for translocation from periplasm to cytoplasm
	*S. enterica*	No resistant strain	na	na
	*K. pneumoniae*	X4722, X4724 and X4745	*ompK36*: insertion of 2 aa between positions 135 and 136	Outer membrane passive porin and OmpC homologue, believed to be McC entry point in *Klebsiella*
		X1084	*aspS*: point mutation Gly^544^Asp	tRNA Asp ligase and final target of McC
MccJ25	*E. coli*	C3309, C4746, C6938 and C8461	*fhuA*: sequence divergence	MccJ25 and ferrichrome outer membrane receptor
		C3309 and C4746	*tonB*: point mutation Glu^75^Lys	Member of the three-component active transporter TonB complex
		C3306, C4746, C6938 and C8461	*yojI*: three-point mutations on positions Ala^430^Val, Arg^313^Cys and Ile^91^Val	ATP binding ATP transporter
	*S. enterica*	C493, C650, C651, C659, C660, C664, C683, AAC1795, AAC1799 and SL1344	*fhuA*: sequence divergence	MccJ25 and ferrichrome outer membrane receptor
	*K. pneumoniae*	na	na	na
MccB17	*E. coli*	C3309, C4746	*ompF*: three-point mutations on positions Ala^95^, Thr^263^ and Ser^270^	Outer membrane passive porin and MccB17 entry point
		C4743 and C4747	*mcbG* and *mcbF* orthologue	Immunity genes in the MccB17 biosynthesis gene cluster
	*S. enterica*	C650, C651, C659, C660, C664 and C683	*gyrA*: point mutation at Ser^83^ position	DNA gyrase subunit A and MccB17 final target, position Ser^83^ critical
	*K. pneumoniae*	C4750 is the only susceptible strain	*ompK36* : 280 nt deletion	Outer membrane passive porin and *E. coli* OmpC homologue
MccE492	*E. coli*	C4743, C6938 and C8461	*fepA*: two-point nonsense mutations and one frameshift mutation	Ferrienterobactin receptor and main MccE492 outer membrane receptor
		C6938 and C8461	*fiu*: one-point nonsense mutation	Catecholate siderophore receptor and MccE492 receptor
	*S. enterica*	C650, C651, C659, C660, C664 and C683	*fepA*: point mutation at Val^585^ position	Ferrienterobactin receptor and main MccE492 outer membrane receptor
		C650, C651, C659, C660, C664 and C683	*cirA*: point mutation at Thr^183^ position	Colicin I receptor and MccE492 receptor
	*K. pneumoniae*	C1771, C1370, C1496, C2613 and C3050	*cirA*: point mutation at Y^183^ position	Colicin I receptor and MccE492 receptor
		C6938	*exbD*: point mutation at Thr^94^ position	Biopolymer transport protein and TonB complex component

### Genome-wide association studies

**Fig. 3.**
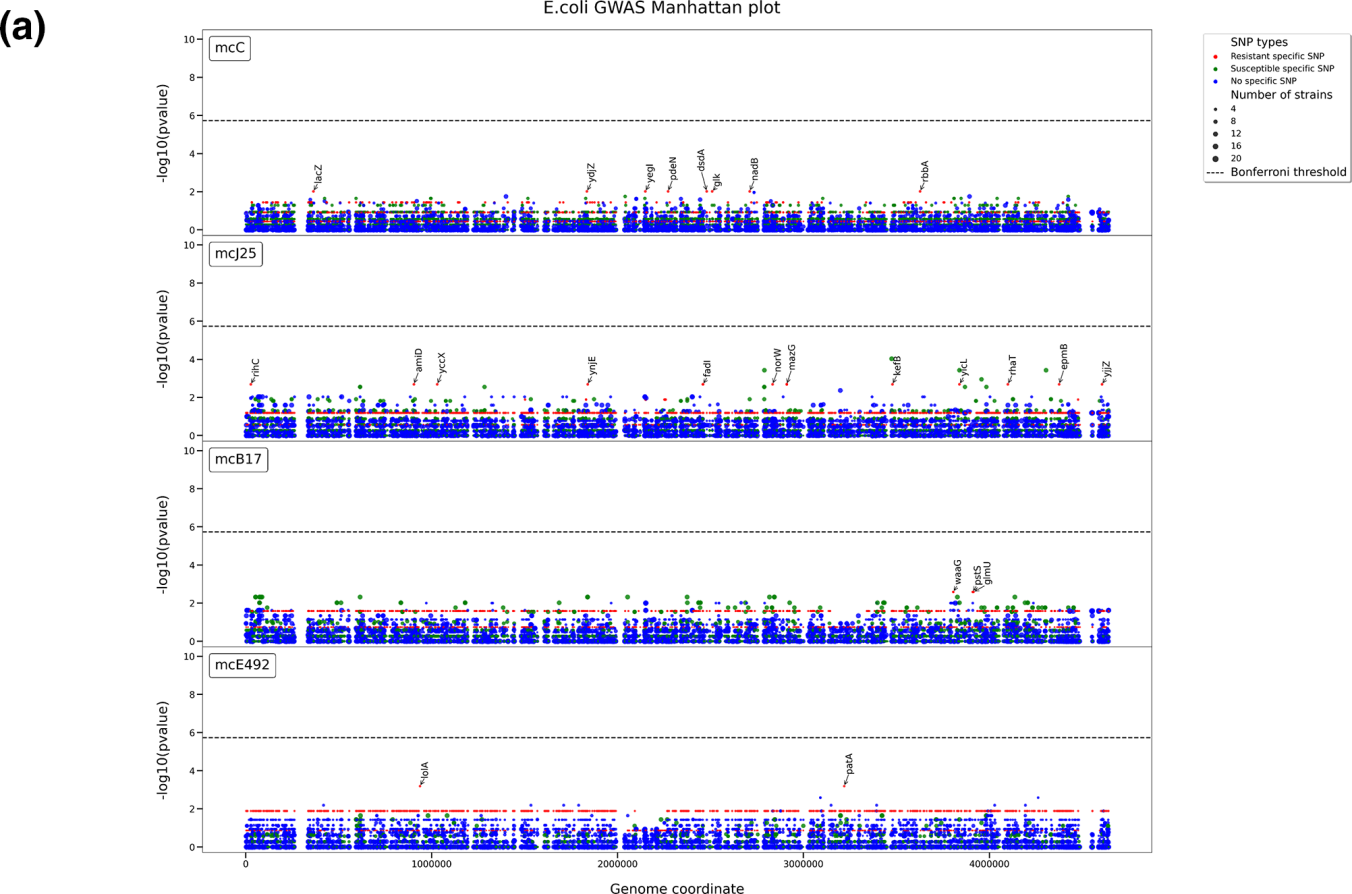

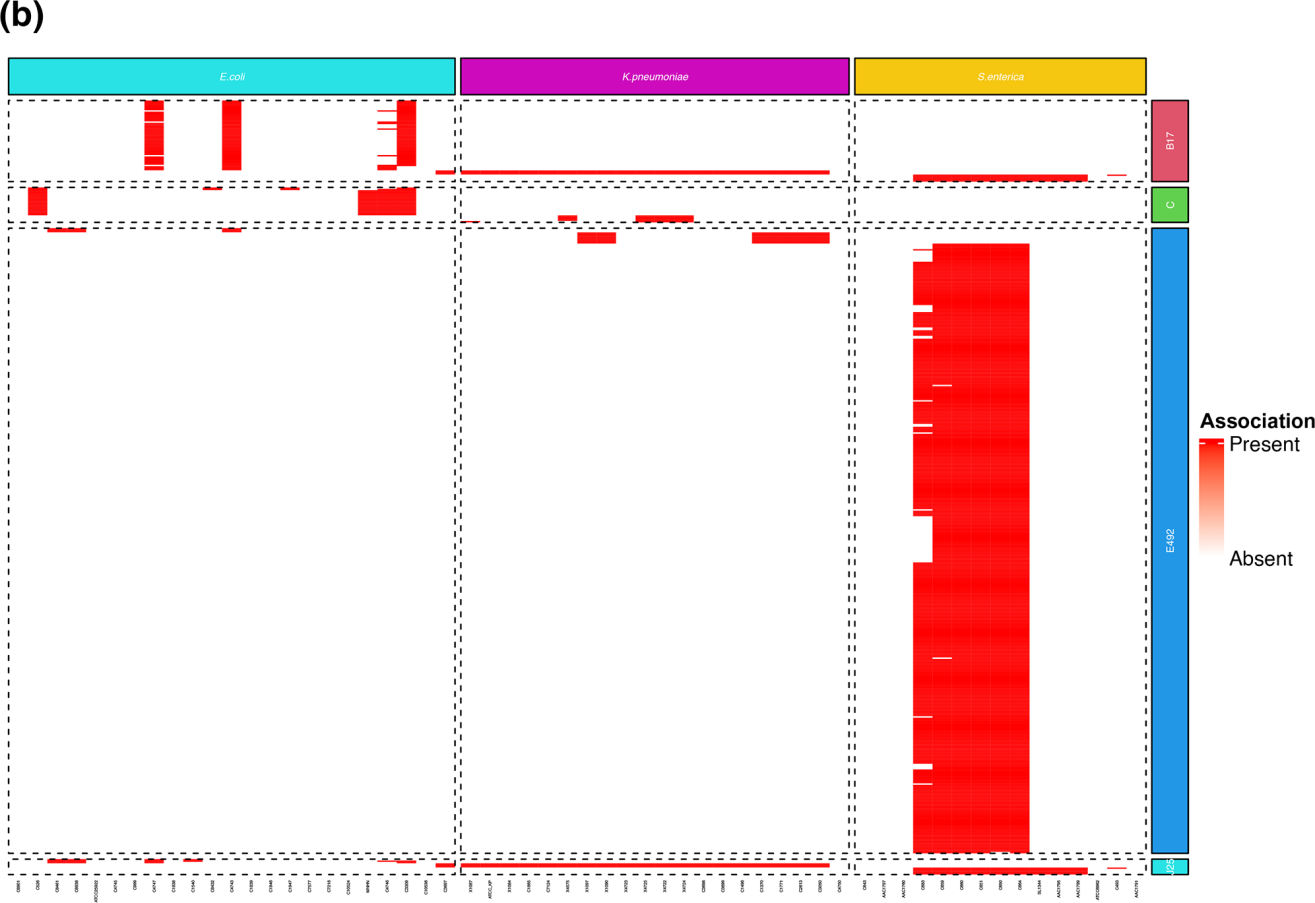

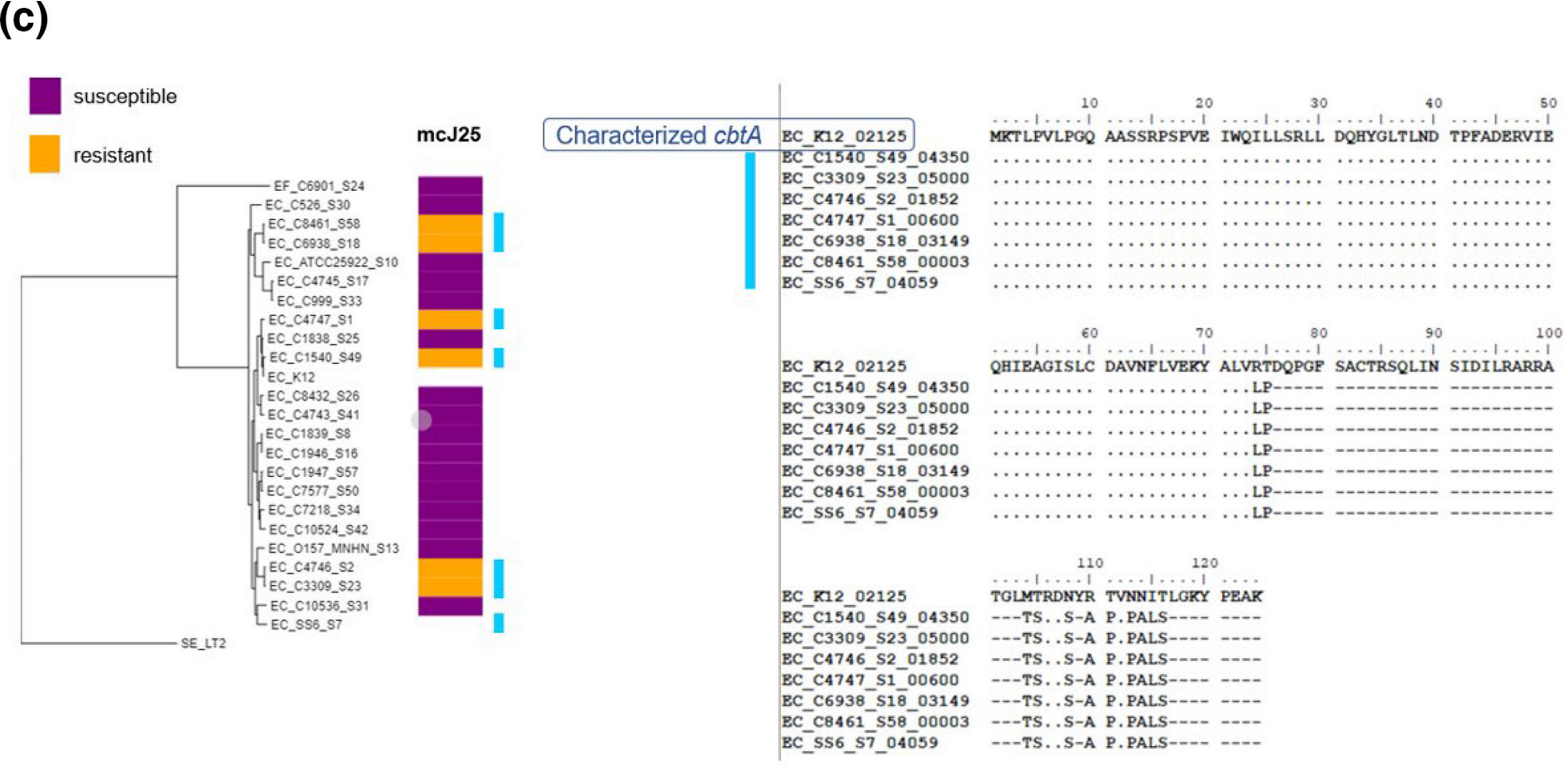
Summary of the GWAS conducted on the *Enterobacteriaceae* strains. (**a**) Manhattan plot summary of the *E. coli* core genome SNP variants associated with microcin resistance (red dots), microcin susceptibility (green dots) and non-specific (blue). The horizontal line represents the corrected *P*-value significance threshold. (**b**) Heatmap summarizing all pangenome genes associated with microcin resistance across all strains detected with the GWAS of gene content. (**c**) Amino acid sequence of the *cbtA* gene associated with MccJ25 resistance in *E. coli* strains.

GWAS was used to detect novel genetic elements associated with the microcin resistance phenotypes. Gene clustering was first performed to determine the core genome (i.e. genes shared by all strains of a given species) and the pangenome (the full complement of genes of a given species) of each species. The core genomes of the studied strains comprised 2636, 3721 and 3842 genes and the pangenomes comprised 14 260, 7113 and 10 425 genes for *E. coli*, *S. enterica* and *K. pneumoniae*, respectively (Fig. S1C). GWAS was then conducted on the core genome SNPs and the gene content of each species (Fig. S2). The core genome SNPs in *E. coli* data and the total pangenome data generated by the GWAS are represented in Fig. 3(a, b) respectively. All data generated by the GWAS can be found in Figs S4–S20. This highlighted 1060 associations of the core genome SNPs (Fig. 3a) and 482 associations of the gene content with microcin resistance phenotypes (Fig. 3b). Most of these associations were found in *S. enterica* strains in relation to their MccE492-resistant phenotypes (1015 core genome SNPs and 368 pangenome genes). More associations were found between the microcin resistance phenotypes and the core-genome SNPs than with the gene content. Yet, they both exhibited a diversity of affected genes. For instance, concerning McC, SNPs associated with resistance in *E. coli* strains were found in the *lacZ*, *pdeN* and *rbbA* genes (Fig. 3a), which fulfil varying functions, *lacZ* coding for β-galactosidase, an enzyme associated with sugar metabolism. On the other hand, *pdeN* is a member of the highly conserved DedA family of inner membrane proteins involved in stress response to pH and temperature. Finally, *rbbA* codes for a ribosome-dependent ATPase associated with the 30S ribosome subunit. Similarly for *E. coli*, sequences associated with resistance to MccJ25 were found in the genes *kefB*, *rihC* and *mazG*. The *mazG* gene is a regulator for the type II toxin–antitoxin system of MazEF, which mediates programmed cell death under nutritional stress. In contrast, *rihC* codes for a ribonuclease hydrolase involved in pyrimidine and purine metabolism. The *kefB* gene codes for glutathione-regulated potassium efflux, which plays a role in protecting the cell from electrophile toxicity. Furthermore, the strongest statistical association was detected in the gene content of *E. coli* with MccJ25 resistance. It was obtained for a specific sequence of the *cbtA* gene, which is found only in resistant strains as opposed to sensitive strains which harbour a wild-type ‘allele’ of *cbtA*. This gene encodes CbtA, a type IV toxin (Fig. 3c).

**Fig. 4.**
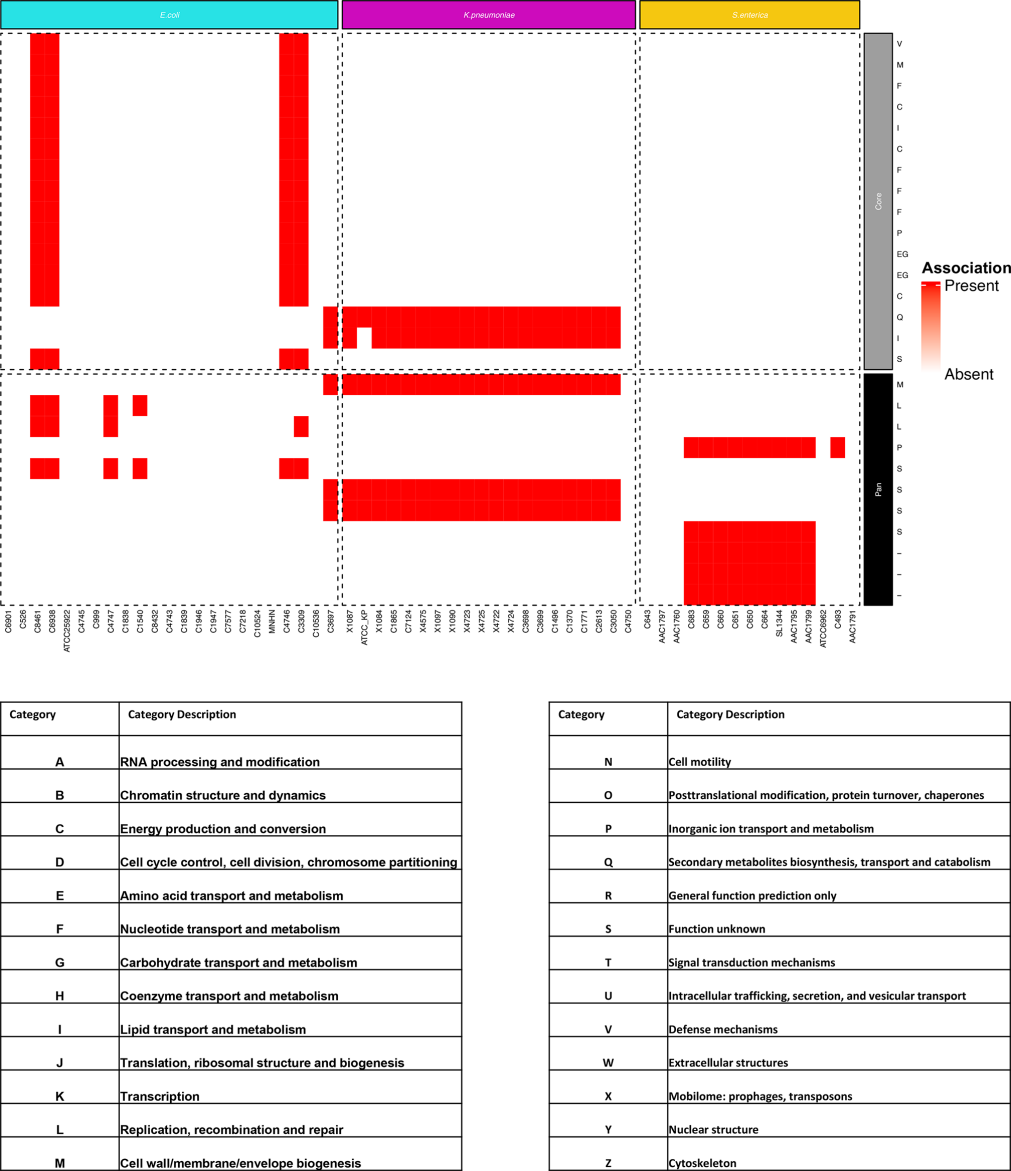
Heatmap of all genetic elements associated with MccJ25 resistance phenotypes. The genetic deviations associated with the MccJ25 resistance phenotype with the COG category of the affected genes as row labels. The description and classes of the COG categories are listed in the table below the heatmap.

It must be noted that some associations were detected in strains already harbouring genetic mutations in genes implicated in the mechanism of action of microcins. For instance, all associations with MccE492 resistance in *S. enterica* were found in strains carrying specific sequences for the genes *fepA* and *cirA* that are involved in MccE492 uptake, which were also highlighted in the gene content GWAS. Additionally, pangenome GWAS revealed a strong association (100 %) between resistance to MccE492 in *S. enterica* and presence of the *mcr-9* gene, which is believed to be involved in resistance to colistin. Given the large number of associations and their diversity, a COG classification was conducted to determine which molecular processes were more affected in the microcin-resistant strains (Figs 4 and S3). In general, genes implicated in cellular processes and metabolism were the most widely detected with the GWAS. Moreover, we compared which COG categories were subject to most mutations. For instance, in the McC-resistant *K. pneumoniae* strains, it was found that they present significantly greater numbers of mutations in genes implicated in cell wall and membrane biogenesis compared to their susceptible counterparts (Fig. S3A). In contrast, pan genes implicated in replication, recombination and repair possessed significantly fewer mutations in the McC-resistant strains compared to their susceptible counterparts (Fig. S3A).

While the gene-targeted approach detected 49 correlations between the microcin resistance phenotypes and mutations in genes implicated in microcin mechanisms of action, the GWAS approach detected an extra 28 without bias correlations, which corresponds to 27.7 % of all observed resistance phenotypes. In total, about 75 % of all microcin-resistant phenotypes were found to be associated with specific genetic sequences. Yet it remains to be seen which are simply statistical associations and which are biologically relevant.

## Discussion

In our previous study [18], we observed microcins to be potent antimicrobial compounds against a collection of natural *Enterobacteriaceae* isolates, including many MDR strains. In the present study, we aimed to explore the underlying genetic elements that could explain the resistance phenotypes observed. It must be noted that ‘resistance’ is not an easily identifiable trait. According to Levin-Reisman *et al*. [49], resistance is a process that can start with tolerance. This means that the relatively easily identifiable AMR genes are simply the tip of the iceberg. In addition, there are different types of resistance: intrinsic, adaptative and acquired [50, 51]. Hence, we have defined a resistance phenotype as the capacity of a strain to grow with the highest tested concentration of a given microcin (50 µg ml^−1^). This, coupled with the small number of genomes tested, does present limits to the statistical significance of the GWAS, yet given the limited data available on microcin resistance, this study can provide additional exploratory insight.

First, we delineated the phylogeny, genotypic profiles, AMR genes and virulence genes of the 54 isolates for two reasons: (i) primarily to identify if there were any closely related strains, which would introduce a greater number of non-specific associations into the GWAS, and (ii) to determine whether relevant genes such as AMR and virulence genes can act as non-specific ‘markers’ to clones resistant to microcins. *E. coli* was the most versatile group with both the highest occurrence of unique STs as well as the most numerous AMR and virulence genes. Interestingly, *K. pneumoniae* came in last, with the lowest number of both AMR and virulence genes, despite presenting similar levels of antibiotic resistance phenotypes and countries of origin.

The presence of multiple AMR genes, involved in resistance to different antibiotics simultaneously, can explain the wide range of antibiotic resistance of the MDR strains in the collection. For instance, *tolC*, widely present in *E. coli* strains, codes for an OM channel required for the function of several efflux pumps such as the AcrA–AcrB–TolC system which is an efflux pump system responsible for cross-resistance to different families of antibiotics. Similarly, for *K. pneumoniae* strains, the *kpnF* gene, which is a member of the major facilitator superfamily (MFS) of antibiotic efflux pumps, is widely present. This is in line with the selection pressure of antibiotic treatment and misuse [52, 53]. Multiple potential cross-resistance phenotypes were predicted between microcins and antibiotics, based on the mechanisms of action of these compounds [11]. Nevertheless, few AMR or virulence genes were associated with the resistance phenotype to microcins; the *tetD* gene, a tetracycline resistance gene, and type III secretion system genes were rare exceptions in the case of McC in *E. coli*.

In a previous study, MccJ25 and rifampicin showed a partial synergy when combined [18], despite both sharing FhuA as an entry point. Concomitantly, based on the observed phenotypic resistances, microcin and antibiotic resistance appeared independent except for a single statistically significant association found between MccJ25 resistance and gentamicin resistance. Yet in this study, no genetic association was observed between gentamicin resistance genes and MccJ25 resistance phenotype.

Following the same line of thought in addressing the statistical limits of the relatively small number of samples (*n*=57), we extended our gene-targeted approach to microcin production genes. Microcin biosynthesis gene clusters usually contain immunity genes providing resistance to the producing strain, so as to not provoke autotoxicity [11]. Sometimes, these immunity genes can be very similar, such as for the immunity genes of both MccV and MccL [54], thus potentially providing co-resistance. Moreover, microcin production was observed to be associated with more invasive phylogroups such as B2 in *E. coli* [55], thus increasing the chance of the microcinogenic strain persisting in an ecological niche and developing resistance. In this case, microcin production genes could be non-specific markers to clones ‘naturally’ resistant to microcins, without a specific biological mechanism behind it. Yet, no microcin biosynthesis genes, with the exception of MccB17 immunity genes, were detected in the tested strains. In fact, given the lack of microcin phenotypic data and therefore the statistical limits of GWAS in this study, it is difficult to reject the hypothesis of non-specific biomarkers. However, given the lack of data on microcin resistance, we have designed the gene-targeted approach to filter out any potential non-specific associations based on biological relevancy, in line with the exploratory nature of this study.

Of all the microcin resistance phenotypes, 48.5 % correlated with mutations in genes implicated in the mechanisms of action of the involved microcins. Most of these mutations occurred on the uptake genes, especially for *fhuA*, *fepA*, *cirA,* and to a lesser extent *ompF* and *ompK36* (in the case of McC). In the case of MccJ25 and *fhuA*, such associations have already been observed in *S. enterica* [34]. Furthermore, *ompK36* is known to be implicated in antibiotic resistance mechanisms in *Klebsiella* [56]. This suggests that OmpK36 could play a role in the uptake of McC, MccB17 and MccJ25. Only two genes coding for microcin final targets, *gyrA* for MccB17 and *aspS* for McC, carried mutations in resistant strains. In the case of the *gyrA* mutations, they all affected Ser^83^, which is known to confer resistance to quinolone antibiotics in *Salmonella* [57, 58]. Interestingly, the mechanism of action of MccB17 in *E. coli* was described to involve the GyrB subunit [21], suggesting that GyrA is central to MccB17 activity in *Salmonella*.

These data imply that genetic variations affecting uptake mechanisms are much more common in bacteria than those affecting the final targets. Indeed, the capacity of *Enterobacteriaceae* to change the permeability of their double layer has already been linked to antibiotic resistance [4, 59]. Hence, it seems plausible that the same can also be true for microcins. Moreover, the over-representation of mutations in *cirA* in MccE492 resistance and in *ompK36* in MccB17 and McC resistance for *K. pneumoniae* strains could be an indicator of the importance of these uptake mechanisms in the antimicrobial activities of these microcins against *Klebsiella*. Essentially just as FepA and OmpF are the main entry points of MccE492, MccB17 and McC in *E. coli*, CirA and OmpK36 could be the main entry points of MccE492, MccB17 and McC in *Klebsiella*. The targeted approach also identified mutations in the *yojI* gene in *E. coli* strains resistant to MccJ25. This gene has already been linked to resistance to MccJ25 [26]. Interestingly, several MccB17-resistant *E. coli* strains revealed the presence of orthologues to MccB17 immunity genes *mcbG* and *mcbF*. Both sequences are flanked by a transposase sequence, suggesting genetic exchange. These genes are observed in the absence of other genes involved in MccB17 biosynthesis. This is an indication that the immunity genes of microcins can be transferred between bacteria without the transfer of the total gene cluster. Such resistance transfer has been observed in the LR05 *E. coli* strain, which harbours the immunity genes of MccV while lacking the other genes for MccV biosynthesis [60]. Note that in this example the immunity genes for MccL and MccV present high sequence similarity, suggesting they could confer co-resistance to both microcins.

The GWAS analysis was not able to identify any significant correlation between resistance to microcins and specific genetic elements. This could be in large part due to the low number of phenotypic data, which are poorly available in the literature. However, some of the associations detected by the GWAS remain biologically relevant and can offer insight into the natural processes of resistance development to microcins. Note that *fepA*, *fhuA* and *ompK36* were found to be associated with microcin resistance in both the gene-targeted and the GWAS approaches. Yet other associations were observed in genes not directly implicated in the microcin mechanisms of action. Previous antibiotic resistance induction experiments [49, 61] have shown that resistance emergence is a cumulative process, whereby bacteria accumulate mutations in genes not directly linked to antibiotic resistance. These mutations would allow the bacteria to tolerate any incoming antibiotic treatment through the persistence process akin to dormancy. Essentially by entering dormancy, the import of extracellular compounds including antibiotics is significantly reduced along with reproduction. Once more favourable environmental conditions are met, reproduction is resumed in the persister cells where additional mutations and/or gene transfers accumulate, until the emergence of mutants capable of growing in the presence of antibiotics. Such a phenomenon was observed in a co-cultivation model of both McC-producing and McC-sensitive *E. coli* strains [62]. The stringent conditions applied through McC provokes persistence in sensitive bacteria through a process requiring ppp(G)ppp and toxin–antitoxin modules. So far, this is the only description of a persistence mechanism made for microcins. This model has been proposed as the process of resistance emergence for chronic infections [63]. In fact, despite the lack of statistical significance of genetic association with resistance, according to our COG analysis, we observed significant accumulation of mutations in genes participating in specific pathways. However, given that we are missing the genomic ancestry of the studied strains, it is difficult to identify which processes accumulated the most mutations. Other potentially biologically relevant associations were observed. For instance, in the case of McC in *E. coli*, SNPs in the *ydjZ* and *pdeN* genes were associated with the resistance phenotype. Both genes are implicated in stress responses and potentially with antibiotic resistance [64, 65]. Concomitantly, *mazG* and *cbtA* were observed to be linked to the MccJ25 resistance phenotype in *E. coli*. The gene *cbtA* encodes a toxin from a type IV toxin–antitoxin system [66], whereas *mazG* codes for a regulator of a type II toxin–antitoxin, which were both linked to bacterial persistence [67, 68], albeit in a controversial way. Interestingly, the biosynthesis gene clusters of lasso peptides other than MccJ25, such as citrocin [69] and rubrinodin [70], harbour toxin–antitoxin encoding genes. These observations suggest that toxin–antitoxin systems and lasso peptides may be functionally related.

In the case of MccE492-resistant *S. enterica* strains, an unusually high number of associations were observed in both the core genome and the pangenome (1015 and 368, respectively). Due to the fact all these strains belong to the same phylogenetic cluster (Fig. 1), many of these associations would be lineage-specific. Essentially, they would be non-specific biomarkers of a clone intrinsically resistant to MccE492. Nevertheless, a more direct molecular biology study is needed to validate these relevant associations.

In conclusion, this study shows that there are no significant associations between antibiotic resistance genes, virulence genes and microcin resistance phenotype. Thus, current antibiotic resistance mechanisms are unlikely to impact the activity of microcins. The most significant indicator of microcin resistance seems to be mutations on the genes implicated in the uptake pathways of these microcins.

## Supplementary Data

Supplementary material 1
