# Supplementary material for: Exploring the genetic basis of natural resistance to microcins
Source: Microb Genom. 2024 Feb 26;10(2):001156. doi: 10.1099/mgen.0.001156 (PMC10926693; doi:10.1099/mgen.0.001156)
Supplement: Supplementary material 1 [file mgen-10-1156-s001.pdf]

Exploring the Genetic Basis of Natural Resistance to Microcins

Soufiane Telhig[1,2], Nguyen Phuong Pham[3], Laila Ben Said[1, 4], Séverine Zirah[2], Marc Ouellette[3], Sylvie Rebuffat[2], Ismail Fliss[1,4]

[1] Food science department, Food and agriculture faculty, Laval University, Quebec, Quebec, Canada

[2] Laboratoire Molécules de Communication et Adaptation des Microorganismes, Muséum national d'Histoire naturelle, Centre national de la Recherche scientifique, Paris, France

[3] Centre de recherche en Infectiologie et Axe des Maladies Infectieuses et Immunitaires du Centre de Recherche du CHU-Québec-Université Laval

[4] Institute of Nutrition and Functional Foods, Laval University, Quebec, Quebec, Canada

Supplementary information

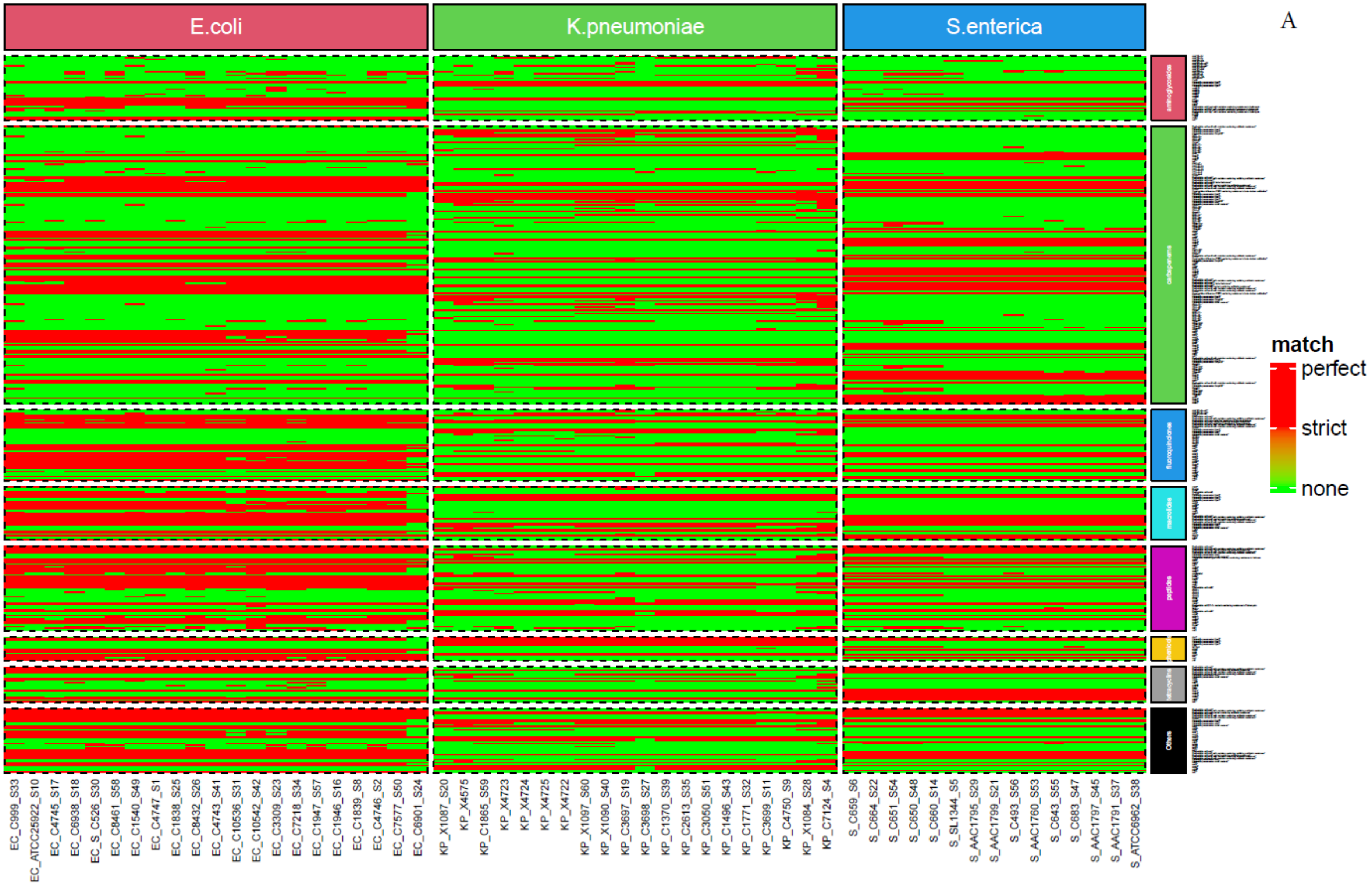

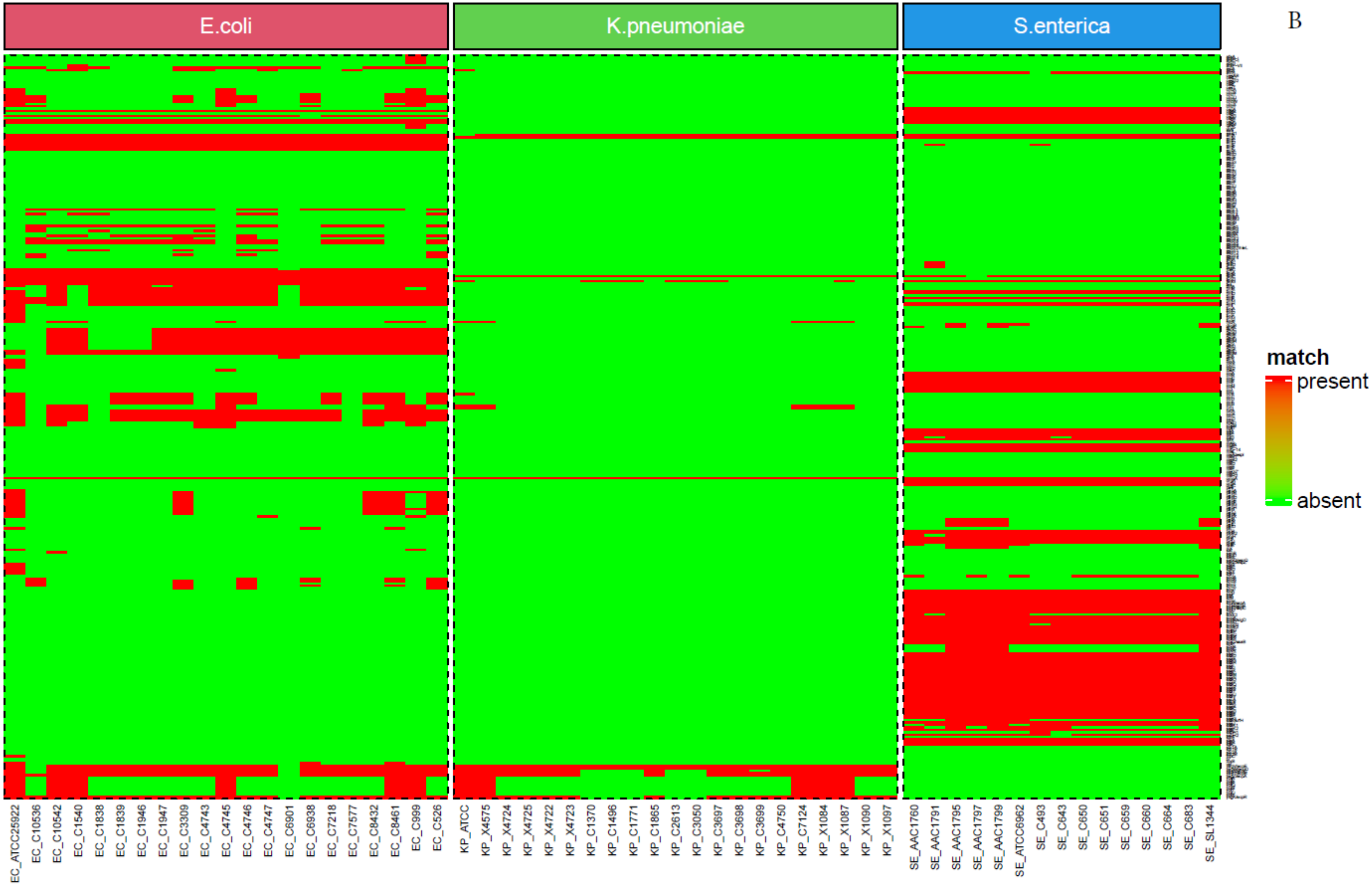
E.coli
K.pneumoniae
S.enterica
B
match
present
absent
EC_ATCC25922
EC_C10536
EC_C10542
EC_C1540
EC_C1838
EC_C1839
EC_C1946
EC_C1947
EC_C3309
EC_C4743
EC_C4745
EC_C4746
EC_C4747
EC_C6901
EC_C6938
EC_C7218
EC_C7577
EC_C8432
EC_C8461
EC_C999
EC_C526
KP_ATCC
KP_X4575
KP_X4724
KP_X4725
KP_X4722
KP_X4723
KP_C1370
KP_C1496
KP_C1771
KP_C1865
KP_C2613
KP_C3050
KP_C3697
KP_C3698
KP_C3699
KP_C4750
KP_C7124
KP_X1084
KP_X1087
KP_X1090
KP_X1097
SE_AAC1760
SE_AAC1791
SE_AAC1795
SE_AAC1797
SE_AAC1799
SE_ATCC6962
SE_C493
SE_C643
SE_C650
SE_C651
SE_C659
SE_C660
SE_C664
SE_C683
SE_SL1344

(C)

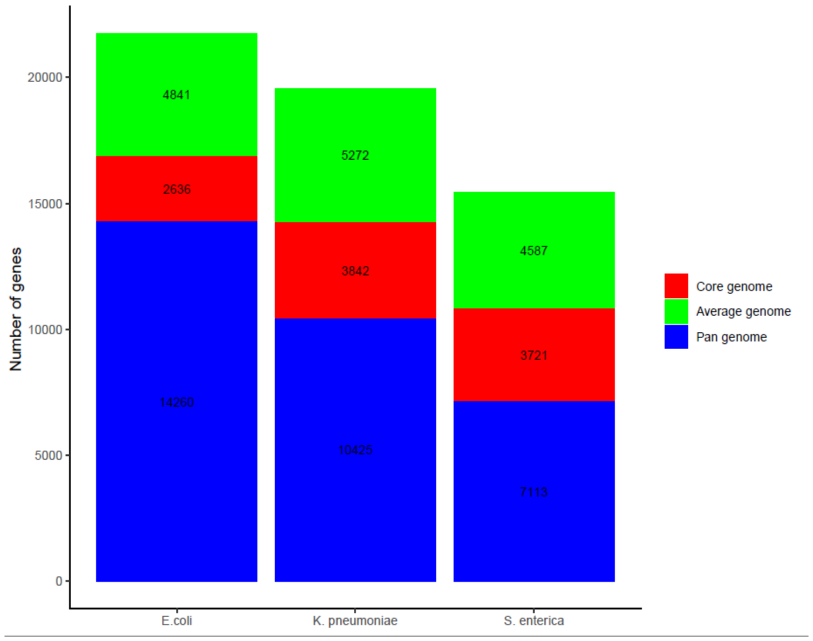

Figure S1 - Overall genomic analysis data. Heatmaps of all detected AMR genes (A) and virulence genes (B) in the studied collection. (C) gene distribution of the sequenced genomes.

Table S1 - List of the major uptake partners of the studied microcins McC, MccJ25, MccB17 and MccE492 and their final targets.

Microcin	Outer membrane (OM) entrance	Uptake	Energy	Final target	References
McC	OmpF	YejABEF		Asp tRNA	(Guijarro et al. 1995; Metlitskaya et al. 2006)
MccJ25	FhuA	SbmA	ExbB-ExbD-TonB	RNA polymerase	(Delgado et al. 2001)
MccB17	OmpF	SbmA		DNA gyrase	(Parks et al. 2007)
MccE492	FepA, Fiu, Cir		ExbB-ExbD-TonB	inner membrane ManYZ	(Huang et al. 2021)

Table S2 - List of natural *E. coli* isolates used in the GWAS study.

[a] human sample, [b] chicken farm sample, *strains with novel housekeeping gene alleles in the MLST analysis.

Antibiotic abbreviations: amoxicillin-clavulanic acid (AMC), amikacin (AMK), ampicillin (AMP), cefotaxime (CTX), ceftazidime (CAZ), chloramphenicol (CHL), ciprofloxacin (CIP), cefoxitin (FOX), gentamicin (GEN), kanamycin (KAN), nalidixic acid (NAL), tetracycline (TET), tobramycin (TOB), sulfamethoxazole (SUL), trimethoprim-sulfamethoxazole (SXT).

**Strain**	**ESBL + other beta-lactamases**	**Phenotype of R**	**ST group**	**Origin**	**Country**
C526	CTX-M-9, TEM-1	AMP/CTX/CAZ/STR/TET/SUL/SXT/NAL	362	Clinical faecal sample[a]	Spain
C999	CTX-M15, TEM-1, OXA-1	AMP/CTX/CAZ/CIP/TET/SUL/SXT/TOB/KAN/STR	131	clinical urine sample[a]	Spain
C1540	CTX-M-15	AMP/CTX/CAZ/NAL/CIP/TET/SUL/SXT/GEN/TOB/STR	10	clinical faecal sample[a]	Spain
C1838	SHV-12	AMP/CTX/CAZ/NAL/TET/SUL/CLO/STR	1137	chicken food[b]	Spain
C1839	CTX-M-14a	AMP/CTX/CAZ/NAL/CIP/TET/STR	359	chicken food[b]	Spain
C1946	CTX-M14a	AMP/CTX/CAZ/NAL/CIP/TET/SUL/SXT/STR/KAN	359	chicken food[b]	Spain
C1947	SHV-12, TEM-1	AMP/CTX/CAZ/NAL/CIP/TET/SUL/CLO/STR	162	chicken food[b]	Spain
C4743	TEM-52c	AMP/CTX/CAZ/KAN/STR	NA	chicken food[b]	Spain
C4745	SHV-12, CTX-M-1	AMP/CTX/CAZ/NAL/TET/SUL	131	chicken food[b]	Spain
C4747	CTX-M-9	AMP/CTX/CAZ/NAL/TET/SUL/SXT/GEN/STR	10	chicken food[b]	Spain
C3309	CMY-2	AMP/CTX/CAZ/AMC/FOX/CIP/STR/CLO/SUL/SXT/TET	57	pork	Mexico
C4746	SHV-12	AMP/CTX/CAZ/CIP/STR/SUL/SXT/TET	57	chicken meat	Spain
C6901	CTX-M-1	AMP/CTX/CAZ/NAL/ STR/ SUL/ SXT/ TET	12029	chicken meat	Tunisia
C7218	CMY-2	AMP/CTX/CAZ/FOX/AMC/STR/CLO/TOB/SXT/TET	155	turtle, faeces	Mexico
C7577		AMP/CTX/CAZ/NAL/STR/TMP/TET	1128	roe deer, faecal sample	Spain
C8432	CTX-M-3	AMP/CTX/CIP/NAL/STR/SUL/SXT/TET	93	chicken, faecal sample	Spain
C8461		AMP/CTX/NAL/STR/SUL/SXT/TET	117* *recA*	chicken faecal sample	Spain
C10524	CTX-M-14	AMP/CTX/CAZ/TET/CIP/STR	453	air pig farm	Spain
C10536	CTX-M-14	AMP/CTX/CAZ/TET/CLO/SXT	2064* *fumC*	air pig farm	Spain
C6938	CMY-2	AMP/CTX/CAZ/AMC/FOX/NAL/CIP/SXT/TET/AMK/CLO	117	chicken, faeces	unknown

Table S3 - List of natural *K. pneumoniae* isolates used in the GWAS study.

[a] human sample, [b] wastewater treatment plant (WWTP), * strains with novel housekeeping gene alleles in the MLST analysis. Antibiotic abbreviations: amoxicillin-clavulanic acid (AMC), amikacin (AMK), ampicillin (AMP), cefotaxime (CTX), ceftazidime (CAZ), chloramphenicol (CHL), ciprofloxacin (CIP), cefoxitin (FOX), gentamicin (GEN), kanamycin (KAN), nalidixic acid (NAL), tetracycline (TET), tobramycin (TOB), sulfamethoxazole (SUL), trimethoprim-sulfamethoxazole (SXT).

**Strain**	**ESBL + other β-lactamases**	**Phenotype of Resistance**	**ST group**	**Origin**	**Country**
C1370	CTX-M-15, SHV-11, OXA-1	AMP/CTX/CAZ/FOX/TOB/KAN/GEN/TET/CLO	433	clinical faecal sample[a]	Spain
C1771	CTX-M-15, SHV-11, OXA-1	AMP/CTX/CAZ/FOX/TOB/KAN/GEN	433	clinical sample[a]	Spain
C1865	CTX-M-15, SHV-28, OXA-1	AMP/CTX/CAZ/FOX/TOB/KAN/TET/CLO/SUL/SXT	15	clinical sample[a]	Spain
C3050	CTX-M-15, SHV-11, OXA-1	AMP/CTX/CAZ/FOX/TOB/KAN/GEN	433	clinical sample[a]	Spain
C1496	CTX-M-15, SHV-11, OXA-1	AMP/CTX/CAZ/FOX/TOB/KAN/GEN	341	clinical sample[a]	Spain
C2613	CTX-M-15, SHV-11, OXA-1	AMP/CTX/CAZ/FOX/CIP/TOB/KAN/GEN/FOS	433	clinical sample[a]	Spain
C3698	CTX-M-3	AMP/CTX/CAZ	54	WWTP[b]	Algeria
C3697	CTX-M-15, TEM-1	AMP/CTX/CAZ/FOX/TET/SUL	147	WWTP[b]	Algeria
C3699	CTX-M-15, SHV-11, OXA-1	AMP/CTX/CAZ/FOX/GEN/SUL	NA	WWTP[b]	Algeria
C4750	SHV-12, TEM-1	TET/SUL/SXT/STR	266* *mdh, pgi, phoE, rpoB* and *tonB*	chicken food[b]	Spain
X4722	CTX-M-15, SHV-28, TEM-1	AMP/CTX/CAZ/FOX/ TET/NAL/CIP/SXT/STR	307	faecal sample, dog	Spain
X4575	CTX-M-15, SHV-1	AMP/CTX/CAZ/CIP	15	faecal sample, dog	Spain
X4724	CTX-M-15, SHV-28	AMP/CTX/CAZ/FOX/TET/CIP/SXT	307	faecal sample, dog	Spain
X4723	SHV1, TEM-1	AMP/CTX/CAZ/FOX/TET/CIP/GEN/STR	307	faecal sample, dog	Spain
X4725	SHV-28, TEM-1	AMP/CTX/CAZ/FOX/TET/CIP/SXT/GEN/STR	307	faecal sample, dog	Spain
X1084	KPC, SHV-1	AMP/CTX/CAZ/FOX/IMP/CIP/SXT/STR/TOB	15	clinical isolate[a]	Portugal
X1087	KPC, SHV-1	AMP/CTX/CAZ/FOX/IMP/CIP/SXT	147* *gapA*	clinical isolate[a]	Portugal
C7124	CTX-M-15, TEM-1b, SHV-28	AMP/CTX/CAZ/FOX/TET/SXT/NAL/TOB	15	Hospital environment	unknown
X1090	CTX-M-15, SHV-27, KPC	AMP/CTX/CAZ/FOX/CIP/SXT/TET/GEN/STR/TOB	11	clinical isolate[a]	Portugal
X1097	CTX-M-15, SHV-11	AMP/CTX/CAZ/FOX/CIP/SXT/TET/TOB	11	clinical isolate[a]	Portugal

Table S4 - List of natural *Salmonella enterica subsp. enterica* isolates used for the GWAS study.

[a] sample from slaughterhouse, [b] sample from human with acute gastroenteritis

Antibiotic abbreviations: amoxicillin-clavulanic acid (AMC), amikacin (AMK), ampicillin (AMP), cefotaxime (CTX), ceftazidime (CAZ), chloramphenicol (CHL), ciprofloxacin (CIP), cefoxitin (FOX), gentamicin (GEN), kanamycin (KAN), nalidixic acid (NAL), tetracycline (TET), tobramycin (TOB), sulfamethoxazole (SUL), trimethoprim-sulfamethoxazole (SXT).

**Strain**	**Serovar**	**Resistance Phenotype**	**ST group**	**Origin**	**Country**
C643	Rissen	AMP/CTX/CAZ/TET/STR/SUL	469	Pig faecal sample[a]	Spain
C659	Virchow	AMP/CTX/CAZ/STR/SUL/SXT/NAL	16	Chicken faecal sample[a]	Spain
C660	Virchow	AMP/CTX/CAZ/STR/SUL/SXT/NAL	16	Chicken faecal sample[a]	Spain
C664	Enteritidis	AMP/CTX/CAZ/TET/STR/SUL/SXT/NAL	16	Chicken faecal sample[a]	Spain
C650	Virchow	AMP/CTX/CAZ/STR/SUL/SXT/NAL	16	faecal sample[b]	Spain
C651	Virchow	AMP/CTX/CAZ/TET/STR/SUL/SXT/NAL	16	faecal sample[b]	Spain
C683	Virchow	AMP/CTX/CAZ/SUL/NAL	16	faecal sample[b]	Spain
C493	Livingstone	AMP/CTX/CAZ/TET/STR/SUL/	457	faecal sample[b]	Spain
AAC 1799	Typhimurium	None	19	Unknown	Canada
AAC 1797	Enteririditis	None	11	Unknown	Canada
AAC 1760	Heidelberg	AMP/AMC/CTX/FOX/CAZ	15	Unknown	Canada
AAC 1791	Kentucky	None	152	Unknown	Canada
AAC 1795	Unknown	None	19	Unknown	Canada
1344	Unknown	None	19	Unknown	France

A

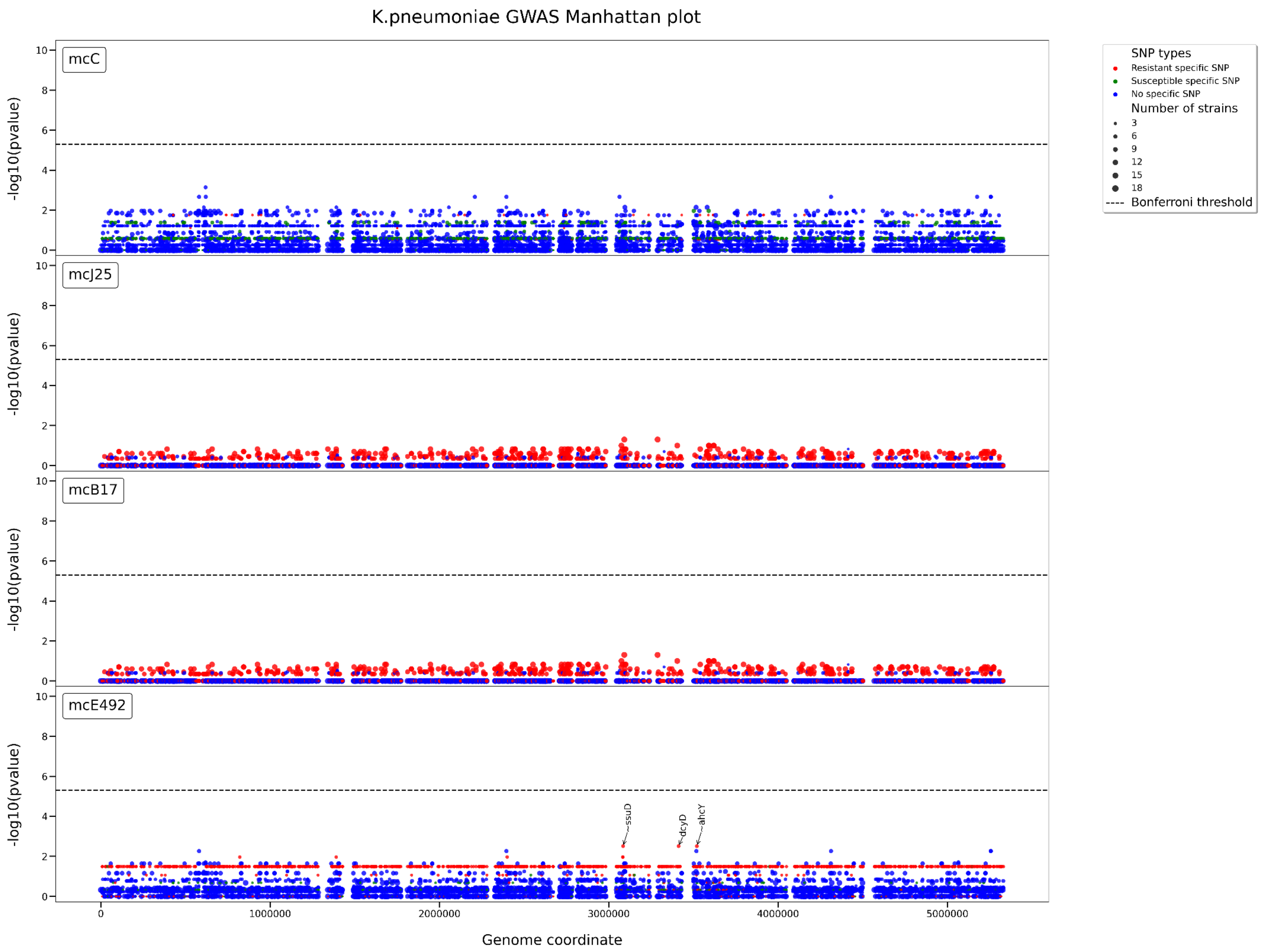
K.pneumoniae GWAS Manhattan plot
mcC
mcJ25
mcB17
mcE492
-log10(pvalue)
Genome coordinate
0
1000000
2000000
3000000
4000000
5000000
ssuD
dcyD
ahcY
SNP types
Resistant specific SNP
Susceptible specific SNP
No specific SNP
Number of strains
3
6
9
12
15
18
Bonferroni threshold

B

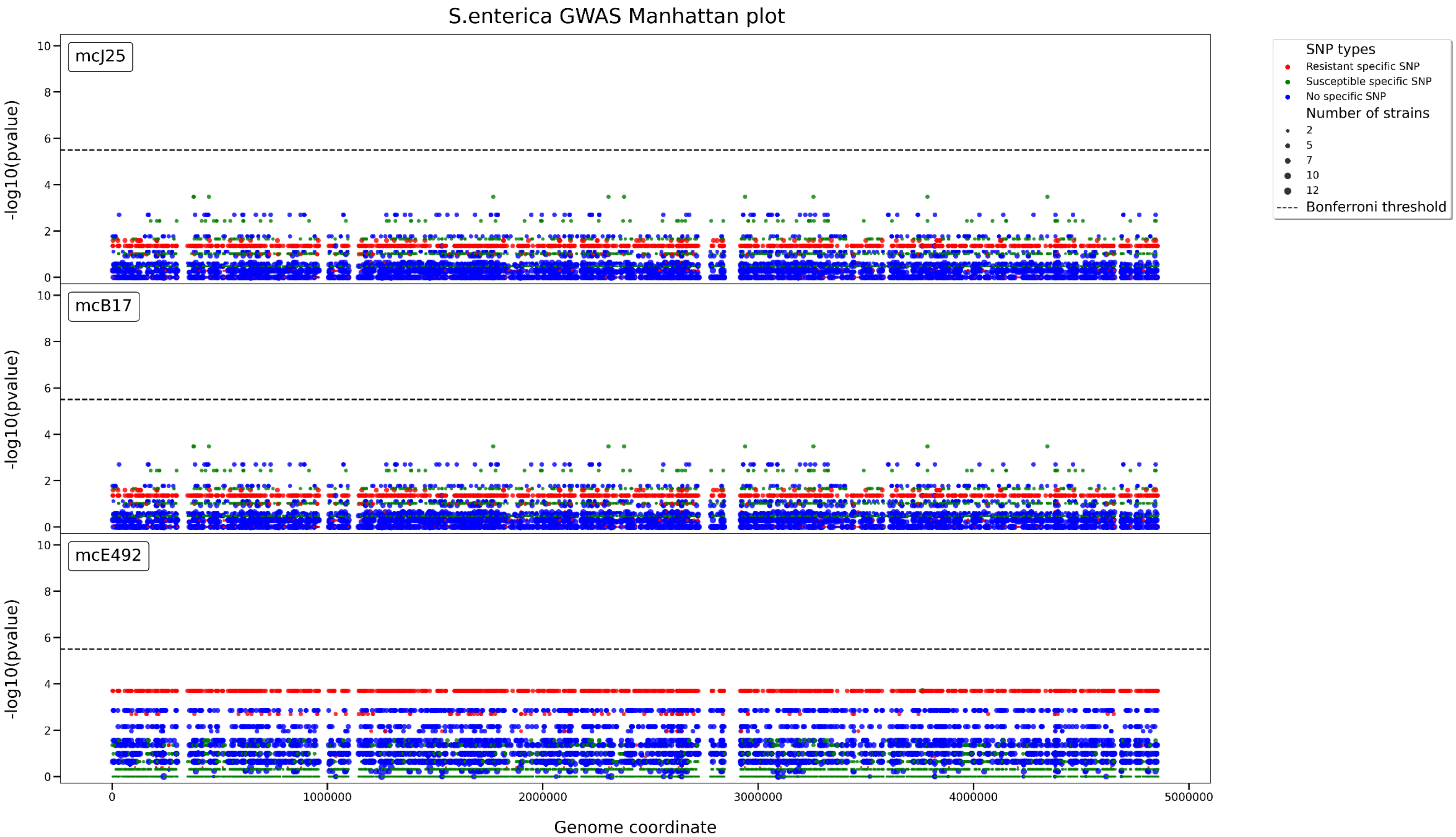

Figure S2: Summary of the genome wide association study (GWAS) conducted on the *Enterobacteriaceae* strains. (A) Manhattan plot summary of the *K. pneumoniae* core genome SNP variants associated with microcin resistance (red dots), microcin susceptibility (green dots) and non-speicific (blue). (B) Manhattan plot summary of the *S. enterica* core genome SNP variants associated with microcin resistance (red dots), microcin susceptibility (green dots) and non-speicific (blue). The horizontal line represents the p-value cut-off = 0.01

A

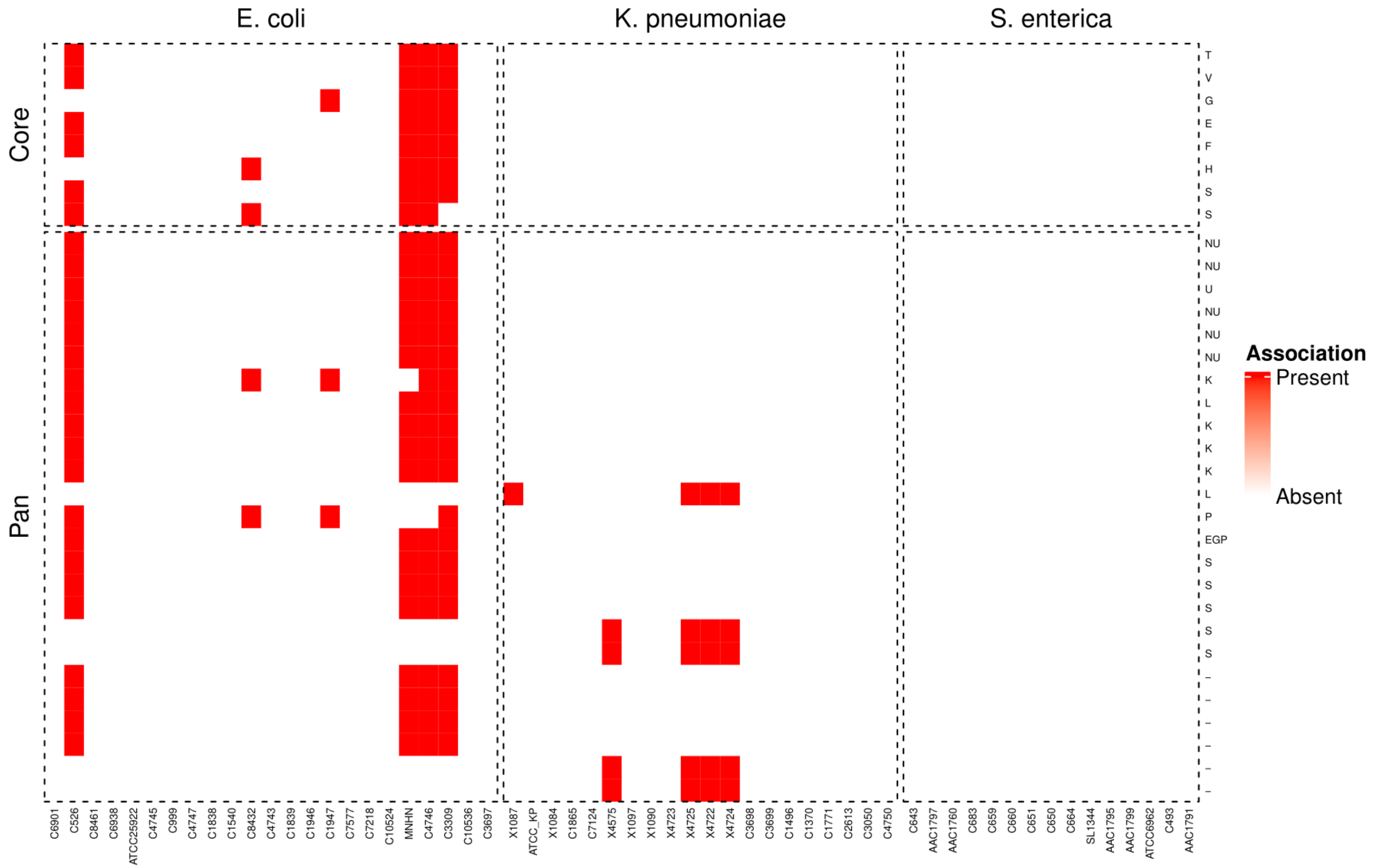

E. coli
K. pneumoniae
S. enterica
Core
Pan
Association
Present
Absent
T
V
G
E
F
H
S
S
NU
NU
U
NU
NU
NU
K
L
K
K
K
L
P
EGP
S
S
S
S
S
C6901
C526
C8461
C6938
ATCC25922
C4745
C999
C4747
C1838
C1540
C8432
C4743
C1839
C1946
C1947
C7577
C7218
C10524
MNHN
C4746
C3309
C10536
C3697
X1087
ATCC_KP
X1084
C1865
C7124
X4575
X1097
X1090
X4723
X4725
X4722
X4724
C3698
C3699
C1496
C1370
C1771
C2613
C3050
C4750
C643
AAC1797
AAC1760
C683
C659
C660
C651
C650
C664
SL1344
AAC1795
AAC1799
ATCC6962
C493
AAC1791

B

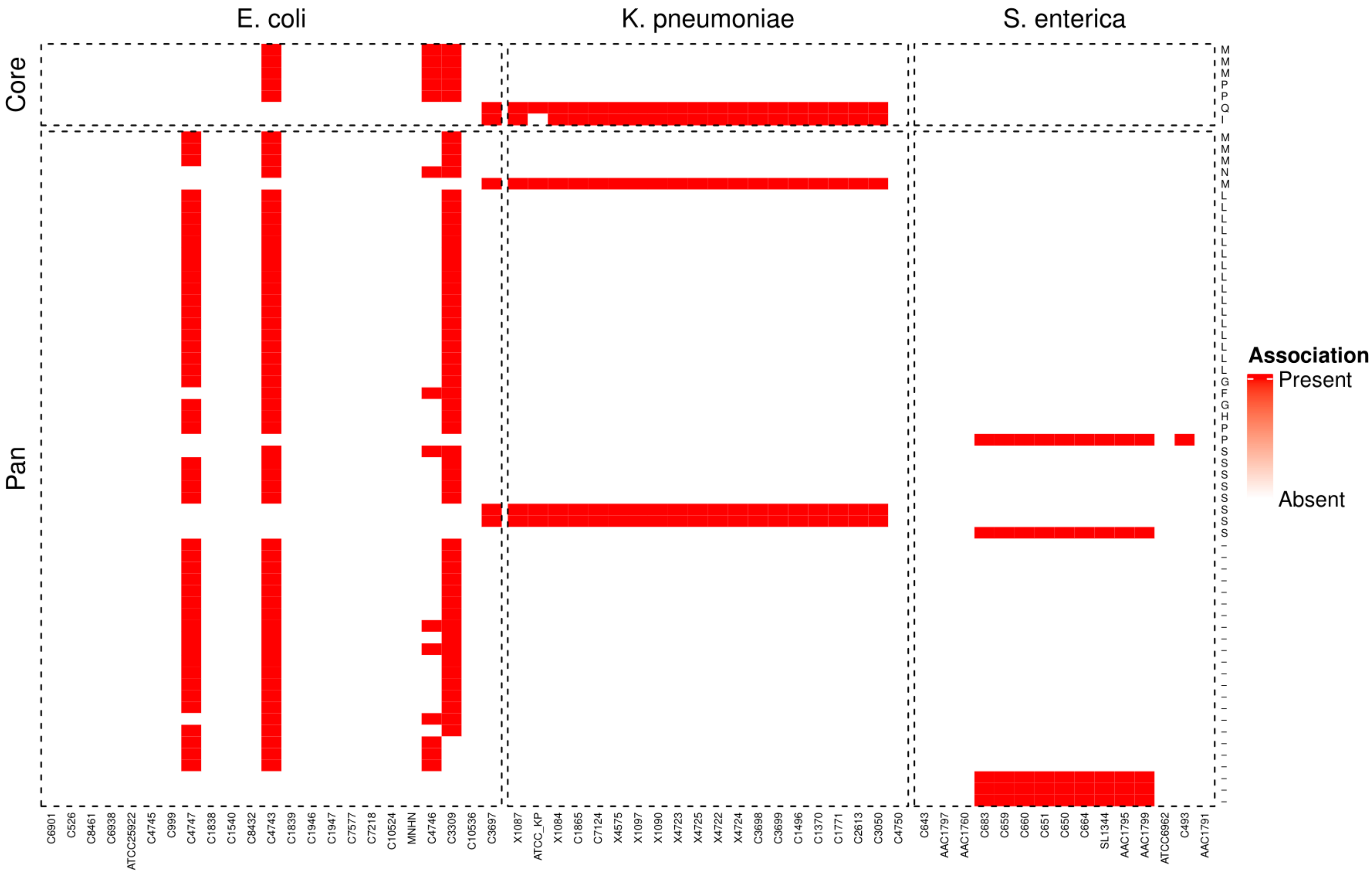
E. coli
K. pneumoniae
S. enterica
Core
Pan
Association
Present
Absent
C6901
C526
C8461
C6938
ATCC25922
C4745
C999
C4747
C1838
C1540
C8432
C4743
C1839
C1946
C1947
C7577
C7218
C10524
MNHN
C4746
C3309
C10536
C3697
X1087
ATCC_KP
X1084
C1865
C7124
X4575
X1097
X1090
X4723
X4725
X4722
X4724
C3698
C3699
C1496
C1370
C1771
C2613
C3050
C4750
C643
AAC1797
AAC1760
C683
C659
C660
C651
C650
C664
SL1344
AAC1795
AAC1799
ATCC6962
C493
AAC1791

C

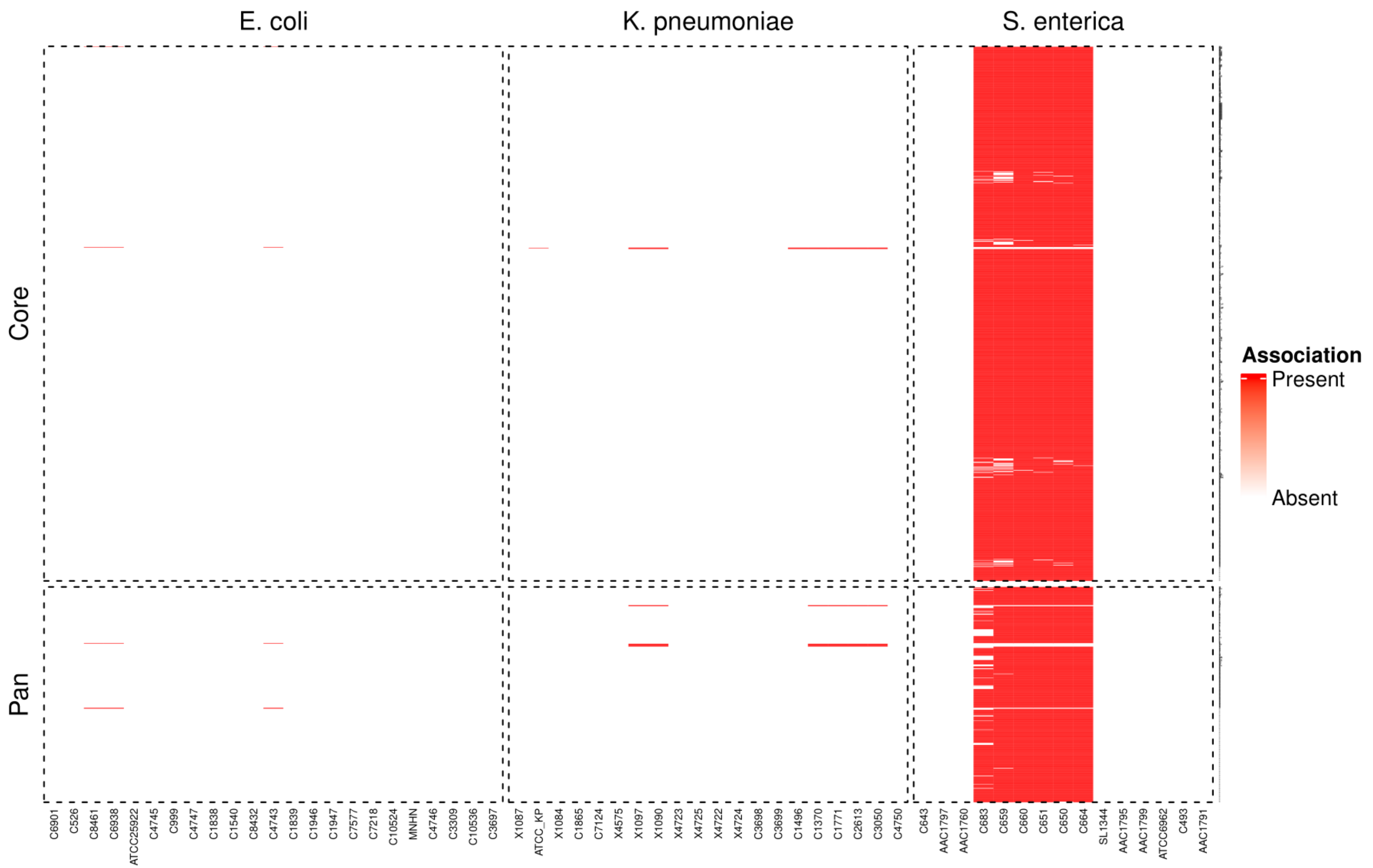

Figure S3 - Heatmap of all genetic elements associated with microcin resistance phenotypes. (A) The genetic deviations associated with McC resistance phenotype with the COG category of the affected genes as row labels; (B) the genetic deviations associated with MccB17 resistance phenotype with the COG category of the affected genes as row labels; (C) the genetic deviations associated with MccE492 resistance phenotype with the COG category of the affected genes as row labels

snp	locus_tag	product	EF_C6901_S24	EC_C526_S30	EC_C8461_S58	EC_C6938_S18	EC_ATCC25922_S10	EC_C4745_S17	EC_C999_S33	EC_C4747_S1	EC_C1838_S25	EC_C1540_S49	EC_C8432_S26	EC_C4743_S41	EC_C1839_S8	EC_C1946_S16	EC_C1947_S57	EC_C7577_S50	EC_C7218_S34	EC_C10524_S42	EC_O157_MNHN_S13	EC_C4746_S2	EC_C3309_S23	EC_C10536_S31	EC_SS6_S7	SUM	SUM R	SUM S	COG_category	Naive_p
EC_K12_00373_p.Glu685Gly	EC_K12_00373	beta-galactosidase	0	0	0	0	0	0	0	0	0	0	0	0	0	0	1	0	0	0	1	1	1	0	0	4	4	0	G	0.009569377990430623
EC_K12_01868_p.Phe96Leu	EC_K12_01868	DedA family protein YdjZ	0	1	0	0	0	0	0	0	0	0	0	0	0	0	0	0	0	0	1	1	1	0	0	4	4	0	S	0.009569377990430623
EC_K12_02197_p.Ile486Met	EC_K12_02197	protein kinase YegI	0	1	0	0	0	0	0	0	0	0	1	0	0	0	0	0	0	0	1	1	0	0	0	4	4	0	S	0.009569377990430623
EC_K12_02313_p.Val411Ile	EC_K12_02313	putative c-di-GMP phosphodiesterase I	0	1	0	0	0	0	0	0	0	0	0	0	0	0	0	0	0	0	1	1	1	0	0	4	4	0	T	0.009569377990430623
EC_K12_02517_p.Gln261Glu	EC_K12_02517	D-serine ammonia-lyase	0	1	0	0	0	0	0	0	0	0	0	0	0	0	0	0	0	0	1	1	1	0	1	5	4	0	E	0.009569377990430623
EC_K12_02539_p.Val125Ile	EC_K12_02539	glucokinase	0	1	0	0	0	0	0	0	0	0	0	0	0	0	0	0	0	0	1	1	1	0	0	4	4	0	F	0.009569377990430623
EC_K12_02742_p.Thr191Lys	EC_K12_02742	L-aspartate oxidase	0	0	0	0	0	0	0	0	0	0	1	0	0	0	0	0	0	0	1	1	1	0	1	5	4	0	H	0.009569377990430623
EC_K12_03679_p.Ala693Ser	EC_K12_03679	ribosome-associated ATPase	0	1	0	0	0	0	0	0	0	0	0	0	0	0	0	0	0	0	1	1	1	0	1	5	4	0	V	0.009569377990430623

Figure S4 - McC Core genome GWAS data for *E. coli* strains. All the SNPs significantly associated with the McC resistance phenotype in the core genomes of the tested *E. coli* strains. Represented are the SNPs, their loci and gene product as predicted in the *E. coli* K12 reference genome for each tested strain. The "SUM", "SUM_R", "SUM_S" are the total number of strains, the number of resistant strains, the number of susceptible strains, respectively, that have the corresponding SNP.

Gene	EF_C6901_S24	EC_C526_S30	EC_C8461_S58	EC_C6938_S18	EC_ATCC25922_S10	EC_C4745_S17	EC_C999_S33	EC_C4747_S1	EC_C1838_S25	EC_C1540_S49	EC_K12	EC_C8432_S26	EC_C4743_S41	EC_C1839_S8	EC_C1946_S16	EC_C1947_S57	EC_C7577_S50	EC_C7218_S34	EC_C10524_S42	EC_O157_MNHN_S13	EC_C4746_S2	EC_C3309_S23	EC_C10536_S31	EC_SS6_S7	SUM	SUM R	SUM S	COG_category	Naive_p
37344_gltS_2.faa	0	1	0	0	0	0	0	0	0	0	0	1	0	0	0	1	0	0	0	0	0	1	0	0	4	4	0	P	0.009569377990430623
37351_tetD.faa	0	1	0	0	0	0	0	0	0	0	0	1	0	0	0	1	0	0	0	0	1	1	0	0	5	5	0	K	0.0021265284423179114
39222_hypothetical_protein.faa	0	1	0	0	0	0	0	0	0	0	0	0	0	0	0	0	0	0	0	1	1	1	0	0	4	4	0	L	0.009569377990430623
40078_hypothetical_protein.faa	0	1	0	0	0	0	0	0	0	0	0	0	0	0	0	0	0	0	0	1	1	1	0	0	4	4	0	-	0.009569377990430623
40083_rcsB_2.faa	0	1	0	0	0	0	0	0	0	0	0	0	0	0	0	0	0	0	0	1	1	1	0	1	5	4	0	K	0.009569377990430623
40092_spaS.faa	0	1	0	0	0	0	0	0	0	0	0	0	0	0	0	0	0	0	0	1	1	1	0	1	5	4	0	NU	0.009569377990430623
40097_hypothetical_protein.faa	0	1	0	0	0	0	0	0	0	0	0	0	0	0	0	0	0	0	0	1	1	1	0	0	4	4	0	-	0.009569377990430623
40098_hypothetical_protein.faa	0	1	0	0	0	0	0	0	0	0	0	0	0	0	0	0	0	0	0	1	1	1	0	1	5	4	0	-	0.009569377990430623
40099_spaL.faa	0	1	0	0	0	0	0	0	0	0	0	0	0	0	0	0	0	0	0	1	1	1	0	1	5	4	0	NU	0.009569377990430623
40100_invA.faa	0	1	0	0	0	0	0	0	0	0	0	0	0	0	0	0	0	0	0	1	1	1	0	1	5	4	0	U	0.009569377990430623
40101_mxiC.faa	0	1	0	0	0	0	0	0	0	0	0	0	0	0	0	0	0	0	0	1	1	1	0	1	5	4	0	S	0.009569377990430623
40103_invF.faa	0	1	0	0	0	0	0	0	0	0	0	0	0	0	0	0	0	0	0	1	1	1	0	1	5	4	0	K	0.009569377990430623
40104_hypothetical_protein.faa	0	1	0	0	0	0	0	0	0	0	0	0	0	0	0	0	0	0	0	1	1	1	0	1	5	4	0	-	0.009569377990430623
40572_pgrR_3.faa	0	1	0	0	0	0	0	0	0	0	0	0	0	0	0	0	0	0	0	1	1	1	0	1	5	4	0	K	0.009569377990430623
40573_hypothetical_protein.faa	0	1	0	0	0	0	0	0	0	0	0	0	0	0	0	0	0	0	0	1	1	1	0	1	5	4	0	S	0.009569377990430623
40574_nepI_1.faa	0	1	0	0	0	0	0	0	0	0	0	0	0	0	0	0	0	0	0	1	1	1	0	1	5	4	0	EGP	0.009569377990430623
42481_yehD_1.faa	0	1	0	0	0	0	0	0	0	0	0	0	0	0	0	0	0	0	0	1	1	1	0	1	5	4	0	NU	0.009569377990430623
42482_yehC_1.faa	0	1	0	0	0	0	0	0	0	0	0	0	0	0	0	0	0	0	0	1	1	1	0	1	5	4	0	NU	0.009569377990430623
42483_yehB_1.faa	0	1	0	0	0	0	0	0	0	0	0	0	0	0	0	0	0	0	0	1	1	1	0	1	5	4	0	NU	0.009569377990430623
43241_hypothetical_protein.faa	0	1	0	0	0	0	0	0	0	0	0	0	0	0	0	0	0	0	0	1	1	1	0	0	4	4	0	S	0.009569377990430623

Figure S5 - McC Pan genome GWAS data for *E. coli* strains. All the genes significantly associated with the McC resistance phenotype in the pangenome or accessory genomes of the tested *E. coli* strains. Represented are the SNPs, their loci and gene product as predicted in the *E. coli* K12 reference genome for each tested strain. The

"SUM", "SUM_R", "SUM_S" are the total number of strains, the number of resistant strains, the number of susceptible strains, respectively, that have the corresponding SNP.

snp	locus_tag	product	EF_C6901_S24	EC_C526_S30	EC_C8461_S58	EC_C6938_S18	EC_ATCC25922_S10	EC_C4745_S17	EC_C999_S33	EC_C4747_S1	EC_C1838_S25	EC_C1540_S49	EC_C8432_S26	EC_C4743_S41	EC_C1839_S8	EC_C1946_S16	EC_C1947_S57	EC_C7577_S50	EC_C7218_S34	EC_C10524_S42	EC_O157_MNHN_S13	EC_C4746_S2	EC_C3309_S23	EC_C10536_S31	EC_SS6_S7	SUM All	SUM R	SUM S	COG_category	Naive_p
EC_K12_03842_p.Ala310Thr	EC_K12_03842	lipopolysaccharide glucosyltransferase I	0	0	0	0	0	0	0	0	0	0	0	1	0	0	0	0	0	0	0	1	1	0	0	3	3	0	M	0.002597402597402594
EC_K12_03842_p.Thr292Ala	EC_K12_03842	lipopolysaccharide glucosyltransferase I	0	0	0	0	0	0	0	0	0	0	0	1	0	0	0	0	0	0	0	1	1	0	0	3	3	0	M	0.002597402597402594
EC_K12_03944_p.Gly147Asp	EC_K12_03944	phosphate ABC transporter periplasmic binding protein	0	0	0	0	0	0	0	0	0	0	0	1	0	0	0	0	0	0	0	1	1	0	0	3	3	0	P	0.002597402597402594
EC_K12_03944_p.Thr296Val	EC_K12_03944	phosphate ABC transporter periplasmic binding protein	0	0	0	0	0	0	0	0	0	0	0	1	0	0	0	0	0	0	0	1	1	0	0	3	3	0	P	0.002597402597402594
EC_K12_03946_p.Val153Ile	EC_K12_03946	fused N-acetylglucosamine-1-phosphate uridyltransferas	0	0	0	0	0	0	0	0	0	0	0	1	0	0	0	0	0	0	0	1	1	0	0	3	3	0	M	0.002597402597402594

Figure S6 - MccB17 Core genome GWAS data for *E. coli* strains. All the SNPs significantly associated with the MccB17 resistance phenotype in the core genomes of the tested *E. coli* strains. Represented are the SNPs, their loci and gene product as predicted in the *E. coli* K12 reference genome for each tested strain. The "SUM", "SUM_R", "SUM_S" are the total number of strains, the number of resistant strains, the number of susceptible strains, respectively, that have the corresponding SNP.

Gene	C6901_S24	C526_S30	C8461_S58	C6938_S18	ATCC25922_S10	C4745_S17	C999_S33	C4747_S1	C1838_S25	C1540_S49	K12	C8432_S26	C4743_S41	C1839_S8	C1946_S16	C1947_S57	C7577_S50	C7218_S34	C10524_S42	O157_MNHN_S13	C4746_S2	C3309_S23	C10536_S31	SS6_S7	SUM	SUM R	SUM S	COG_catego	Naive_p
39537_hypothetical_protein.faa	0	0	0	0	0	0	0	1	0	0	0	0	1	0	0	0	0	0	0	0	0	1	0	0	3	3	0	-	0.002597402597402594
39538_hypothetical_protein.faa	0	0	0	0	0	0	0	1	0	0	0	0	1	0	0	0	0	0	0	0	0	1	0	0	3	3	0	L	0.002597402597402594
39541_hypothetical_protein.faa	0	0	0	0	0	0	0	1	0	0	0	0	1	0	0	0	0	0	0	0	0	1	0	0	3	3	0	-	0.002597402597402594
39542_yagA.faa	0	0	0	0	0	0	0	1	0	0	0	0	1	0	0	0	0	0	0	0	0	1	0	0	3	3	0	L	0.002597402597402594
39690_hypothetical_protein.faa	0	0	0	0	0	0	0	1	0	0	0	0	1	0	0	0	0	0	0	0	0	1	0	0	3	3	0	-	0.002597402597402594
39691_hypothetical_protein.faa	0	0	0	0	0	0	0	1	0	0	0	0	1	0	0	0	0	0	0	0	0	1	0	0	3	3	0	L	0.002597402597402594
39866_hypothetical_protein.faa	0	0	0	0	0	0	0	1	0	0	0	0	1	0	0	0	0	0	0	0	0	1	0	0	3	3	0	-	0.002597402597402594
40009_hypothetical_protein.faa	0	0	0	0	0	0	0	0	0	0	0	0	1	0	0	0	0	0	0	0	1	1	0	1	4	3	0	S	0.002597402597402594
40229_hypothetical_protein.faa	0	0	0	0	0	0	0	1	0	0	0	0	1	0	0	0	0	0	0	0	0	1	0	0	3	3	0	-	0.002597402597402594
40230_hypothetical_protein.faa	0	0	0	0	0	0	0	1	0	0	0	0	1	0	0	0	0	0	0	0	0	1	0	0	3	3	0	-	0.002597402597402594
40231_hypothetical_protein.faa	0	0	0	0	0	0	0	1	0	0	0	0	1	0	0	0	0	0	0	0	0	1	0	0	3	3	0	-	0.002597402597402594
40232_hypothetical_protein.faa	0	0	0	0	0	0	0	1	0	0	0	0	1	0	0	0	0	0	0	0	0	1	0	0	3	3	0	G	0.002597402597402594
40233_hypothetical_protein.faa	0	0	0	0	0	0	0	1	0	0	0	0	1	0	0	0	0	0	0	0	0	1	0	0	3	3	0	M	0.002597402597402594
40234_bshA.faa	0	0	0	0	0	0	0	1	0	0	0	0	1	0	0	0	0	0	0	0	0	1	0	0	3	3	0	M	0.002597402597402594
40235_eptC_1.faa	0	0	0	0	0	0	0	1	0	0	0	0	1	0	0	0	0	0	0	0	0	1	0	0	3	3	0	S	0.002597402597402594
40637_hypothetical_protein.faa	0	0	0	0	0	0	0	0	0	0	0	0	1	0	0	0	0	0	0	0	1	1	0	0	3	3	0	F	0.002597402597402594
40638_hypothetical_protein.faa	0	0	0	0	0	0	0	1	0	0	0	0	1	0	0	0	0	0	0	0	1	1	0	0	4	4	0	-	0.0001367053998632947
40939_crr_1.faa	0	0	0	0	0	0	0	1	0	0	0	0	1	0	0	0	0	0	0	0	0	1	0	0	3	3	0	G	0.002597402597402594
40940_scrK_1.faa	0	0	0	0	0	0	0	1	0	0	0	0	1	0	0	0	0	0	0	0	0	1	0	0	3	3	0	H	0.002597402597402594
40980_hypothetical_protein.faa	0	0	0	0	0	0	0	1	0	0	0	0	1	0	0	0	0	0	0	0	0	1	0	0	3	3	0	-	0.002597402597402594
41008_hypothetical_protein.faa	0	0	0	0	0	0	0	1	0	0	0	0	1	0	0	0	0	0	0	0	1	1	0	0	4	4	0	-	0.0001367053998632947
41048_ISL3_family_transpos...faa	0	0	0	0	0	0	0	1	0	0	0	0	1	0	0	0	0	0	0	0	0	1	0	0	3	3	0	L	0.002597402597402594
41049_ISL3_family_transpos...faa	0	0	0	0	0	0	0	1	0	0	0	0	1	0	0	0	0	0	0	0	0	1	0	0	3	3	0	L	0.002597402597402594
41254_hypothetical_protein.faa	0	0	0	0	0	0	0	1	0	0	0	0	1	0	0	0	0	0	0	0	0	1	0	0	3	3	0	L	0.002597402597402594
41255_hypothetical_protein.faa	0	0	0	0	0	0	0	1	0	0	0	0	1	0	0	0	0	0	0	0	0	1	0	0	3	3	0	-	0.002597402597402594
41261_hypothetical_protein.faa	0	0	0	0	0	0	0	1	0	0	0	0	1	0	0	0	0	0	0	0	0	1	0	0	3	3	0	L	0.002597402597402594
41263_hypothetical_protein.faa	0	0	0	0	0	0	0	1	0	0	0	0	1	0	0	0	0	0	0	0	0	1	0	0	3	3	0	L	0.002597402597402594
41264_IS110_family_transpo...faa	0	0	0	0	0	0	0	1	0	0	0	0	1	0	0	0	0	0	0	0	0	1	0	0	3	3	0	L	0.002597402597402594
41265_IS110_family_transpo...faa	0	0	0	0	0	0	0	1	0	0	0	0	1	0	0	0	0	0	0	0	0	1	0	0	3	3	0	L	0.002597402597402594
41268_hypothetical_protein.faa	0	0	0	0	0	0	0	1	0	0	0	0	1	0	0	0	0	0	0	0	0	1	0	0	3	3	0	L	0.002597402597402594
41273_IS630_family_transpo...faa	0	0	0	0	0	0	0	1	0	0	0	0	1	0	0	0	0	0	0	0	0	1	0	1	4	3	0	L	0.002597402597402594
41274_hypothetical_protein.faa	0	0	0	0	0	0	0	1	0	0	0	0	1	0	0	0	0	0	0	0	0	1	0	0	3	3	0	-	0.002597402597402594
41276_hypothetical_protein.faa	0	0	0	0	0	0	0	1	0	0	0	0	1	0	0	0	0	0	0	0	0	1	0	0	3	3	0	-	0.002597402597402594
41278_hypothetical_protein.faa	0	0	0	0	0	0	0	1	0	0	0	0	1	0	0	0	0	0	0	0	0	1	0	0	3	3	0	-	0.002597402597402594
41279_hypothetical_protein.faa	0	0	0	0	0	0	0	1	0	0	0	0	1	0	0	0	0	0	0	0	0	1	0	0	3	3	0	S	0.002597402597402594
41281_hypothetical_protein.faa	0	0	0	0	0	0	0	1	0	0	0	0	1	0	0	0	0	0	0	0	0	1	0	0	3	3	0	S	0.002597402597402594
41283_hypothetical_protein.faa	0	0	0	0	0	0	0	1	0	0	0	0	1	0	0	0	0	0	0	0	0	1	0	0	3	3	0	M	0.002597402597402594
41284_hypothetical_protein.faa	0	0	0	0	0	0	0	1	0	0	0	0	1	0	0	0	0	0	0	0	0	1	0	0	3	3	0	S	0.002597402597402594
41391_hypothetical_protein.faa	0	0	0	0	0	0	0	1	0	0	0	0	1	0	0	0	0	0	0	0	0	1	0	0	3	3	0	-	0.002597402597402594
42307_hypothetical_protein.faa	0	0	0	0	0	0	0	0	0	0	0	0	1	0	0	0	0	0	0	0	1	1	0	0	3	3	0	-	0.002597402597402594
42381_IS200-IS605_family_t...faa	0	0	0	0	0	0	0	1	0	0	0	0	1	0	0	0	0	0	0	0	0	1	0	0	3	3	0	L	0.002597402597402594
42382_hypothetical_protein.faa	0	0	0	0	0	0	0	1	0	0	0	0	1	0	0	0	0	0	0	0	0	1	0	0	3	3	0	L	0.002597402597402594
42654_ugpB_2.faa	0	0	0	0	0	0	0	1	0	0	0	0	1	0	0	0	0	0	0	0	0	1	0	0	3	3	0	-	0.002597402597402594
42655_ugpB_3.faa	0	0	0	0	0	0	0	1	0	0	0	0	1	0	0	0	0	0	0	0	0	1	0	0	3	3	0	P	0.002597402597402594
42656_hypothetical_protein.faa	0	0	0	0	0	0	0	1	0	0	0	0	1	0	0	0	0	0	0	0	0	1	0	0	3	3	0	L	0.002597402597402594
42657_Single-stranded_DNA-...faa	0	0	0	0	0	0	0	1	0	0	0	0	1	0	0	0	0	0	0	0	0	1	0	0	3	3	0	L	0.002597402597402594
43170_fljB.faa	0	0	0	0	0	0	0	0	0	0	0	0	1	0	0	0	0	0	0	0	1	1	0	0	3	3	0	N	0.002597402597402594
44654_hypothetical_protein.faa	0	0	0	0	0	0	0	1	0	0	0	0	1	0	0	0	0	0	0	0	1	0	0	0	3	3	0	-	0.002597402597402594
45839_hypothetical_protein.faa	0	0	0	0	0	0	0	1	0	0	0	0	1	0	0	0	0	0	0	0	1	0	0	0	3	3	0	-	0.002597402597402594
48903_hypothetical_protein.faa	0	0	0	0	0	0	0	1	0	0	0	0	1	0	0	0	0	0	0	0	1	0	0	0	3	3	0	-	0.002597402597402594

Figure S7 - MccB17 Pan genome GWAS data for *E. coli* strains. All the genes significantly associated with the MccB17 resistance phenotype in the pan or accessory genomes of the tested *E. coli* strains. Represented are the SNPs, their loci and gene product as predicted in the *E. coli* K12 reference genome for each tested strain. The "SUM", "SUM_R", "SUM_S" are the total number of strains, the number of resistant strains, the number of susceptible strains, respectively, that have the corresponding SNP.

snp	locus_tag	product	EF_C6901_S24	EC_C526_S30	EC_C8461_S58	EC_C6938_S18	EC_ATCC25922_S10	EC_C4745_S17	EC_C999_S33	EC_C4747_S1	EC_C1838_S25	EC_C1540_S49	EC_C8432_S26	EC_C4743_S41	EC_C1839_S8	EC_C1946_S16	EC_C1947_S57	EC_C7577_S50	EC_C7218_S34	EC_C10524_S42	EC_O157_MNHN_S13	EC_C4746_S2	EC_C3309_S23	EC_C10536_S31	EC_SS6_S7	SUM All	SUM R	SUM S	COG_category	Naive_p
EC_K12_00030_p.Met112Leu	EC_K12_00030	ribonucleoside hydrolase RihC	0	0	1	1	0	0	0	0	0	0	0	0	0	0	0	0	0	0	0	1	1	0	0	4	4	0	F	0.0020505809979494177
EC_K12_00932_p.Ala10Val	EC_K12_00932	N-acetylmuramoyl-L-alanine amidase D	0	0	1	1	0	0	0	0	0	0	0	0	0	0	0	0	0	0	0	1	1	0	0	4	4	0	V	0.0020505809979494177
EC_K12_01037_p.Thr33Ser	EC_K12_01037	acylphosphatase	0	0	1	1	0	0	0	0	0	0	0	0	0	0	0	0	0	0	0	1	1	0	0	4	4	0	C	0.0020505809979494177
EC_K12_01873_p.Ser74Asn	EC_K12_01873	molybdopterin synthase sulfurtransfera	0	0	1	1	0	0	0	0	0	0	0	0	0	0	0	0	0	0	0	1	1	0	0	4	4	0	M	0.0020505809979494177
EC_K12_02493_p.Ser335Ala	EC_K12_02493	3-ketoacyl-CoA thiolase FadI	0	0	1	1	0	0	0	0	0	0	0	0	0	0	0	0	0	0	0	1	1	0	1	5	4	0	I	0.0020505809979494177
EC_K12_02878_p.Asp140Asn	EC_K12_02878	NADH:flavorubredoxin reductase	0	0	1	1	0	0	0	0	0	0	0	0	0	0	0	0	0	0	0	1	1	0	0	4	4	0	C	0.0020505809979494177
EC_K12_02950_p.Glu106Gly	EC_K12_02950	nucleoside triphosphate pyrophosphohy	0	0	1	1	0	0	0	0	0	0	0	0	0	0	0	0	0	0	0	1	1	0	0	4	4	0	F	0.0020505809979494177
EC_K12_02950_p.Ser103Gly	EC_K12_02950	nucleoside triphosphate pyrophosphohy	0	0	1	1	0	0	0	0	0	0	0	0	0	0	0	0	0	0	0	1	1	0	0	4	4	0	F	0.0020505809979494177
EC_K12_02950_p.Ser109Arg	EC_K12_02950	nucleoside triphosphate pyrophosphohy	0	0	1	1	0	0	0	0	0	0	0	0	0	0	0	0	0	0	0	1	1	0	0	4	4	0	F	0.0020505809979494177
EC_K12_03543_p.Ala564Thr	EC_K12_03543	K(+) : H(+) antiporter KefB	0	0	1	1	0	0	0	0	0	0	0	0	0	0	0	0	0	0	0	1	1	0	0	4	4	0	P	0.0020505809979494177
EC_K12_03872_p.HisLys306AspGlu	EC_K12_03872	putative inner membrane protein	0	0	1	1	0	0	0	0	0	0	0	0	0	0	0	0	0	0	0	1	1	0	0	4	4	0	EG	0.0020505809979494177
EC_K12_04141_p.Ile340Met	EC_K12_04141	rhamnose/lyxose:H(+) symporter	0	0	1	1	0	0	0	0	0	0	0	0	0	0	0	0	0	0	0	1	1	0	1	5	4	0	EG	0.0020505809979494177
EC_K12_04418_p.Asp61Glu	EC_K12_04418	lysine 2,3-aminomutase	0	0	1	1	0	0	0	0	0	0	0	0	0	0	0	0	0	0	0	1	1	0	0	4	4	0	C	0.0020505809979494177
EC_K12_04653_p.Val35Ile	EC_K12_04653	protein YjjZ	0	0	1	1	0	0	0	0	0	0	0	0	0	0	0	0	0	0	0	1	1	0	0	4	4	0	S	0.0020505809979494177

Figure S8 - MccJ25 Core genome GWAS data for *E. coli* strains. All the SNPs significantly associated with the MccJ25 resistance phenotype in the core genomes of the tested *E. coli* strains. Represented are the SNPs, their loci and gene product as predicted in the *E. coli* K12 reference genome for each tested strain. The "SUM", "SUM_R", "SUM_S" are the total number of strains, the number of resistant strains, the number of susceptible strains, respectively, that have the corresponding SNP.

Gene	EF_C6901_S24	EC_C526_S30	EC_C8461_S58	EC_C6938_S18	EC_ATCC25922_S10	EC_C4745_S17	EC_C999_S33	EC_C4747_S1	EC_C1838_S25	EC_C1540_S49	EC_K12	EC_C8432_S26	EC_C4743_S41	EC_C1839_S8	EC_C1946_S16	EC_C1947_S57	EC_C7577_S50	EC_C7218_S34	EC_C10524_S42	EC_O157_MNHN_S13	EC_C4746_S2	EC_C3309_S23	EC_C10536_S31	EC_SS6_S7	SUM	SUM R	SUM S	COG_category	Naive_p
18565_hypothetical_protein.faa	0	0	1	1	0	0	0	1	0	1	0	0	0	0	0	0	0	0	0	0	0	0	0	0	4	4	0	L	0.0020505809979494177
18590_cbtA_1.faa	0	0	1	1	0	0	0	1	0	1	0	0	0	0	0	0	0	0	0	0	1	1	0	1	7	6	0	S	1.3402490182675911e-05
39602_IS5_family_transposa...faa	0	0	1	1	0	0	0	1	0	0	0	0	0	0	0	0	0	0	0	0	0	1	0	0	4	4	0	L	0.0020505809979494177

Figure S9 - MccJ25 Pan genome GWAS data for *E. coli* strains. All the genes significantly associated with the MccJ25 resistance phenotype in the Pan or accessory genomes of the tested *E. coli* strains. Represented are the SNPs, their loci and gene product as predicted in the *E. coli* K12 reference genome for each tested strain. The "SUM", "SUM_R", "SUM_S" are the total number of strains, the number of resistant strains, the number of susceptible strains, respectively, that have the corresponding SNP.

snp	locus_tag	product	EF_C6901_S24	EC_C526_S30	EC_C8461_S58	EC_C6938_S18	EC_ATCC25922_S10	EC_C4745_S17	EC_C999_S33	EC_C4747_S1	EC_C1838_S25	EC_C1540_S49	EC_C8432_S26	EC_C4743_S41	EC_C1839_S8	EC_C1946_S16	EC_C1947_S57	EC_C7577_S50	EC_C7218_S34	EC_C10524_S42	EC_O157_MNHN_S13	EC_C4746_S2	EC_C3309_S23	EC_C10536_S31	EC_SS6_S7	SUM All	SUM R	SUM S	COG_category	Naive_p
EC_K12_00957_p.Gly48Asp	EC_K12_00957	outer membrane lipoprotein carrier protein	0	0	1	1	0	0	0	0	0	0	0	1	0	0	0	0	0	0	0	0	0	0	0	3	3	0	M	0.0006493506493506496
EC_K12_03255_p.Glu4Ala	EC_K12_03255	putrescine aminotransferase	0	0	1	1	0	0	0	0	0	0	0	1	0	0	0	0	0	0	0	0	0	0	0	3	3	0	H	0.0006493506493506496

Figure S10 - MccE492 Core genome GWAS data for *E. coli* strains. All the SNPs significantly associated with the MccE492 resistance phenotype in the core genomes of the tested *E. coli* strains. Represented are the SNPs, their loci and gene product as predicted in the *E. coli* K12 reference genome for each tested strain. The "SUM", "SUM_R", "SUM_S" are the total number of strains, the number of resistant strains, the number of susceptible strains, respectively, that have the corresponding SNP.

Gene	EF_C6901_S24	EC_C526_S30	EC_C8461_S58	EC_C6938_S18	EC_ATCC25922_S10	EC_C4745_S17	EC_C999_S33	EC_C4747_S1	EC_C1838_S25	EC_C1540_S49	EC_K12	EC_C8432_S26	EC_C4743_S41	EC_C1839_S8	EC_C1946_S16	EC_C1947_S57	EC_C7577_S50	EC_C7218_S34	EC_C10524_S42	EC_O157_MNHN_S13	EC_C4746_S2	EC_C3309_S23	EC_C10536_S31	EC_SS6_S7	SUM	SUM R	SUM S	COG_category	Naive_p
44981_hypothetical_protein.faa	0	0	1	1	0	0	0	0	0	0	0	0	1	0	0	0	0	0	0	0	0	0	0	0	3	3	0	-	0.0006493506493506496
47333_hypothetical_protein.faa	0	0	1	1	0	0	0	0	0	0	0	0	1	0	0	0	0	0	0	0	0	0	0	1	4	3	0	-	0.0006493506493506496
48535_fepA_2.faa	0	0	1	1	0	0	0	0	0	0	0	0	1	0	0	0	0	0	0	0	0	0	0	0	3	3	0	P	0.0006493506493506496

Figure S11 - MccE492 Pan genome GWAS data for *E. coli* strains. All the genes significantly associated with the MccE492 resistance phenotype in the pan or accessory genomes of the tested *E. coli* strains. Represented are the SNPs, their loci and gene product as predicted in the *E. coli* K12 reference genome for each tested strain. The "SUM", "SUM_R", "SUM_S" are the total number of strains, the number of resistant strains, the number of susceptible strains, respectively, that have the corresponding SNP.

Gene	KP_C3697_S19	KP_X1087_S20	KP_ATCC_S52	KP_X1084_S28	KP_C1865_S59	KP_C7124_S4	KP_C10142_S12	KP_HS11286	KP_X1097_S60	KP_X1090_S40	KP_C10146_S36	KP_C10132_S44	KP_C10138_S3	KP_C10131_S46	KP_C3698_S27	KP_C3699_S11	KP_C1496_S43	KP_C1370_S39	KP_C1771_S32	KP_C2613_S35	KP_C3050_S51	KP_C4750_S9	SUM	SUM R	SUM S	COG_category	Naive_p
2263_hypothetical_protein.faa	0	0	1	0	0	0	1	0	0	0	0	1	1	1	0	0	0	0	0	0	0	0	5	4	0	-	0.00309597523219814
2264_smc_2.faa	0	0	1	0	0	0	1	0	0	0	0	1	1	1	0	0	0	0	0	0	0	0	5	4	0	NT	0.00309597523219814
2266_hypothetical_protein.faa	0	0	1	0	0	0	1	0	0	0	0	1	1	1	0	0	0	0	0	0	0	0	5	4	0	M	0.00309597523219814
2267_hypothetical_protein.faa	0	0	1	0	0	0	1	0	0	0	0	1	1	1	0	0	0	0	0	0	0	0	5	4	0	S	0.00309597523219814
2284_hypothetical_protein.faa	0	0	1	0	0	0	1	0	0	0	0	1	1	1	0	0	0	0	0	0	0	0	5	4	0	-	0.00309597523219814
6275_hypothetical_protein.faa	0	0	0	0	0	0	1	0	0	0	0	1	1	1	0	0	0	0	0	0	0	0	4	4	0	-	0.00309597523219814
9092_hypothetical_protein.faa	0	0	0	0	0	0	1	0	0	0	0	1	1	1	0	0	0	0	0	0	0	0	4	4	0	-	0.00309597523219814
9093_hypothetical_protein.faa	0	0	0	0	0	0	1	0	0	0	0	1	1	1	0	0	0	0	0	0	0	0	4	4	0	S	0.00309597523219814
9094_hypothetical_protein.faa	0	0	0	0	0	0	1	0	0	0	0	1	1	1	0	0	0	0	0	0	0	0	4	4	0	S	0.00309597523219814
9095_hypothetical_protein.faa	0	0	0	0	0	0	1	0	0	0	0	1	1	1	0	0	0	0	0	0	0	0	4	4	0	S	0.00309597523219814
9096_hypothetical_protein.faa	0	0	0	0	0	0	1	0	0	0	0	1	1	1	0	0	0	0	0	0	0	0	4	4	0	S	0.00309597523219814
9097_hypothetical_protein.faa	0	0	0	0	0	0	1	0	0	0	0	1	1	1	0	0	0	0	0	0	0	0	4	4	0	-	0.00309597523219814
9099_hypothetical_protein.faa	0	0	0	0	0	0	1	0	0	0	0	1	1	1	0	0	0	0	0	0	0	0	4	4	0	-	0.00309597523219814
10927_IS481_family_transpo...faa	0	1	0	0	0	0	0	0	0	0	0	1	1	1	0	0	0	0	0	0	0	0	4	4	0	L	0.00309597523219814

Figure S12 - McC Pan genome GWAS data for *E. coli* strains. All the genes significantly associated with the McC resistance phenotype in the pan or accessory genomes of the tested *K. pneumoniae* strains. Represented are the SNPs, their loci and gene product as predicted in the *K. pneumoniae* HS11286 reference genome for each tested strain. The "SUM", "SUM_R", "SUM_S" are the total number of strains, the number of resistant strains, the number of susceptible strains, respectively, that have the corresponding SNP.

snp	locus_tag	product	KP_C3697_S19	KP_X1087_S20	KP_ATCC_S52	KP_X1084_S28	KP_C1865_S59	KP_C7124_S4	KP_C10142_S12	KP_X1097_S60	KP_X1090_S40	KP_C10146_S36	KP_C10132_S44	KP_C10138_S3	KP_C10131_S46	KP_C3698_S27	KP_C3699_S11	KP_C1496_S43	KP_C1370_S39	KP_C1771_S32	KP_C2613_S35	KP_C3050_S51	KP_C4750_S9	SUM	SUM R	SUM S	COG_category	Naive_p
KP_HS11286_03111_p.Val227Ala	KP_HS11286_03111	acetaldehyde dehydrogenase	1	1	1	1	1	1	1	1	1	1	1	1	1	1	1	1	1	1	1	1	0	20	19	0	Q	0.04999999999999996
KP_HS11286_03306_p.Tyr336His	KP_HS11286_03306	putative fatty acid desaturase	1	1	0	1	1	1	1	1	1	1	1	1	1	1	1	1	1	1	1	1	0	19	19	0	I	0.04999999999999996

Figure S13 - MccB17 and MccJ25 Core genome GWAS data for *K. pneumoniae* strains. All the SNPs significantly associated with both MccB17 and MccJ25 resistance phenotypes in the core genomes of the tested *K. pneumoniae* strains. Represented are the SNPs, their loci and gene product as predicted in the *K. pneumoniae* HS111286 reference genome for each tested strain. The "SUM", "SUM_R", "SUM_S" are the total number of strains, the number of resistant strains, the number of susceptible strains, respectively, that have the corresponding SNP.

Gene	KP_C3697_S19	KP_X1087_S20	KP_ATCC_S52	KP_X1084_S28	KP_C1865_S59	KP_C7124_S4	KP_C10142_S12	KP_HS11286	KP_X1097_S60	KP_X1090_S40	KP_C10146_S36	KP_C10132_S44	KP_C10138_S3	KP_C10131_S46	KP_C3698_S27	KP_C3699_S11	KP_C1496_S43	KP_C1370_S39	KP_C1771_S32	KP_C2613_S35	KP_C3050_S51	KP_C4750_S9	SUM	SUM R	SUM S	COG_category	Naive_p
93808_hypothetical_protein.faa	1	1	1	1	1	1	1	1	1	1	1	1	1	1	1	1	1	1	1	1	1	0	21	19	0	S	0.04999999999999996
94583_OmpK36_porin.faa	1	1	1	1	1	1	1	1	1	1	1	1	1	1	1	1	1	1	1	1	1	0	21	19	0	M	0.04999999999999996
94958_lipoprotein.faa	1	1	1	1	1	1	1	1	1	1	1	1	1	1	1	1	1	1	1	1	1	0	21	19	0	S	0.04999999999999996

Figure S14 - MccB17 and MccJ25 Pan genome GWAS data for *K. pneumoniae* strains. All the genes significantly associated with both MccB17 and MccJ25 resistance phenotypes in the pan or accessory genomes of the tested *K. pneumoniae* strains. Represented are the SNPs, their loci and gene product as predicted in the *K. pneumoniae* HS111286 reference genome for each tested strain. The "SUM", "SUM_R", "SUM_S" are the total number of strains, the number of resistant strains, the number of susceptible strains, respectively, that have the corresponding SNP.

snp	locus_tag	product	KP_C3697_S19	KP_X1087_S20	KP_ATCC_S52	KP_X1084_S28	KP_C1865_S59	KP_C7124_S4	KP_C10142_S12	KP_X1097_S60	KP_X1090_S40	KP_C10146_S36	KP_C10132_S44	KP_C10138_S3	KP_C10131_S46	KP_C3698_S27	KP_C3699_S11	KP_C1496_S43	KP_C1370_S39	KP_C1771_S32	KP_C2613_S35	KP_C3050_S51	KP_C4750_S9	SUM	SUM R	SUM S	COG_category	Naive_p
KP_HS11286_03103_p.Gly98Glu	KP_HS11286_03103	putative lavin-dependent oxidoreductase	0	0	0	0	0	0	0	1	1	0	0	0	0	0	0	1	1	1	1	1	0	7	7	0	C	0.0030959752321981426
KP_HS11286_03436_p.Val232Glu	KP_HS11286_03436	D-cysteine desulfhydrase	0	0	1	0	0	0	0	1	1	0	0	0	0	0	0	1	1	1	1	1	0	8	7	0	E	0.0030959752321981426
KP_HS11286_03522_p.Phe128Tyr	KP_HS11286_03522	putative S-adenosylhomocysteine hydrolase	0	0	0	0	0	0	0	1	1	0	0	0	0	0	0	1	1	1	1	1	0	7	7	0	H	0.0030959752321981426

Figure S15 – MccE492 Core genome GWAS data for *K. pneumoniae* strains. All the SNPs significantly associated with MccE492 resistance phenotypes in the core genomes of the tested *K. pneumoniae* strains. Represented are the SNPs, their loci and gene product as predicted in the *K. pneumoniae* HS111286 reference genome for each tested strain. The "SUM", "SUM_R", "SUM_S" are the total number of strains, the number of resistant strains, the number of susceptible strains, respectively, that have the corresponding SNP.

Gene	KP_C3697_S19	KP_X1087_S20	KP_ATCC_S52	KP_X1084_S28	KP_C1865_S59	KP_C7124_S4	KP_C10142_S12	KP_HS11286	KP_X1097_S60	KP_X1090_S40	KP_C10146_S36	KP_C10132_S44	KP_C10138_S3	KP_C10131_S46	KP_C3698_S27	KP_C3699_S11	KP_C1496_S43	KP_C1370_S39	KP_C1771_S32	KP_C2613_S35	KP_C3050_S51	KP_C4750_S9	SUM	SUM R	SUM S	COG_category	Naive_p
33831_hypothetical_protein.faa	0	0	0	0	0	0	0	0	1	1	0	0	0	0	0	0	0	1	1	1	1	0	6	6	0	L	0.010835913312693499
33832_amiC_3.faa	0	0	0	0	0	0	0	0	1	1	0	0	0	0	0	0	0	1	1	1	1	0	6	6	0	E	0.010835913312693499
33833_hypothetical_protein.faa	0	0	0	0	0	0	0	0	1	1	0	0	0	0	0	0	0	1	1	1	1	0	6	6	0	K	0.010835913312693499
33834_gsiD_3.faa	0	0	0	0	0	0	0	0	1	1	0	0	0	0	0	0	0	1	1	1	1	0	6	6	0	EP	0.010835913312693499
33835_gsiC_4.faa	0	0	0	0	0	0	0	0	1	1	0	0	0	0	0	0	0	1	1	1	1	0	6	6	0	P	0.010835913312693499
33836_gsiB_2.faa	0	0	0	0	0	0	0	0	1	1	0	0	0	0	0	0	0	1	1	1	1	0	6	6	0	E	0.010835913312693499
33837_fmdA.faa	0	0	0	0	0	0	0	0	1	1	0	0	0	0	0	0	0	1	1	1	1	0	6	6	0	C	0.010835913312693499
33838_gsiA_3.faa	0	0	0	0	0	0	0	0	1	1	0	0	0	0	0	0	0	1	1	1	1	0	6	6	0	P	0.010835913312693499

Figure S16 - MccE492 Pan genome GWAS data for *K. pneumoniae* strains. All the genes significantly associated with MccE492 resistance phenotypes in the pan or accessory genomes of the tested *K. pneumoniae* strains. Represented are the SNPs, their loci and gene product as predicted in the *K. pneumoniae* HS111286 reference genome for each tested strain. The "SUM", "SUM_R", "SUM_S" are the total number of strains, the number of resistant strains, the number of susceptible strains, respectively, that have the corresponding SNP.

snp	locus_tag	product	SE_C643_S55	SE_AAC1797_S45	SE_AAC1760_S53	SE_C683_S47	SE_C659_S6	SE_C660_S14	SE_C651_S54	SE_C650_S48	SE_C664_S22	SE_SL1344_S5	SE_AAC1795_S29	SE_AAC1799_S21	SE_ATCC6962_S38	SE_C493_S56	SE_AAC1791_S37	SUM	SUM R	SUM S	COG_category	Naive_p
SE_LT2_00356_p.Arg207Leu	SE_LT2_00356	fimbrial assembly protein	1	1	1	0	0	0	0	0	0	0	0	0	1	0	1	5	0	5	M	0.00033300033300033327
SE_LT2_00356_p.Thr249Ile	SE_LT2_00356	fimbrial assembly protein	1	1	1	0	0	0	0	0	0	0	0	0	1	0	1	5	0	5	M	0.00033300033300033327
SE_LT2_00416_p.Glu261Asp	SE_LT2_00416	exonuclease subunit SbcD	1	1	1	0	0	0	0	0	0	0	0	0	1	0	1	5	0	5	L	0.00033300033300033327
SE_LT2_01772_p.Ala298Glu	SE_LT2_01772	putative LysR family transcriptional regulator	1	1	1	0	0	0	0	0	0	0	0	0	1	0	1	5	0	5	K	0.00033300033300033327
SE_LT2_02322_p.Val95Ala	SE_LT2_02322	PTS fructose transporter subunit IIBC	1	1	1	0	0	0	0	0	0	0	0	0	1	0	1	5	0	5	G	0.00033300033300033327
SE_LT2_02393_p.Arg186Lys	SE_LT2_02393	MR-MLE family protein	1	1	1	0	0	0	0	0	0	0	0	0	1	0	1	5	0	5	M	0.00033300033300033327
SE_LT2_02931_p.Gln64Arg	SE_LT2_02931	hydroxyglutarate oxidase	1	1	1	0	0	0	0	0	0	0	0	0	1	0	1	5	0	5	S	0.00033300033300033327
SE_LT2_03253_p.Ser121Asn	SE_LT2_03253	putative cytoplasmic protein	1	1	1	0	0	0	0	0	0	0	0	0	1	0	1	5	0	5	H	0.00033300033300033327
SE_LT2_03795_p.Ile188Val	SE_LT2_03795	hypothetical protein	1	1	1	0	0	0	0	0	0	0	0	0	1	0	1	5	0	5	M	0.00033300033300033327

Figure S17 - MccB17 and MccJ25 Core genome GWAS data for *S. enterica* strains. All the SNPs significantly associated with both MccB17 and MccJ25 sensitivity phenotypes in the core genomes of the tested *S. enterica* strains. Represented are the SNPs, their loci and gene product as predicted in the *S. enterica* LT2 reference genome for each tested strain. The "SUM", "SUM_R", "SUM_S" are the total number of strains, the number of resistant strains, the number of susceptible strains, respectively, that have the corresponding SNP.

Gene	SE_C643_S55	SE_AAC1797_S45	SE_AAC1760_S53	SE_C683_S47	SE_C659_S6	SE_C660_S14	SE_C651_S54	SE_C650_S48	SE_C664_S22	SE_LT2	SE_SL1344_S5	SE_AAC1795_S29	SE_AAC1799_S21	SE_ATCC6962_S38	SE_C493_S56	SE_AAC1791_S37	SUM	SUM R	SUM S	COG_category	Naive_p
64719_fhuA	0	0	0	1	1	1	1	1	1	1	1	1	1	0	1	0	11	10	0	P	0.00033300033300033327
65540_Gifsy-2_prophage_pro..	0	0	0	1	1	1	1	1	1	1	1	1	1	0	0	0	10	9	0	-	0.001998001998002002
66076_putative_cytoplasmic..	0	0	0	1	1	1	1	1	1	1	1	1	1	0	0	0	10	9	0	S	0.001998001998002002
68414_hypothetical_protein	0	0	0	1	1	1	1	1	1	1	1	1	1	0	0	0	10	9	0	-	0.001998001998002002
68857_hypothetical_protein	0	0	0	1	1	1	1	1	1	1	1	1	1	0	0	0	10	9	0	-	0.001998001998002002

Figure S18 - MccB17 and MccJ25 Pan genome GWAS data for *S. enterica* strains. All the genes significantly associated with both MccB17 and MccJ25 resistance phenotypes in the pan or accessory genomes of the tested *S. enterica* strains. Represented are the SNPs, their loci and gene product as predicted in the *S. enterica* LT2 reference genome for each tested strain. The "SUM", "SUM_R", "SUM_S" are the total number of strains, the number of resistant strains, the number of susceptible strains, respectively, that have the corresponding SNP.

snp	locus_tag	product	SE_C643_S55	SE_AAC1797_S45	SE_AAC1760_S53	SE_C683_S47	SE_C659_S6	SE_C660_S14	SE_C651_S54	SE_C650_S48	SE_C664_S22	SE_SL1344_S5	SE_AAC1795_S29	SE_AAC1799_S21	SE_ATCC6962_S38	SE_C493_S56	SE_AAC1791_S37	SUM	SUM R	SUM S	COG_category	Naive_p
SE_LT2_00003_p.Ala202Val	SE_LT2_00003	homoserine kinase	0	0	0	1	1	1	1	1	1	0	0	0	0	0	0	6	6	0	F	0.0001998001998002
SE_LT2_00004_p.Pro126Leu	SE_LT2_00004	threonine synthase	0	0	0	1	1	1	1	1	1	0	0	0	0	0	0	6	6	0	E	0.0001998001998002
SE_LT2_00024_p.Leu210Phe	SE_LT2_00024	outer membrane usher protein	0	0	0	1	1	1	1	1	1	0	0	0	0	0	0	6	6	0	NU	0.0001998001998002
SE_LT2_00024_p.Leu401Val	SE_LT2_00024	outer membrane usher protein	0	0	0	1	1	1	1	1	1	0	0	0	0	0	0	6	6	0	NU	0.0001998001998002
SE_LT2_00028_p.Arg213Trp	SE_LT2_00028	fimbrial chaparone	0	0	0	1	1	1	1	1	1	0	0	0	0	0	0	6	6	0	NU	0.0001998001998002
SE_LT2_00028_p.Gln4Pro	SE_LT2_00028	fimbrial chaparone	0	0	0	1	1	1	1	1	1	0	0	0	0	0	0	6	6	0	NU	0.0001998001998002
SE_LT2_00029_p.Ala214Val	SE_LT2_00029	hypothetical protein	0	0	0	1	1	1	1	1	1	0	0	0	0	0	0	6	6	0	O	0.0001998001998002
SE_LT2_00031_p.Phe140Met	SE_LT2_00031	transcriptional regulator	0	0	0	1	1	1	1	1	1	0	0	0	0	0	0	6	6	0	K	0.0001998001998002
SE_LT2_00052_p.Asp210Asn	SE_LT2_00052	putative nitrite reductase	0	0	0	1	1	1	1	1	1	0	0	0	0	0	0	6	6	0	-	0.0001998001998002
SE_LT2_00065_p.Asn2Ser	SE_LT2_00065	triphosphoribosyl-dephospho-CoA s	0	0	0	1	1	1	1	1	1	0	0	0	0	0	0	6	6	0	F	0.0001998001998002
SE_LT2_00065_p.Asp183Asn	SE_LT2_00065	triphosphoribosyl-dephospho-CoA s	0	0	0	1	1	1	1	1	1	0	0	0	0	0	0	6	6	0	F	0.0001998001998002
SE_LT2_00065_p.Glu21Asp	SE_LT2_00065	triphosphoribosyl-dephospho-CoA s	0	0	0	1	1	1	1	1	1	0	0	0	0	0	0	6	6	0	F	0.0001998001998002
SE_LT2_00069_p.ThrAlaIleGln25	SE_LT2_00069	carbamoyl phosphate synthase smal	0	0	0	1	1	1	1	1	1	0	0	0	0	0	0	6	6	0	F	0.0001998001998002
SE_LT2_00072_p.Ser22Cys	SE_LT2_00072	phenylacetic acid degradation prote	0	0	0	1	1	1	1	1	1	0	0	0	0	0	0	6	6	0	S	0.0001998001998002
SE_LT2_00073_p.Ser183Asn	SE_LT2_00073	carnitinyl-CoA dehydratase	0	0	0	1	1	1	1	1	1	0	0	0	0	0	0	6	6	0	H	0.0001998001998002
SE_LT2_00074_p.Ala158Ser	SE_LT2_00074	crotonobetaine/carnitine-CoA ligase	0	0	0	1	1	1	1	1	1	0	0	0	0	0	0	6	6	0	IQ	0.0001998001998002
SE_LT2_00074_p.Ala162Thr	SE_LT2_00074	crotonobetaine/carnitine-CoA ligase	0	0	0	1	1	1	1	1	1	0	0	0	0	0	0	6	6	0	IQ	0.0001998001998002
SE_LT2_00077_p.Pro226Gln	SE_LT2_00077	L-carnitine:gamma-butyrobetaine a	0	0	0	1	1	1	1	1	1	0	0	0	0	0	0	6	6	0	M	0.0001998001998002
SE_LT2_00078_p.Ala106Ser	SE_LT2_00078	electron transfer flavoprotein	0	0	0	1	1	1	1	1	1	0	0	0	0	0	0	6	6	0	C	0.0001998001998002
SE_LT2_00078_p.Val87Ile	SE_LT2_00078	electron transfer flavoprotein	0	0	0	1	1	1	1	1	1	0	0	0	0	0	0	6	6	0	C	0.0001998001998002
SE_LT2_00079_p.Ala135Val	SE_LT2_00079	nitrogen fixation protein FixB	0	0	0	1	1	1	0	1	1	0	0	0	0	0	0	5	5	0	C	0.00199800199800199
SE_LT2_00079_p.Pro141Ser	SE_LT2_00079	nitrogen fixation protein FixB	0	0	0	1	1	1	1	1	1	0	0	0	0	0	0	6	6	0	C	0.0001998001998002
SE_LT2_00099_p.Ile46Val	SE_LT2_00099	bifunctional tRNA pseudouridine(32	0	0	0	1	1	1	1	1	1	0	0	0	0	0	0	6	6	0	J	0.0001998001998002
SE_LT2_00100_p.Ala298Thr	SE_LT2_00100	RNA polymerase-binding ATPase	0	0	0	1	0	1	1	1	1	0	0	0	0	0	0	5	5	0	K	0.00199800199800199
SE_LT2_00102_p.Gln177Lys	SE_LT2_00102	putative secreted protein	0	0	0	1	1	1	1	1	1	0	0	0	0	0	0	6	6	0	S	0.0001998001998002
SE_LT2_00110_p.Pro319Leu	SE_LT2_00110	thiamine/thiamine pyrophosphate	0	0	0	1	1	1	1	1	1	0	0	0	0	0	0	6	6	0	P	0.0001998001998002
SE_LT2_00110_p.Val314Ile	SE_LT2_00110	thiamine/thiamine pyrophosphate	0	0	0	1	1	1	1	1	1	0	0	0	0	0	0	6	6	0	P	0.0001998001998002
SE_LT2_00113_p.Gly314Ser	SE_LT2_00113	transcriptional regulator SgrR	0	0	0	1	1	1	1	1	1	0	0	0	0	0	0	6	6	0	K	0.0001998001998002
SE_LT2_00117_p.Glu260Lys	SE_LT2_00117	3-isopropylmalate dehydratase larg	0	0	0	1	1	1	1	1	1	0	0	0	0	0	0	6	6	0	E	0.0001998001998002
SE_LT2_00122_p.Thr203Ile	SE_LT2_00122	valine sensitive acetolactate syntha	0	0	0	1	1	1	1	1	1	0	0	0	0	0	0	6	6	0	H	0.0001998001998002
SE_LT2_00124_p.Thr181Lys	SE_LT2_00124	DNA-binding transcriptional regulat	0	0	0	1	1	1	1	1	1	0	0	0	0	0	0	6	6	0	K	0.0001998001998002
SE_LT2_00125_p.Asn67Ser	SE_LT2_00125	cell division/cell wall cluster transcr	0	0	0	1	1	1	1	1	1	0	0	0	0	0	0	6	6	0	K	0.0001998001998002
SE_LT2_00129_p.Ser72Ala	SE_LT2_00129	UDP-N-acetylmuramoyl-L-alanyl-D-	0	0	0	1	1	1	1	1	1	0	0	0	0	0	0	6	6	0	M	0.0001998001998002
SE_LT2_00130_p.Leu24Phe	SE_LT2_00130	UDP-N-acetylmuramoyl-tripeptide-	0	0	0	1	1	1	1	1	1	0	0	0	0	0	0	6	6	0	M	0.0001998001998002
SE_LT2_00142_p.Arg45Lys	SE_LT2_00142	putative inner membrane protein	0	0	0	1	0	1	1	1	1	0	0	0	0	0	0	5	5	0	S	0.00199800199800199

SE_LT2_00145_p.AlaLeu130SerPı	SE_LT2_00145	8-oxo-dGTP diphosphatase	0	0	0	0	1	1	1	1	1	0	0	0	0	0	0	5	5	0	L	0.00199800199800199
SE_LT2_00145_p.Asp113Glu	SE_LT2_00145	8-oxo-dGTP diphosphatase	0	0	0	1	1	1	1	1	1	0	0	0	0	0	0	6	6	0	L	0.0001998001998002
SE_LT2_00149_p.Thr90Lys	SE_LT2_00149	GMP reductase	0	0	0	1	1	1	1	1	1	0	0	0	0	0	0	6	6	0	F	0.0001998001998002
SE_LT2_00150_p.Ser214Ala	SE_LT2_00150	type IV pilin biogenesis protein	0	0	0	1	1	1	1	1	1	0	0	0	0	0	0	6	6	0	NU	0.0001998001998002
SE_LT2_00156_p.GluSer60LysThr	SE_LT2_00156	alpha-N-arabinofuranosidase	0	0	0	1	1	1	1	1	1	0	0	0	0	0	0	6	6	0	G	0.0001998001998002
SE_LT2_00162_p.Ala197Glu	SE_LT2_00162	pyruvate dehydrogenase complex d	0	0	0	1	1	1	1	1	1	0	0	0	0	0	0	6	6	0	C	0.0001998001998002
SE_LT2_00174_p.Arg224Cys	SE_LT2_00174	DeoR family transcriptional regulato	0	0	0	1	1	1	1	1	1	0	0	0	0	0	0	6	6	0	K	0.0001998001998002
SE_LT2_00183_p.Val140Ile	SE_LT2_00183	ABC transporter permease	0	0	0	1	1	1	1	1	1	0	0	0	0	0	0	6	6	0	V	0.0001998001998002
SE_LT2_00184_p.Met6Ile	SE_LT2_00184	fimbrial protein StiH	0	0	0	1	1	1	1	1	1	0	0	0	0	0	0	6	6	0	NU	0.0001998001998002
SE_LT2_00185_p.Arg140Gln	SE_LT2_00185	fimbrial assembly protein	0	0	0	1	1	1	1	1	1	0	0	0	0	0	0	6	6	0	NU	0.0001998001998002
SE_LT2_00185_p.Asp382Gly	SE_LT2_00185	fimbrial assembly protein	0	0	0	1	1	1	1	1	1	0	0	0	0	0	0	6	6	0	NU	0.0001998001998002
SE_LT2_00185_p.Asp562Asn	SE_LT2_00185	fimbrial assembly protein	0	0	0	1	1	1	1	1	1	0	0	0	0	0	0	6	6	0	NU	0.0001998001998002
SE_LT2_00185_p.Asp836Glu	SE_LT2_00185	fimbrial assembly protein	0	0	0	1	1	1	1	1	1	0	0	0	0	0	0	6	6	0	NU	0.0001998001998002
SE_LT2_00185_p.Ser132Asn	SE_LT2_00185	fimbrial assembly protein	0	0	0	1	1	1	1	1	1	0	0	0	0	0	0	6	6	0	NU	0.0001998001998002
SE_LT2_00186_p.Pro118Leu	SE_LT2_00186	long polar fimbrial chaperone LpfB	0	0	0	0	1	1	1	1	1	0	0	0	0	0	0	5	5	0	M	0.00199800199800199
SE_LT2_00195_p.AlaThr254ProGl	SE_LT2_00195	putative glutamyl t-RNA synthetase	0	0	0	1	0	1	1	1	1	0	0	0	0	0	0	5	5	0	J	0.00199800199800199
SE_LT2_00199_p.Ala207Thr	SE_LT2_00199	ATP-dependent helicase	0	0	0	1	1	1	1	1	1	0	0	0	0	0	0	6	6	0	L	0.0001998001998002
SE_LT2_00207_p.Arg253Gln	SE_LT2_00207	outer membrane usher protein	0	0	0	1	1	1	1	1	1	0	0	0	0	0	0	6	6	0	NU	0.0001998001998002
SE_LT2_00207_p.Asn573Asp	SE_LT2_00207	outer membrane usher protein	0	0	0	1	1	1	1	1	1	0	0	0	0	0	0	6	6	0	NU	0.0001998001998002
SE_LT2_00210_p.Asp43Glu	SE_LT2_00210	fimbrial protein	0	0	0	1	1	1	1	1	1	0	0	0	0	0	0	6	6	0	NU	0.0001998001998002
SE_LT2_00212_p.Leu279Ile	SE_LT2_00212	putative protein YfcO	0	0	0	1	1	1	1	1	1	0	0	0	0	0	0	6	6	0	S	0.0001998001998002
SE_LT2_00213_p.Ala342Val	SE_LT2_00213	glutamate-1-semialdehyde-2,1-amii	0	0	0	1	1	1	1	1	1	0	0	0	0	0	0	6	6	0	H	0.0001998001998002
SE_LT2_00219_p.Ile84Val	SE_LT2_00219	dGTPase	0	0	0	1	1	1	1	1	1	0	0	0	0	0	0	6	6	0	F	0.0001998001998002
SE_LT2_00226_p.Asp768Glu	SE_LT2_00226	[protein-PII] uridylyltransferase	0	0	0	1	1	1	1	1	1	0	0	0	0	0	0	6	6	0	O	0.0001998001998002
SE_LT2_00227_p.Thr200Ala	SE_LT2_00227	type I methionyl aminopeptidase	0	0	0	1	1	1	1	1	1	0	0	0	0	0	0	6	6	0	E	0.0001998001998002
SE_LT2_00234_p.Val290Ala	SE_LT2_00234	1-deoxy-D-xylulose-5-phosphate re	0	0	0	1	1	1	1	1	1	0	0	0	0	0	0	6	6	0	I	0.0001998001998002
SE_LT2_00235_p.His160Asn	SE_LT2_00235	(2E,6E)-farnesyl- diphosphate-speci	0	0	0	1	1	1	1	1	1	0	0	0	0	0	0	6	6	0	F	0.0001998001998002
SE_LT2_00236_p.Asn214Asp	SE_LT2_00236	phosphatidate cytidylyltransferase	0	0	0	1	1	1	1	1	1	0	0	0	0	0	0	6	6	0	I	0.0001998001998002
SE_LT2_00250_p.Val343Ala	SE_LT2_00250	tRNA lysidine(34) synthetase TilS	0	0	0	1	1	1	1	1	1	0	0	0	0	0	0	6	6	0	J	0.0001998001998002
SE_LT2_00252_p.Arg11Gln	SE_LT2_00252	putative cytoplasmic protein	0	0	0	1	1	1	1	1	1	0	0	0	0	0	0	6	6	0	S	0.0001998001998002
SE_LT2_00270_p.Ala241Val	SE_LT2_00270	2,5-didehydrogluconate reductase E	0	0	0	1	1	1	1	1	1	0	0	0	0	0	0	6	6	0	H	0.0001998001998002
SE_LT2_00270_p.Asp146His	SE_LT2_00270	2,5-didehydrogluconate reductase E	0	0	0	1	1	1	1	1	1	0	0	0	0	0	0	6	6	0	H	0.0001998001998002
SE_LT2_00271_p.Ile122Met	SE_LT2_00271	putative LysR family transcriptional	0	0	0	1	1	1	1	1	1	0	0	0	0	0	0	6	6	0	K	0.0001998001998002
SE_LT2_00272_p.Ala275Val	SE_LT2_00272	MFS sugar transporter	0	0	0	1	1	1	1	1	1	0	0	0	0	0	0	6	6	0	U	0.0001998001998002
SE_LT2_00272_p.Met376Val	SE_LT2_00272	MFS sugar transporter	0	0	0	1	1	1	1	1	1	0	0	0	0	0	0	6	6	0	U	0.0001998001998002
SE_LT2_00275_p.Arg311Gln	SE_LT2_00275	murein transglycosylase D	0	0	0	1	1	1	1	1	1	0	0	0	0	0	0	6	6	0	M	0.0001998001998002
SE_LT2_00325_p.Lys23Gln	SE_LT2_00325	adhesin	0	0	0	1	1	1	1	1	1	0	0	0	0	0	0	6	6	0	M	0.0001998001998002
SE_LT2_00333_p.Glu250Lys	SE_LT2_00333	putative cytoplasmic protein	0	0	0	1	1	1	1	1	1	0	0	0	0	0	0	6	6	0	S	0.0001998001998002
SE_LT2_00342_p.Ala11Val	SE_LT2_00342	glutamate-5-semialdehyde dehydrc	0	0	0	1	1	1	1	1	1	0	0	0	0	0	0	6	6	0	E	0.0001998001998002
SE_LT2_00342_p.Ala330Thr	SE_LT2_00342	glutamate-5-semialdehyde dehydrc	0	0	0	1	1	1	1	1	1	0	0	0	0	0	0	6	6	0	E	0.0001998001998002
SE_LT2_00353_p.Ala133Thr	SE_LT2_00353	putative LysR family transcriptional	0	0	0	1	1	1	1	1	1	0	0	0	0	0	0	6	6	0	K	0.0001998001998002

SE_LT2_00355_p.Ala125Thr	SE_LT2_00355	hypothetical protein	0	0	0	1	1	1	1	1	1	0	0	0	0	0	0	6	6	0	-	0.0001998001998002
SE_LT2_00356_p.Asn246Ser	SE_LT2_00356	fimbrial assembly protein	0	0	0	1	1	1	1	1	1	0	0	0	0	0	0	6	6	0	M	0.0001998001998002
SE_LT2_00356_p.Val13Ala	SE_LT2_00356	fimbrial assembly protein	0	0	0	1	1	1	1	1	1	0	0	0	0	0	0	6	6	0	M	0.0001998001998002
SE_LT2_00357_p.Thr269Lys	SE_LT2_00357	fimbrial protein	0	0	0	1	1	1	1	1	1	0	0	0	0	0	0	6	6	0	NU	0.0001998001998002
SE_LT2_00358_p.Arg469His	SE_LT2_00358	fimbrial assembly protein	0	0	0	1	0	1	1	1	1	0	0	0	0	0	0	5	5	0	NU	0.00199800199800199
SE_LT2_00358_p.Gly215Arg	SE_LT2_00358	fimbrial assembly protein	0	0	0	1	1	1	1	1	1	0	0	0	0	0	0	6	6	0	NU	0.0001998001998002
SE_LT2_00359_p.Pro251Ser	SE_LT2_00359	fimbrial chaperone protein StbB	0	0	0	1	1	1	1	1	1	0	0	0	0	0	0	6	6	0	M	0.0001998001998002
SE_LT2_00364_p.His282Asp	SE_LT2_00364	putative diguanylate cyclase/phospl	0	0	0	1	1	1	1	1	1	0	0	0	0	0	0	6	6	0	T	0.0001998001998002
SE_LT2_00365_p.Asn168Thr	SE_LT2_00365	putative response regulator	0	0	0	1	1	1	1	1	1	0	0	0	0	0	0	6	6	0	K	0.0001998001998002
SE_LT2_00365_p.Thr113Met	SE_LT2_00365	putative response regulator	0	0	0	1	1	1	1	1	1	0	0	0	0	0	0	6	6	0	K	0.0001998001998002
SE_LT2_00370_p.Asn467Arg	SE_LT2_00370	homology to outer membrane efflu:	0	0	0	1	1	1	1	1	1	0	0	0	0	0	0	6	6	0	M	0.0001998001998002
SE_LT2_00371_p.Ala607Thr	SE_LT2_00371	multidrug efflux RND transporter pe	0	0	0	1	1	1	1	1	1	0	0	0	0	0	0	6	6	0	V	0.0001998001998002
SE_LT2_00371_p.Ala966Thr	SE_LT2_00371	multidrug efflux RND transporter pe	0	0	0	1	1	1	1	1	1	0	0	0	0	0	0	6	6	0	V	0.0001998001998002
SE_LT2_00371_p.Val517Phe	SE_LT2_00371	multidrug efflux RND transporter pe	0	0	0	1	1	1	1	1	1	0	0	0	0	0	0	6	6	0	V	0.0001998001998002
SE_LT2_00373_p.Ala59Glu	SE_LT2_00373	copper-translocating P-type ATPase	0	0	0	1	1	1	1	1	1	0	0	0	0	0	0	6	6	0	P	0.0001998001998002
SE_LT2_00376_p.Arg197Ser	SE_LT2_00376	MFS transporter	0	0	0	1	1	1	1	1	1	0	0	0	0	0	0	6	6	0	P	0.0001998001998002
SE_LT2_00377_p.Ala260Thr	SE_LT2_00377	restriction endonuclease	0	0	0	1	1	1	1	1	1	0	0	0	0	0	0	6	6	0	L	0.0001998001998002
SE_LT2_00378_p.Gly491Asp	SE_LT2_00378	DNA restriction (DNA helicase)	0	0	0	1	1	1	1	1	1	0	0	0	0	0	0	6	6	0	V	0.0001998001998002
SE_LT2_00381_p.Val165Ile	SE_LT2_00381	cytochrome BD2 subunit II	0	0	0	1	1	1	1	1	1	0	0	0	0	0	0	6	6	0	C	0.0001998001998002
SE_LT2_00387_p.Ser278Phe	SE_LT2_00387	propionate catabolism operon regul	0	0	0	1	1	1	1	1	1	0	0	0	0	0	0	6	6	0	KT	0.0001998001998002
SE_LT2_00401_p.Gly5Asp	SE_LT2_00401	hypothetical protein	0	0	0	1	1	1	1	1	1	0	0	0	0	0	0	6	6	0	S	0.0001998001998002
SE_LT2_00415_p.Val875Ala	SE_LT2_00415	exonuclease subunit SbcC	0	0	0	1	1	1	1	1	1	0	0	0	0	0	0	6	6	0	L	0.0001998001998002
SE_LT2_00418_p.Glu406Gln	SE_LT2_00418	two-component system sensor histi	0	0	0	1	1	1	1	1	1	0	0	0	0	0	0	6	6	0	T	0.0001998001998002
SE_LT2_00421_p.Asn509Ser	SE_LT2_00421	maltodextrin glucosidase	0	0	0	1	1	1	1	1	1	0	0	0	0	0	0	6	6	0	G	0.0001998001998002
SE_LT2_00424_p.Ala245Val	SE_LT2_00424	S-adenosylmethionine-tRNA ribosyl	0	0	0	1	1	1	1	1	1	0	0	0	0	0	0	6	6	0	F	0.0001998001998002
SE_LT2_00424_p.Ser351Phe	SE_LT2_00424	S-adenosylmethionine-tRNA ribosyl	0	0	0	1	1	1	1	1	1	0	0	0	0	0	0	6	6	0	F	0.0001998001998002
SE_LT2_00427_p.Ser195Gly	SE_LT2_00427	protein-export membrane protein S	0	0	0	1	1	1	1	1	1	0	0	0	0	0	0	6	6	0	U	0.0001998001998002
SE_LT2_00451_p.Gln195His	SE_LT2_00451	phosphonate utilization transcriptio	0	0	0	1	1	1	1	1	1	0	0	0	0	0	0	6	6	0	K	0.0001998001998002
SE_LT2_00451_p.Val176Ile	SE_LT2_00451	phosphonate utilization transcriptio	0	0	0	1	1	1	1	1	1	0	0	0	0	0	0	6	6	0	K	0.0001998001998002
SE_LT2_00452_p.Glu295Lys	SE_LT2_00452	2-aminoethylphosphonate--pyruvat	0	0	0	1	1	1	1	1	1	0	0	0	0	0	0	6	6	0	E	0.0001998001998002
SE_LT2_00457_p.Leu322Met	SE_LT2_00457	putative MFS family transporter	0	0	0	1	1	1	1	1	1	0	0	0	0	0	0	6	6	0	EGP	0.0001998001998002
SE_LT2_00458_p.Met445Ile	SE_LT2_00458	putative TPR repeat protein	0	0	0	1	1	1	1	1	1	0	0	0	0	0	0	6	6	0	S	0.0001998001998002
SE_LT2_00458_p.Trp226Cys	SE_LT2_00458	putative TPR repeat protein	0	0	0	1	1	1	1	1	1	0	0	0	0	0	0	6	6	0	S	0.0001998001998002
SE_LT2_00475_p.Gln106Leu	SE_LT2_00475	putative protein	0	0	0	1	1	1	1	1	1	0	0	0	0	0	0	6	6	0	L	0.0001998001998002
SE_LT2_00478_p.Ala513Val	SE_LT2_00478	peptide ABC transporter substrate-t	0	0	0	1	1	1	1	1	1	0	0	0	0	0	0	6	6	0	S	0.0001998001998002
SE_LT2_00480_p.Ala345Glu	SE_LT2_00480	putative cysteine synthase/cystathi	0	0	0	1	1	1	1	1	1	0	0	0	0	0	0	6	6	0	E	0.0001998001998002
SE_LT2_00501_p.Val18Met	SE_LT2_00501	hypothetical protein	0	0	0	1	1	1	1	1	1	0	0	0	0	0	0	6	6	0	S	0.0001998001998002
SE_LT2_00511_p.Pro226Leu	SE_LT2_00511	ferrochelatase	0	0	0	1	1	1	1	1	1	0	0	0	0	0	0	6	6	0	H	0.0001998001998002
SE_LT2_00514_p.Asp480Glu	SE_LT2_00514	Kef family K(+) transporter	0	0	0	1	1	1	1	1	1	0	0	0	0	0	0	6	6	0	P	0.0001998001998002
SE_LT2_00521_p.Thr7Pro	SE_LT2_00521	hypothetical protein	0	0	0	1	1	1	1	1	1	0	0	0	0	0	0	6	6	0	S	0.0001998001998002
SE_LT2_00527_p.Ala171Thr	SE_LT2_00527	iron export ABC transporter permea	0	0	0	1	1	1	1	1	1	0	0	0	0	0	0	6	6	0	S	0.0001998001998002
SE_LT2_00529_p.Glu66Asp	SE_LT2_00529	putative oxidoreductase	0	0	0	1	1	1	1	1	1	0	0	0	0	0	0	6	6	0	S	0.0001998001998002
SE_LT2_00535_p.Ser122Arg	SE_LT2_00535	metal ABC transporter substrate-bir	0	0	0	1	1	1	1	1	1	0	0	0	0	0	0	6	6	0	P	0.0001998001998002

SE_LT2_00538_p.Arg307Ser	SE_LT2_00538	tRNA 2-selenouridine(34) synthase	0	0	0	1	1	1	1	1	1	0	0	0	0	0	0	6	6	0	H	0.0001998001998002
SE_LT2_00544_p.Arg102Leu	SE_LT2_00544	2-hydroxy-3-oxopropionate reducta	0	0	0	1	1	1	1	1	1	0	0	0	0	0	0	6	6	0	I	0.0001998001998002
SE_LT2_00549_p.Thr159Ala	SE_LT2_00549	glycerate kinase	0	0	0	0	1	1	1	1	1	0	0	0	0	0	0	5	5	0	G	0.00199800199800199
SE_LT2_00554_p.Thr242Met	SE_LT2_00554	acyl-CoA synthetase FdrA	0	0	0	1	1	1	1	1	1	0	0	0	0	0	0	6	6	0	C	0.0001998001998002
SE_LT2_00554_p.Thr495Pro	SE_LT2_00554	acyl-CoA synthetase FdrA	0	0	0	1	1	1	1	1	1	0	0	0	0	0	0	6	6	0	C	0.0001998001998002
SE_LT2_00556_p.Val25Leu	SE_LT2_00556	carboxylase	0	0	0	1	1	1	1	1	1	0	0	0	0	0	0	6	6	0	S	0.0001998001998002
SE_LT2_00557_p.Ala135Ser	SE_LT2_00557	putative carbamate kinase	0	0	0	1	1	1	1	1	1	0	0	0	0	0	0	6	6	0	E	0.0001998001998002
SE_LT2_00563_p.Ser33Asn	SE_LT2_00563	hypothetical protein	0	0	0	1	1	1	1	1	1	0	0	0	0	0	0	6	6	0	S	0.0001998001998002
SE_LT2_00564_p.Ile11Val	SE_LT2_00564	hypothetical protein	0	0	0	1	1	1	1	1	1	0	0	0	0	0	0	6	6	0	-	0.0001998001998002
SE_LT2_00566_p.Val68Ile	SE_LT2_00566	ribosome-associated protein	0	0	0	1	1	1	1	1	1	0	0	0	0	0	0	6	6	0	S	0.0001998001998002
SE_LT2_00569_p.Thr58Ser	SE_LT2_00569	fimbrial protein FimI	0	0	0	1	1	1	1	1	1	0	0	0	0	0	0	6	6	0	NU	0.0001998001998002
SE_LT2_00571_p.Asp440Asn	SE_LT2_00571	outer membrane usher protein	0	0	0	1	1	1	1	1	1	0	0	0	0	0	0	6	6	0	NU	0.0001998001998002
SE_LT2_00572_p.Ala71Glu	SE_LT2_00572	fimbrial adhesin FimH	0	0	0	1	1	1	1	1	1	0	0	0	0	0	0	6	6	0	NU	0.0001998001998002
SE_LT2_00572_p.Thr74Met	SE_LT2_00572	fimbrial adhesin FimH	0	0	0	1	1	1	1	1	1	0	0	0	0	0	0	6	6	0	NU	0.0001998001998002
SE_LT2_00575_p.Arg153Gln	SE_LT2_00575	fimbriae Y protein	0	0	0	1	1	1	1	1	1	0	0	0	0	0	0	6	6	0	KT	0.0001998001998002
SE_LT2_00575_p.Thr75Ala	SE_LT2_00575	fimbriae Y protein	0	0	0	1	1	1	1	1	1	0	0	0	0	0	0	6	6	0	KT	0.0001998001998002
SE_LT2_00575_p.Tyr43Asn	SE_LT2_00575	fimbriae Y protein	0	0	0	1	1	1	1	1	1	0	0	0	0	0	0	6	6	0	KT	0.0001998001998002
SE_LT2_00583_p.His82Tyr	SE_LT2_00583	putative glycosyltransferase	0	0	0	1	1	1	1	1	1	0	0	0	0	0	0	6	6	0	M	0.0001998001998002
SE_LT2_00583_p.Ile3Val	SE_LT2_00583	putative glycosyltransferase	0	0	0	1	1	1	1	1	1	0	0	0	0	0	0	6	6	0	M	0.0001998001998002
SE_LT2_00584_p.Ile105Thr	SE_LT2_00584	translocase	0	0	0	1	1	1	1	1	1	0	0	0	0	0	0	6	6	0	U	0.0001998001998002
SE_LT2_00587_p.Pro337Ser	SE_LT2_00587	pyridine nucleotide-disulfide oxido	0	0	0	1	1	1	1	1	1	0	0	0	0	0	0	6	6	0	C	0.0001998001998002
SE_LT2_00590_p.Arg328Leu	SE_LT2_00590	DNA repair protein	0	0	0	1	1	1	1	1	1	0	0	0	0	0	0	6	6	0	O	0.0001998001998002
SE_LT2_00592_p.Ser53Ile	SE_LT2_00592	miniconductance mechanosensitive	0	0	0	1	1	1	1	1	1	0	0	0	0	0	0	6	6	0	M	0.0001998001998002
SE_LT2_00593_p.Ala617Ser	SE_LT2_00593	autotransporter outer membrane be	0	0	0	1	0	1	1	1	1	0	0	0	0	0	0	5	5	0	IN	0.00199800199800199
SE_LT2_00593_p.Val359Ile	SE_LT2_00593	autotransporter outer membrane be	0	0	0	1	1	1	1	1	1	0	0	0	0	0	0	6	6	0	IN	0.0001998001998002
SE_LT2_00601_p.Arg10His	SE_LT2_00601	NAD(P)H nitroreductase	0	0	0	1	1	1	1	1	1	0	0	0	0	0	0	6	6	0	C	0.0001998001998002
SE_LT2_00609_p.Val585Ala	SE_LT2_00609	outer membrane receptor protein	0	0	0	1	1	1	1	1	1	0	0	0	0	0	0	6	6	0	M	0.0001998001998002
SE_LT2_00610_p.Asp153Gly	SE_LT2_00610	enterochelin esterase	0	0	0	1	1	1	1	1	1	0	0	0	0	0	0	6	6	0	P	0.0001998001998002
SE_LT2_00612_p.Ala709Val	SE_LT2_00612	non-ribosomal peptide synthetase	0	0	0	1	0	1	1	1	1	0	0	0	0	0	0	5	5	0	Q	0.00199800199800199
SE_LT2_00612_p.Ile84Leu	SE_LT2_00612	non-ribosomal peptide synthetase	0	0	0	1	1	1	1	1	1	0	0	0	0	0	0	6	6	0	Q	0.0001998001998002
SE_LT2_00616_p.Leu57Met	SE_LT2_00616	iron-enterobactin transporter	0	0	0	1	1	1	1	1	1	0	0	0	0	0	0	6	6	0	P	0.0001998001998002
SE_LT2_00617_p.Ala112Thr	SE_LT2_00617	MFS transporter	0	0	0	1	1	1	1	1	1	0	0	0	0	0	0	6	6	0	EGP	0.0001998001998002
SE_LT2_00617_p.Ala64Thr	SE_LT2_00617	MFS transporter	0	0	0	1	1	1	1	1	1	0	0	0	0	0	0	6	6	0	EGP	0.0001998001998002
SE_LT2_00620_p.Leu375Val	SE_LT2_00620	2,3-dihydroxybenzoate-AMP ligase	0	0	0	1	1	1	1	1	1	0	0	0	0	0	0	6	6	0	Q	0.0001998001998002
SE_LT2_00622_p.Asp181Asn	SE_LT2_00622	2,3-dihydro-2,3-dihydroxybenzoate	0	0	0	1	1	1	1	1	1	0	0	0	0	0	0	6	6	0	IQ	0.0001998001998002
SE_LT2_00624_p.Ala619Ser	SE_LT2_00624	carbon starvation protein A	0	0	0	1	1	1	1	1	1	0	0	0	0	0	0	6	6	0	T	0.0001998001998002
SE_LT2_00631_p.Ala15Ser	SE_LT2_00631	thiol:disulfide interchange protein	0	0	0	1	1	1	1	1	1	0	0	0	0	0	0	6	6	0	O	0.0001998001998002
SE_LT2_00636_p.Asn305Ser	SE_LT2_00636	DMSO reductase	0	0	0	1	1	1	1	1	1	0	0	0	0	0	0	6	6	0	C	0.0001998001998002
SE_LT2_00644_p.Arg458Lys	SE_LT2_00644	anion permease	0	0	0	1	1	1	1	1	1	0	0	0	0	0	0	6	6	0	P	0.0001998001998002
SE_LT2_00651_p.Ala389Thr	SE_LT2_00651	histidine kinase	0	0	0	1	1	1	1	1	1	0	0	0	0	0	0	6	6	0	T	0.0001998001998002
SE_LT2_00651_p.Cys478Ser	SE_LT2_00651	histidine kinase	0	0	0	1	1	1	1	1	1	0	0	0	0	0	0	6	6	0	T	0.0001998001998002
SE_LT2_00658_p.Ile83Val	SE_LT2_00658	putative hydrolase	0	0	0	1	1	1	1	1	1	0	0	0	0	0	0	6	6	0	S	0.0001998001998002
SE_LT2_00658_p.Val130Ala	SE_LT2_00658	putative hydrolase	0	0	0	1	1	1	0	1	1	0	0	0	0	0	0	5	5	0	S	0.00199800199800199
SE_LT2_00670_p.ValPro71ThrLeu	SE_LT2_00670	alpha-ribazole phosphatase	0	0	0	1	1	1	1	1	1	0	0	0	0	0	0	6	6	0	G	0.0001998001998002
SE_LT2_00677_p.Gln64Arg	SE_LT2_00677	hypothetical protein	0	0	0	1	1	1	1	1	1	0	0	0	0	0	0	6	6	0	G	0.0001998001998002
SE_LT2_00694_p.Ala236Thr	SE_LT2_00694	apolipoprotein N-acyltransferase	0	0	0	1	1	1	0	1	1	0	0	0	0	0	0	5	5	0	M	0.00199800199800199
SE_LT2_00708_p.Asp128Asn	SE_LT2_00708	asparagine synthetase B	0	0	0	1	1	1	1	1	1	0	0	0	0	0	0	6	6	0	E	0.0001998001998002
SE_LT2_00711_p.Arg10Trp	SE_LT2_00711	N-acetylglucosamine-6-phosphate	0	0	0	1	1	1	1	1	1	0	0	0	0	0	0	6	6	0	G	0.0001998001998002
SE_LT2_00725_p.Ala58Val	SE_LT2_00725	replication initiation regulator SeqA	0	0	0	1	1	1	1	1	1	0	0	0	0	0	0	6	6	0	L	0.0001998001998002
SE_LT2_00731_p.Ala72Val	SE_LT2_00731	two-component system response re	0	0	0	1	1	1	1	1	1	0	0	0	0	0	0	6	6	0	K	0.0001998001998002

SE_LT2_00732_p.Asp321Ala	SE_LT2_00732	two-component system sensor histi	0	0	0	1	0	1	1	1	1	0	0	0	0	0	0	5	5	0	T	0.00199800199800199
SE_LT2_00735_p.Ala301Thr	SE_LT2_00735	potassium-transporting ATPase subu	0	0	0	1	0	1	1	1	1	0	0	0	0	0	0	5	5	0	P	0.00199800199800199
SE_LT2_00737_p.Leu32Arg	SE_LT2_00737	deoxyribodipyrimidine photo-lyase	0	0	0	1	1	1	1	1	1	0	0	0	0	0	0	6	6	0	H	0.0001998001998002
SE_LT2_00738_p.Ala357Thr	SE_LT2_00738	MFS transporter	0	0	0	1	1	1	1	1	1	0	0	0	0	0	0	6	6	0	U	0.0001998001998002
SE_LT2_00739_p.Lys79Arg	SE_LT2_00739	GTP cyclohydrolase 1 type 2	0	0	0	1	1	1	1	1	1	0	0	0	0	0	0	6	6	0	S	0.0001998001998002
SE_LT2_00778_p.Ser204Leu	SE_LT2_00778	cell division protein CpoB	0	0	0	1	1	1	1	1	1	0	0	0	0	0	0	6	6	0	D	0.0001998001998002
SE_LT2_00795_p.Val98Ala	SE_LT2_00795	fumarate hydratase	0	0	0	1	1	1	1	1	1	0	0	0	0	0	0	6	6	0	C	0.0001998001998002
SE_LT2_00798_p.Phe246Leu	SE_LT2_00798	putative cation transporter	0	0	0	1	0	1	1	1	1	0	0	0	0	0	0	5	5	0	P	0.00199800199800199
SE_LT2_00804_p.Ala35Val	SE_LT2_00804	iron ABC transporter permease	0	0	0	1	1	1	1	1	1	0	0	0	0	0	0	6	6	0	U	0.0001998001998002
SE_LT2_00804_p.Glu68Gly	SE_LT2_00804	iron ABC transporter permease	0	0	0	1	1	1	1	1	1	0	0	0	0	0	0	6	6	0	U	0.0001998001998002
SE_LT2_00804_p.Pro114Ser	SE_LT2_00804	iron ABC transporter permease	0	0	0	1	1	1	1	1	1	0	0	0	0	0	0	6	6	0	U	0.0001998001998002
SE_LT2_00809_p.Asp88Asn	SE_LT2_00809	galactose-1-phosphate uridylyltrans	0	0	0	1	1	1	1	1	1	0	0	0	0	0	0	6	6	0	H	0.0001998001998002
SE_LT2_00810_p.Ser9Asn	SE_LT2_00810	UDP-glucose 4-epimerase	0	0	0	1	1	1	1	1	1	0	0	0	0	0	0	6	6	0	M	0.0001998001998002
SE_LT2_00811_p.Ile178Val	SE_LT2_00811	hypothetical protein	0	0	0	1	1	1	1	1	1	0	0	0	0	0	0	6	6	0	S	0.0001998001998002
SE_LT2_00812_p.Met186Ile	SE_LT2_00812	molybdate ABC transporter ATP-bin	0	0	0	1	1	1	1	1	1	0	0	0	0	0	0	6	6	0	P	0.0001998001998002
SE_LT2_00813_p.Gly244Asp	SE_LT2_00813	molybdenum-dependent transcripti	0	0	0	1	1	1	1	1	1	0	0	0	0	0	0	6	6	0	H	0.0001998001998002
SE_LT2_00818_p.Val175Ile	SE_LT2_00818	pyridoxal phosphatase	0	0	0	1	1	1	1	1	1	0	0	0	0	0	0	6	6	0	S	0.0001998001998002
SE_LT2_00822_p.Arg165Gln	SE_LT2_00822	formimidoylglutamase	0	0	0	1	1	1	1	1	1	0	0	0	0	0	0	6	6	0	F	0.0001998001998002
SE_LT2_00822_p.Arg25Gln	SE_LT2_00822	formimidoylglutamase	0	0	0	1	1	1	1	1	1	0	0	0	0	0	0	6	6	0	F	0.0001998001998002
SE_LT2_00822_p.Asp180Ala	SE_LT2_00822	formimidoylglutamase	0	0	0	1	1	1	1	1	1	0	0	0	0	0	0	6	6	0	F	0.0001998001998002
SE_LT2_00822_p.Ile19Leu	SE_LT2_00822	formimidoylglutamase	0	0	0	1	1	1	1	1	1	0	0	0	0	0	0	6	6	0	F	0.0001998001998002
SE_LT2_00822_p.Ser38Ala	SE_LT2_00822	formimidoylglutamase	0	0	0	1	1	1	1	1	1	0	0	0	0	0	0	6	6	0	F	0.0001998001998002
SE_LT2_00826_p.Ala200Val	SE_LT2_00826	histidine ammonia-lyase	0	0	0	1	1	1	1	1	1	0	0	0	0	0	0	6	6	0	E	0.0001998001998002
SE_LT2_00826_p.GluGln471GlnA	SE_LT2_00826	histidine ammonia-lyase	0	0	0	1	1	1	1	1	1	0	0	0	0	0	0	6	6	0	E	0.0001998001998002
SE_LT2_00826_p.Val253Ala	SE_LT2_00826	histidine ammonia-lyase	0	0	0	1	1	1	1	1	1	0	0	0	0	0	0	6	6	0	E	0.0001998001998002
SE_LT2_00827_p.Ala155Val	SE_LT2_00827	kinase inhibitor	0	0	0	1	1	1	1	1	1	0	0	0	0	0	0	6	6	0	S	0.0001998001998002
SE_LT2_00831_p.Arg233Trp	SE_LT2_00831	malonyl-ACP O-methyltransferase B	0	0	0	1	1	1	1	1	1	0	0	0	0	0	0	6	6	0	H	0.0001998001998002
SE_LT2_00847_p.Ala280Thr	SE_LT2_00847	Inner membrane protein YbhN	0	0	0	0	1	1	1	1	1	0	0	0	0	0	0	5	5	0	S	0.00199800199800199
SE_LT2_00848_p.Asp334Asn	SE_LT2_00848	cardiolipin synthase B	0	0	0	1	1	1	1	1	1	0	0	0	0	0	0	6	6	0	I	0.0001998001998002
SE_LT2_00860_p.Arg79His	SE_LT2_00860	putative protein YbiI	0	0	0	1	1	1	1	1	1	0	0	0	0	0	0	6	6	0	T	0.0001998001998002
SE_LT2_00860_p.Gly4Cys	SE_LT2_00860	putative protein YbiI	0	0	0	1	1	1	1	1	1	0	0	0	0	0	0	6	6	0	T	0.0001998001998002
SE_LT2_00867_p.Arg65His	SE_LT2_00867	threonine/homoserine exporter Rht	0	0	0	1	1	1	1	1	1	0	0	0	0	0	0	6	6	0	S	0.0001998001998002
SE_LT2_00869_p.Ala500Thr	SE_LT2_00869	phosphoethanolamine transferase	0	0	0	1	1	1	1	1	1	0	0	0	0	0	0	6	6	0	S	0.0001998001998002
SE_LT2_00874_p.Ala371Thr	SE_LT2_00874	ABC-F family ATPase	0	0	0	1	1	1	1	1	1	0	0	0	0	0	0	6	6	0	S	0.0001998001998002
SE_LT2_00877_p.Ala381Thr	SE_LT2_00877	putative protein YbiU	0	0	0	1	1	1	1	1	1	0	0	0	0	0	0	6	6	0	S	0.0001998001998002
SE_LT2_00880_p.Ala209Ser	SE_LT2_00880	pyruvate formate lyase-activating pr	0	0	0	1	1	1	1	1	1	0	0	0	0	0	0	6	6	0	H	0.0001998001998002
SE_LT2_00880_p.Gln192Arg	SE_LT2_00880	pyruvate formate lyase-activating pr	0	0	0	1	1	1	1	1	1	0	0	0	0	0	0	6	6	0	H	0.0001998001998002
SE_LT2_00882_p.Ala170Ser	SE_LT2_00882	molybdopterin biosynthesis protein	0	0	0	1	1	1	1	1	1	0	0	0	0	0	0	6	6	0	H	0.0001998001998002
SE_LT2_00885_p.Ala21Val	SE_LT2_00885	glutathione ABC transporter substra	0	0	0	1	1	1	1	1	1	0	0	0	0	0	0	6	6	0	E	0.0001998001998002
SE_LT2_00887_p.Val18Ile	SE_LT2_00887	glutathione ABC transporter permea	0	0	0	1	1	1	1	1	1	0	0	0	0	0	0	6	6	0	EP	0.0001998001998002
SE_LT2_00906_p.Val96Ile	SE_LT2_00906	Inner membrane protein YbjJ	0	0	0	1	1	1	1	1	1	0	0	0	0	0	0	6	6	0	G	0.0001998001998002
SE_LT2_00917_p.Val1?	SE_LT2_00917	ABC transporter ATP-binding protein	0	0	0	1	1	1	1	1	1	0	0	0	0	0	0	6	6	0	E	0.0001998001998002
SE_LT2_00919_p.Ala180Pro	SE_LT2_00919	putrescine ABC transporter permeas	0	0	0	1	1	1	1	1	1	0	0	0	0	0	0	6	6	0	P	0.0001998001998002
SE_LT2_00919_p.Gly133Asp	SE_LT2_00919	putrescine ABC transporter permeas	0	0	0	1	0	1	1	1	1	0	0	0	0	0	0	5	5	0	P	0.00199800199800199
SE_LT2_00919_p.Val256Ile	SE_LT2_00919	putrescine ABC transporter permeas	0	0	0	1	1	1	1	1	1	0	0	0	0	0	0	6	6	0	P	0.0001998001998002
SE_LT2_00921_p.Gly49Ser	SE_LT2_00921	23S rRNA (uracil(747)-C(5))-methyltr	0	0	0	1	1	0	1	1	1	0	0	0	0	0	0	5	5	0	J	0.00199800199800199
SE_LT2_00924_p.Val495fs	SE_LT2_00924	putative sulfatase	0	0	0	1	1	1	1	1	1	0	0	0	0	0	0	6	6	0	P	0.0001998001998002
SE_LT2_00928_p.Ala189Val	SE_LT2_00928	arginine ABC transporter substrate-b	0	0	0	1	1	1	1	1	1	0	0	0	0	0	0	6	6	0	ET	0.0001998001998002
SE_LT2_00993_p.Ala208Thr	SE_LT2_00993	N-acetylmuramoyl-L-alanine amidas	0	0	0	1	1	1	1	1	1	0	0	0	0	0	0	6	6	0	V	0.0001998001998002
SE_LT2_00998_p.Arg18His	SE_LT2_00998	NADH oxidoreductase for hcp gene	0	0	0	1	1	1	1	1	1	0	0	0	0	0	0	6	6	0	C	0.0001998001998002

SE_LT2_01006_p.Arg19Cys	SE_LT2_01006	ATP-dependent Clp protease adapte	0	0	0	1	1	1	1	1	1	0	0	0	0	0	0	6	6	0	E	0.0001998001998002
SE_LT2_01013_p.Asp186Asn	SE_LT2_01013	hydrolase	0	0	0	1	1	1	1	1	1	0	0	0	0	0	0	6	6	0	Q	0.0001998001998002
SE_LT2_01018_p.Ala329Thr	SE_LT2_01018	cysteine/glutathione ABC transporte	0	0	0	1	1	1	1	1	1	0	0	0	0	0	0	6	6	0	CO	0.0001998001998002
SE_LT2_01018_p.ThrPro331SerLe	SE_LT2_01018	cysteine/glutathione ABC transporte	0	0	0	1	1	1	1	1	1	0	0	0	0	0	0	6	6	0	CO	0.0001998001998002
SE_LT2_01022_p.Ile371Met	SE_LT2_01022	DNA translocase FtsK	0	0	0	1	0	1	1	1	1	0	0	0	0	0	0	5	5	0	D	0.00199800199800199
SE_LT2_01045_p.Ala155Glu	SE_LT2_01045	ComEC family protein	0	0	0	1	1	1	1	1	1	0	0	0	0	0	0	6	6	0	S	0.0001998001998002
SE_LT2_01048_p.Glu198Gly	SE_LT2_01048	hypothetical protein	0	0	0	1	1	1	1	1	1	0	0	0	0	0	0	6	6	0	S	0.0001998001998002
SE_LT2_01054_p.Ala420Val	SE_LT2_01054	condensin subunit MukF	0	0	0	1	1	1	1	1	1	0	0	0	0	0	0	6	6	0	D	0.0001998001998002
SE_LT2_01056_p.Glu500Gln	SE_LT2_01056	cell division protein MukB	0	0	0	1	1	1	1	1	1	0	0	0	0	0	0	6	6	0	D	0.0001998001998002
SE_LT2_01057_p.Asp142Asn	SE_LT2_01057	L,D-transpeptidase	0	0	0	1	0	1	1	1	1	0	0	0	0	0	0	5	5	0	S	0.00199800199800199
SE_LT2_01060_p.Asp356Ala	SE_LT2_01060	aromatic amino acid aminotransfera	0	0	0	1	1	1	1	1	1	0	0	0	0	0	0	6	6	0	E	0.0001998001998002
SE_LT2_01066_p.Glu460Val	SE_LT2_01066	amino acid:proton symporter	0	0	0	1	1	1	1	1	1	0	0	0	0	0	0	6	6	0	U	0.0001998001998002
SE_LT2_01127_p.Ala486Thr	SE_LT2_01127	aminopeptidase N	0	0	0	1	1	1	1	0	1	0	0	0	0	0	0	5	5	0	E	0.00199800199800199
SE_LT2_01127_p.Phe519Val	SE_LT2_01127	aminopeptidase N	0	0	0	1	1	1	1	1	1	0	0	0	0	0	0	6	6	0	E	0.0001998001998002
SE_LT2_01131_p.Ile255Leu	SE_LT2_01131	putative protein YcbX	0	0	0	1	1	1	1	1	1	0	0	0	0	0	0	6	6	0	C	0.0001998001998002
SE_LT2_01132_p.Phe363Cys	SE_LT2_01132	23S rRNA (guanine(2445)-N(2))/(gua	0	0	0	1	1	1	1	1	1	0	0	0	0	0	0	6	6	0	J	0.0001998001998002
SE_LT2_01138_p.Ala310Glu	SE_LT2_01138	Lon protease	0	0	0	1	1	1	1	1	1	0	0	0	0	0	0	6	6	0	E	0.0001998001998002
SE_LT2_01138_p.Ser300Ala	SE_LT2_01138	Lon protease	0	0	0	1	1	1	1	0	1	0	0	0	0	0	0	5	5	0	E	0.00199800199800199
SE_LT2_01143_p.Thr130Ala	SE_LT2_01143	TIGR01666 family membrane protein	0	0	0	1	1	1	1	1	1	0	0	0	0	0	0	6	6	0	S	0.0001998001998002
SE_LT2_01145_p.Gln172Arg	SE_LT2_01145	DNA helicase IV	0	0	0	1	1	1	1	1	1	0	0	0	0	0	0	6	6	0	L	0.0001998001998002
SE_LT2_01145_p.Val293Ile	SE_LT2_01145	DNA helicase IV	0	0	0	0	1	1	1	1	1	0	0	0	0	0	0	5	5	0	L	0.00199800199800199
SE_LT2_01157_p.Thr81Ile	SE_LT2_01157	virulence protein	0	0	0	1	1	1	1	1	1	0	0	0	0	0	0	6	6	0	E	0.0001998001998002
SE_LT2_01158_p.Ala199Thr	SE_LT2_01158	effector protein PipB	0	0	0	1	1	1	1	1	1	0	0	0	0	0	0	6	6	0	S	0.0001998001998002
SE_LT2_01158_p.Asp151Gly	SE_LT2_01158	effector protein PipB	0	0	0	1	1	1	1	1	1	0	0	0	0	0	0	6	6	0	S	0.0001998001998002
SE_LT2_01158_p.Asp165Asn	SE_LT2_01158	effector protein PipB	0	0	0	1	1	1	1	1	1	0	0	0	0	0	0	6	6	0	S	0.0001998001998002
SE_LT2_01158_p.His163Asn	SE_LT2_01158	effector protein PipB	0	0	0	1	1	1	1	1	1	0	0	0	0	0	0	6	6	0	S	0.0001998001998002
SE_LT2_01158_p.Lys23Glu	SE_LT2_01158	effector protein PipB	0	0	0	1	1	1	1	1	1	0	0	0	0	0	0	6	6	0	S	0.0001998001998002
SE_LT2_01163_p.Ser325Gly	SE_LT2_01163	dipeptidase	0	0	0	1	1	1	1	1	1	0	0	0	0	0	0	6	6	0	M	0.0001998001998002
SE_LT2_01168_p.Ala119Ser	SE_LT2_01168	4-hydroxyphenylacetate 3-monooxy	0	0	0	0	1	1	1	1	1	0	0	0	0	0	0	5	5	0	Q	0.00199800199800199
SE_LT2_01175_p.Ala8Thr	SE_LT2_01175	2,4-dihydroxyhept-2-ene-1,7-dioic a	0	0	0	1	1	1	1	1	1	0	0	0	0	0	0	6	6	0	G	0.0001998001998002
SE_LT2_01184_p.Gln139His	SE_LT2_01184	disulfide bond formation protein Ds	0	0	0	1	1	1	1	1	1	0	0	0	0	0	0	6	6	0	O	0.0001998001998002
SE_LT2_01192_p.Asp419Glu	SE_LT2_01192	trifunctional transcriptional regulato	0	0	0	1	1	1	1	1	1	0	0	0	0	0	0	6	6	0	CEK	0.0001998001998002
SE_LT2_01192_p.Gln130Arg	SE_LT2_01192	trifunctional transcriptional regulato	0	0	0	0	1	1	1	1	1	0	0	0	0	0	0	5	5	0	CEK	0.00199800199800199
SE_LT2_01192_p.Leu204Gln	SE_LT2_01192	trifunctional transcriptional regulato	0	0	0	0	1	1	1	1	1	0	0	0	0	0	0	5	5	0	CEK	0.00199800199800199
SE_LT2_01215_p.Gln115Lys	SE_LT2_01215	curli assembly protein CsgE	0	0	0	1	1	1	1	1	1	0	0	0	0	0	0	6	6	0	S	0.0001998001998002
SE_LT2_01224_p.Leu391Met	SE_LT2_01224	periplasmic glucans biosynthesis pro	0	0	0	1	1	1	1	1	1	0	0	0	0	0	0	6	6	0	P	0.0001998001998002
SE_LT2_01225_p.AlaArg846ValGl	SE_LT2_01225	glucan biosynthesis glucosyltransfer	0	0	0	1	1	1	1	1	1	0	0	0	0	0	0	6	6	0	M	0.0001998001998002
SE_LT2_01229_p.Ter307Ter	SE_LT2_01229	lipid A biosynthesis lauroyl acyltrans	0	0	0	1	1	1	1	1	1	0	0	0	0	0	0	6	6	0	M	0.0001998001998002
SE_LT2_01232_p.Ile68Val	SE_LT2_01232	cytochrome B	0	0	0	1	1	1	1	1	1	0	0	0	0	0	0	6	6	0	C	0.0001998001998002
SE_LT2_01240_p.Ser56Asn	SE_LT2_01240	glutaredoxin, GrxB family	0	0	0	1	1	1	1	1	1	0	0	0	0	0	0	6	6	0	O	0.0001998001998002
SE_LT2_01244_p.Thr171Ile	SE_LT2_01244	virulence factor MviM	0	0	0	1	1	1	1	1	1	0	0	0	0	0	0	6	6	0	S	0.0001998001998002
SE_LT2_01248_p.Arg128Lys	SE_LT2_01248	flagella basal body P-ring formation	0	0	0	1	1	1	1	1	1	0	0	0	0	0	0	6	6	0	N	0.0001998001998002
SE_LT2_01248_p.Thr94Ile	SE_LT2_01248	flagella basal body P-ring formation	0	0	0	1	1	1	1	1	1	0	0	0	0	0	0	6	6	0	N	0.0001998001998002
SE_LT2_01252_p.Ala256Lys	SE_LT2_01252	flagellar hook protein FlgE	0	0	0	1	1	1	1	1	1	0	0	0	0	0	0	6	6	0	N	0.0001998001998002
SE_LT2_01252_p.Asn245Asp	SE_LT2_01252	flagellar hook protein FlgE	0	0	0	1	1	1	1	1	1	0	0	0	0	0	0	6	6	0	N	0.0001998001998002
SE_LT2_01252_p.Thr247Glu	SE_LT2_01252	flagellar hook protein FlgE	0	0	0	1	1	1	1	1	1	0	0	0	0	0	0	6	6	0	N	0.0001998001998002
SE_LT2_01262_p.Thr318Met	SE_LT2_01262	23S rRNA pseudouridine(955/2504/2	0	0	0	1	1	1	1	1	1	0	0	0	0	0	0	6	6	0	J	0.0001998001998002
SE_LT2_01263_p.Ile49Val	SE_LT2_01263	putative inner membrane lipoprote	0	0	0	1	1	1	1	1	1	0	0	0	0	0	0	6	6	0	-	0.0001998001998002
SE_LT2_01263_p.Lys159Arg	SE_LT2_01263	putative inner membrane lipoprote	0	0	0	1	1	1	1	1	1	0	0	0	0	0	0	6	6	0	-	0.0001998001998002
SE_LT2_01269_p.Leu108Phe	SE_LT2_01269	phosphate acyltransferase	0	0	0	1	1	1	1	1	1	0	0	0	0	0	0	6	6	0	I	0.0001998001998002

SE_LT2_01276_p.Ala299Val	SE_LT2_01276	cell division protein YceG	0	0	0	1	1	1	1	1	1	0	0	0	0	0	0	6	6	0	S	0.0001998001998002
SE_LT2_01284_p.Pro42Ser	SE_LT2_01284	penicillin-binding protein activator	0	0	0	1	1	1	1	1	1	0	0	0	0	0	0	6	6	0	M	0.0001998001998002
SE_LT2_01285_p.Thr10Met	SE_LT2_01285	thiamine kinase	0	0	0	1	1	1	1	1	1	0	0	0	0	0	0	6	6	0	F	0.0001998001998002
SE_LT2_01291_p.Gln59Glu	SE_LT2_01291	multiple stress resistance protein Bh	0	0	0	1	1	1	1	1	1	0	0	0	0	0	0	6	6	0	S	0.0001998001998002
SE_LT2_01292_p.Ala314Pro	SE_LT2_01292	L,D-transpeptidase	0	0	0	1	1	1	1	1	1	0	0	0	0	0	0	6	6	0	M	0.0001998001998002
SE_LT2_01301_p.Ala44Glu	SE_LT2_01301	effector protein SifA	0	0	0	1	1	1	1	1	1	0	0	0	0	0	0	6	6	0	S	0.0001998001998002
SE_LT2_01301_p.Asp133Asn	SE_LT2_01301	effector protein SifA	0	0	0	1	1	1	1	1	1	0	0	0	0	0	0	6	6	0	S	0.0001998001998002
SE_LT2_01301_p.His87Tyr	SE_LT2_01301	effector protein SifA	0	0	0	1	1	1	1	1	1	0	0	0	0	0	0	6	6	0	S	0.0001998001998002
SE_LT2_01301_p.Ile137Thr	SE_LT2_01301	effector protein SifA	0	0	0	1	1	1	1	1	1	0	0	0	0	0	0	6	6	0	S	0.0001998001998002
SE_LT2_01314_p.Arg237Cys	SE_LT2_01314	23S rRNA pseudouridine synthase E	0	0	0	1	1	1	1	1	1	0	0	0	0	0	0	6	6	0	J	0.0001998001998002
SE_LT2_01317_p.Gly26Arg	SE_LT2_01317	hypothetical protein	0	0	0	1	1	1	1	1	1	0	0	0	0	0	0	6	6	0	S	0.0001998001998002
SE_LT2_01319_p.Glu227Gly	SE_LT2_01319	lipoprotein EnvF	0	0	0	1	1	1	1	1	1	0	0	0	0	0	0	6	6	0	-	0.0001998001998002
SE_LT2_01319_p.Val235Ile	SE_LT2_01319	lipoprotein EnvF	0	0	0	1	1	1	1	1	1	0	0	0	0	0	0	6	6	0	-	0.0001998001998002
SE_LT2_01322_p.Thr8Asn	SE_LT2_01322	lipoprotein EnvE	0	0	0	1	1	1	1	1	1	0	0	0	0	0	0	6	6	0	M	0.0001998001998002
SE_LT2_01332_p.Leu208Val	SE_LT2_01332	hypothetical protein	0	0	0	1	1	1	1	1	1	0	0	0	0	0	0	6	6	0	G	0.0001998001998002
SE_LT2_01332_p.Thr34Lys	SE_LT2_01332	hypothetical protein	0	0	0	1	1	1	1	1	1	0	0	0	0	0	0	6	6	0	G	0.0001998001998002
SE_LT2_01334_p.Thr87Ala	SE_LT2_01334	hypothetical protein	0	0	0	1	1	1	1	1	1	0	0	0	0	0	0	6	6	0	M	0.0001998001998002
SE_LT2_01336_p.Leu318Gln	SE_LT2_01336	peptide ABC transporter permease	0	0	0	1	1	1	1	1	1	0	0	0	0	0	0	6	6	0	EP	0.0001998001998002
SE_LT2_01337_p.Val84Ile	SE_LT2_01337	peptide ABC transporter permease	0	0	0	1	1	1	1	1	1	0	0	0	0	0	0	6	6	0	P	0.0001998001998002
SE_LT2_01338_p.Glu136Ala	SE_LT2_01338	peptide ABC transporter ATP-bindin	0	0	0	1	1	1	1	1	1	0	0	0	0	0	0	6	6	0	P	0.0001998001998002
SE_LT2_01339_p.Ile144Val	SE_LT2_01339	peptide ABC transporter ATP-bindin	0	0	0	1	1	1	1	1	1	0	0	0	0	0	0	6	6	0	EP	0.0001998001998002
SE_LT2_01345_p.Thr223Pro	SE_LT2_01345	aminoglycoside resistance protein	0	0	0	1	1	1	1	1	1	0	0	0	0	0	0	6	6	0	H	0.0001998001998002
SE_LT2_01349_p.Ala155Thr	SE_LT2_01349	putative chorismate mutase	0	0	0	1	1	1	1	1	1	0	0	0	0	0	0	6	6	0	M	0.0001998001998002
SE_LT2_01349_p.Gln156His	SE_LT2_01349	putative chorismate mutase	0	0	0	1	1	1	1	1	1	0	0	0	0	0	0	6	6	0	M	0.0001998001998002
SE_LT2_01362_p.Gly32_Gly34del	SE_LT2_01362	hypothetical protein	0	0	0	1	1	1	1	1	1	0	0	0	0	0	0	6	6	0	-	0.0001998001998002
SE_LT2_01362_p.Val27Ile	SE_LT2_01362	hypothetical protein	0	0	0	1	1	1	1	1	1	0	0	0	0	0	0	6	6	0	-	0.0001998001998002
SE_LT2_01368_p.Ala109Ser	SE_LT2_01368	anaerobic sulfatase maturase	0	0	0	1	1	1	1	1	1	0	0	0	0	0	0	6	6	0	C	0.0001998001998002
SE_LT2_01368_p.Pro52Leu	SE_LT2_01368	anaerobic sulfatase maturase	0	0	0	1	1	1	1	1	1	0	0	0	0	0	0	6	6	0	C	0.0001998001998002
SE_LT2_01380_p.Asp357Ala	SE_LT2_01380	glutamate dehydrogenase	0	0	0	1	1	1	1	1	1	0	0	0	0	0	0	6	6	0	E	0.0001998001998002
SE_LT2_01383_p.Ser17Leu	SE_LT2_01383	exodeoxyribonuclease III	0	0	0	1	1	1	1	1	1	0	0	0	0	0	0	6	6	0	L	0.0001998001998002
SE_LT2_01384_p.Leu395Met	SE_LT2_01384	aspartate aminotransferase family p	0	0	0	1	1	1	1	0	1	0	0	0	0	0	0	5	5	0	E	0.00199800199800199
SE_LT2_01385_p.Leu44Gln	SE_LT2_01385	arginine N-succinyltransferase	0	0	0	1	1	1	1	1	1	0	0	0	0	0	0	6	6	0	E	0.0001998001998002
SE_LT2_01386_p.Gln56Glu	SE_LT2_01386	succinylglutamate-semialdehyde de	0	0	0	0	1	1	1	1	1	0	0	0	0	0	0	5	5	0	C	0.00199800199800199
SE_LT2_01386_p.Pro472Ser	SE_LT2_01386	succinylglutamate-semialdehyde de	0	0	0	1	1	1	1	1	1	0	0	0	0	0	0	6	6	0	C	0.0001998001998002
SE_LT2_01386_p.Val301Ile	SE_LT2_01386	succinylglutamate-semialdehyde de	0	0	0	1	1	1	1	1	1	0	0	0	0	0	0	6	6	0	C	0.0001998001998002
SE_LT2_01388_p.Gln188Lys	SE_LT2_01388	succinylglutamate desuccinylase	0	0	0	1	1	1	1	1	1	0	0	0	0	0	0	6	6	0	E	0.0001998001998002
SE_LT2_01389_p.Met69Leu	SE_LT2_01389	hypothetical protein	0	0	0	1	1	1	1	1	1	0	0	0	0	0	0	6	6	0	-	0.0001998001998002
SE_LT2_01391_p.Gln292Arg	SE_LT2_01391	putative nuclease subunit of the exc	0	0	0	1	1	1	1	1	1	0	0	0	0	0	0	6	6	0	L	0.0001998001998002
SE_LT2_01400_p.Gly326Ser	SE_LT2_01400	catalase HPII	0	0	0	1	1	1	1	1	1	0	0	0	0	0	0	6	6	0	P	0.0001998001998002
SE_LT2_01407_p.Thr14Asn	SE_LT2_01407	hypothetical protein	0	0	0	1	1	1	1	1	1	0	0	0	0	0	0	6	6	0	S	0.0001998001998002
SE_LT2_01425_p.Val159Ile	SE_LT2_01425	glutathione peroxidase	0	0	0	1	1	1	1	1	1	0	0	0	0	0	0	6	6	0	O	0.0001998001998002
SE_LT2_01439_p.Glu288Ala	SE_LT2_01439	AraC family transcriptional regulator	0	0	0	1	1	1	1	1	1	0	0	0	0	0	0	6	6	0	K	0.0001998001998002
SE_LT2_01445_p.Ala364Thr	SE_LT2_01445	MFS transporter	0	0	0	1	1	1	1	1	1	0	0	0	0	0	0	6	6	0	EGP	0.0001998001998002
SE_LT2_01449_p.Arg981Gly	SE_LT2_01449	putative protein	0	0	0	1	1	1	1	1	1	0	0	0	0	0	0	6	6	0	C	0.0001998001998002
SE_LT2_01453_p.Ala9Val	SE_LT2_01453	L-cystine transporter	0	0	0	1	1	1	1	1	1	0	0	0	0	0	0	6	6	0	U	0.0001998001998002
SE_LT2_01455_p.Glu139Lys	SE_LT2_01455	Fe-S cluster assembly protein SufB	0	0	0	1	1	1	1	1	1	0	0	0	0	0	0	6	6	0	O	0.0001998001998002
SE_LT2_01458_p.Pro328Leu	SE_LT2_01458	bifunctional cysteine desulfurase/se	0	0	0	1	1	1	1	1	1	0	0	0	0	0	0	6	6	0	H	0.0001998001998002
SE_LT2_01459_p.Ala18Thr	SE_LT2_01459	cysteine desufuration protein SufE	0	0	0	1	1	1	1	1	1	0	0	0	0	0	0	6	6	0	S	0.0001998001998002
SE_LT2_01460_p.Pro267Ser	SE_LT2_01460	murein L,D-transpeptidase	0	0	0	1	1	1	1	1	1	0	0	0	0	0	0	6	6	0	S	0.0001998001998002
SE_LT2_01474_p.Val103Ala	SE_LT2_01474	hypothetical protein	0	0	0	1	1	1	1	1	1	0	0	0	0	0	0	6	6	0	S	0.0001998001998002

SE_LT2_01519_p.Val129Ala	SE_LT2_01519	putative cell wall-associated hydrola	0	0	0	1	1	1	1	1	1	0	0	0	0	0	0	6	6	0	M	0.0001998001998002
SE_LT2_01523_p.Ala93Asp	SE_LT2_01523	N-ethylmaleimide reductase	0	0	0	1	1	1	1	1	1	0	0	0	0	0	0	6	6	0	C	0.0001998001998002
SE_LT2_01523_p.Asp153Glu	SE_LT2_01523	N-ethylmaleimide reductase	0	0	0	1	1	1	1	1	1	0	0	0	0	0	0	6	6	0	C	0.0001998001998002
SE_LT2_01524_p.Asp143Glu	SE_LT2_01524	TetR family transcriptional regulator	0	0	0	1	1	1	1	1	1	0	0	0	0	0	0	6	6	0	K	0.0001998001998002
SE_LT2_01534_p.Asn111Asp	SE_LT2_01534	lysozyme inhibitor	0	0	0	1	1	1	1	1	1	0	0	0	0	0	0	6	6	0	S	0.0001998001998002
SE_LT2_01536_p.Ser392Pro	SE_LT2_01536	tyrosine--tRNA ligase	0	0	0	1	1	1	1	1	1	0	0	0	0	0	0	6	6	0	J	0.0001998001998002
SE_LT2_01546_p.Val149Leu	SE_LT2_01546	electron transport complex subunit	0	0	0	1	1	1	1	1	1	0	0	0	0	0	0	6	6	0	C	0.0001998001998002
SE_LT2_01557_p.Gln240Glu	SE_LT2_01557	putative periplasmic protein	0	0	0	1	1	1	1	1	1	0	0	0	0	0	0	6	6	0	S	0.0001998001998002
SE_LT2_01557_p.His514Asn	SE_LT2_01557	putative periplasmic protein	0	0	0	1	1	1	1	1	1	0	0	0	0	0	0	6	6	0	S	0.0001998001998002
SE_LT2_01558_p.Gly229Glu	SE_LT2_01558	putative porin	0	0	0	1	1	1	1	1	1	0	0	0	0	0	0	6	6	0	M	0.0001998001998002
SE_LT2_01574_p.Ala128Ser	SE_LT2_01574	voltage-gated ClC-type chloride cha	0	0	0	1	0	1	1	1	1	0	0	0	0	0	0	5	5	0	P	0.00199800199800199
SE_LT2_01582_p.Asp681Asn	SE_LT2_01582	dimethyl sulfoxide reductase subun	0	0	0	1	0	1	1	1	1	0	0	0	0	0	0	5	5	0	C	0.00199800199800199
SE_LT2_01597_p.Ala254Val	SE_LT2_01597	dipeptidyl carboxypeptidase II	0	0	0	1	1	1	1	1	1	0	0	0	0	0	0	6	6	0	E	0.0001998001998002
SE_LT2_01598_p.Glu16Gln	SE_LT2_01598	stress-induced acidophilic repeat m	0	0	0	1	1	1	1	1	1	0	0	0	0	0	0	6	6	0	S	0.0001998001998002
SE_LT2_01603_p.Ser15Ala	SE_LT2_01603	transporter	0	0	0	1	1	1	1	1	1	0	0	0	0	0	0	6	6	0	EGP	0.0001998001998002
SE_LT2_01609_p.Ala325Ser	SE_LT2_01609	sugar transporter	0	0	0	1	1	1	1	1	1	0	0	0	0	0	0	6	6	0	EGP	0.0001998001998002
SE_LT2_01610_p.Thr289Ala	SE_LT2_01610	putative LysR family transcriptional	0	0	0	1	1	1	1	1	1	0	0	0	0	0	0	6	6	0	K	0.0001998001998002
SE_LT2_01612_p.Gln87Leu	SE_LT2_01612	putative glutaminase	0	0	0	1	1	1	1	0	1	0	0	0	0	0	0	5	5	0	E	0.00199800199800199
SE_LT2_01614_p.Arg311His	SE_LT2_01614	putative inner membrane protein	0	0	0	1	1	1	1	1	1	0	0	0	0	0	0	6	6	0	S	0.0001998001998002
SE_LT2_01614_p.Glu155Gly	SE_LT2_01614	putative inner membrane protein	0	0	0	1	1	1	1	1	1	0	0	0	0	0	0	6	6	0	S	0.0001998001998002
SE_LT2_01614_p.Val125Ile	SE_LT2_01614	putative inner membrane protein	0	0	0	1	1	1	1	1	1	0	0	0	0	0	0	6	6	0	S	0.0001998001998002
SE_LT2_01615_p.Glu41Lys	SE_LT2_01615	hypothetical protein	0	0	0	1	1	1	1	1	1	0	0	0	0	0	0	6	6	0	S	0.0001998001998002
SE_LT2_01620_p.Val9Leu	SE_LT2_01620	hydrogenase-1 operon protein HyaF	0	0	0	1	0	1	1	1	1	0	0	0	0	0	0	5	5	0	C	0.00199800199800199
SE_LT2_01628_p.Gly190Ser	SE_LT2_01628	putative hydrolase	0	0	0	1	1	1	1	1	1	0	0	0	0	0	0	6	6	0	M	0.0001998001998002
SE_LT2_01630_p.Glu26Lys	SE_LT2_01630	alcohol dehydrogenase	0	0	0	1	1	1	1	1	1	0	0	0	0	0	0	6	6	0	E	0.0001998001998002
SE_LT2_01632_p.Pro52Ser	SE_LT2_01632	PhoPQ-regulated protein	0	0	0	1	1	1	1	1	1	0	0	0	0	0	0	6	6	0	S	0.0001998001998002
SE_LT2_01635_p.Val84Ile	SE_LT2_01635	MarR family transcriptional regulato	0	0	0	1	1	1	1	1	1	0	0	0	0	0	0	6	6	0	K	0.0001998001998002
SE_LT2_01636_p.Asn236His	SE_LT2_01636	putative S-adenosylmethionine:tRN	0	0	0	1	1	1	1	1	1	0	0	0	0	0	0	6	6	0	H	0.0001998001998002
SE_LT2_01647_p.Arg314His	SE_LT2_01647	Na+/H+ antiporter NhaC	0	0	0	1	1	1	1	1	1	0	0	0	0	0	0	6	6	0	C	0.0001998001998002
SE_LT2_01648_p.Glu311Val	SE_LT2_01648	putative aminotransferase	0	0	0	1	1	1	1	1	1	0	0	0	0	0	0	6	6	0	E	0.0001998001998002
SE_LT2_01648_p.Thr282Ile	SE_LT2_01648	putative aminotransferase	0	0	0	1	1	1	1	1	1	0	0	0	0	0	0	6	6	0	E	0.0001998001998002
SE_LT2_01650_p.Ser789Pro	SE_LT2_01650	malto-oligosyltrehalose synthase	0	0	0	1	1	1	1	1	1	0	0	0	0	0	0	6	6	0	G	0.0001998001998002
SE_LT2_01651_p.Thr59Met	SE_LT2_01651	malto-oligosyltrehalose trehalohydı	0	0	0	1	1	1	1	1	1	0	0	0	0	0	0	6	6	0	G	0.0001998001998002
SE_LT2_01655_p.Ter72Ter	SE_LT2_01655	biofilm-dependent modulation prot	0	0	0	1	1	1	1	1	1	0	0	0	0	0	0	6	6	0	S	0.0001998001998002
SE_LT2_01661_p.AspAspPheAlal	SE_LT2_01661	putative molybdopterin oxidoreduc	0	0	0	1	1	1	1	1	1	0	0	0	0	0	0	6	6	0	C	0.0001998001998002
SE_LT2_01664_p.Ala162Glu	SE_LT2_01664	putative permease	0	0	0	1	1	1	1	1	1	0	0	0	0	0	0	6	6	0	EG	0.0001998001998002
SE_LT2_01665_p.Asp180Glu	SE_LT2_01665	phosphoporin PhoE	0	0	0	1	0	1	1	1	1	0	0	0	0	0	0	5	5	0	M	0.00199800199800199
SE_LT2_01666_p.Ser55Ala	SE_LT2_01666	putative cytoplasmic protein	0	0	0	1	1	1	1	1	1	0	0	0	0	0	0	6	6	0	S	0.0001998001998002
SE_LT2_01670_p.Ala675Val	SE_LT2_01670	nitrate reductase subunit alpha	0	0	0	1	1	1	1	1	1	0	0	0	0	0	0	6	6	0	C	0.0001998001998002
SE_LT2_01670_p.Arg459Cys	SE_LT2_01670	nitrate reductase subunit alpha	0	0	0	1	1	1	1	1	1	0	0	0	0	0	0	6	6	0	C	0.0001998001998002
SE_LT2_01670_p.His27Tyr	SE_LT2_01670	nitrate reductase subunit alpha	0	0	0	1	0	1	1	1	1	0	0	0	0	0	0	5	5	0	C	0.00199800199800199
SE_LT2_01670_p.Thr895Ile	SE_LT2_01670	nitrate reductase subunit alpha	0	0	0	1	1	1	1	1	1	0	0	0	0	0	0	6	6	0	C	0.0001998001998002
SE_LT2_01671_p.Leu146Pro	SE_LT2_01671	nitrate reductase subunit beta	0	0	0	1	1	1	1	1	1	0	0	0	0	0	0	6	6	0	C	0.0001998001998002
SE_LT2_01672_p.Asp86Asn	SE_LT2_01672	nitrate reductase molybdenum cofa	0	0	0	1	0	1	1	1	1	0	0	0	0	0	0	5	5	0	C	0.00199800199800199
SE_LT2_01680_p.Ala717Thr	SE_LT2_01680	TonB-dependent receptor	0	0	0	1	1	1	1	1	1	0	0	0	0	0	0	6	6	0	P	0.0001998001998002
SE_LT2_01682_p.Gly71Asp	SE_LT2_01682	putative NADP-dependent oxidored	0	0	0	1	1	1	1	1	1	0	0	0	0	0	0	6	6	0	S	0.0001998001998002
SE_LT2_01682_p.Val33Ile	SE_LT2_01682	putative NADP-dependent oxidored	0	0	0	1	1	1	1	1	1	0	0	0	0	0	0	6	6	0	S	0.0001998001998002
SE_LT2_01685_p.Val43Ala	SE_LT2_01685	putative protein YdcY	0	0	0	1	1	1	1	1	1	0	0	0	0	0	0	6	6	0	S	0.0001998001998002
SE_LT2_01686_p.Asp151Asn	SE_LT2_01686	ssrAB-activated protein	0	0	0	1	1	1	1	1	1	0	0	0	0	0	0	6	6	0	S	0.0001998001998002
SE_LT2_01686_p.Val159Ile	SE_LT2_01686	ssrAB-activated protein	0	0	0	1	0	1	1	1	1	0	0	0	0	0	0	5	5	0	S	0.00199800199800199

SE_LT2_01687_p.Ala126Val	SE_LT2_01687	virulence factor SrfB	0	0	0	1	1	1	1	1	1	0	0	0	0	0	0	6	6	0	S	0.0001998001998002
SE_LT2_01690_p.Arg203Leu	SE_LT2_01690	putative aldehyde dehydrogenase	0	0	0	1	1	1	1	1	1	0	0	0	0	0	0	6	6	0	C	0.0001998001998002
SE_LT2_01691_p.Ala189Val	SE_LT2_01691	GntR family transcriptional regulato	0	0	0	1	1	1	1	1	1	0	0	0	0	0	0	6	6	0	K	0.0001998001998002
SE_LT2_01697_p.Asn145Thr	SE_LT2_01697	protease	0	0	0	1	1	1	1	1	1	0	0	0	0	0	0	6	6	0	O	0.0001998001998002
SE_LT2_01699_p.Ala374Val	SE_LT2_01699	benzoate transporter	0	0	0	1	1	1	1	1	0	0	0	0	0	0	0	5	5	0	Q	0.00199800199800199
SE_LT2_01699_p.Leu34Ile	SE_LT2_01699	benzoate transporter	0	0	0	1	1	1	1	1	1	0	0	0	0	0	0	6	6	0	Q	0.0001998001998002
SE_LT2_01699_p.Val170Ile	SE_LT2_01699	benzoate transporter	0	0	0	1	1	1	1	1	1	0	0	0	0	0	0	6	6	0	Q	0.0001998001998002
SE_LT2_01700_p.Ala174Val	SE_LT2_01700	hypothetical protein	0	0	0	1	1	1	1	1	1	0	0	0	0	0	0	6	6	0	M	0.0001998001998002
SE_LT2_01702_p.Ala98Ser	SE_LT2_01702	dicarboxylate transporter/tellurite-	0	0	0	1	1	1	1	1	1	0	0	0	0	0	0	6	6	0	P	0.0001998001998002
SE_LT2_01703_p.Ala66Thr	SE_LT2_01703	hypothetical protein	0	0	0	1	1	1	1	1	1	0	0	0	0	0	0	6	6	0	M	0.0001998001998002
SE_LT2_01703_p.Arg11Cys	SE_LT2_01703	hypothetical protein	0	0	0	1	1	1	1	1	1	0	0	0	0	0	0	6	6	0	M	0.0001998001998002
SE_LT2_01703_p.Val141Ile	SE_LT2_01703	hypothetical protein	0	0	0	1	1	1	1	1	1	0	0	0	0	0	0	6	6	0	M	0.0001998001998002
SE_LT2_01704_p.Ala33Thr	SE_LT2_01704	ribosomal protein L7/L12-serine ace	0	0	0	1	1	1	1	1	1	0	0	0	0	0	0	6	6	0	J	0.0001998001998002
SE_LT2_01707_p.Ala387Thr	SE_LT2_01707	PTS sugar transporter subunit IIC	0	0	0	1	1	1	1	1	1	0	0	0	0	0	0	6	6	0	G	0.0001998001998002
SE_LT2_01715_p.Ala395Val	SE_LT2_01715	glucan biosynthesis protein D	0	0	0	1	1	1	1	1	1	0	0	0	0	0	0	6	6	0	P	0.0001998001998002
SE_LT2_01715_p.Gln43Leu	SE_LT2_01715	glucan biosynthesis protein D	0	0	0	1	1	1	1	1	1	0	0	0	0	0	0	6	6	0	P	0.0001998001998002
SE_LT2_01717_p.Thr384Ile	SE_LT2_01717	hypothetical protein	0	0	0	1	0	1	1	1	1	0	0	0	0	0	0	5	5	0	S	0.00199800199800199
SE_LT2_01717_p.Thr405Met	SE_LT2_01717	hypothetical protein	0	0	0	1	0	1	1	1	1	0	0	0	0	0	0	5	5	0	S	0.00199800199800199
SE_LT2_01724_p.Arg226Ile	SE_LT2_01724	pathogenicity island 2 effector prote	0	0	0	1	1	1	1	1	1	0	0	0	0	0	0	6	6	0	I	0.0001998001998002
SE_LT2_01735_p.Ala83Thr	SE_LT2_01735	Protein YdcF	0	0	0	1	1	1	1	1	1	0	0	0	0	0	0	6	6	0	S	0.0001998001998002
SE_LT2_01741_p.Glu593Lys	SE_LT2_01741	hypothetical protein	0	0	0	1	1	1	1	1	1	0	0	0	0	0	0	6	6	0	S	0.0001998001998002
SE_LT2_01742_p.Ala323Gly	SE_LT2_01742	2-hydroxyacid dehydrogenase	0	0	0	1	1	1	1	1	1	0	0	0	0	0	0	6	6	0	CH	0.0001998001998002
SE_LT2_01745_p.Glu1144Lys	SE_LT2_01745	putative pyruvate-flavodoxin oxido	0	0	0	1	1	1	1	1	1	0	0	0	0	0	0	6	6	0	C	0.0001998001998002
SE_LT2_01749_p.Val272Ile	SE_LT2_01749	tRNA 2-thiocytidine(32) synthetase	0	0	0	1	1	1	1	1	1	0	0	0	0	0	0	6	6	0	J	0.0001998001998002
SE_LT2_01750_p.Gln255Glu	SE_LT2_01750	ATP-dependent RNA helicase DbpA	0	0	0	1	1	1	1	1	1	0	0	0	0	0	0	6	6	0	F	0.0001998001998002
SE_LT2_01753_p.Gln359Pro	SE_LT2_01753	chemoreceptor protein	0	0	0	1	1	1	1	1	1	0	0	0	0	0	0	6	6	0	NT	0.0001998001998002
SE_LT2_01758_p.Met4Ile	SE_LT2_01758	hypothetical protein	0	0	0	1	1	1	1	1	1	0	0	0	0	0	0	6	6	0	S	0.0001998001998002
SE_LT2_01760_p.Glu20Ala	SE_LT2_01760	XRE family transcriptional regulator	0	0	0	1	1	1	1	1	1	0	0	0	0	0	0	6	6	0	K	0.0001998001998002
SE_LT2_01760_p.Met35Ile	SE_LT2_01760	XRE family transcriptional regulator	0	0	0	1	1	1	1	1	1	0	0	0	0	0	0	6	6	0	K	0.0001998001998002
SE_LT2_01761_p.Ala96Ser	SE_LT2_01761	hypothetical protein	0	0	0	1	1	1	1	1	1	0	0	0	0	0	0	6	6	0	S	0.0001998001998002
SE_LT2_01761_p.ThrValLeu65Ala	SE_LT2_01761	hypothetical protein	0	0	0	0	1	1	1	1	1	0	0	0	0	0	0	5	5	0	S	0.00199800199800199
SE_LT2_01762_p.Ile31Val	SE_LT2_01762	putative thiol peroxidase	0	0	0	1	1	1	1	1	1	0	0	0	0	0	0	6	6	0	O	0.0001998001998002
SE_LT2_01764_p.Gln110Glu	SE_LT2_01764	invasin	0	0	0	1	1	1	1	0	1	0	0	0	0	0	0	5	5	0	S	0.00199800199800199
SE_LT2_01764_p.His467Tyr	SE_LT2_01764	invasin	0	0	0	1	1	1	1	1	1	0	0	0	0	0	0	6	6	0	S	0.0001998001998002
SE_LT2_01764_p.Leu562Ile	SE_LT2_01764	invasin	0	0	0	1	1	1	1	1	1	0	0	0	0	0	0	6	6	0	S	0.0001998001998002
SE_LT2_01764_p.Val17Ile	SE_LT2_01764	invasin	0	0	0	1	1	1	1	1	1	0	0	0	0	0	0	6	6	0	S	0.0001998001998002
SE_LT2_01771_p.Val191Glu	SE_LT2_01771	aldo/keto reductase	0	0	0	1	1	1	1	1	1	0	0	0	0	0	0	6	6	0	S	0.0001998001998002
SE_LT2_01772_p.Leu115Trp	SE_LT2_01772	putative LysR family transcriptional	0	0	0	1	1	1	1	1	1	0	0	0	0	0	0	6	6	0	K	0.0001998001998002
SE_LT2_01772_p.ProAla297SerGl	SE_LT2_01772	putative LysR family transcriptional	0	0	0	1	1	1	1	1	1	0	0	0	0	0	0	6	6	0	K	0.0001998001998002
SE_LT2_01775_p.Glu121Lys	SE_LT2_01775	murein peptide amidase A	0	0	0	1	1	1	1	1	1	0	0	0	0	0	0	6	6	0	E	0.0001998001998002
SE_LT2_01777_p.Val165Ile	SE_LT2_01777	lipid hydroperoxide peroxidase	0	0	0	1	1	1	1	1	1	0	0	0	0	0	0	6	6	0	O	0.0001998001998002
SE_LT2_01780_p.Ser428Lys	SE_LT2_01780	putative ATPase	0	0	0	1	0	1	1	1	1	0	0	0	0	0	0	5	5	0	S	0.00199800199800199
SE_LT2_01787_p.Asp353Gly	SE_LT2_01787	peptide ABC transporter substrate-b	0	0	0	1	1	1	1	1	1	0	0	0	0	0	0	6	6	0	E	0.0001998001998002
SE_LT2_01794_p.Asn405Asp	SE_LT2_01794	secreted effector kinase SteC	0	0	0	1	1	1	1	1	1	0	0	0	0	0	0	6	6	0	-	0.0001998001998002
SE_LT2_01794_p.Ile366Met	SE_LT2_01794	secreted effector kinase SteC	0	0	0	1	1	1	1	1	1	0	0	0	0	0	0	6	6	0	-	0.0001998001998002
SE_LT2_01806_p.Val24Gly	SE_LT2_01806	phosphatidylglycerophosphatase B	0	0	0	1	1	1	1	1	1	0	0	0	0	0	0	6	6	0	I	0.0001998001998002
SE_LT2_01808_p.Ile566Val	SE_LT2_01808	aconitate hydratase 1	0	0	0	1	1	1	1	1	1	0	0	0	0	0	0	6	6	0	C	0.0001998001998002
SE_LT2_01808_p.Ser602Leu	SE_LT2_01808	aconitate hydratase 1	0	0	0	1	1	1	1	1	1	0	0	0	0	0	0	6	6	0	C	0.0001998001998002
SE_LT2_01814_p.His253Tyr	SE_LT2_01814	protease SohB	0	0	0	1	1	1	1	1	1	0	0	0	0	0	0	6	6	0	OU	0.0001998001998002
SE_LT2_01815_p.Ser187Pro	SE_LT2_01815	YciK family oxidoreductase	0	0	0	1	1	1	1	1	1	0	0	0	0	0	0	6	6	0	IQ	0.0001998001998002

SE_LT2_01819_p.Glu105His	SE_LT2_01819	phosphatase	0	0	0	1	1	1	1	1	1	0	0	0	0	0	0	6	6	0	S	0.0001998001998002
SE_LT2_01821_p.Ala63Ser	SE_LT2_01821	anthranilate synthase subunit I	0	0	0	1	1	1	1	1	1	0	0	0	0	0	0	6	6	0	E	0.0001998001998002
SE_LT2_01821_p.Arg203Leu	SE_LT2_01821	anthranilate synthase subunit I	0	0	0	1	1	1	1	1	1	0	0	0	0	0	0	6	6	0	E	0.0001998001998002
SE_LT2_01824_p.Val387Ile	SE_LT2_01824	tryptophan synthase subunit beta	0	0	0	1	1	1	1	1	1	0	0	0	0	0	0	6	6	0	E	0.0001998001998002
SE_LT2_01838_p.Val18Ile	SE_LT2_01838	cardiolipin synthase	0	0	0	1	1	1	1	1	1	0	0	0	0	0	0	6	6	0	I	0.0001998001998002
SE_LT2_01866_p.Pro680Ser	SE_LT2_01866	nitrate reductase subunit alpha	0	0	0	1	1	1	1	1	1	0	0	0	0	0	0	6	6	0	C	0.0001998001998002
SE_LT2_01888_p.Gln363Pro	SE_LT2_01888	[Ni/Fe] hydrogenase small subunit	0	0	0	1	1	1	1	1	1	0	0	0	0	0	0	6	6	0	C	0.0001998001998002
SE_LT2_01891_p.Gln147Leu	SE_LT2_01891	hydrogenase expression/formation	0	0	0	1	1	1	1	1	1	0	0	0	0	0	0	6	6	0	C	0.0001998001998002
SE_LT2_01895_p.Asp156Asn	SE_LT2_01895	cytochrome d ubiquinol oxidase sub	0	0	0	1	1	1	1	1	1	0	0	0	0	0	0	6	6	0	C	0.0001998001998002
SE_LT2_01895_p.Ser317Asn	SE_LT2_01895	cytochrome d ubiquinol oxidase sub	0	0	0	1	1	1	1	1	1	0	0	0	0	0	0	6	6	0	C	0.0001998001998002
SE_LT2_01901_p.Ile12Met	SE_LT2_01901	lytic murein transglycosylase	0	0	0	1	1	1	1	1	1	0	0	0	0	0	0	6	6	0	M	0.0001998001998002
SE_LT2_01902_p.Ala119Val	SE_LT2_01902	muramoyltetrapeptide carboxypept	0	0	0	1	0	1	1	1	1	0	0	0	0	0	0	5	5	0	V	0.00199800199800199
SE_LT2_01902_p.Val121Ile	SE_LT2_01902	muramoyltetrapeptide carboxypept	0	0	0	1	0	1	1	1	1	0	0	0	0	0	0	5	5	0	V	0.00199800199800199
SE_LT2_01904_p.Tyr187His	SE_LT2_01904	alanine racemase	0	0	0	1	1	1	1	1	1	0	0	0	0	0	0	6	6	0	E	0.0001998001998002
SE_LT2_01908_p.Val159Ala	SE_LT2_01908	Na+/H+ antiporter NhaB	0	0	0	1	1	1	1	1	1	0	0	0	0	0	0	6	6	0	P	0.0001998001998002
SE_LT2_01908_p.Val31Ile	SE_LT2_01908	Na+/H+ antiporter NhaB	0	0	0	1	1	1	1	1	1	0	0	0	0	0	0	6	6	0	P	0.0001998001998002
SE_LT2_01914_p.Arg90Cys	SE_LT2_01914	isomerase/hydrolase	0	0	0	1	1	1	1	1	1	0	0	0	0	0	0	6	6	0	Q	0.0001998001998002
SE_LT2_01915_p.Pro94His	SE_LT2_01915	putative cytoplasmic protein	0	0	0	1	1	1	1	1	1	0	0	0	0	0	0	6	6	0	S	0.0001998001998002
SE_LT2_01924_p.Ser325Leu	SE_LT2_01924	ATP-dependent helicase	0	0	0	1	0	1	1	1	1	0	0	0	0	0	0	5	5	0	F	0.00199800199800199
SE_LT2_01927_p.Glu435Asp	SE_LT2_01927	p-aminobenzoate synthetase, comp	0	0	0	1	1	1	1	1	1	0	0	0	0	0	0	6	6	0	EH	0.0001998001998002
SE_LT2_01929_p.Gly72Ser	SE_LT2_01929	L-serine ammonia-lyase	0	0	0	1	1	1	1	1	1	0	0	0	0	0	0	6	6	0	E	0.0001998001998002
SE_LT2_01935_p.Ser64Gly	SE_LT2_01935	hypothetical protein	0	0	0	1	1	1	1	1	1	0	0	0	0	0	0	6	6	0	S	0.0001998001998002
SE_LT2_01936_p.Val179Ala	SE_LT2_01936	putative YebN family transport prot	0	0	0	1	1	1	1	1	1	0	0	0	0	0	0	6	6	0	P	0.0001998001998002
SE_LT2_01949_p.Ala176Gly	SE_LT2_01949	putative GAF domain-containing pro	0	0	0	1	1	1	0	1	1	0	0	0	0	0	0	5	5	0	T	0.00199800199800199
SE_LT2_01959_p.Ala79Asp	SE_LT2_01959	putative protein YdfO	0	0	0	1	1	1	1	1	1	0	0	0	0	0	0	6	6	0	S	0.0001998001998002
SE_LT2_01988_p.Gly75Ser	SE_LT2_01988	damage-inducible protein YebG	0	0	0	1	1	1	1	1	1	0	0	0	0	0	0	6	6	0	S	0.0001998001998002
SE_LT2_01992_p.Ser100Asn	SE_LT2_01992	glucose-6-phosphate dehydrogenas	0	0	0	1	1	1	1	1	1	0	0	0	0	0	0	6	6	0	G	0.0001998001998002
SE_LT2_01997_p.Ala317Val	SE_LT2_01997	ABC superfamily high affinity Zn tra	0	0	0	1	1	1	1	1	1	0	0	0	0	0	0	6	6	0	P	0.0001998001998002
SE_LT2_02012_p.Gly309Asp	SE_LT2_02012	tRNA 5-methoxyuridine(34)/uridine	0	0	0	1	1	1	1	1	1	0	0	0	0	0	0	6	6	0	J	0.0001998001998002
SE_LT2_02012_p.Leu168Phe	SE_LT2_02012	tRNA 5-methoxyuridine(34)/uridine	0	0	0	1	1	1	1	1	1	0	0	0	0	0	0	6	6	0	J	0.0001998001998002
SE_LT2_02018_p.Tyr75His	SE_LT2_02018	glycoside hydrolase 105 family prote	0	0	0	1	1	1	1	1	1	0	0	0	0	0	0	6	6	0	S	0.0001998001998002
SE_LT2_02019_p.Gly69Ser	SE_LT2_02019	flagellar protein FlhE	0	0	0	1	0	1	1	1	1	0	0	0	0	0	0	5	5	0	N	0.00199800199800199
SE_LT2_02035_p.Asp66Asn	SE_LT2_02035	trehalose-phosphatase	0	0	0	1	1	1	1	1	1	0	0	0	0	0	0	6	6	0	G	0.0001998001998002
SE_LT2_02036_p.Ala160Gly	SE_LT2_02036	DJ-1 family protein	0	0	0	1	1	1	1	1	1	0	0	0	0	0	0	6	6	0	S	0.0001998001998002
SE_LT2_02042_p.Thr326Met	SE_LT2_02042	tyrosine transporter TyrP	0	0	0	1	1	1	1	0	1	0	0	0	0	0	0	5	5	0	U	0.00199800199800199
SE_LT2_02043_p.Asp77Glu	SE_LT2_02043	hypothetical protein	0	0	0	1	1	1	1	1	1	0	0	0	0	0	0	6	6	0	S	0.0001998001998002
SE_LT2_02045_p.Glu52Asp	SE_LT2_02045	putative glucose-6-phosphate dehy	0	0	0	1	1	1	1	1	1	0	0	0	0	0	0	6	6	0	-	0.0001998001998002
SE_LT2_02046_p.Ala103Thr	SE_LT2_02046	putative cell wall-associated hydrol	0	0	0	1	1	1	1	1	1	0	0	0	0	0	0	6	6	0	M	0.0001998001998002
SE_LT2_02046_p.PheThrGly32Va	SE_LT2_02046	putative cell wall-associated hydrol	0	0	0	1	1	1	1	1	1	0	0	0	0	0	0	6	6	0	M	0.0001998001998002
SE_LT2_02052_p.Ala189Thr	SE_LT2_02052	excinuclease ABC subunit C	0	0	0	1	1	1	1	1	1	0	0	0	0	0	0	6	6	0	L	0.0001998001998002
SE_LT2_02052_p.Ala500Thr	SE_LT2_02052	excinuclease ABC subunit C	0	0	0	1	1	1	1	1	1	0	0	0	0	0	0	6	6	0	L	0.0001998001998002
SE_LT2_02052_p.Leu336Phe	SE_LT2_02052	excinuclease ABC subunit C	0	0	0	1	1	1	1	1	1	0	0	0	0	0	0	6	6	0	L	0.0001998001998002
SE_LT2_02055_p.Phe135Val	SE_LT2_02055	transcriptional regulator SdiA	0	0	0	1	1	1	1	1	1	0	0	0	0	0	0	6	6	0	K	0.0001998001998002
SE_LT2_02056_p.Ala119Val	SE_LT2_02056	L-cystine ABC transporter ATP-bindi	0	0	0	1	1	1	1	1	1	0	0	0	0	0	0	6	6	0	E	0.0001998001998002
SE_LT2_02057_p.Arg52Leu	SE_LT2_02057	amino acid ABC transporter permea	0	0	0	1	1	1	1	1	1	0	0	0	0	0	0	6	6	0	P	0.0001998001998002
SE_LT2_02064_p.Ala10Val	SE_LT2_02064	lysine-N-methylase	0	0	0	1	1	1	1	1	1	0	0	0	0	0	0	6	6	0	H	0.0001998001998002
SE_LT2_02064_p.Ser348Cys	SE_LT2_02064	lysine-N-methylase	0	0	0	1	1	1	1	1	1	0	0	0	0	0	0	6	6	0	H	0.0001998001998002
SE_LT2_02069_p.Arg397Gln	SE_LT2_02069	alpha-amylase	0	0	0	1	1	1	1	1	1	0	0	0	0	0	0	6	6	0	G	0.0001998001998002
SE_LT2_02076_p.Gln89Arg	SE_LT2_02076	flagellar assembly protein FliH	0	0	0	1	1	1	1	1	1	0	0	0	0	0	0	6	6	0	N	0.0001998001998002
SE_LT2_02086_p.Val23Ile	SE_LT2_02086	flagellar biosynthetic protein FliR	0	0	0	1	1	1	1	1	1	0	0	0	0	0	0	6	6	0	N	0.0001998001998002

SE_LT2_02098_p.Ala198Thr	SE_LT2_02098	phosphohydrolase	0	0	0	1	1	1	1	1	1	0	0	0	0	0	0	6	6	0	S	0.0001998001998002
SE_LT2_02124_p.Pro57Ser	SE_LT2_02124	adenosylcobinamide-GDP ribazoletr	0	0	0	1	1	1	1	1	1	0	0	0	0	0	0	6	6	0	H	0.0001998001998002
SE_LT2_02125_p.Glu97Ala	SE_LT2_02125	bifunctional adenosylcobinamide ki	0	0	0	1	1	1	1	1	1	0	0	0	0	0	0	6	6	0	H	0.0001998001998002
SE_LT2_02126_p.Thr289Ala	SE_LT2_02126	synthesis of vitamin B12 adenosyl co	0	0	0	1	1	1	1	1	1	0	0	0	0	0	0	6	6	0	H	0.0001998001998002
SE_LT2_02126_p.Thr497Ser	SE_LT2_02126	synthesis of vitamin B12 adenosyl co	0	0	0	1	1	1	1	1	1	0	0	0	0	0	0	6	6	0	H	0.0001998001998002
SE_LT2_02126_p.Val220Ile	SE_LT2_02126	synthesis of vitamin B12 adenosyl co	0	0	0	1	1	1	1	1	1	0	0	0	0	0	0	6	6	0	H	0.0001998001998002
SE_LT2_02144_p.His98Tyr	SE_LT2_02144	HTH-type transcriptional activator Rl	0	0	0	1	1	1	1	1	1	0	0	0	0	0	0	6	6	0	K	0.0001998001998002
SE_LT2_02155_p.Glu115Asp	SE_LT2_02155	propanediol utilization protein	0	0	0	1	1	1	1	1	1	0	0	0	0	0	0	6	6	0	CQ	0.0001998001998002
SE_LT2_02155_p.Ile104Met	SE_LT2_02155	propanediol utilization protein	0	0	0	1	1	1	1	1	1	0	0	0	0	0	0	6	6	0	CQ	0.0001998001998002
SE_LT2_02155_p.Thr89Ala	SE_LT2_02155	propanediol utilization protein	0	0	0	1	1	1	1	1	1	0	0	0	0	0	0	6	6	0	CQ	0.0001998001998002
SE_LT2_02157_p.Arg82Cys	SE_LT2_02157	microcompartment protein PduM	0	0	0	0	1	1	1	1	1	0	0	0	0	0	0	5	5	0	S	0.00199800199800199
SE_LT2_02157_p.Thr5Ile	SE_LT2_02157	microcompartment protein PduM	0	0	0	1	1	1	1	1	1	0	0	0	0	0	0	6	6	0	S	0.0001998001998002
SE_LT2_02159_p.His192Arg	SE_LT2_02159	ATP:cob(I)alamin adenosyltransfera	0	0	0	1	1	1	1	1	1	0	0	0	0	0	0	6	6	0	S	0.0001998001998002
SE_LT2_02159_p.Pro198Ser	SE_LT2_02159	ATP:cob(I)alamin adenosyltransfera	0	0	0	1	1	1	1	1	1	0	0	0	0	0	0	6	6	0	S	0.0001998001998002
SE_LT2_02159_p.Thr55Ala	SE_LT2_02159	ATP:cob(I)alamin adenosyltransfera	0	0	0	1	0	1	1	1	1	0	0	0	0	0	0	5	5	0	S	0.00199800199800199
SE_LT2_02160_p.Asn261Ser	SE_LT2_02160	aldehyde dehydrogenase EutE	0	0	0	1	1	1	1	1	1	0	0	0	0	0	0	6	6	0	C	0.0001998001998002
SE_LT2_02160_p.Thr3Asn	SE_LT2_02160	aldehyde dehydrogenase EutE	0	0	0	1	1	1	1	1	1	0	0	0	0	0	0	6	6	0	C	0.0001998001998002
SE_LT2_02162_p.Pro244Leu	SE_LT2_02162	NADH dehydrogenase	0	0	0	1	1	1	1	1	1	0	0	0	0	0	0	6	6	0	C	0.0001998001998002
SE_LT2_02163_p.Asp30Asn	SE_LT2_02163	propanediol utilization protein	0	0	0	1	1	1	1	1	1	0	0	0	0	0	0	6	6	0	CQ	0.0001998001998002
SE_LT2_02164_p.Arg100His	SE_LT2_02164	propanediol utilization protein	0	0	0	0	1	1	1	1	1	0	0	0	0	0	0	5	5	0	E	0.00199800199800199
SE_LT2_02166_p.Ala39Thr	SE_LT2_02166	acetate kinase	0	0	0	1	1	1	1	1	1	0	0	0	0	0	0	6	6	0	H	0.0001998001998002
SE_LT2_02167_p.Arg180Cys	SE_LT2_02167	GHMP kinase	0	0	0	1	1	1	1	1	1	0	0	0	0	0	0	6	6	0	Q	0.0001998001998002
SE_LT2_02168_p.Asp10Asn	SE_LT2_02168	hypothetical protein	0	0	0	1	1	1	1	1	1	0	0	0	0	0	0	6	6	0	S	0.0001998001998002
SE_LT2_02170_p.Lys155Gln	SE_LT2_02170	DNA gyrase inhibitor	0	0	0	1	1	1	1	1	1	0	0	0	0	0	0	6	6	0	L	0.0001998001998002
SE_LT2_02171_p.Gly375Ser	SE_LT2_02171	serine-type D-Ala-D-Ala carboxypep	0	0	0	1	1	1	1	1	1	0	0	0	0	0	0	6	6	0	M	0.0001998001998002
SE_LT2_02173_p.Arg169Leu	SE_LT2_02173	thiosulfate reductase electron trans	0	0	0	1	1	1	1	1	1	0	0	0	0	0	0	6	6	0	C	0.0001998001998002
SE_LT2_02176_p.Ala423Thr	SE_LT2_02176	exodeoxyribonuclease I	0	0	0	1	1	1	1	1	1	0	0	0	0	0	0	6	6	0	L	0.0001998001998002
SE_LT2_02179_p.Leu229Met	SE_LT2_02179	putative LysR family transcriptional	0	0	0	1	1	1	1	1	1	0	0	0	0	0	0	6	6	0	K	0.0001998001998002
SE_LT2_02211_p.Ala254Val	SE_LT2_02211	colanic acid biosynthesis protein Wc	0	0	0	1	1	1	1	1	1	0	0	0	0	0	0	6	6	0	S	0.0001998001998002
SE_LT2_02212_p.Pro284Ser	SE_LT2_02212	colanic acid biosynthesis glycosyltra	0	0	0	1	1	1	1	1	1	0	0	0	0	0	0	6	6	0	M	0.0001998001998002
SE_LT2_02213_p.Ala425Glu	SE_LT2_02213	colanic acid biosynthesis pyruvyl tra	0	0	0	1	1	1	1	1	1	0	0	0	0	0	0	6	6	0	S	0.0001998001998002
SE_LT2_02215_p.Ala218Ser	SE_LT2_02215	undecaprenyl-phosphate glucose ph	0	0	0	1	1	1	1	1	1	0	0	0	0	0	0	6	6	0	M	0.0001998001998002
SE_LT2_02219_p.Ala165Val	SE_LT2_02219	putative glycosyl transferase	0	0	0	1	1	1	1	1	1	0	0	0	0	0	0	6	6	0	G	0.0001998001998002
SE_LT2_02219_p.Thr382Met	SE_LT2_02219	putative glycosyl transferase	0	0	0	1	1	1	1	1	1	0	0	0	0	0	0	6	6	0	G	0.0001998001998002
SE_LT2_02220_p.Cys53Arg	SE_LT2_02220	GDP-mannose mannosyl hydrolase	0	0	0	1	1	1	1	1	1	0	0	0	0	0	0	6	6	0	F	0.0001998001998002
SE_LT2_02222_p.Gln76Arg	SE_LT2_02222	GDP-mannose 4,6-dehydratase	0	0	0	1	1	1	1	1	1	0	0	0	0	0	0	6	6	0	H	0.0001998001998002
SE_LT2_02229_p.Thr185Lys	SE_LT2_02229	putative tyrosine-protein kinase	0	0	0	1	1	1	1	1	1	0	0	0	0	0	0	6	6	0	DM	0.0001998001998002
SE_LT2_02238_p.Cys74Tyr	SE_LT2_02238	DNA-3-methyladenine glycosylase 2	0	0	0	1	1	1	1	1	1	0	0	0	0	0	0	6	6	0	G	0.0001998001998002
SE_LT2_02238_p.Pro200Ser	SE_LT2_02238	DNA-3-methyladenine glycosylase 2	0	0	0	1	1	1	1	1	1	0	0	0	0	0	0	6	6	0	G	0.0001998001998002
SE_LT2_02241_p.Glu52Lys	SE_LT2_02241	hypothetical protein	0	0	0	1	1	1	1	1	1	0	0	0	0	0	0	6	6	0	-	0.0001998001998002
SE_LT2_02243_p.Thr986Asn	SE_LT2_02243	multidrug transporter subunit MdtB	0	0	0	1	1	1	1	1	1	0	0	0	0	0	0	6	6	0	U	0.0001998001998002
SE_LT2_02243_p.Val3Gly	SE_LT2_02243	multidrug transporter subunit MdtB	0	0	0	1	1	1	1	1	1	0	0	0	0	0	0	6	6	0	U	0.0001998001998002
SE_LT2_02246_p.Met98Ile	SE_LT2_02246	two-component system sensor histi	0	0	0	1	1	1	1	1	1	0	0	0	0	0	0	6	6	0	T	0.0001998001998002
SE_LT2_02248_p.His256Tyr	SE_LT2_02248	hypothetical protein	0	0	0	1	1	1	1	1	1	0	0	0	0	0	0	6	6	0	S	0.0001998001998002
SE_LT2_02248_p.Lys373Glu	SE_LT2_02248	hypothetical protein	0	0	0	1	1	1	1	1	1	0	0	0	0	0	0	6	6	0	S	0.0001998001998002
SE_LT2_02249_p.Gln162Leu	SE_LT2_02249	hypothetical protein	0	0	0	1	1	1	1	1	1	0	0	0	0	0	0	6	6	0	S	0.0001998001998002
SE_LT2_02250_p.Glu178Ala	SE_LT2_02250	hypothetical protein	0	0	0	1	1	1	1	1	1	0	0	0	0	0	0	6	6	0	S	0.0001998001998002
SE_LT2_02250_p.Lys646Arg	SE_LT2_02250	hypothetical protein	0	0	0	1	1	1	1	1	1	0	0	0	0	0	0	6	6	0	S	0.0001998001998002
SE_LT2_02256_p.Arg55Lys	SE_LT2_02256	lipid kinase YegS	0	0	0	1	1	1	1	1	1	0	0	0	0	0	0	6	6	0	F	0.0001998001998002
SE_LT2_02258_p.Ala92Val	SE_LT2_02258	MFS transporter	0	0	0	1	1	1	1	1	1	0	0	0	0	0	0	6	6	0	G	0.0001998001998002

SE_LT2_02258_p.Ile280Val	SE_LT2_02258	MFS transporter	0	0	0	1	1	1	1	1	1	0	0	0	0	0	0	6	6	0	G	0.0001998001998002
SE_LT2_02258_p.Phe84Leu	SE_LT2_02258	MFS transporter	0	0	0	1	1	1	1	1	1	0	0	0	0	0	0	6	6	0	G	0.0001998001998002
SE_LT2_02259_p.Asn79Ser	SE_LT2_02259	putative ADP-ribosylglycohydrolase	0	0	0	1	1	1	1	1	1	0	0	0	0	0	0	6	6	0	O	0.0001998001998002
SE_LT2_02259_p.His228Arg	SE_LT2_02259	putative ADP-ribosylglycohydrolase	0	0	0	0	1	1	1	1	1	0	0	0	0	0	0	5	5	0	O	0.00199800199800199
SE_LT2_02259_p.Thr188Ala	SE_LT2_02259	putative ADP-ribosylglycohydrolase	0	0	0	1	1	1	1	1	1	0	0	0	0	0	0	6	6	0	O	0.0001998001998002
SE_LT2_02260_p.Pro233Ser	SE_LT2_02260	putative sugar kinase	0	0	0	1	1	1	1	1	1	0	0	0	0	0	0	6	6	0	G	0.0001998001998002
SE_LT2_02261_p.Ala19Ser	SE_LT2_02261	GntR family transcriptional regulato	0	0	0	1	1	1	1	1	1	0	0	0	0	0	0	6	6	0	K	0.0001998001998002
SE_LT2_02274_p.Glu107Lys	SE_LT2_02274	hypothetical protein	0	0	0	1	1	1	1	1	1	0	0	0	0	0	0	6	6	0	S	0.0001998001998002
SE_LT2_02274_p.Glu51Lys	SE_LT2_02274	hypothetical protein	0	0	0	1	1	1	1	1	1	0	0	0	0	0	0	6	6	0	S	0.0001998001998002
SE_LT2_02274_p.Gly32Glu	SE_LT2_02274	hypothetical protein	0	0	0	1	1	1	1	1	1	0	0	0	0	0	0	6	6	0	S	0.0001998001998002
SE_LT2_02275_p.Arg51Ser	SE_LT2_02275	hypothetical protein	0	0	0	1	1	1	1	1	1	0	0	0	0	0	0	6	6	0	S	0.0001998001998002
SE_LT2_02275_p.Asp31Asn	SE_LT2_02275	hypothetical protein	0	0	0	1	1	1	1	1	1	0	0	0	0	0	0	6	6	0	S	0.0001998001998002
SE_LT2_02279_p.Val3Ile	SE_LT2_02279	hypothetical protein	0	0	0	1	1	1	1	1	1	0	0	0	0	0	0	6	6	0	-	0.0001998001998002
SE_LT2_02284_p.Asn524Asp	SE_LT2_02284	beta-glucosidase	0	0	0	1	1	1	1	1	1	0	0	0	0	0	0	6	6	0	G	0.0001998001998002
SE_LT2_02284_p.Ile711Leu	SE_LT2_02284	beta-glucosidase	0	0	0	1	1	1	1	1	1	0	0	0	0	0	0	6	6	0	G	0.0001998001998002
SE_LT2_02293_p.Cys88Arg	SE_LT2_02293	salicylate hydroxylase	0	0	0	1	1	1	1	1	1	0	0	0	0	0	0	6	6	0	CH	0.0001998001998002
SE_LT2_02296_p.Arg209Cys	SE_LT2_02296	gentisate 1,2-dioxygenase	0	0	0	0	1	1	1	1	1	0	0	0	0	0	0	5	5	0	Q	0.00199800199800199
SE_LT2_02308_p.Ala301Val	SE_LT2_02308	methyl-galactoside ABC transporter	0	0	0	1	1	1	1	1	1	0	0	0	0	0	0	6	6	0	G	0.0001998001998002
SE_LT2_02314_p.Ala189Gly	SE_LT2_02314	L-serine ammonia-lyase	0	0	0	1	1	1	1	1	1	0	0	0	0	0	0	6	6	0	E	0.0001998001998002
SE_LT2_02314_p.Pro308Ser	SE_LT2_02314	L-serine ammonia-lyase	0	0	0	1	1	1	1	1	1	0	0	0	0	0	0	6	6	0	E	0.0001998001998002
SE_LT2_02315_p.Ala40Thr	SE_LT2_02315	phosphoserine phosphatase SerB	0	0	0	1	1	1	1	1	1	0	0	0	0	0	0	6	6	0	E	0.0001998001998002
SE_LT2_02315_p.Asp36Asn	SE_LT2_02315	phosphoserine phosphatase SerB	0	0	0	1	1	1	1	1	1	0	0	0	0	0	0	6	6	0	E	0.0001998001998002
SE_LT2_02316_p.Phe31Ile	SE_LT2_02316	MFS transporter	0	0	0	1	1	1	1	1	1	0	0	0	0	0	0	6	6	0	G	0.0001998001998002
SE_LT2_02317_p.Thr183Ile	SE_LT2_02317	catecholate siderophore receptor Ci	0	0	0	1	1	1	1	1	1	0	0	0	0	0	0	6	6	0	P	0.0001998001998002
SE_LT2_02319_p.Thr250Ser	SE_LT2_02319	putative LysR family transcriptional	0	0	0	1	1	1	1	1	1	0	0	0	0	0	0	6	6	0	K	0.0001998001998002
SE_LT2_02322_p.AlaVal94ValAla	SE_LT2_02322	PTS fructose transporter subunit IIB	0	0	0	1	0	1	1	1	1	0	0	0	0	0	0	5	5	0	G	0.00199800199800199
SE_LT2_02323_p.Ala179Ser	SE_LT2_02323	1-phosphofructokinase	0	0	0	1	1	1	1	1	1	0	0	0	0	0	0	6	6	0	H	0.0001998001998002
SE_LT2_02340_p.Gln56Leu	SE_LT2_02340	16S rRNA pseudouridine(516) syntha	0	0	0	1	1	1	1	1	1	0	0	0	0	0	0	6	6	0	J	0.0001998001998002
SE_LT2_02344_p.Ser332Leu	SE_LT2_02344	nucleoid-associated protein YejK	0	0	0	1	1	1	1	1	1	0	0	0	0	0	0	6	6	0	S	0.0001998001998002
SE_LT2_02346_p.Ala425Thr	SE_LT2_02346	Inner membrane protein YejM	0	0	0	1	1	1	1	1	1	0	0	0	0	0	0	6	6	0	S	0.0001998001998002
SE_LT2_02360_p.Thr615Met	SE_LT2_02360	E3 ubiquitin--protein ligase	0	0	0	1	1	1	1	1	1	0	0	0	0	0	0	6	6	0	S	0.0001998001998002
SE_LT2_02385_p.Asp219Glu	SE_LT2_02385	bifunctional transcriptional regulato	0	0	0	1	1	1	1	1	1	0	0	0	0	0	0	6	6	0	F	0.0001998001998002
SE_LT2_02385_p.Leu240Pro	SE_LT2_02385	bifunctional transcriptional regulato	0	0	0	1	1	1	1	1	1	0	0	0	0	0	0	6	6	0	F	0.0001998001998002
SE_LT2_02386_p.Glu37Lys	SE_LT2_02386	FAD:protein FMN transferase ApbE	0	0	0	1	1	1	1	1	1	0	0	0	0	0	0	6	6	0	H	0.0001998001998002
SE_LT2_02386_p.Leu345Met	SE_LT2_02386	FAD:protein FMN transferase ApbE	0	0	0	1	1	1	1	1	1	0	0	0	0	0	0	6	6	0	H	0.0001998001998002
SE_LT2_02389_p.Ser337Thr	SE_LT2_02389	phosphotransferase RcsD	0	0	0	1	1	1	1	1	1	0	0	0	0	0	0	6	6	0	T	0.0001998001998002
SE_LT2_02392_p.Ser83Phe	SE_LT2_02392	DNA gyrase subunit A	0	0	0	0	1	1	1	1	1	0	0	0	0	0	0	5	5	0	L	0.00199800199800199
SE_LT2_02394_p.Met337Val	SE_LT2_02394	MFS transporter	0	0	0	1	1	1	1	1	1	0	0	0	0	0	0	6	6	0	G	0.0001998001998002
SE_LT2_02397_p.HisAsp384AsnG	SE_LT2_02397	ribonucleoside-diphosphate reducta	0	0	0	1	1	1	1	1	1	0	0	0	0	0	0	6	6	0	F	0.0001998001998002
SE_LT2_02401_p.Thr246Ala	SE_LT2_02401	transcriptional regulator	0	0	0	1	1	1	1	1	1	0	0	0	0	0	0	6	6	0	K	0.0001998001998002
SE_LT2_02406_p.Gly190Asp	SE_LT2_02406	sn-glycerol-3-phosphate dehydroge	0	0	0	1	1	1	1	1	1	0	0	0	0	0	0	6	6	0	C	0.0001998001998002
SE_LT2_02408_p.Lys260Thr	SE_LT2_02408	putative cytoplasmic protein	0	0	0	1	1	1	1	1	1	0	0	0	0	0	0	6	6	0	O	0.0001998001998002
SE_LT2_02408_p.SerAspGluValA	SE_LT2_02408	putative cytoplasmic protein	0	0	0	1	1	1	1	1	1	0	0	0	0	0	0	6	6	0	O	0.0001998001998002
SE_LT2_02411_p.Ala80Val	SE_LT2_02411	MFS transporter	0	0	0	1	1	1	1	1	1	0	0	0	0	0	0	6	6	0	G	0.0001998001998002
SE_LT2_02412_p.Asp361Ala	SE_LT2_02412	putative galactonate dehydratase	0	0	0	1	1	1	1	1	1	0	0	0	0	0	0	6	6	0	M	0.0001998001998002
SE_LT2_02437_p.Asn99Asp	SE_LT2_02437	NADH dehydrogenase I chain N	0	0	0	1	1	1	1	1	1	0	0	0	0	0	0	6	6	0	C	0.0001998001998002
SE_LT2_02437_p.Gln102Arg	SE_LT2_02437	NADH dehydrogenase I chain N	0	0	0	1	1	1	1	1	1	0	0	0	0	0	0	6	6	0	C	0.0001998001998002
SE_LT2_02444_p.Val315Ile	SE_LT2_02444	NADH-quinone oxidoreductase subu	0	0	0	1	1	1	1	1	1	0	0	0	0	0	0	6	6	0	C	0.0001998001998002
SE_LT2_02446_p.Val4Ile	SE_LT2_02446	NADH-quinone oxidoreductase subu	0	0	0	1	1	1	1	1	1	0	0	0	0	0	0	6	6	0	C	0.0001998001998002
SE_LT2_02453_p.Asp110Asn	SE_LT2_02453	5'-deoxynucleotidase	0	0	0	1	1	1	1	1	1	0	0	0	0	0	0	6	6	0	F	0.0001998001998002

SE_LT2_02456_p.Val143Ile	SE_LT2_02456	hypothetical protein	0	0	0	1	1	1	1	1	1	0	0	0	0	0	0	6	6	0	S	0.0001998001998002
SE_LT2_02465_p.Val126Ala	SE_LT2_02465	PTS ascorbate transporter subunit II	0	0	0	1	1	1	1	1	1	0	0	0	0	0	0	6	6	0	G	0.0001998001998002
SE_LT2_02468_p.Ser174Ile	SE_LT2_02468	YfcE family phosphodiesterase	0	0	0	1	1	1	1	1	1	0	0	0	0	0	0	6	6	0	S	0.0001998001998002
SE_LT2_02481_p.Ala375Val	SE_LT2_02481	putative diaminopimelate decarbox	0	0	0	1	0	1	1	1	1	0	0	0	0	0	0	5	5	0	E	0.00199800199800199
SE_LT2_02481_p.Lys406Arg	SE_LT2_02481	putative diaminopimelate decarbox	0	0	0	1	1	1	1	1	1	0	0	0	0	0	0	6	6	0	E	0.0001998001998002
SE_LT2_02486_p.Ala114Thr	SE_LT2_02486	bifunctional tetrahydrofolate synth	0	0	0	1	1	1	1	1	1	0	0	0	0	0	0	6	6	0	H	0.0001998001998002
SE_LT2_02486_p.Thr232Ala	SE_LT2_02486	bifunctional tetrahydrofolate synth	0	0	0	1	1	1	1	1	1	0	0	0	0	0	0	6	6	0	H	0.0001998001998002
SE_LT2_02491_p.Thr288Ile	SE_LT2_02491	4-phosphoerythronate dehydrogena	0	0	0	1	1	1	1	1	1	0	0	0	0	0	0	6	6	0	H	0.0001998001998002
SE_LT2_02498_p.Gly41Ala	SE_LT2_02498	hypothetical protein	0	0	0	1	1	1	1	1	1	0	0	0	0	0	0	6	6	0	S	0.0001998001998002
SE_LT2_02506_p.Asn247Ser	SE_LT2_02506	ribosomal protein L3 N(5)-glutamine	0	0	0	1	1	1	1	1	1	0	0	0	0	0	0	6	6	0	J	0.0001998001998002
SE_LT2_02518_p.Gly206Arg	SE_LT2_02518	transcriptional regulator	0	0	0	1	1	1	1	1	1	0	0	0	0	0	0	6	6	0	T	0.0001998001998002
SE_LT2_02519_p.Thr24Met	SE_LT2_02519	sensor histidine kinase	0	0	0	1	1	1	1	1	1	0	0	0	0	0	0	6	6	0	T	0.0001998001998002
SE_LT2_02528_p.Ala158Val	SE_LT2_02528	indolepyruvate decarboxylase	0	0	0	1	1	1	1	1	1	0	0	0	0	0	0	6	6	0	GH	0.0001998001998002
SE_LT2_02528_p.Ala293Val	SE_LT2_02528	indolepyruvate decarboxylase	0	0	0	1	1	1	1	1	1	0	0	0	0	0	0	6	6	0	GH	0.0001998001998002
SE_LT2_02528_p.Glu398Lys	SE_LT2_02528	indolepyruvate decarboxylase	0	0	0	1	1	1	1	1	1	0	0	0	0	0	0	6	6	0	GH	0.0001998001998002
SE_LT2_02546_p.Arg92Cys	SE_LT2_02546	purine-nucleoside phosphorylase	0	0	0	1	1	1	1	1	1	0	0	0	0	0	0	6	6	0	F	0.0001998001998002
SE_LT2_02551_p.Gly480Val	SE_LT2_02551	DNA ligase (NAD(+)) LigA	0	0	0	1	1	1	1	1	1	0	0	0	0	0	0	6	6	0	L	0.0001998001998002
SE_LT2_02560_p.Arg351Cys	SE_LT2_02560	transcriptional regulator PtsJ	0	0	0	1	1	1	1	1	1	0	0	0	0	0	0	6	6	0	K	0.0001998001998002
SE_LT2_02560_p.Ile292Val	SE_LT2_02560	transcriptional regulator PtsJ	0	0	0	1	0	1	1	1	1	0	0	0	0	0	0	5	5	0	K	0.00199800199800199
SE_LT2_02560_p.Ile424Val	SE_LT2_02560	transcriptional regulator PtsJ	0	0	0	1	1	1	1	1	1	0	0	0	0	0	0	6	6	0	K	0.0001998001998002
SE_LT2_02560_p.Tyr342Cys	SE_LT2_02560	transcriptional regulator PtsJ	0	0	0	1	1	1	1	1	1	0	0	0	0	0	0	6	6	0	K	0.0001998001998002
SE_LT2_02561_p.Cys65Ser	SE_LT2_02561	putative GMP synthase	0	0	0	1	1	1	1	1	1	0	0	0	0	0	0	6	6	0	F	0.0001998001998002
SE_LT2_02561_p.His236Gln	SE_LT2_02561	putative GMP synthase	0	0	0	1	1	1	1	1	1	0	0	0	0	0	0	6	6	0	F	0.0001998001998002
SE_LT2_02564_p.Ile274Val	SE_LT2_02564	cysteine synthase B	0	0	0	1	1	1	1	1	1	0	0	0	0	0	0	6	6	0	E	0.0001998001998002
SE_LT2_02567_p.Ile27Thr	SE_LT2_02567	sulfate ABC transporter permease su	0	0	0	1	1	1	1	1	1	0	0	0	0	0	0	6	6	0	P	0.0001998001998002
SE_LT2_02568_p.Pro10Leu	SE_LT2_02568	thiosulfate transporter subunit	0	0	0	1	1	1	1	1	1	0	0	0	0	0	0	6	6	0	P	0.0001998001998002
SE_LT2_02568_p.Ser314Thr	SE_LT2_02568	thiosulfate transporter subunit	0	0	0	1	1	1	1	1	1	0	0	0	0	0	0	6	6	0	P	0.0001998001998002
SE_LT2_02572_p.AspAlaVal82Glu	SE_LT2_02572	Inner membrane protein YfeZ	0	0	0	1	1	1	1	1	1	0	0	0	0	0	0	6	6	0	S	0.0001998001998002
SE_LT2_02585_p.Ala106Val	SE_LT2_02585	alcohol dehydrogenase EutG	0	0	0	0	1	1	1	1	1	0	0	0	0	0	0	5	5	0	C	0.00199800199800199
SE_LT2_02586_p.His3Tyr	SE_LT2_02586	ethanolamine utilization protein Eu	0	0	0	1	0	1	1	1	1	0	0	0	0	0	0	5	5	0	E	0.00199800199800199
SE_LT2_02590_p.Leu224Phe	SE_LT2_02590	phosphate acetyltransferase	0	0	0	1	1	1	1	1	1	0	0	0	0	0	0	6	6	0	C	0.0001998001998002
SE_LT2_02601_p.Ile309Val	SE_LT2_02601	hypothetical protein	0	0	0	1	1	1	1	1	1	0	0	0	0	0	0	6	6	0	S	0.0001998001998002
SE_LT2_02602_p.Thr24Ile	SE_LT2_02602	GDP-mannose pyrophosphatase Nu	0	0	0	1	1	1	1	1	1	0	0	0	0	0	0	6	6	0	L	0.0001998001998002
SE_LT2_02604_p.Asp319Asn	SE_LT2_02604	oxidoreductase FeS-binding subunit	0	0	0	1	1	1	1	1	1	0	0	0	0	0	0	6	6	0	C	0.0001998001998002
SE_LT2_02611_p.Ala342Val	SE_LT2_02611	tRNA cytosine(34) acetyltransferase	0	0	0	1	0	1	1	1	1	0	0	0	0	0	0	5	5	0	J	0.00199800199800199
SE_LT2_02611_p.Ala42Val	SE_LT2_02611	tRNA cytosine(34) acetyltransferase	0	0	0	1	1	1	1	1	1	0	0	0	0	0	0	6	6	0	J	0.0001998001998002
SE_LT2_02611_p.Gln8Arg	SE_LT2_02611	tRNA cytosine(34) acetyltransferase	0	0	0	1	0	1	1	1	1	0	0	0	0	0	0	5	5	0	J	0.00199800199800199
SE_LT2_02611_p.Phe158Leu	SE_LT2_02611	tRNA cytosine(34) acetyltransferase	0	0	0	1	0	1	1	1	1	0	0	0	0	0	0	5	5	0	J	0.00199800199800199
SE_LT2_02613_p.Val15Leu	SE_LT2_02613	phosphoribosylaminoimidazolesucc	0	0	0	1	1	1	1	1	1	0	0	0	0	0	0	6	6	0	F	0.0001998001998002
SE_LT2_02633_p.Asn170His	SE_LT2_02633	glutamine-hydrolyzing GMP synthas	0	0	0	1	1	1	1	1	1	0	0	0	0	0	0	6	6	0	F	0.0001998001998002
SE_LT2_02633_p.Ser162Thr	SE_LT2_02633	glutamine-hydrolyzing GMP synthas	0	0	0	1	1	1	1	1	1	0	0	0	0	0	0	6	6	0	F	0.0001998001998002
SE_LT2_02633_p.SerAsp152GluG	SE_LT2_02633	glutamine-hydrolyzing GMP synthas	0	0	0	0	1	1	1	1	1	0	0	0	0	0	0	5	5	0	F	0.00199800199800199
SE_LT2_02635_p.Ala224Thr	SE_LT2_02635	exodeoxyribonuclease VII large sub	0	0	0	1	1	1	1	1	1	0	0	0	0	0	0	6	6	0	L	0.0001998001998002
SE_LT2_02636_p.Ala423Thr	SE_LT2_02636	hypothetical protein	0	0	0	1	1	1	1	1	1	0	0	0	0	0	0	6	6	0	MU	0.0001998001998002
SE_LT2_02636_p.Ala91Lys	SE_LT2_02636	hypothetical protein	0	0	0	1	1	1	1	1	1	0	0	0	0	0	0	6	6	0	MU	0.0001998001998002
SE_LT2_02636_p.AlaLys162ThrGl	SE_LT2_02636	hypothetical protein	0	0	0	1	1	1	1	1	1	0	0	0	0	0	0	6	6	0	MU	0.0001998001998002
SE_LT2_02636_p.Arg330Asn	SE_LT2_02636	hypothetical protein	0	0	0	1	1	1	1	1	1	0	0	0	0	0	0	6	6	0	MU	0.0001998001998002
SE_LT2_02636_p.Arg349fs	SE_LT2_02636	hypothetical protein	0	0	0	1	1	1	1	1	1	0	0	0	0	0	0	6	6	0	MU	0.0001998001998002
SE_LT2_02636_p.Asn147Lys	SE_LT2_02636	hypothetical protein	0	0	0	1	1	1	1	1	1	0	0	0	0	0	0	6	6	0	MU	0.0001998001998002
SE_LT2_02636_p.Asn250Thr	SE_LT2_02636	hypothetical protein	0	0	0	1	1	1	1	1	1	0	0	0	0	0	0	6	6	0	MU	0.0001998001998002

SE_LT2_02636_p.Asn489Lys	SE_LT2_02636	hypothetical protein	0	0	0	1	1	1	1	1	1	0	0	0	0	0	0	6	6	0	MU	0.0001998001998002
SE_LT2_02636_p.Asp1503Thr	SE_LT2_02636	hypothetical protein	0	0	0	1	1	1	1	1	1	0	0	0	0	0	0	6	6	0	MU	0.0001998001998002
SE_LT2_02636_p.Asp1721_Gly17	SE_LT2_02636	hypothetical protein	0	0	0	1	0	1	1	1	1	0	0	0	0	0	0	5	5	0	MU	0.00199800199800199
SE_LT2_02636_p.AspAsp137Asn/	SE_LT2_02636	hypothetical protein	0	0	0	1	1	1	1	1	1	0	0	0	0	0	0	6	6	0	MU	0.0001998001998002
SE_LT2_02636_p.AspGly209SerA	SE_LT2_02636	hypothetical protein	0	0	0	1	0	1	1	1	1	0	0	0	0	0	0	5	5	0	MU	0.00199800199800199
SE_LT2_02636_p.Glu205Ala	SE_LT2_02636	hypothetical protein	0	0	0	1	1	1	1	1	1	0	0	0	0	0	0	6	6	0	MU	0.0001998001998002
SE_LT2_02636_p.Glu255Ala	SE_LT2_02636	hypothetical protein	0	0	0	1	1	1	1	1	1	0	0	0	0	0	0	6	6	0	MU	0.0001998001998002
SE_LT2_02636_p.Gly1520Asp	SE_LT2_02636	hypothetical protein	0	0	0	1	1	1	1	1	1	0	0	0	0	0	0	6	6	0	MU	0.0001998001998002
SE_LT2_02636_p.Gly433Lys	SE_LT2_02636	hypothetical protein	0	0	0	1	1	1	1	1	1	0	0	0	0	0	0	6	6	0	MU	0.0001998001998002
SE_LT2_02636_p.Gly475Ser	SE_LT2_02636	hypothetical protein	0	0	0	1	1	1	1	1	1	0	0	0	0	0	0	6	6	0	MU	0.0001998001998002
SE_LT2_02636_p.Ile1255Leu	SE_LT2_02636	hypothetical protein	0	0	0	1	0	1	1	1	1	0	0	0	0	0	0	5	5	0	MU	0.00199800199800199
SE_LT2_02636_p.Leu151Ile	SE_LT2_02636	hypothetical protein	0	0	0	1	1	1	1	1	1	0	0	0	0	0	0	6	6	0	MU	0.0001998001998002
SE_LT2_02636_p.Lys1534Gln	SE_LT2_02636	hypothetical protein	0	0	0	1	1	1	1	1	1	0	0	0	0	0	0	6	6	0	MU	0.0001998001998002
SE_LT2_02636_p.LysThrMet411A	SE_LT2_02636	hypothetical protein	0	0	0	1	1	1	1	1	1	0	0	0	0	0	0	6	6	0	MU	0.0001998001998002
SE_LT2_02636_p.Met1565Ile	SE_LT2_02636	hypothetical protein	0	0	0	1	1	1	1	1	1	0	0	0	0	0	0	6	6	0	MU	0.0001998001998002
SE_LT2_02636_p.MetPheThr443I	SE_LT2_02636	hypothetical protein	0	0	0	1	1	1	1	1	1	0	0	0	0	0	0	6	6	0	MU	0.0001998001998002
SE_LT2_02636_p.Pro95Ala	SE_LT2_02636	hypothetical protein	0	0	0	1	1	1	1	1	1	0	0	0	0	0	0	6	6	0	MU	0.0001998001998002
SE_LT2_02636_p.Ser1501Thr	SE_LT2_02636	hypothetical protein	0	0	0	1	1	1	1	1	1	0	0	0	0	0	0	6	6	0	MU	0.0001998001998002
SE_LT2_02636_p.Ser267Ile	SE_LT2_02636	hypothetical protein	0	0	0	1	1	1	1	1	1	0	0	0	0	0	0	6	6	0	MU	0.0001998001998002
SE_LT2_02636_p.Ser363Thr	SE_LT2_02636	hypothetical protein	0	0	0	1	1	1	1	1	1	0	0	0	0	0	0	6	6	0	MU	0.0001998001998002
SE_LT2_02636_p.Ser439Arg	SE_LT2_02636	hypothetical protein	0	0	0	1	1	1	1	1	1	0	0	0	0	0	0	6	6	0	MU	0.0001998001998002
SE_LT2_02636_p.Thr123Lys	SE_LT2_02636	hypothetical protein	0	0	0	1	1	1	1	1	1	0	0	0	0	0	0	6	6	0	MU	0.0001998001998002
SE_LT2_02636_p.Thr190Asn	SE_LT2_02636	hypothetical protein	0	0	0	1	1	1	1	1	1	0	0	0	0	0	0	6	6	0	MU	0.0001998001998002
SE_LT2_02636_p.Thr236Lys	SE_LT2_02636	hypothetical protein	0	0	0	1	1	1	1	1	1	0	0	0	0	0	0	6	6	0	MU	0.0001998001998002
SE_LT2_02636_p.Thr253Ala	SE_LT2_02636	hypothetical protein	0	0	0	1	1	1	1	1	1	0	0	0	0	0	0	6	6	0	MU	0.0001998001998002
SE_LT2_02636_p.Thr293Asn	SE_LT2_02636	hypothetical protein	0	0	0	1	1	1	1	1	1	0	0	0	0	0	0	6	6	0	MU	0.0001998001998002
SE_LT2_02636_p.Thr311Ser	SE_LT2_02636	hypothetical protein	0	0	0	1	1	1	1	1	1	0	0	0	0	0	0	6	6	0	MU	0.0001998001998002
SE_LT2_02636_p.Thr406Ala	SE_LT2_02636	hypothetical protein	0	0	0	1	1	1	1	1	1	0	0	0	0	0	0	6	6	0	MU	0.0001998001998002
SE_LT2_02636_p.ThrAspGlu101V	SE_LT2_02636	hypothetical protein	0	0	0	1	0	1	1	1	1	0	0	0	0	0	0	5	5	0	MU	0.00199800199800199
SE_LT2_02636_p.ThrGln171SerLe	SE_LT2_02636	hypothetical protein	0	0	0	1	1	1	1	1	1	0	0	0	0	0	0	6	6	0	MU	0.0001998001998002
SE_LT2_02636_p.ThrGly167AspS	SE_LT2_02636	hypothetical protein	0	0	0	1	1	1	1	1	1	0	0	0	0	0	0	6	6	0	MU	0.0001998001998002
SE_LT2_02636_p.Val1818Leu	SE_LT2_02636	hypothetical protein	0	0	0	1	1	1	1	1	1	0	0	0	0	0	0	6	6	0	MU	0.0001998001998002
SE_LT2_02638_p.Ala1756fs	SE_LT2_02638	hypothetical protein	0	0	0	1	1	1	1	1	1	0	0	0	0	0	0	6	6	0	M	0.0001998001998002
SE_LT2_02638_p.Asn1666Thr	SE_LT2_02638	hypothetical protein	0	0	0	1	0	1	1	1	1	0	0	0	0	0	0	5	5	0	M	0.00199800199800199
SE_LT2_02638_p.Asn1828Asp	SE_LT2_02638	hypothetical protein	0	0	0	1	1	1	1	1	1	0	0	0	0	0	0	6	6	0	M	0.0001998001998002
SE_LT2_02638_p.Asp1617Glu	SE_LT2_02638	hypothetical protein	0	0	0	1	1	1	1	1	1	0	0	0	0	0	0	6	6	0	M	0.0001998001998002
SE_LT2_02638_p.Asp1759fs	SE_LT2_02638	hypothetical protein	0	0	0	1	1	1	1	1	1	0	0	0	0	0	0	6	6	0	M	0.0001998001998002
SE_LT2_02638_p.Asp594Glu	SE_LT2_02638	hypothetical protein	0	0	0	1	1	1	1	1	1	0	0	0	0	0	0	6	6	0	M	0.0001998001998002
SE_LT2_02638_p.Asp764Ser	SE_LT2_02638	hypothetical protein	0	0	0	0	1	1	1	1	1	0	0	0	0	0	0	5	5	0	M	0.00199800199800199
SE_LT2_02638_p.AspLys1689Asn	SE_LT2_02638	hypothetical protein	0	0	0	1	0	1	1	1	1	0	0	0	0	0	0	5	5	0	M	0.00199800199800199
SE_LT2_02638_p.Glu1777Gln	SE_LT2_02638	hypothetical protein	0	0	0	1	1	1	1	1	1	0	0	0	0	0	0	6	6	0	M	0.0001998001998002
SE_LT2_02638_p.GluLysAlaThrTh	SE_LT2_02638	hypothetical protein	0	0	0	1	1	1	1	1	1	0	0	0	0	0	0	6	6	0	M	0.0001998001998002
SE_LT2_02638_p.Gly1353Ala	SE_LT2_02638	hypothetical protein	0	0	0	1	1	1	1	1	1	0	0	0	0	0	0	6	6	0	M	0.0001998001998002
SE_LT2_02638_p.GlyMet757Asp\	SE_LT2_02638	hypothetical protein	0	0	0	0	1	1	1	1	1	0	0	0	0	0	0	5	5	0	M	0.00199800199800199
SE_LT2_02638_p.Leu1853Phe	SE_LT2_02638	hypothetical protein	0	0	0	1	1	1	1	1	1	0	0	0	0	0	0	6	6	0	M	0.0001998001998002
SE_LT2_02638_p.Phe1763Leu	SE_LT2_02638	hypothetical protein	0	0	0	1	1	1	1	1	1	0	0	0	0	0	0	6	6	0	M	0.0001998001998002
SE_LT2_02638_p.Ser65Thr	SE_LT2_02638	hypothetical protein	0	0	0	1	1	1	1	1	1	0	0	0	0	0	0	6	6	0	M	0.0001998001998002
SE_LT2_02638_p.Thr1770Ala	SE_LT2_02638	hypothetical protein	0	0	0	1	1	1	1	1	1	0	0	0	0	0	0	6	6	0	M	0.0001998001998002
SE_LT2_02638_p.Thr84Ala	SE_LT2_02638	hypothetical protein	0	0	0	1	1	1	1	1	1	0	0	0	0	0	0	6	6	0	M	0.0001998001998002
SE_LT2_02638_p.Thr898Ser	SE_LT2_02638	hypothetical protein	0	0	0	1	1	1	1	1	1	0	0	0	0	0	0	6	6	0	M	0.0001998001998002

SE_LT2_02638_p.Tyr1748Ser	SE_LT2_02638	hypothetical protein	0	0	0	1	1	1	1	1	1	0	0	0	0	0	0	6	6	0	M	0.0001998001998002
SE_LT2_02638_p.Val1137Ala	SE_LT2_02638	hypothetical protein	0	0	0	1	0	1	1	1	1	0	0	0	0	0	0	5	5	0	M	0.00199800199800199
SE_LT2_02638_p.ValAla1349Asn	SE_LT2_02638	hypothetical protein	0	0	0	1	1	1	1	1	1	0	0	0	0	0	0	6	6	0	M	0.0001998001998002
SE_LT2_02639_p.Thr267Ser	SE_LT2_02639	hypothetical protein	0	0	0	1	1	1	1	1	1	0	0	0	0	0	0	6	6	0	-	0.0001998001998002
SE_LT2_02641_p.Leu33Ile	SE_LT2_02641	putative protein YfgJ	0	0	0	1	1	1	1	1	1	0	0	0	0	0	0	6	6	0	S	0.0001998001998002
SE_LT2_02642_p.Ala191Val	SE_LT2_02642	ribosome biogenesis GTPase Der	0	0	0	1	1	1	1	1	1	0	0	0	0	0	0	6	6	0	S	0.0001998001998002
SE_LT2_02652_p.Ser16Ala	SE_LT2_02652	dimethyl sulfoxide reductase	0	0	0	1	1	1	1	1	1	0	0	0	0	0	0	6	6	0	S	0.0001998001998002
SE_LT2_02652_p.Thr150Ser	SE_LT2_02652	dimethyl sulfoxide reductase	0	0	0	1	1	1	1	1	1	0	0	0	0	0	0	6	6	0	S	0.0001998001998002
SE_LT2_02653_p.Arg105Cys	SE_LT2_02653	dimethylsulfoxide reductase subuni	0	0	0	1	1	1	1	1	1	0	0	0	0	0	0	6	6	0	C	0.0001998001998002
SE_LT2_02654_p.Lys711Thr	SE_LT2_02654	putative anaerobic dimethylsulfoxid	0	0	0	1	1	1	1	1	1	0	0	0	0	0	0	6	6	0	C	0.0001998001998002
SE_LT2_02654_p.Thr715Ala	SE_LT2_02654	putative anaerobic dimethylsulfoxid	0	0	0	1	1	1	1	1	1	0	0	0	0	0	0	6	6	0	C	0.0001998001998002
SE_LT2_02654_p.Val720Met	SE_LT2_02654	putative anaerobic dimethylsulfoxid	0	0	0	1	1	1	1	1	1	0	0	0	0	0	0	6	6	0	C	0.0001998001998002
SE_LT2_02655_p.Ala228Glu	SE_LT2_02655	penicillin-binding protein 1C	0	0	0	1	1	1	1	1	1	0	0	0	0	0	0	6	6	0	M	0.0001998001998002
SE_LT2_02656_p.Asn1420Ser	SE_LT2_02656	Alpha-2-macroglobulin	0	0	0	1	1	1	1	1	1	0	0	0	0	0	0	6	6	0	S	0.0001998001998002
SE_LT2_02656_p.Asp1458Gly	SE_LT2_02656	Alpha-2-macroglobulin	0	0	0	1	1	1	1	1	1	0	0	0	0	0	0	6	6	0	S	0.0001998001998002
SE_LT2_02656_p.Phe1085Leu	SE_LT2_02656	Alpha-2-macroglobulin	0	0	0	1	1	1	1	0	1	0	0	0	0	0	0	5	5	0	S	0.00199800199800199
SE_LT2_02656_p.Ser893Arg	SE_LT2_02656	Alpha-2-macroglobulin	0	0	0	1	1	1	1	1	1	0	0	0	0	0	0	6	6	0	S	0.0001998001998002
SE_LT2_02657_p.Tyr42Cys	SE_LT2_02657	3-mercaptopyruvate sulfurtransfera	0	0	0	1	1	1	1	1	1	0	0	0	0	0	0	6	6	0	H	0.0001998001998002
SE_LT2_02657_p.Val154Met	SE_LT2_02657	3-mercaptopyruvate sulfurtransfera	0	0	0	1	1	1	1	1	1	0	0	0	0	0	0	6	6	0	H	0.0001998001998002
SE_LT2_02658_p.Asp35Asn	SE_LT2_02658	hypothetical protein	0	0	0	1	1	1	1	1	1	0	0	0	0	0	0	6	6	0	-	0.0001998001998002
SE_LT2_02666_p.Arg128Cys	SE_LT2_02666	Fe-S protein assembly chaperone Hs	0	0	0	1	1	1	1	1	1	0	0	0	0	0	0	6	6	0	F	0.0001998001998002
SE_LT2_02674_p.Ala145Val	SE_LT2_02674	inositol monophosphatase	0	0	0	1	1	1	1	1	1	0	0	0	0	0	0	6	6	0	G	0.0001998001998002
SE_LT2_02677_p.Thr86Met	SE_LT2_02677	anaerobic sulfite reductase subunit	0	0	0	1	1	1	1	1	1	0	0	0	0	0	0	6	6	0	C	0.0001998001998002
SE_LT2_02680_p.Asp154Ala	SE_LT2_02680	hypothetical protein	0	0	0	1	1	1	1	1	1	0	0	0	0	0	0	6	6	0	S	0.0001998001998002
SE_LT2_02685_p.Gln411His	SE_LT2_02685	transcriptional regulator CadC	0	0	0	1	1	1	1	1	1	0	0	0	0	0	0	6	6	0	K	0.0001998001998002
SE_LT2_02686_p.Ser435_Asp436	SE_LT2_02686	lysine:cadaverine antiporter	0	0	0	1	1	0	1	1	1	0	0	0	0	0	0	5	5	0	E	0.00199800199800199
SE_LT2_02691_p.Gln198Lys	SE_LT2_02691	putative transcriptional regulator of	0	0	0	1	1	1	1	1	1	0	0	0	0	0	0	6	6	0	NU	0.0001998001998002
SE_LT2_02692_p.Pro270Ser	SE_LT2_02692	putative sensory kinase in regulator	0	0	0	1	1	1	1	1	1	0	0	0	0	0	0	6	6	0	T	0.0001998001998002
SE_LT2_02694_p.AsnHis1113Thr	SE_LT2_02694	phosphoribosylformylglycinamidine	0	0	0	1	1	1	1	1	1	0	0	0	0	0	0	6	6	0	F	0.0001998001998002
SE_LT2_02698_p.Thr348Ile	SE_LT2_02698	PTS sugar transporter subunit IIC	0	0	0	1	1	1	1	1	1	0	0	0	0	0	0	6	6	0	G	0.0001998001998002
SE_LT2_02703_p.Asn109Ser	SE_LT2_02703	transcriptional regulator	0	0	0	1	1	1	1	1	1	0	0	0	0	0	0	6	6	0	K	0.0001998001998002
SE_LT2_02783_p.Glu80Lys	SE_LT2_02783	putative LysR family transcriptional	0	0	0	1	1	1	1	1	1	0	0	0	0	0	0	6	6	0	K	0.0001998001998002
SE_LT2_02783_p.Gly89Asp	SE_LT2_02783	putative LysR family transcriptional	0	0	0	1	1	1	1	1	1	0	0	0	0	0	0	6	6	0	K	0.0001998001998002
SE_LT2_02790_p.Val352Ile	SE_LT2_02790	protein acetyltransferase	0	0	0	1	1	1	1	1	1	0	0	0	0	0	0	6	6	0	CJ	0.0001998001998002
SE_LT2_02820_p.Val175Met	SE_LT2_02820	hypothetical protein	0	0	0	1	1	1	1	1	1	0	0	0	0	0	0	6	6	0	-	0.0001998001998002
SE_LT2_02822_p.Asp137Glu	SE_LT2_02822	NAD(+) kinase	0	0	0	1	1	1	1	1	1	0	0	0	0	0	0	6	6	0	F	0.0001998001998002
SE_LT2_02828_p.Ala1572Thr	SE_LT2_02828	hypothetical protein	0	0	0	1	1	1	1	1	1	0	0	0	0	0	0	6	6	0	M	0.0001998001998002
SE_LT2_02828_p.Ala2621Val	SE_LT2_02828	hypothetical protein	0	0	0	1	1	1	0	1	1	0	0	0	0	0	0	5	5	0	M	0.00199800199800199
SE_LT2_02828_p.Arg1882His	SE_LT2_02828	hypothetical protein	0	0	0	1	1	1	1	1	1	0	0	0	0	0	0	6	6	0	M	0.0001998001998002
SE_LT2_02828_p.Asn1912Ile	SE_LT2_02828	hypothetical protein	0	0	0	1	1	1	1	1	1	0	0	0	0	0	0	6	6	0	M	0.0001998001998002
SE_LT2_02828_p.Gln1941Leu	SE_LT2_02828	hypothetical protein	0	0	0	1	1	1	1	1	1	0	0	0	0	0	0	6	6	0	M	0.0001998001998002
SE_LT2_02828_p.Gly452Val	SE_LT2_02828	hypothetical protein	0	0	0	1	1	1	1	1	1	0	0	0	0	0	0	6	6	0	M	0.0001998001998002
SE_LT2_02828_p.Lys827Asn	SE_LT2_02828	hypothetical protein	0	0	0	1	1	1	1	1	1	0	0	0	0	0	0	6	6	0	M	0.0001998001998002
SE_LT2_02828_p.Pro946Gln	SE_LT2_02828	hypothetical protein	0	0	0	1	1	1	1	1	1	0	0	0	0	0	0	6	6	0	M	0.0001998001998002
SE_LT2_02828_p.Ser2317Asn	SE_LT2_02828	hypothetical protein	0	0	0	1	1	1	1	1	1	0	0	0	0	0	0	6	6	0	M	0.0001998001998002
SE_LT2_02828_p.Thr2539Met	SE_LT2_02828	hypothetical protein	0	0	0	1	1	1	1	1	1	0	0	0	0	0	0	6	6	0	M	0.0001998001998002
SE_LT2_02828_p.Thr810Ala	SE_LT2_02828	hypothetical protein	0	0	0	1	1	1	1	1	1	0	0	0	0	0	0	6	6	0	M	0.0001998001998002
SE_LT2_02828_p.Val2507Ile	SE_LT2_02828	hypothetical protein	0	0	0	1	1	1	1	1	1	0	0	0	0	0	0	6	6	0	M	0.0001998001998002
SE_LT2_02828_p.Val825Ile	SE_LT2_02828	hypothetical protein	0	0	0	1	1	1	1	1	1	0	0	0	0	0	0	6	6	0	M	0.0001998001998002
SE_LT2_02830_p.Met371Ile	SE_LT2_02830	type I secretion protein TolC	0	0	0	1	1	1	1	1	1	0	0	0	0	0	0	6	6	0	MU	0.0001998001998002

SE_LT2_02831_p.Asp90Gly	SE_LT2_02831	putative ABC transporter transmem	0	0	0	1	1	1	1	1	1	0	0	0	0	0	0	6	6	0	V	0.0001998001998002
SE_LT2_02913_p.Asn148Asp	SE_LT2_02913	putative glycosyl transferase	0	0	0	1	1	1	1	1	1	0	0	0	0	0	0	6	6	0	CG	0.0001998001998002
SE_LT2_02915_p.Arg193His	SE_LT2_02915	enterochelin esterase	0	0	0	1	1	1	1	1	1	0	0	0	0	0	0	6	6	0	P	0.0001998001998002
SE_LT2_02918_p.Gly97Arg	SE_LT2_02918	putative inner membrane protein	0	0	0	1	1	1	1	1	1	0	0	0	0	0	0	6	6	0	-	0.0001998001998002
SE_LT2_02926_p.Asp265Glu	SE_LT2_02926	tricarboxylic transporter	0	0	0	1	1	1	1	1	1	0	0	0	0	0	0	6	6	0	S	0.0001998001998002
SE_LT2_02930_p.Thr19Ile	SE_LT2_02930	putative cytoplasmic protein	0	0	0	1	1	1	1	1	1	0	0	0	0	0	0	6	6	0	C	0.0001998001998002
SE_LT2_02932_p.Ala317Ser	SE_LT2_02932	succinate-semialdehyde dehydroge	0	0	0	1	1	1	1	1	1	0	0	0	0	0	0	6	6	0	C	0.0001998001998002
SE_LT2_02932_p.Asn55Asp	SE_LT2_02932	succinate-semialdehyde dehydroge	0	0	0	1	1	1	1	1	1	0	0	0	0	0	0	6	6	0	C	0.0001998001998002
SE_LT2_02933_p.Glu382Lys	SE_LT2_02933	4-aminobutyrate--2-oxoglutarate tra	0	0	0	1	1	1	1	1	1	0	0	0	0	0	0	6	6	0	H	0.0001998001998002
SE_LT2_02935_p.Leu152Val	SE_LT2_02935	transcriptional regulator	0	0	0	1	1	1	1	1	1	0	0	0	0	0	0	6	6	0	K	0.0001998001998002
SE_LT2_02945_p.Pro79Ser	SE_LT2_02945	DNA-binding transcriptional regulat	0	0	0	1	1	1	1	1	1	0	0	0	0	0	0	6	6	0	K	0.0001998001998002
SE_LT2_02946_p.Ala118Thr	SE_LT2_02946	alkylhydroperoxidase	0	0	0	1	1	1	1	1	1	0	0	0	0	0	0	6	6	0	S	0.0001998001998002
SE_LT2_02946_p.His98Tyr	SE_LT2_02946	alkylhydroperoxidase	0	0	0	1	1	1	1	1	1	0	0	0	0	0	0	6	6	0	S	0.0001998001998002
SE_LT2_02947_p.Ala79Val	SE_LT2_02947	NrdH-redoxin	0	0	0	1	1	1	0	1	1	0	0	0	0	0	0	5	5	0	O	0.00199800199800199
SE_LT2_02948_p.Ser10Arg	SE_LT2_02948	ribonucleotide reductase assembly	0	0	0	0	1	1	1	1	1	0	0	0	0	0	0	5	5	0	F	0.00199800199800199
SE_LT2_02949_p.Thr550Met	SE_LT2_02949	ribonucleotide-diphosphate reducta	0	0	0	1	1	1	1	1	1	0	0	0	0	0	0	6	6	0	F	0.0001998001998002
SE_LT2_02950_p.Val36Met	SE_LT2_02950	class 1b ribonucleoside-diphosphate	0	0	0	1	1	1	1	1	1	0	0	0	0	0	0	6	6	0	F	0.0001998001998002
SE_LT2_02953_p.Thr196Ile	SE_LT2_02953	glycine betaine ABC transporter sub	0	0	0	1	1	1	1	1	1	0	0	0	0	0	0	6	6	0	E	0.0001998001998002
SE_LT2_02956_p.Ala202Thr	SE_LT2_02956	multidrug export protein EmrA	0	0	0	1	1	1	1	1	1	0	0	0	0	0	0	6	6	0	V	0.0001998001998002
SE_LT2_02957_p.Pro392Ser	SE_LT2_02957	MFS transporter	0	0	0	1	1	1	1	1	1	0	0	0	0	0	0	6	6	0	U	0.0001998001998002
SE_LT2_02981_p.Thr4Ile	SE_LT2_02981	transcriptional regulator GutM	0	0	0	1	1	1	1	1	1	0	0	0	0	0	0	6	6	0	K	0.0001998001998002
SE_LT2_02986_p.Ala117Thr	SE_LT2_02986	FAD-dependent oxidoreductase	0	0	0	1	1	1	1	1	1	0	0	0	0	0	0	6	6	0	C	0.0001998001998002
SE_LT2_02987_p.Thr445Ile	SE_LT2_02987	carbamoyltransferase HypF	0	0	0	1	1	1	1	1	1	0	0	0	0	0	0	6	6	0	O	0.0001998001998002
SE_LT2_02990_p.Glu24Gly	SE_LT2_02990	hydrogenase maturation peptidase	0	0	0	1	1	1	1	1	1	0	0	0	0	0	0	6	6	0	E	0.0001998001998002
SE_LT2_02996_p.Gly147Ser	SE_LT2_02996	formate hydrogenlyase subunit 3	0	0	0	1	1	1	1	1	1	0	0	0	0	0	0	6	6	0	CP	0.0001998001998002
SE_LT2_03023_p.Arg542Ser	SE_LT2_03023	transcriptional regulator	0	0	0	1	1	1	1	1	1	0	0	0	0	0	0	6	6	0	K	0.0001998001998002
SE_LT2_03047_p.Ala139Thr	SE_LT2_03047	invasion lipoprotein InvH	0	0	0	1	1	1	1	1	1	0	0	0	0	0	0	6	6	0	S	0.0001998001998002
SE_LT2_03056_p.Ter856Ter	SE_LT2_03056	DNA mismatch repair protein MutS	0	0	0	1	1	1	1	1	1	0	0	0	0	0	0	6	6	0	F	0.0001998001998002
SE_LT2_03056_p.Thr583Ile	SE_LT2_03056	DNA mismatch repair protein MutS	0	0	0	1	1	1	1	1	1	0	0	0	0	0	0	6	6	0	F	0.0001998001998002
SE_LT2_03057_p.GlyVal22GluMe	SE_LT2_03057	hypothetical protein	0	0	0	1	1	1	1	1	1	0	0	0	0	0	0	6	6	0	S	0.0001998001998002
SE_LT2_03058_p.Ala191Thr	SE_LT2_03058	MFS transporter	0	0	0	1	1	1	1	1	1	0	0	0	0	0	0	6	6	0	EGP	0.0001998001998002
SE_LT2_03058_p.Lys124Glu	SE_LT2_03058	MFS transporter	0	0	0	1	1	1	1	1	1	0	0	0	0	0	0	6	6	0	EGP	0.0001998001998002
SE_LT2_03059_p.Asp295Asn	SE_LT2_03059	putative LysR family transcriptional	0	0	0	1	1	1	1	1	1	0	0	0	0	0	0	6	6	0	K	0.0001998001998002
SE_LT2_03060_p.Gln199Arg	SE_LT2_03060	gluconate permease	0	0	0	1	1	1	1	1	1	0	0	0	0	0	0	6	6	0	EG	0.0001998001998002
SE_LT2_03064_p.Arg277Cys	SE_LT2_03064	3-oxo-tetronate kinase	0	0	0	1	1	1	1	1	1	0	0	0	0	0	0	6	6	0	S	0.0001998001998002
SE_LT2_03070_p.Ala9Thr	SE_LT2_03070	Protein VdcD	0	0	0	1	1	1	1	1	1	0	0	0	0	0	0	6	6	0	S	0.0001998001998002
SE_LT2_03075_p.His82Tyr	SE_LT2_03075	tRNA pseudouridine(13) synthase Tr	0	0	0	1	1	1	1	1	1	0	0	0	0	0	0	6	6	0	J	0.0001998001998002
SE_LT2_03079_p.Ala93Val	SE_LT2_03079	putative inner membrane protein	0	0	0	1	1	1	1	1	1	0	0	0	0	0	0	6	6	0	S	0.0001998001998002
SE_LT2_03080_p.Ala153Ser	SE_LT2_03080	adenylyl-sulfate kinase	0	0	0	1	1	1	1	1	1	0	0	0	0	0	0	6	6	0	F	0.0001998001998002
SE_LT2_03081_p.Val446Met	SE_LT2_03081	sulfate adenylyltransferase subunit	0	0	0	1	1	1	1	1	1	0	0	0	0	0	0	6	6	0	H	0.0001998001998002
SE_LT2_03094_p.Glu533Gln	SE_LT2_03094	sulfite reductase subunit beta	0	0	0	1	1	1	1	1	1	0	0	0	0	0	0	6	6	0	H	0.0001998001998002
SE_LT2_03105_p.Val265Ile	SE_LT2_03105	23S rRNA (uracil(1939)-C(5))-methyl	0	0	0	1	1	1	1	1	1	0	0	0	0	0	0	6	6	0	J	0.0001998001998002
SE_LT2_03105_p.Val401Ala	SE_LT2_03105	23S rRNA (uracil(1939)-C(5))-methyl	0	0	0	1	1	1	1	1	1	0	0	0	0	0	0	6	6	0	J	0.0001998001998002
SE_LT2_03106_p.Pro797Gln	SE_LT2_03106	two-component sensor histidine kir	0	0	0	1	1	1	1	1	1	0	0	0	0	0	0	6	6	0	T	0.0001998001998002
SE_LT2_03107_p.Ala57Val	SE_LT2_03107	glycerate kinase	0	0	0	1	1	1	1	1	1	0	0	0	0	0	0	6	6	0	G	0.0001998001998002
SE_LT2_03107_p.Ser177Arg	SE_LT2_03107	glycerate kinase	0	0	0	1	1	1	1	1	1	0	0	0	0	0	0	6	6	0	G	0.0001998001998002
SE_LT2_03109_p.Arg225Cys	SE_LT2_03109	putative d-glucarate dehydratase	0	0	0	1	1	1	1	1	1	0	0	0	0	0	0	6	6	0	M	0.0001998001998002
SE_LT2_03121_p.Glu316Gly	SE_LT2_03121	lactaldehyde reductase	0	0	0	1	1	1	1	1	1	0	0	0	0	0	0	6	6	0	C	0.0001998001998002
SE_LT2_03124_p.Glu228Ala	SE_LT2_03124	L-fucose isomerase	0	0	0	1	1	1	1	1	1	0	0	0	0	0	0	6	6	0	G	0.0001998001998002
SE_LT2_03130_p.Ala244Ser	SE_LT2_03130	transcriptional regulator GcvA	0	0	0	1	1	1	1	1	1	0	0	0	0	0	0	6	6	0	K	0.0001998001998002

SE_LT2_03134_p.Ser326Ala	SE_LT2_03134	cysteine sulfinate desulfinase	0	0	0	1	1	1	1	1	1	0	0	0	0	0	0	6	6	0	E		0.0001998001998002
SE_LT2_03137_p.Cys220Trp	SE_LT2_03137	tRNA cyclic N6-threonylcarbamoylad	0	0	0	1	1	1	1	1	1	0	0	0	0	0	0	6	6	0	H		0.0001998001998002
SE_LT2_03143_p.Asp149Glu	SE_LT2_03143	exodeoxyribonuclease V subunit alp	0	0	0	1	0	1	1	1	1	0	0	0	0	0	0	5	5	0	L		0.00199800199800199
SE_LT2_03143_p.Gly406Arg	SE_LT2_03143	exodeoxyribonuclease V subunit alp	0	0	0	1	1	1	1	1	1	0	0	0	0	0	0	6	6	0	L		0.0001998001998002
SE_LT2_03143_p.Thr343Met	SE_LT2_03143	exodeoxyribonuclease V subunit alp	0	0	0	1	1	1	1	1	1	0	0	0	0	0	0	6	6	0	L		0.0001998001998002
SE_LT2_03143_p.Thr427Ala	SE_LT2_03143	exodeoxyribonuclease V subunit alp	0	0	0	1	1	1	1	1	1	0	0	0	0	0	0	6	6	0	L		0.0001998001998002
SE_LT2_03145_p.Ile219Ser	SE_LT2_03145	pitrilysin	0	0	0	1	1	1	1	1	1	0	0	0	0	0	0	6	6	0	O		0.0001998001998002
SE_LT2_03146_p.His759Tyr	SE_LT2_03146	exodeoxyribonuclease V subunit ga	0	0	0	1	1	1	1	1	1	0	0	0	0	0	0	6	6	0	L		0.0001998001998002
SE_LT2_03151_p.Pro102Ser	SE_LT2_03151	thymidylate synthase	0	0	0	1	1	1	1	1	1	0	0	0	0	0	0	6	6	0	F		0.0001998001998002
SE_LT2_03160_p.Ala367Glu	SE_LT2_03160	bifunctional 2-acylglycerophosphoe	0	0	0	1	1	1	1	1	1	0	0	0	0	0	0	6	6	0	I		0.0001998001998002
SE_LT2_03169_p.Glu47Asp	SE_LT2_03169	2-deoxy-D-gluconate 3-dehydrogen	0	0	0	1	1	1	1	1	1	0	0	0	0	0	0	6	6	0	IQ		0.0001998001998002
SE_LT2_03169_p.Gly71Ser	SE_LT2_03169	2-deoxy-D-gluconate 3-dehydrogen	0	0	0	1	1	1	1	1	1	0	0	0	0	0	0	6	6	0	IQ		0.0001998001998002
SE_LT2_03185_p.Arg75Cys	SE_LT2_03185	resistance to complement killing	0	0	0	1	1	1	1	1	1	0	0	0	0	0	0	6	6	0	M		0.0001998001998002
SE_LT2_03185_p.Asn54Ser	SE_LT2_03185	resistance to complement killing	0	0	0	1	0	1	1	1	1	0	0	0	0	0	0	5	5	0	M		0.00199800199800199
SE_LT2_03197_p.Ala153Ser	SE_LT2_03197	isopentenyl-diphosphate delta-ison	0	0	0	1	1	1	1	1	1	0	0	0	0	0	0	6	6	0	F		0.0001998001998002
SE_LT2_03204_p.Ala26Ser	SE_LT2_03204	Inner membrane protein YgfX	0	0	0	1	1	1	1	1	1	0	0	0	0	0	0	6	6	0	S		0.0001998001998002
SE_LT2_03209_p.Gly16Val	SE_LT2_03209	ASCH domain-containing protein	0	0	0	1	1	1	1	1	1	0	0	0	0	0	0	6	6	0	S		0.0001998001998002
SE_LT2_03215_p.Pro220Ser	SE_LT2_03215	FAD-dependent 2-octaprenylpheno	0	0	0	1	1	1	1	1	1	0	0	0	0	0	0	6	6	0	CH		0.0001998001998002
SE_LT2_03217_p.Thr229Ile	SE_LT2_03217	Xaa-Pro aminopeptidase	0	0	0	1	1	1	1	1	1	0	0	0	0	0	0	6	6	0	E		0.0001998001998002
SE_LT2_03226_p.Ala174Val	SE_LT2_03226	oxidative stress defense protein	0	0	0	1	1	1	1	1	1	0	0	0	0	0	0	6	6	0	S		0.0001998001998002
SE_LT2_03233_p.Ala2Ser	SE_LT2_03233	ECF transporter S component	0	0	0	1	1	1	1	1	1	0	0	0	0	0	0	6	6	0	T		0.0001998001998002
SE_LT2_03235_p.His139Tyr	SE_LT2_03235	cobalt ABC transporter ATP-binding	0	0	0	1	1	1	1	1	1	0	0	0	0	0	0	6	6	0	P		0.0001998001998002
SE_LT2_03236_p.Leu82Arg	SE_LT2_03236	ABC transporter ATP-binding protei	0	0	0	1	1	1	1	1	1	0	0	0	0	0	0	6	6	0	P		0.0001998001998002
SE_LT2_03236_p.Ser109Pro	SE_LT2_03236	ABC transporter ATP-binding protei	0	0	0	1	1	1	1	1	1	0	0	0	0	0	0	6	6	0	P		0.0001998001998002
SE_LT2_03237_p.Thr572Ala	SE_LT2_03237	transketolase	0	0	0	1	1	1	1	1	1	0	0	0	0	0	0	6	6	0	G		0.0001998001998002
SE_LT2_03237_p.Tyr105Phe	SE_LT2_03237	transketolase	0	0	0	1	1	1	1	1	1	0	0	0	0	0	0	6	6	0	G		0.0001998001998002
SE_LT2_03244_p.Ala457Val	SE_LT2_03244	fructuronate reductase	0	0	0	1	1	1	1	1	1	0	0	0	0	0	0	6	6	0	G		0.0001998001998002
SE_LT2_03246_p.Leu221Gln	SE_LT2_03246	hypothetical protein	0	0	0	1	1	1	1	1	1	0	0	0	0	0	0	6	6	0	-		0.0001998001998002
SE_LT2_03262_p.Leu52Ile	SE_LT2_03262	RdgB/HAM1 family non-canonical pu	0	0	0	1	1	1	1	1	1	0	0	0	0	0	0	6	6	0	F		0.0001998001998002
SE_LT2_03263_p.Arg362Gln	SE_LT2_03263	YggW family oxidoreductase	0	0	0	1	1	1	1	1	1	0	0	0	0	0	0	6	6	0	H		0.0001998001998002
SE_LT2_03263_p.Asn45Ser	SE_LT2_03263	YggW family oxidoreductase	0	0	0	1	1	1	1	1	1	0	0	0	0	0	0	6	6	0	H		0.0001998001998002
SE_LT2_03263_p.Gly276Ser	SE_LT2_03263	YggW family oxidoreductase	0	0	0	1	1	1	1	1	1	0	0	0	0	0	0	6	6	0	H		0.0001998001998002
SE_LT2_03263_p.Leu296Gln	SE_LT2_03263	YggW family oxidoreductase	0	0	0	1	1	1	1	1	1	0	0	0	0	0	0	6	6	0	H		0.0001998001998002
SE_LT2_03264_p.Val309Leu	SE_LT2_03264	hypothetical protein	0	0	0	1	1	1	1	1	1	0	0	0	0	0	0	6	6	0	S		0.0001998001998002
SE_LT2_03273_p.Ala565Glu	SE_LT2_03273	ornithine decarboxylase	0	0	0	1	1	1	1	1	1	0	0	0	0	0	0	6	6	0	E		0.0001998001998002
SE_LT2_03273_p.Gly10Ser	SE_LT2_03273	ornithine decarboxylase	0	0	0	1	1	1	1	1	1	0	0	0	0	0	0	6	6	0	E		0.0001998001998002
SE_LT2_03284_p.Ala147Ser	SE_LT2_03284	helix-turn-helix transcriptional regu	0	0	0	1	1	1	1	1	1	0	0	0	0	0	0	6	6	0	K		0.0001998001998002
SE_LT2_03284_p.AlaGlnHis224Se	SE_LT2_03284	helix-turn-helix transcriptional regu	0	0	0	1	1	1	1	1	1	0	0	0	0	0	0	6	6	0	K		0.0001998001998002
SE_LT2_03286_p.His188Tyr	SE_LT2_03286	putative amino acid transporter	0	0	0	1	1	1	1	1	1	0	0	0	0	0	0	6	6	0	E		0.0001998001998002
SE_LT2_03286_p.Leu149Ile	SE_LT2_03286	putative amino acid transporter	0	0	0	1	1	1	1	1	1	0	0	0	0	0	0	6	6	0	E		0.0001998001998002
SE_LT2_03289_p.Ala224Val	SE_LT2_03289	NAD-dependent phenylacetaldehyd	0	0	0	1	1	1	1	1	1	0	0	0	0	0	0	6	6	0	C		0.0001998001998002
SE_LT2_03300_p.Gly19Ser	SE_LT2_03300	glutathione-dependent disulfide-bo	0	0	0	1	1	1	1	1	1	0	0	0	0	0	0	6	6	0	O		0.0001998001998002
SE_LT2_03302_p.Pro100Leu	SE_LT2_03302	putative protein	0	0	0	1	1	1	1	1	1	0	0	0	0	0	0	6	6	0	P		0.0001998001998002
SE_LT2_03312_p.Ala172Thr	SE_LT2_03312	putative methyl-accepting chemota	0	0	0	1	1	1	1	1	1	0	0	0	0	0	0	6	6	0	NT		0.0001998001998002
SE_LT2_03313_p.Ser88Thr	SE_LT2_03313	hypothetical protein	0	0	0	1	1	1	1	1	1	0	0	0	0	0	0	6	6	0	S		0.0001998001998002
SE_LT2_03314_p.Thr85Ala	SE_LT2_03314	putative ATP-dependent RNA helica	0	0	0	1	1	1	1	1	1	0	0	0	0	0	0	6	6	0	-		0.0001998001998002
SE_LT2_03321_p.Val371Ile	SE_LT2_03321	cystathionine beta-lyase	0	0	0	1	1	1	1	1	1	0	0	0	0	0	0	6	6	0	E		0.0001998001998002
SE_LT2_03327_p.MetHis1?	SE_LT2_03327	putative cation transporter	0	0	0	1	1	1	1	1	1	0	0	0	0	0	0	6	6	0	P		0.0001998001998002
SE_LT2_03331_p.Ala298Val	SE_LT2_03331	C4-dicarboxylate ABC transporter su	0	0	0	1	1	1	1	1	1	0	0	0	0	0	0	6	6	0	G		0.0001998001998002
SE_LT2_03331_p.Glu279Gly	SE_LT2_03331	C4-dicarboxylate ABC transporter su	0	0	0	1	1	1	1	1	1	0	0	0	0	0	0	6	6	0	G		0.0001998001998002

SE_LT2_03336_p.Arg359Cys	SE_LT2_03336	DNA topoisomerase IV, subunit A	0	0	0	1	1	1	1	1	1	0	0	0	0	0	0	6	6	0	L	0.0001998001998002
SE_LT2_03336_p.Glu637Lys	SE_LT2_03336	DNA topoisomerase IV, subunit A	0	0	0	1	1	1	1	1	1	0	0	0	0	0	0	6	6	0	L	0.0001998001998002
SE_LT2_03340_p.Ile169Leu	SE_LT2_03340	two-component system sensor histi	0	0	0	1	1	1	1	1	1	0	0	0	0	0	0	6	6	0	T	0.0001998001998002
SE_LT2_03367_p.His415Tyr	SE_LT2_03367	CYTH domain-containing protein	0	0	0	1	1	1	1	1	1	0	0	0	0	0	0	6	6	0	S	0.0001998001998002
SE_LT2_03412_p.Ser220Asn	SE_LT2_03412	transcriptional regulator TdcA	0	0	0	1	1	1	1	1	1	0	0	0	0	0	0	6	6	0	K	0.0001998001998002
SE_LT2_03432_p.Arg274His	SE_LT2_03432	penicillin-binding protein activator	0	0	0	1	1	1	1	1	1	0	0	0	0	0	0	6	6	0	M	0.0001998001998002
SE_LT2_03432_p.Asp169Glu	SE_LT2_03432	penicillin-binding protein activator	0	0	0	1	1	1	1	1	1	0	0	0	0	0	0	6	6	0	M	0.0001998001998002
SE_LT2_03433_p.Arg55Cys	SE_LT2_03433	YraN family protein	0	0	0	1	1	1	1	1	1	0	0	0	0	0	0	6	6	0	L	0.0001998001998002
SE_LT2_03438_p.His23Tyr	SE_LT2_03438	hypothetical protein	0	0	0	0	1	1	1	1	1	0	0	0	0	0	0	5	5	0	S	0.00199800199800199
SE_LT2_03444_p.Ile165Val	SE_LT2_03444	hypothetical protein	0	0	0	1	1	1	1	1	1	0	0	0	0	0	0	6	6	0	C	0.0001998001998002
SE_LT2_03444_p.Val10Ala	SE_LT2_03444	hypothetical protein	0	0	0	1	1	1	1	1	1	0	0	0	0	0	0	6	6	0	C	0.0001998001998002
SE_LT2_03447_p.GlnProAla176Th	SE_LT2_03447	tryptophan permease	0	0	0	0	1	1	1	1	1	0	0	0	0	0	0	5	5	0	U	0.00199800199800199
SE_LT2_03448_p.Arg102Ser	SE_LT2_03448	cysteine sulfinate desulfinase	0	0	0	1	1	1	1	1	1	0	0	0	0	0	0	6	6	0	F	0.0001998001998002
SE_LT2_03461_p.Thr357Ser	SE_LT2_03461	argininosuccinate synthetase	0	0	0	1	1	1	1	1	1	0	0	0	0	0	0	6	6	0	F	0.0001998001998002
SE_LT2_03467_p.Glu514Asp	SE_LT2_03467	ATP-dependent metalloprotease	0	0	0	1	1	1	1	1	1	0	0	0	0	0	0	6	6	0	O	0.0001998001998002
SE_LT2_03485_p.Ala165Thr	SE_LT2_03485	outer membrane lipid asymmetry m	0	0	0	1	1	1	1	1	1	0	0	0	0	0	0	6	6	0	Q	0.0001998001998002
SE_LT2_03489_p.Met45Ile	SE_LT2_03489	D-arabinose 5-phosphate isomerase	0	0	0	1	1	1	1	1	1	0	0	0	0	0	0	6	6	0	M	0.0001998001998002
SE_LT2_03508_p.Val230Ile	SE_LT2_03508	cytosine permease	0	0	0	1	1	1	1	1	1	0	0	0	0	0	0	6	6	0	F	0.0001998001998002
SE_LT2_03510_p.Ala154Thr	SE_LT2_03510	toxin-antitoxin biofilm protein TabA	0	0	0	1	1	1	1	1	1	0	0	0	0	0	0	6	6	0	G	0.0001998001998002
SE_LT2_03525_p.Thr104Ala	SE_LT2_03525	2-(5''-triphosphoribosyl)-3'-dephosp	0	0	0	1	1	1	1	1	1	0	0	0	0	0	0	6	6	0	H	0.0001998001998002
SE_LT2_03525_p.Val140Ile	SE_LT2_03525	2-(5''-triphosphoribosyl)-3'-dephosp	0	0	0	1	1	1	1	1	1	0	0	0	0	0	0	6	6	0	H	0.0001998001998002
SE_LT2_03533_p.Ser36Cys	SE_LT2_03533	GntR family transcriptional regulato	0	0	0	1	1	1	1	1	1	0	0	0	0	0	0	6	6	0	K	0.0001998001998002
SE_LT2_03543_p.Ala345Thr	SE_LT2_03543	metalloprotease TldD	0	0	0	1	1	1	1	1	1	0	0	0	0	0	0	6	6	0	S	0.0001998001998002
SE_LT2_03544_p.Ser1061Asn	SE_LT2_03544	TIGR02099 family protein	0	0	0	1	1	1	1	1	1	0	0	0	0	0	0	6	6	0	S	0.0001998001998002
SE_LT2_03553_p.Ile122Val	SE_LT2_03553	sulfoxide reductase heme-binding s	0	0	0	1	1	1	1	1	1	0	0	0	0	0	0	6	6	0	C	0.0001998001998002
SE_LT2_03557_p.Ala372Val	SE_LT2_03557	sodium/panthothenate symporter	0	0	0	1	1	1	1	1	1	0	0	0	0	0	0	6	6	0	H	0.0001998001998002
SE_LT2_03557_p.Ser211Asn	SE_LT2_03557	sodium/panthothenate symporter	0	0	0	1	1	1	1	1	1	0	0	0	0	0	0	6	6	0	H	0.0001998001998002
SE_LT2_03564_p.His409Tyr	SE_LT2_03564	histidine kinase	0	0	0	1	1	1	1	1	1	0	0	0	0	0	0	6	6	0	T	0.0001998001998002
SE_LT2_03566_p.Ala34Val	SE_LT2_03566	multidrug exporter AcrE	0	0	0	1	1	1	1	1	1	0	0	0	0	0	0	6	6	0	M	0.0001998001998002
SE_LT2_03579_p.Ala168Val	SE_LT2_03579	L-threonylcarbamoyladenylate synt	0	0	0	1	1	1	1	1	1	0	0	0	0	0	0	6	6	0	J	0.0001998001998002
SE_LT2_03648_p.Glu79Lys	SE_LT2_03648	cell filamentation protein Fic	0	0	0	1	1	1	1	1	1	0	0	0	0	0	0	6	6	0	D	0.0001998001998002
SE_LT2_03652_p.Ala788Glu	SE_LT2_03652	nitrite reductase large subunit	0	0	0	1	1	1	1	1	1	0	0	0	0	0	0	6	6	0	C	0.0001998001998002
SE_LT2_03652_p.Glu311Ala	SE_LT2_03652	nitrite reductase large subunit	0	0	0	1	1	1	1	1	1	0	0	0	0	0	0	6	6	0	C	0.0001998001998002
SE_LT2_03652_p.Ile730Leu	SE_LT2_03652	nitrite reductase large subunit	0	0	0	1	1	1	1	1	1	0	0	0	0	0	0	6	6	0	C	0.0001998001998002
SE_LT2_03670_p.Thr147Met	SE_LT2_03670	hypothetical protein	0	0	0	1	1	1	1	1	1	0	0	0	0	0	0	6	6	0	S	0.0001998001998002
SE_LT2_03679_p.Thr176Ile	SE_LT2_03679	hypothetical protein	0	0	0	1	0	1	1	1	1	0	0	0	0	0	0	5	5	0	L	0.00199800199800199
SE_LT2_03680_p.Gly106Asp	SE_LT2_03680	phosphoenolpyruvate carboxykinas	0	0	0	1	1	1	1	1	1	0	0	0	0	0	0	6	6	0	F	0.0001998001998002
SE_LT2_03684_p.Ala271Ser	SE_LT2_03684	putative RNase R	0	0	0	1	1	1	1	1	1	0	0	0	0	0	0	6	6	0	K	0.0001998001998002
SE_LT2_03684_p.Asp260Asn	SE_LT2_03684	putative RNase R	0	0	0	1	1	1	1	1	1	0	0	0	0	0	0	6	6	0	K	0.0001998001998002
SE_LT2_03686_p.Arg757His	SE_LT2_03686	ferrous iron transporter B	0	0	0	1	1	1	1	1	1	0	0	0	0	0	0	6	6	0	P	0.0001998001998002
SE_LT2_03692_p.Val293Ile	SE_LT2_03692	gluconate transporter	0	0	0	1	1	1	1	1	1	0	0	0	0	0	0	6	6	0	EG	0.0001998001998002
SE_LT2_03693_p.Ala50Ser	SE_LT2_03693	4-alpha-glucanotransferase	0	0	0	1	1	1	1	1	1	0	0	0	0	0	0	6	6	0	G	0.0001998001998002
SE_LT2_03694_p.Arg378Ser	SE_LT2_03694	maltodextrin phosphorylase	0	0	0	1	1	1	1	1	1	0	0	0	0	0	0	6	6	0	G	0.0001998001998002
SE_LT2_03694_p.Glu193Lys	SE_LT2_03694	maltodextrin phosphorylase	0	0	0	1	1	1	1	1	1	0	0	0	0	0	0	6	6	0	G	0.0001998001998002
SE_LT2_03710_p.Ala49Thr	SE_LT2_03710	hypothetical protein	0	0	0	1	1	1	1	1	1	0	0	0	0	0	0	6	6	0	-	0.0001998001998002
SE_LT2_03710_p.Asp108Glu	SE_LT2_03710	hypothetical protein	0	0	0	1	1	1	1	1	1	0	0	0	0	0	0	6	6	0	-	0.0001998001998002
SE_LT2_03710_p.Ser136Pro	SE_LT2_03710	hypothetical protein	0	0	0	1	1	1	1	1	1	0	0	0	0	0	0	6	6	0	-	0.0001998001998002
SE_LT2_03712_p.Ala324Val	SE_LT2_03712	putative glycerol dehydrogenase	0	0	0	1	1	1	1	1	1	0	0	0	0	0	0	6	6	0	C	0.0001998001998002
SE_LT2_03713_p.Val316Ala	SE_LT2_03713	MFS transporter	0	0	0	1	1	1	1	1	1	0	0	0	0	0	0	6	6	0	G	0.0001998001998002
SE_LT2_03715_p.Ile153Met	SE_LT2_03715	dihydrodipicolinate synthase family	0	0	0	1	1	1	1	1	1	0	0	0	0	0	0	6	6	0	EM	0.0001998001998002

SE_LT2_03720_p.Leu306Ile	SE_LT2_03720	glycogen-debranching enzyme GlgX	0	0	0	1	1	1	1	1	1	0	0	0	0	0	0	6	6	0	G	0.0001998001998002
SE_LT2_03723_p.Ser231Ala	SE_LT2_03723	gluconate transporter	0	0	0	1	1	1	1	1	1	0	0	0	0	0	0	6	6	0	EG	0.0001998001998002
SE_LT2_03725_p.Asp267Gly	SE_LT2_03725	transcriptional regulator	0	0	0	1	1	1	1	1	1	0	0	0	0	0	0	6	6	0	K	0.0001998001998002
SE_LT2_03735_p.Thr263Ile	SE_LT2_03735	gamma-glutamyltransferase	0	0	0	1	1	1	1	1	1	0	0	0	0	0	0	6	6	0	M	0.0001998001998002
SE_LT2_03736_	SE_LT2_03736	putative protein YhhA	0	0	0	1	1	1	1	1	1	0	0	0	0	0	0	6	6	0	S	0.0001998001998002
SE_LT2_03737_p.Ala158Val	SE_LT2_03737	glycerophosphodiester phosphodie	0	0	0	1	1	1	1	1	1	0	0	0	0	0	0	6	6	0	C	0.0001998001998002
SE_LT2_03759_p.Ala525Thr	SE_LT2_03759	zinc/cadmium/mercury/lead-transp	0	0	0	1	0	1	1	1	1	0	0	0	0	0	0	5	5	0	P	0.00199800199800199
SE_LT2_03769_p.Lys80Arg	SE_LT2_03769	putative ABC-type multidrug transp	0	0	0	1	1	1	1	1	1	0	0	0	0	0	0	6	6	0	V	0.0001998001998002
SE_LT2_03776_p.Ala363dup	SE_LT2_03776	dipeptide/tripeptide permease	0	0	0	1	1	1	1	1	1	0	0	0	0	0	0	6	6	0	U	0.0001998001998002
SE_LT2_03776_p.Gly486Asp	SE_LT2_03776	dipeptide/tripeptide permease	0	0	0	1	1	1	1	1	1	0	0	0	0	0	0	6	6	0	U	0.0001998001998002
SE_LT2_03780_p.Ala66Ser	SE_LT2_03780	23S rRNA (adenine(2030)-N(6))-met	0	0	0	1	1	1	1	1	1	0	0	0	0	0	0	6	6	0	H	0.0001998001998002
SE_LT2_03781_p.Thr74Ala	SE_LT2_03781	glutathione-disulfide reductase	0	0	0	1	1	1	1	1	1	0	0	0	0	0	0	6	6	0	C	0.0001998001998002
SE_LT2_03782_p.Val63Glu	SE_LT2_03782	putative L-asparaginase	0	0	0	1	1	1	1	1	1	0	0	0	0	0	0	6	6	0	EJ	0.0001998001998002
SE_LT2_03784_p.Ala217Thr	SE_LT2_03784	fructoselysine 6-kinase	0	0	0	1	1	1	1	1	1	0	0	0	0	0	0	6	6	0	G	0.0001998001998002
SE_LT2_03786_p.Ter240Ter	SE_LT2_03786	GntR family transcriptional regulato	0	0	0	1	1	1	1	1	1	0	0	0	0	0	0	6	6	0	K	0.0001998001998002
SE_LT2_03788_p.GlyAla342AlaGl	SE_LT2_03788	hypothetical protein	0	0	0	1	1	1	1	1	1	0	0	0	0	0	0	6	6	0	-	0.0001998001998002
SE_LT2_03788_p.Phe75Leu	SE_LT2_03788	hypothetical protein	0	0	0	1	1	1	1	1	1	0	0	0	0	0	0	6	6	0	-	0.0001998001998002
SE_LT2_03788_p.Thr480Ile	SE_LT2_03788	hypothetical protein	0	0	0	1	1	1	1	1	1	0	0	0	0	0	0	6	6	0	-	0.0001998001998002
SE_LT2_03790_p.Val64Met	SE_LT2_03790	helix-turn-helix transcriptional regu	0	0	0	1	1	1	1	1	1	0	0	0	0	0	0	6	6	0	K	0.0001998001998002
SE_LT2_03792_p.Ser128Asn	SE_LT2_03792	putative LysR family transcriptional	0	0	0	1	1	1	1	1	1	0	0	0	0	0	0	6	6	0	K	0.0001998001998002
SE_LT2_03793_p.Gln3Lys	SE_LT2_03793	Inner membrane protein YhjD	0	0	0	1	1	1	1	1	1	0	0	0	0	0	0	6	6	0	S	0.0001998001998002
SE_LT2_03795_p.Ala651Thr	SE_LT2_03795	hypothetical protein	0	0	0	1	1	1	1	1	1	0	0	0	0	0	0	6	6	0	M	0.0001998001998002
SE_LT2_03795_p.Ile509Val	SE_LT2_03795	hypothetical protein	0	0	0	1	1	1	1	1	1	0	0	0	0	0	0	6	6	0	M	0.0001998001998002
SE_LT2_03797_p.Gly277Asp	SE_LT2_03797	ketodeoxygluconokinase	0	0	0	1	1	1	1	1	1	0	0	0	0	0	0	6	6	0	G	0.0001998001998002
SE_LT2_03801_p.Ala753Thr	SE_LT2_03801	cellulose biosynthesis protein BcsC	0	0	0	1	1	1	1	1	1	0	0	0	0	0	0	6	6	0	O	0.0001998001998002
SE_LT2_03801_p.Val736Ile	SE_LT2_03801	cellulose biosynthesis protein BcsC	0	0	0	1	1	1	1	1	1	0	0	0	0	0	0	6	6	0	O	0.0001998001998002
SE_LT2_03807_p.Asp60Glu	SE_LT2_03807	putative cytoplasmic protein	0	0	0	1	1	1	1	1	1	0	0	0	0	0	0	6	6	0	S	0.0001998001998002
SE_LT2_03807_p.Gln33Lys	SE_LT2_03807	putative cytoplasmic protein	0	0	0	1	1	1	1	1	1	0	0	0	0	0	0	6	6	0	S	0.0001998001998002
SE_LT2_03812_p.Leu184Val	SE_LT2_03812	transporter	0	0	0	1	1	1	1	1	1	0	0	0	0	0	0	6	6	0	E	0.0001998001998002
SE_LT2_03823_p.ValGln557*	SE_LT2_03823	lipid A phosphoethanolamine transf	0	0	0	1	1	1	1	1	1	0	0	0	0	0	0	6	6	0	S	0.0001998001998002
SE_LT2_03827_p.Ala120Val	SE_LT2_03827	long polar fimbrial chaperone LpfB	0	0	0	1	1	1	1	1	1	0	0	0	0	0	0	6	6	0	M	0.0001998001998002
SE_LT2_03832_p.Asn572Asp	SE_LT2_03832	trimethylamine N-oxide reductase I	0	0	0	1	1	1	1	1	1	0	0	0	0	0	0	6	6	0	C	0.0001998001998002
SE_LT2_03834_p.Ala97Thr	SE_LT2_03834	bifunctional glyoxylate/hydroxypyru	0	0	0	1	1	1	1	1	1	0	0	0	0	0	0	6	6	0	C	0.0001998001998002
SE_LT2_03834_p.Val151Ile	SE_LT2_03834	bifunctional glyoxylate/hydroxypyru	0	0	0	1	1	1	1	1	1	0	0	0	0	0	0	6	6	0	C	0.0001998001998002
SE_LT2_03839_p.Ser4Pro	SE_LT2_03839	hypothetical protein	0	0	0	1	1	1	1	1	1	0	0	0	0	0	0	6	6	0	-	0.0001998001998002
SE_LT2_03844_p.Asn201Ser	SE_LT2_03844	glycine--tRNA ligase subunit beta	0	0	0	1	1	1	1	1	1	0	0	0	0	0	0	6	6	0	J	0.0001998001998002
SE_LT2_03846_p.Thr36Arg	SE_LT2_03846	hypothetical protein	0	0	0	1	1	1	1	1	1	0	0	0	0	0	0	6	6	0	M	0.0001998001998002
SE_LT2_03847_p.Val109Ala	SE_LT2_03847	acetyltransferase	0	0	0	1	1	1	1	1	1	0	0	0	0	0	0	6	6	0	M	0.0001998001998002
SE_LT2_03849_p.Ala463Thr	SE_LT2_03849	xylulokinase	0	0	0	1	1	1	1	1	1	0	0	0	0	0	0	6	6	0	F	0.0001998001998002
SE_LT2_03849_p.Arg54Cys	SE_LT2_03849	xylulokinase	0	0	0	1	1	1	1	1	1	0	0	0	0	0	0	6	6	0	F	0.0001998001998002
SE_LT2_03853_p.Asp425Gly	SE_LT2_03853	alpha-amylase	0	0	0	1	1	1	1	1	1	0	0	0	0	0	0	6	6	0	M	0.0001998001998002
SE_LT2_03853_p.Thr474Ala	SE_LT2_03853	alpha-amylase	0	0	0	1	1	1	1	1	1	0	0	0	0	0	0	6	6	0	M	0.0001998001998002
SE_LT2_03855_p.His146Arg	SE_LT2_03855	electron transporter	0	0	0	1	1	1	1	1	1	0	0	0	0	0	0	6	6	0	C	0.0001998001998002
SE_LT2_03855_p.Thr36Met	SE_LT2_03855	electron transporter	0	0	0	1	1	1	1	1	1	0	0	0	0	0	0	6	6	0	C	0.0001998001998002
SE_LT2_03880_p.Ala960Val	SE_LT2_03880	Autotransporter adhesin SadA	0	0	0	1	1	1	1	1	1	0	0	0	0	0	0	6	6	0	UW	0.0001998001998002
SE_LT2_03880_p.Asp954Asn	SE_LT2_03880	Autotransporter adhesin SadA	0	0	0	1	1	1	1	1	1	0	0	0	0	0	0	6	6	0	UW	0.0001998001998002
SE_LT2_03880_p.Lys171Arg	SE_LT2_03880	Autotransporter adhesin SadA	0	0	0	1	1	1	1	1	1	0	0	0	0	0	0	6	6	0	UW	0.0001998001998002
SE_LT2_03895_p.Ala390Ser	SE_LT2_03895	murein hydrolase activator EnvC	0	0	0	1	1	1	1	1	1	0	0	0	0	0	0	6	6	0	D	0.0001998001998002
SE_LT2_03897_p.Val184Ala	SE_LT2_03897	putative glycosyltransferase	0	0	0	1	1	1	1	1	1	0	0	0	0	0	0	6	6	0	S	0.0001998001998002
SE_LT2_03899_p.Val23Ala	SE_LT2_03899	L-threonine 3-dehydrogenase	0	0	0	1	1	1	1	1	1	0	0	0	0	0	0	6	6	0	C	0.0001998001998002

SE_LT2_03900_p.Ala18Val	SE_LT2_03900	glycine C-acetyltransferase	0	0	0	1	1	1	1	1	1	0	0	0	0	0	0	6	6	0	H	0.0001998001998002
SE_LT2_03904_p.Gly300Ser	SE_LT2_03904	O-antigen ligase	0	0	0	1	1	1	1	1	1	0	0	0	0	0	0	6	6	0	M	0.0001998001998002
SE_LT2_03904_p.Pro61Ser	SE_LT2_03904	O-antigen ligase	0	0	0	1	1	1	1	1	1	0	0	0	0	0	0	6	6	0	M	0.0001998001998002
SE_LT2_03907_p.Arg225His	SE_LT2_03907	heptose kinase	0	0	0	1	1	1	1	1	1	0	0	0	0	0	0	6	6	0	S	0.0001998001998002
SE_LT2_03908_p.Lys189Gln	SE_LT2_03908	lipopolysaccharide 1,2-glucosyltrans	0	0	0	1	1	1	1	1	1	0	0	0	0	0	0	6	6	0	M	0.0001998001998002
SE_LT2_03908_p.Val48Ile	SE_LT2_03908	lipopolysaccharide 1,2-glucosyltrans	0	0	0	1	1	1	1	1	1	0	0	0	0	0	0	6	6	0	M	0.0001998001998002
SE_LT2_03909_p.Ile327Val	SE_LT2_03909	lipopolysaccharide 1,3-galactosyltra	0	0	0	1	1	1	1	1	1	0	0	0	0	0	0	6	6	0	M	0.0001998001998002
SE_LT2_03909_p.Val129Ile	SE_LT2_03909	lipopolysaccharide 1,3-galactosyltra	0	0	0	1	1	1	1	1	1	0	0	0	0	0	0	6	6	0	M	0.0001998001998002
SE_LT2_03910_p.Ser245Arg	SE_LT2_03910	glycosyl transferase	0	0	0	1	1	1	1	1	1	0	0	0	0	0	0	6	6	0	M	0.0001998001998002
SE_LT2_03910_p.Val258Ile	SE_LT2_03910	glycosyl transferase	0	0	0	1	1	1	1	1	1	0	0	0	0	0	0	6	6	0	M	0.0001998001998002
SE_LT2_03913_p.Ala334Val	SE_LT2_03913	glucosyltransferase I RfaG	0	0	0	1	1	1	1	1	1	0	0	0	0	0	0	6	6	0	M	0.0001998001998002
SE_LT2_03936_p.Gln189His	SE_LT2_03936	ATP-dependent DNA helicase RecG	0	0	0	1	1	1	1	1	1	0	0	0	0	0	0	6	6	0	L	0.0001998001998002
SE_LT2_03936_p.His545Gln	SE_LT2_03936	ATP-dependent DNA helicase RecG	0	0	0	1	1	1	1	1	1	0	0	0	0	0	0	6	6	0	L	0.0001998001998002
SE_LT2_03939_p.Ile100Val	SE_LT2_03939	xanthine permease XanP	0	0	0	1	1	1	1	1	1	0	0	0	0	0	0	6	6	0	F	0.0001998001998002
SE_LT2_03941_p.Ile34Val	SE_LT2_03941	putative alpha-xylosidase	0	0	0	1	1	1	1	1	1	0	0	0	0	0	0	6	6	0	G	0.0001998001998002
SE_LT2_03950_p.Ser105Cys	SE_LT2_03950	autotransporter outer membrane be	0	0	0	1	1	1	1	1	1	0	0	0	0	0	0	6	6	0	MU	0.0001998001998002
SE_LT2_03954_p.Lys57Arg	SE_LT2_03954	hypothetical protein	0	0	0	1	1	1	1	1	1	0	0	0	0	0	0	6	6	0	S	0.0001998001998002
SE_LT2_03956_p.Glu170Asp	SE_LT2_03956	magnesium-translocating P-type AT	0	0	0	1	1	1	1	1	1	0	0	0	0	0	0	6	6	0	P	0.0001998001998002
SE_LT2_03956_p.Ser530Phe	SE_LT2_03956	magnesium-translocating P-type AT	0	0	0	1	1	1	1	1	1	0	0	0	0	0	0	6	6	0	P	0.0001998001998002
SE_LT2_03984_p.Asp185Asn	SE_LT2_03984	DNA-binding response regulator	0	0	0	1	1	1	1	1	1	0	0	0	0	0	0	6	6	0	K	0.0001998001998002
SE_LT2_03994_p.Ala191Glu	SE_LT2_03994	integral membrane protein	0	0	0	1	1	1	1	1	1	0	0	0	0	0	0	6	6	0	EG	0.0001998001998002
SE_LT2_03996_p.Ala351Val	SE_LT2_03996	hypothetical protein	0	0	0	1	1	1	1	1	1	0	0	0	0	0	0	6	6	0	G	0.0001998001998002
SE_LT2_03996_p.Leu212Pro	SE_LT2_03996	hypothetical protein	0	0	0	1	1	1	1	1	1	0	0	0	0	0	0	6	6	0	G	0.0001998001998002
SE_LT2_03996_p.Pro143Ser	SE_LT2_03996	hypothetical protein	0	0	0	1	1	1	1	1	1	0	0	0	0	0	0	6	6	0	G	0.0001998001998002
SE_LT2_03999_p.SerVal398AlaTh	SE_LT2_03999	D-serine ammonia-lyase	0	0	0	1	1	1	1	1	1	0	0	0	0	0	0	6	6	0	E	0.0001998001998002
SE_LT2_04008_p.Arg214Ser	SE_LT2_04008	hypothetical protein	0	0	0	1	1	1	1	1	1	0	0	0	0	0	0	6	6	0	U	0.0001998001998002
SE_LT2_04019_p.Pro476Thr	SE_LT2_04019	trimethylamine N-oxide reductase I	0	0	0	1	1	1	1	1	1	0	0	0	0	0	0	6	6	0	C	0.0001998001998002
SE_LT2_04023_p.Arg804Leu	SE_LT2_04023	TMAO reductase system sensor histi	0	0	0	1	1	1	1	1	1	0	0	0	0	0	0	6	6	0	T	0.0001998001998002
SE_LT2_04029_p.Asn148Ala	SE_LT2_04029	sugar-phosphatase	0	0	0	1	1	1	1	1	1	0	0	0	0	0	0	6	6	0	S	0.0001998001998002
SE_LT2_04029_p.His273Tyr	SE_LT2_04029	sugar-phosphatase	0	0	0	1	1	1	1	1	1	0	0	0	0	0	0	6	6	0	S	0.0001998001998002
SE_LT2_04052_p.Thr215Met	SE_LT2_04052	putative xanthine/uracil permeases	0	0	0	1	1	1	1	1	1	0	0	0	0	0	0	6	6	0	S	0.0001998001998002
SE_LT2_04062_p.Ala221Thr	SE_LT2_04062	SgrR family transcriptional regulator	0	0	0	1	1	1	1	1	0	0	0	0	0	0	0	5	5	0	K	0.00199800199800199
SE_LT2_04063_p.Leu183Met	SE_LT2_04063	glutamine--fructose-6-phosphate ar	0	0	0	1	1	1	1	1	1	0	0	0	0	0	0	6	6	0	M	0.0001998001998002
SE_LT2_04066_p.Leu100Phe	SE_LT2_04066	putative permease	0	0	0	1	1	1	1	1	1	0	0	0	0	0	0	6	6	0	S	0.0001998001998002
SE_LT2_04078_p.GlyAla111AspTl	SE_LT2_04078	FMN-binding protein MioC	0	0	0	1	1	1	1	1	1	0	0	0	0	0	0	6	6	0	C	0.0001998001998002
SE_LT2_04080_p.Thr132Ser	SE_LT2_04080	aspartate--ammonia ligase	0	0	0	1	1	1	1	1	1	0	0	0	0	0	0	6	6	0	F	0.0001998001998002
SE_LT2_04081_p.Met18Ile	SE_LT2_04081	putative inner membrane protein	0	0	0	1	1	1	1	1	1	0	0	0	0	0	0	6	6	0	S	0.0001998001998002
SE_LT2_04103_p.Arg190His	SE_LT2_04103	acetolactate synthase 2 catalytic sub	0	0	0	1	1	1	1	1	1	0	0	0	0	0	0	6	6	0	H	0.0001998001998002
SE_LT2_04106_p.Val60Ile	SE_LT2_04106	dihydroxy-acid dehydratase	0	0	0	1	1	1	1	1	1	0	0	0	0	0	0	6	6	0	E	0.0001998001998002
SE_LT2_04114_p.Asp464Asn	SE_LT2_04114	guanosine-5'-triphosphate,3'-diphos	0	0	0	1	1	1	1	1	1	0	0	0	0	0	0	6	6	0	F	0.0001998001998002
SE_LT2_04126_p.Ala47Val	SE_LT2_04126	dTDP-4-amino-4,6-dideoxygalactose	0	0	0	1	1	1	1	1	1	0	0	0	0	0	0	6	6	0	E	0.0001998001998002
SE_LT2_04126_p.Phe309Ser	SE_LT2_04126	dTDP-4-amino-4,6-dideoxygalactose	0	0	0	1	1	1	1	1	1	0	0	0	0	0	0	6	6	0	E	0.0001998001998002
SE_LT2_04129_p.Ser225Cys	SE_LT2_04129	enterobacterial common antigen po	0	0	0	1	1	1	1	1	1	0	0	0	0	0	0	6	6	0	S	0.0001998001998002
SE_LT2_04130_p.Ala65Thr	SE_LT2_04130	lipopolysaccharide N-acetylmannos	0	0	0	1	1	1	1	1	1	0	0	0	0	0	0	6	6	0	F	0.0001998001998002
SE_LT2_04131_p.Arg399Cys	SE_LT2_04131	amino acid permease	0	0	0	1	1	1	1	1	1	0	0	0	0	0	0	6	6	0	U	0.0001998001998002
SE_LT2_04140_p.Met278Ile	SE_LT2_04140	porphobilinogen deaminase	0	0	0	1	1	1	1	1	1	0	0	0	0	0	0	6	6	0	H	0.0001998001998002
SE_LT2_04169_p.Gln230Glu	SE_LT2_04169	putative arylsulfatase regulator	0	0	0	1	1	1	1	1	1	0	0	0	0	0	0	6	6	0	C	0.0001998001998002
SE_LT2_04180_p.Ala167Asp	SE_LT2_04180	hydrolase TatD	0	0	0	1	1	1	1	1	1	0	0	0	0	0	0	6	6	0	L	0.0001998001998002
SE_LT2_04187_p.AlaLeuGlu439V	SE_LT2_04187	3-hydroxyacyl-coA dehydrogenase	0	0	0	1	1	1	1	1	1	0	0	0	0	0	0	6	6	0	I	0.0001998001998002
SE_LT2_04187_p.Gln416Asp	SE_LT2_04187	3-hydroxyacyl-coA dehydrogenase	0	0	0	1	1	1	1	1	1	0	0	0	0	0	0	6	6	0	I	0.0001998001998002

SE_LT2_04187_p.Glu421Asp	SE_LT2_04187	3-hydroxyacyl-coA dehydrogenase	0	0	0	1	1	1	1	1	1	0	0	0	0	0	0	6	6	0	I	0.0001998001998002
SE_LT2_04187_p.Gly434Asn	SE_LT2_04187	3-hydroxyacyl-coA dehydrogenase	0	0	0	1	1	1	1	1	1	0	0	0	0	0	0	6	6	0	I	0.0001998001998002
SE_LT2_04187_p.Ser579Asn	SE_LT2_04187	3-hydroxyacyl-coA dehydrogenase	0	0	0	1	1	1	1	1	1	0	0	0	0	0	0	6	6	0	I	0.0001998001998002
SE_LT2_04198_p.Ala66Thr	SE_LT2_04198	molybdopterin-guanine dinucleotid	0	0	0	1	1	1	1	1	1	0	0	0	0	0	0	6	6	0	H	0.0001998001998002
SE_LT2_04209_p.Ala97Thr	SE_LT2_04209	GTPase-activating protein	0	0	0	1	1	1	1	1	1	0	0	0	0	0	0	6	6	0	S	0.0001998001998002
SE_LT2_04210_p.Asp102Asn	SE_LT2_04210	oxygen-independent coproporphyri	0	0	0	1	1	1	1	1	1	0	0	0	0	0	0	6	6	0	H	0.0001998001998002
SE_LT2_04226_p.Ala580Thr	SE_LT2_04226	alpha-glucosidase	0	0	0	1	1	1	1	1	1	0	0	0	0	0	0	6	6	0	G	0.0001998001998002
SE_LT2_04226_p.Asp230Ala	SE_LT2_04226	alpha-glucosidase	0	0	0	1	1	1	1	1	1	0	0	0	0	0	0	6	6	0	G	0.0001998001998002
SE_LT2_04226_p.Glu648Asp	SE_LT2_04226	alpha-glucosidase	0	0	0	1	1	1	1	1	1	0	0	0	0	0	0	6	6	0	G	0.0001998001998002
SE_LT2_04228_p.Lys80Asn	SE_LT2_04228	sugar isomerase	0	0	0	1	1	1	1	1	1	0	0	0	0	0	0	6	6	0	G	0.0001998001998002
SE_LT2_04229_p.Met141Ile	SE_LT2_04229	putative aldolase	0	0	0	1	1	1	1	1	1	0	0	0	0	0	0	6	6	0	G	0.0001998001998002
SE_LT2_04230_p.Pro66Ser	SE_LT2_04230	6-phosphogluconate dehydrogenase	0	0	0	1	1	1	1	1	1	0	0	0	0	0	0	6	6	0	I	0.0001998001998002
SE_LT2_04239_p.Asp56Asn	SE_LT2_04239	lipase	0	0	0	1	1	1	1	1	1	0	0	0	0	0	0	6	6	0	I	0.0001998001998002
SE_LT2_04239_p.Glu54Lys	SE_LT2_04239	lipase	0	0	0	1	1	1	1	1	1	0	0	0	0	0	0	6	6	0	I	0.0001998001998002
SE_LT2_04240_p.His32Tyr	SE_LT2_04240	Toxin HigB-2	0	0	0	1	1	1	1	1	1	0	0	0	0	0	0	6	6	0	S	0.0001998001998002
SE_LT2_04242_p.Ser298Thr	SE_LT2_04242	formate dehydrogenase accessory p	0	0	0	1	1	1	1	1	1	0	0	0	0	0	0	6	6	0	O	0.0001998001998002
SE_LT2_04250_p.Ala67_Thr77del	SE_LT2_04250	putative cytoplasmic protein	0	0	0	1	1	1	1	1	1	0	0	0	0	0	0	6	6	0	M	0.0001998001998002
SE_LT2_04256_p.Lys341Thr	SE_LT2_04256	putative iron-containing alcohol del	0	0	0	1	1	1	1	1	1	0	0	0	0	0	0	6	6	0	C	0.0001998001998002
SE_LT2_04258_p.Ala276Thr	SE_LT2_04258	L-rhamnose isomerase	0	0	0	1	1	1	1	1	1	0	0	0	0	0	0	6	6	0	G	0.0001998001998002
SE_LT2_04259_p.Val454Ile	SE_LT2_04259	rhamnulokinase	0	0	0	1	1	1	1	1	1	0	0	0	0	0	0	6	6	0	F	0.0001998001998002
SE_LT2_04262_p.Ala343Thr	SE_LT2_04262	rhamnose/proton symporter RhaT	0	0	0	1	1	1	1	1	1	0	0	0	0	0	0	6	6	0	U	0.0001998001998002
SE_LT2_04263_p.Asn154Lys	SE_LT2_04263	putative outer membrane protein	0	0	0	1	1	1	1	1	1	0	0	0	0	0	0	6	6	0	M	0.0001998001998002
SE_LT2_04269_p.Glu128Asp	SE_LT2_04269	hypothetical protein	0	0	0	1	1	1	1	1	1	0	0	0	0	0	0	6	6	0	S	0.0001998001998002
SE_LT2_04270_p.Lys455Arg	SE_LT2_04270	two-component system sensor histi	0	0	0	1	1	1	1	1	1	0	0	0	0	0	0	6	6	0	T	0.0001998001998002
SE_LT2_04282_p.Lys92Asn	SE_LT2_04282	hypothetical protein	0	0	0	1	1	1	1	1	1	0	0	0	0	0	0	6	6	0	S	0.0001998001998002
SE_LT2_04291_p.Thr234Met	SE_LT2_04291	epimerase	0	0	0	1	1	1	1	1	1	0	0	0	0	0	0	6	6	0	G	0.0001998001998002
SE_LT2_04295_p.Val48Ile	SE_LT2_04295	ferredoxin--NADP(+) reductase	0	0	0	1	1	1	1	1	1	0	0	0	0	0	0	6	6	0	C	0.0001998001998002
SE_LT2_04305_p.Gln135del	SE_LT2_04305	cell division protein FtsN	0	0	0	1	1	1	1	1	1	0	0	0	0	0	0	6	6	0	D	0.0001998001998002
SE_LT2_04307_p.Lys720Arg	SE_LT2_04307	factor Y	0	0	0	1	1	1	1	1	1	0	0	0	0	0	0	6	6	0	L	0.0001998001998002
SE_LT2_04331_p.Gly65Ala	SE_LT2_04331	Phosphoethanolamine transferase (	0	0	0	1	1	1	1	1	1	0	0	0	0	0	0	6	6	0	S	0.0001998001998002
SE_LT2_04352_p.Val21Ala	SE_LT2_04352	UDP-N-acetylenolpyruvoylglucosam	0	0	0	1	1	1	1	1	1	0	0	0	0	0	0	6	6	0	M	0.0001998001998002
SE_LT2_04352_p.Val52Ile	SE_LT2_04352	UDP-N-acetylenolpyruvoylglucosam	0	0	0	1	1	1	1	1	1	0	0	0	0	0	0	6	6	0	M	0.0001998001998002
SE_LT2_04353_p.Ala69Val	SE_LT2_04353	biotin--[acetyl-CoA-carboxylase] sy	0	0	0	1	1	1	1	1	1	0	0	0	0	0	0	6	6	0	H	0.0001998001998002
SE_LT2_04372_p.Ala25Thr	SE_LT2_04372	thiamine biosynthesis protein ThiH	0	0	0	1	1	1	1	1	1	0	0	0	0	0	0	6	6	0	C	0.0001998001998002
SE_LT2_04372_p.Ala272Thr	SE_LT2_04372	thiamine biosynthesis protein ThiH	0	0	0	1	0	1	1	1	1	0	0	0	0	0	0	5	5	0	C	0.00199800199800199
SE_LT2_04372_p.AlaHis35ProGln	SE_LT2_04372	thiamine biosynthesis protein ThiH	0	0	0	1	1	1	0	1	1	0	0	0	0	0	0	5	5	0	C	0.00199800199800199
SE_LT2_04373_p.Glu28Lys	SE_LT2_04373	thiazole synthase	0	0	0	1	1	1	1	1	1	0	0	0	0	0	0	6	6	0	H	0.0001998001998002
SE_LT2_04373_p.His189Gln	SE_LT2_04373	thiazole synthase	0	0	0	1	1	1	1	1	1	0	0	0	0	0	0	6	6	0	H	0.0001998001998002
SE_LT2_04373_p.Lys164Arg	SE_LT2_04373	thiazole synthase	0	0	0	1	1	1	1	1	1	0	0	0	0	0	0	6	6	0	H	0.0001998001998002
SE_LT2_04373_p.Met215Arg	SE_LT2_04373	thiazole synthase	0	0	0	0	1	1	1	1	1	0	0	0	0	0	0	5	5	0	H	0.00199800199800199
SE_LT2_04376_p.Asp111Asn	SE_LT2_04376	thiamine-phosphate diphosphoryla	0	0	0	1	1	1	1	1	1	0	0	0	0	0	0	6	6	0	H	0.0001998001998002
SE_LT2_04376_p.Ser31Val	SE_LT2_04376	thiamine-phosphate diphosphoryla	0	0	0	1	1	1	1	1	1	0	0	0	0	0	0	6	6	0	H	0.0001998001998002
SE_LT2_04377_p.Ala631Val	SE_LT2_04377	phosphomethylpyrimidine synthase	0	0	0	1	1	1	1	1	1	0	0	0	0	0	0	6	6	0	H	0.0001998001998002
SE_LT2_04382_p.Ala89Val	SE_LT2_04382	endonuclease V	0	0	0	1	1	1	1	1	1	0	0	0	0	0	0	6	6	0	L	0.0001998001998002
SE_LT2_04383_p.Gln152Leu	SE_LT2_04383	putative protein	0	0	0	1	1	1	1	1	1	0	0	0	0	0	0	6	6	0	S	0.0001998001998002
SE_LT2_04388_p.Ala77Val	SE_LT2_04388	two-component system response re	0	0	0	1	1	1	1	1	1	0	0	0	0	0	0	6	6	0	T	0.0001998001998002
SE_LT2_04388_p.Tyr395Cys	SE_LT2_04388	two-component system response re	0	0	0	1	1	1	1	0	1	0	0	0	0	0	0	5	5	0	T	0.00199800199800199
SE_LT2_04389_p.Asp26Gly	SE_LT2_04389	phosphoribosylamine--glycine ligas	0	0	0	1	1	1	1	1	1	0	0	0	0	0	0	6	6	0	F	0.0001998001998002
SE_LT2_04396_p.Met120Ile	SE_LT2_04396	putative acetyltransferase	0	0	0	1	1	1	1	1	1	0	0	0	0	0	0	6	6	0	K	0.0001998001998002
SE_LT2_04398_p.Ala264Glu	SE_LT2_04398	malate synthase A	0	0	0	1	1	1	1	1	1	0	0	0	0	0	0	6	6	0	H	0.0001998001998002

SE_LT2_04398_p.Arg509His	SE_LT2_04398	malate synthase A	0	0	0	1	1	1	1	1	1	0	0	0	0	0	0	6	6	0	H	0.0001998001998002
SE_LT2_04399_p.Tyr309Ser	SE_LT2_04399	isocitrate lyase	0	0	0	1	1	1	1	1	1	0	0	0	0	0	0	6	6	0	C	0.0001998001998002
SE_LT2_04403_p.Asp633Glu	SE_LT2_04403	B12-dependent homocysteine-N5-m	0	0	0	1	1	1	1	1	1	0	0	0	0	0	0	6	6	0	H	0.0001998001998002
SE_LT2_04405_p.Ala161Thr	SE_LT2_04405	dipeptidase E	0	0	0	1	1	1	1	1	1	0	0	0	0	0	0	6	6	0	E	0.0001998001998002
SE_LT2_04418_p.Asp86Glu	SE_LT2_04418	glycosyltransferase	0	0	0	1	1	1	1	1	1	0	0	0	0	0	0	6	6	0	M	0.0001998001998002
SE_LT2_04418_p.His82Tyr	SE_LT2_04418	glycosyltransferase	0	0	0	1	1	1	1	1	1	0	0	0	0	0	0	6	6	0	M	0.0001998001998002
SE_LT2_04418_p.Val116Gly	SE_LT2_04418	glycosyltransferase	0	0	0	1	1	1	1	1	1	0	0	0	0	0	0	6	6	0	M	0.0001998001998002
SE_LT2_04434_p.Gly251Ser	SE_LT2_04434	glucose-6-phosphate isomerase	0	0	0	1	1	1	1	1	1	0	0	0	0	0	0	6	6	0	G	0.0001998001998002
SE_LT2_04440_p.Leu82Phe	SE_LT2_04440	phosphate-starvation-inducible pro	0	0	0	1	1	1	1	1	1	0	0	0	0	0	0	6	6	0	S	0.0001998001998002
SE_LT2_04456_p.Leu193Ile	SE_LT2_04456	conjugal transfer protein	0	0	0	1	1	1	1	1	1	0	0	0	0	0	0	6	6	0	S	0.0001998001998002
SE_LT2_04456_p.Leu335Gln	SE_LT2_04456	conjugal transfer protein	0	0	0	1	1	1	1	1	1	0	0	0	0	0	0	6	6	0	S	0.0001998001998002
SE_LT2_04461_p.Gln183Arg	SE_LT2_04461	alanine racemase	0	0	0	1	1	1	1	1	1	0	0	0	0	0	0	6	6	0	E	0.0001998001998002
SE_LT2_04461_p.His146Arg	SE_LT2_04461	alanine racemase	0	0	0	1	1	1	1	1	1	0	0	0	0	0	0	6	6	0	E	0.0001998001998002
SE_LT2_04462_p.Ala383Thr	SE_LT2_04462	aromatic amino acid aminotransfera	0	0	0	1	1	1	1	1	1	0	0	0	0	0	0	6	6	0	E	0.0001998001998002
SE_LT2_04462_p.Asp87Asn	SE_LT2_04462	aromatic amino acid aminotransfera	0	0	0	1	1	1	1	1	1	0	0	0	0	0	0	6	6	0	E	0.0001998001998002
SE_LT2_04462_p.Thr48Pro	SE_LT2_04462	aromatic amino acid aminotransfera	0	0	0	1	1	1	1	1	1	0	0	0	0	0	0	6	6	0	E	0.0001998001998002
SE_LT2_04476_p.Asp116Glu	SE_LT2_04476	antibiotic ABC transporter ATP-bind	0	0	0	1	1	1	1	1	1	0	0	0	0	0	0	6	6	0	V	0.0001998001998002
SE_LT2_04476_p.Glu87Gly	SE_LT2_04476	antibiotic ABC transporter ATP-bind	0	0	0	1	1	1	1	1	1	0	0	0	0	0	0	6	6	0	V	0.0001998001998002
SE_LT2_04477_p.Val78Ala	SE_LT2_04477	hypothetical protein	0	0	0	1	1	1	1	1	1	0	0	0	0	0	0	6	6	0	S	0.0001998001998002
SE_LT2_04486_p.Leu52Phe	SE_LT2_04486	murein hydrolase regulator LrgA	0	0	0	1	1	1	1	1	1	0	0	0	0	0	0	6	6	0	S	0.0001998001998002
SE_LT2_04503_p.Pro2Ser	SE_LT2_04503	putative regulator in phn operon	0	0	0	1	1	1	1	1	1	0	0	0	0	0	0	6	6	0	K	0.0001998001998002
SE_LT2_04514_p.Ala541Thr	SE_LT2_04514	arginine decarboxylase	0	0	0	1	1	1	1	1	1	0	0	0	0	0	0	6	6	0	E	0.0001998001998002
SE_LT2_04517_p.Gly102Glu	SE_LT2_04517	melibiose:sodium transporter MelB	0	0	0	1	1	1	1	1	1	0	0	0	0	0	0	6	6	0	G	0.0001998001998002
SE_LT2_04527_p.Asp305Asn	SE_LT2_04527	hypothetical protein	0	0	0	1	1	1	1	1	1	0	0	0	0	0	0	6	6	0	S	0.0001998001998002
SE_LT2_04534_p.Val10Ala	SE_LT2_04534	AraC family transcriptional regulato	0	0	0	1	1	1	1	1	1	0	0	0	0	0	0	6	6	0	K	0.0001998001998002
SE_LT2_04551_p.Gly257Glu	SE_LT2_04551	hypothetical protein	0	0	0	1	1	1	1	1	1	0	0	0	0	0	0	6	6	0	S	0.0001998001998002
SE_LT2_04555_p.His110Asn	SE_LT2_04555	LuxR family transcriptional regulato	0	0	0	1	1	1	1	1	1	0	0	0	0	0	0	6	6	0	K	0.0001998001998002
SE_LT2_04574_p.Ser320Asn	SE_LT2_04574	putative Fe-S protein	0	0	0	1	1	1	1	1	1	0	0	0	0	0	0	6	6	0	C	0.0001998001998002
SE_LT2_04576_p.Asp16Gly	SE_LT2_04576	tRNA (adenosine(37)-N6)-threonylc	0	0	0	1	1	1	1	1	1	0	0	0	0	0	0	6	6	0	S	0.0001998001998002
SE_LT2_04577_p.Gly422Ser	SE_LT2_04577	N-acetylmuramoyl-L-alanine amidas	0	0	0	1	1	1	1	1	1	0	0	0	0	0	0	6	6	0	M	0.0001998001998002
SE_LT2_04577_p.Pro131Gln	SE_LT2_04577	N-acetylmuramoyl-L-alanine amidas	0	0	0	1	1	1	1	1	1	0	0	0	0	0	0	6	6	0	M	0.0001998001998002
SE_LT2_04578_p.Gly408Ser	SE_LT2_04578	DNA mismatch repair protein MutL	0	0	0	1	1	1	1	1	1	0	0	0	0	0	0	6	6	0	L	0.0001998001998002
SE_LT2_04581_p.Ser85Asn	SE_LT2_04581	GTPase HflX	0	0	0	1	1	1	1	1	1	0	0	0	0	0	0	6	6	0	S	0.0001998001998002
SE_LT2_04588_p.Met202Ile	SE_LT2_04588	23S rRNA (guanosine(2251)-2'-O)-me	0	0	0	1	1	1	1	1	1	0	0	0	0	0	0	6	6	0	J	0.0001998001998002
SE_LT2_04594_p.Asn64Lys	SE_LT2_04594	hypothetical protein	0	0	0	1	1	1	1	1	1	0	0	0	0	0	0	6	6	0	S	0.0001998001998002
SE_LT2_04598_p.Ala10Val	SE_LT2_04598	biofilm stress/motility protein A	0	0	0	1	1	1	1	1	1	0	0	0	0	0	0	6	6	0	S	0.0001998001998002
SE_LT2_04598_p.Val87Ile	SE_LT2_04598	biofilm stress/motility protein A	0	0	0	1	1	1	1	1	1	0	0	0	0	0	0	6	6	0	S	0.0001998001998002
SE_LT2_04599_p.Ala160Val	SE_LT2_04599	esterase	0	0	0	1	0	1	1	1	1	0	0	0	0	0	0	5	5	0	S	0.00199800199800199
SE_LT2_04599_p.Lys22Arg	SE_LT2_04599	esterase	0	0	0	1	1	1	1	1	1	0	0	0	0	0	0	6	6	0	S	0.0001998001998002
SE_LT2_04614_p.Ser140Asn	SE_LT2_04614	EamA family transporter	0	0	0	1	1	1	1	1	1	0	0	0	0	0	0	6	6	0	EG	0.0001998001998002
SE_LT2_04616_p.Thr110Met	SE_LT2_04616	FKBP-type 22KD peptidyl-prolyl cis-t	0	0	0	1	1	1	1	1	1	0	0	0	0	0	0	6	6	0	G	0.0001998001998002
SE_LT2_04618_p.Gln24Arg	SE_LT2_04618	iron-sulfur cluster repair di-iron pro	0	0	0	1	1	1	1	1	1	0	0	0	0	0	0	6	6	0	D	0.0001998001998002
SE_LT2_04622_p.Ala569Ser	SE_LT2_04622	2',3'-cyclic-nucleotide 2'-phosphodie	0	0	0	1	1	1	1	1	1	0	0	0	0	0	0	6	6	0	F	0.0001998001998002
SE_LT2_04628_p.Asn147Ser	SE_LT2_04628	peptide-methionine (S)-S-oxide red	0	0	0	1	1	1	1	1	1	0	0	0	0	0	0	6	6	0	O	0.0001998001998002
SE_LT2_04629_p.Lys146Arg	SE_LT2_04629	Translocation and assembly module	0	0	0	1	1	1	1	1	1	0	0	0	0	0	0	6	6	0	M	0.0001998001998002
SE_LT2_04630_p.Arg585Leu	SE_LT2_04630	translocation/assembly module Tam	0	0	0	1	1	1	1	1	1	0	0	0	0	0	0	6	6	0	S	0.0001998001998002
SE_LT2_04630_p.Lys146Asn	SE_LT2_04630	translocation/assembly module Tam	0	0	0	1	1	1	1	1	1	0	0	0	0	0	0	6	6	0	S	0.0001998001998002
SE_LT2_04630_p.Met1009Arg	SE_LT2_04630	translocation/assembly module Tam	0	0	0	0	1	1	1	1	1	0	0	0	0	0	0	5	5	0	S	0.00199800199800199
SE_LT2_04656_p.Glu20_Asp21de	SE_LT2_04656	hypothetical protein	0	0	0	1	1	1	1	1	1	0	0	0	0	0	0	6	6	0	S	0.0001998001998002
SE_LT2_04664_p.Asp220Glu	SE_LT2_04664	putative dihydroorotase	0	0	0	1	1	1	1	1	1	0	0	0	0	0	0	6	6	0	S	0.0001998001998002

SE_LT2_04665_p.Ile33Val	SE_LT2_04665	L-seryl-tRNA selenium transferase	0	0	0	1	1	1	1	1	1	0	0	0	0	0	0	6	6	0	E	0.0001998001998002
SE_LT2_04666_p.Gly19Ser	SE_LT2_04666	2-dehydro-3-deoxyphosphooctonat	0	0	0	1	1	1	1	1	1	0	0	0	0	0	0	6	6	0	S	0.0001998001998002
SE_LT2_04667_p.Val119Ile	SE_LT2_04667	transcription antiterminator BglG	0	0	0	1	1	1	1	1	1	0	0	0	0	0	0	6	6	0	K	0.0001998001998002
SE_LT2_04667_p.Val189Leu	SE_LT2_04667	transcription antiterminator BglG	0	0	0	1	1	1	1	1	1	0	0	0	0	0	0	6	6	0	K	0.0001998001998002
SE_LT2_04678_p.Arg75Cys	SE_LT2_04678	magnesium-translocating P-type AT	0	0	0	1	1	1	1	1	1	0	0	0	0	0	0	6	6	0	P	0.0001998001998002
SE_LT2_04678_p.Ser604Asn	SE_LT2_04678	magnesium-translocating P-type AT	0	0	0	1	1	1	1	1	1	0	0	0	0	0	0	6	6	0	P	0.0001998001998002
SE_LT2_04687_p.Arg254Cys	SE_LT2_04687	putative carbamate kinase	0	0	0	1	1	1	1	1	1	0	0	0	0	0	0	6	6	0	E	0.0001998001998002
SE_LT2_04688_p.Val78Met	SE_LT2_04688	putative arginine deiminase	0	0	0	1	1	1	1	1	1	0	0	0	0	0	0	6	6	0	E	0.0001998001998002
SE_LT2_04689_p.Ala81Val	SE_LT2_04689	toxin-antitoxin biofilm protein TabA	0	0	0	1	1	1	1	1	1	0	0	0	0	0	0	6	6	0	G	0.0001998001998002
SE_LT2_04695_p.Ala66Thr	SE_LT2_04695	Inner membrane protein YjgN	0	0	0	1	1	1	1	1	1	0	0	0	0	0	0	6	6	0	S	0.0001998001998002
SE_LT2_04696_p.Ala465Gln	SE_LT2_04696	valine--tRNA ligase	0	0	0	1	1	1	1	1	1	0	0	0	0	0	0	6	6	0	J	0.0001998001998002
SE_LT2_04696_p.Asp452Glu	SE_LT2_04696	valine--tRNA ligase	0	0	0	1	1	1	1	1	1	0	0	0	0	0	0	6	6	0	J	0.0001998001998002
SE_LT2_04696_p.Ser730Ala	SE_LT2_04696	valine--tRNA ligase	0	0	0	1	1	1	1	1	1	0	0	0	0	0	0	6	6	0	J	0.0001998001998002
SE_LT2_04696_p.ThrGlyGlySer72	SE_LT2_04696	valine--tRNA ligase	0	0	0	1	1	1	1	1	1	0	0	0	0	0	0	6	6	0	J	0.0001998001998002
SE_LT2_04705_p.Val261Met	SE_LT2_04705	L-idonate 5-dehydrogenase	0	0	0	1	1	1	1	1	1	0	0	0	0	0	0	6	6	0	E	0.0001998001998002
SE_LT2_04723_p.Phe8Ser	SE_LT2_04723	putative SAM-dependent methyltra	0	0	0	1	1	1	1	1	1	0	0	0	0	0	0	6	6	0	HJ	0.0001998001998002
SE_LT2_04737_p.Asp294Asn	SE_LT2_04737	putative LysR family transcriptional	0	0	0	1	1	1	1	1	1	0	0	0	0	0	0	6	6	0	K	0.0001998001998002
SE_LT2_04738_p.Ser236Tyr	SE_LT2_04738	beta-aspartyl-peptidase	0	0	0	1	1	1	1	1	1	0	0	0	0	0	0	6	6	0	E	0.0001998001998002
SE_LT2_04745_p.Glu336Gly	SE_LT2_04745	succinate-semialdehyde dehydroge	0	0	0	1	1	1	1	1	1	0	0	0	0	0	0	6	6	0	C	0.0001998001998002
SE_LT2_04745_p.Lys333Asn	SE_LT2_04745	succinate-semialdehyde dehydroge	0	0	0	1	1	1	1	1	1	0	0	0	0	0	0	6	6	0	C	0.0001998001998002
SE_LT2_04756_p.Glu267Ala	SE_LT2_04756	putative cobalamin synthesis protei	0	0	0	1	1	1	1	1	1	0	0	0	0	0	0	6	6	0	S	0.0001998001998002
SE_LT2_04767_p.Ser735Ala	SE_LT2_04767	phosphoglycerol transferase I	0	0	0	1	1	1	1	1	1	0	0	0	0	0	0	6	6	0	M	0.0001998001998002
SE_LT2_04770_p.Asp9Asn	SE_LT2_04770	primosomal protein DnaI	0	0	0	1	1	1	1	1	1	0	0	0	0	0	0	6	6	0	J	0.0001998001998002
SE_LT2_04773_p.Cys93Ser	SE_LT2_04773	putative LuxR/UhpA family transcrip	0	0	0	1	1	1	1	1	1	0	0	0	0	0	0	6	6	0	K	0.0001998001998002
SE_LT2_04786_p.Ala477Thr	SE_LT2_04786	peptide chain release factor 3	0	0	0	1	1	1	1	1	1	0	0	0	0	0	0	6	6	0	J	0.0001998001998002
SE_LT2_04787_p.Glu130Asp	SE_LT2_04787	molecular chaperone OsmY	0	0	0	1	1	1	1	1	1	0	0	0	0	0	0	6	6	0	S	0.0001998001998002
SE_LT2_04794_p.Ala429Val	SE_LT2_04794	thymidine phosphorylase	0	0	0	1	1	1	1	1	1	0	0	0	0	0	0	6	6	0	F	0.0001998001998002
SE_LT2_04802_p.Ala30Thr	SE_LT2_04802	lipoate--protein ligase	0	0	0	1	1	1	1	1	1	0	0	0	0	0	0	6	6	0	H	0.0001998001998002
SE_LT2_04802_p.Ile333Met	SE_LT2_04802	lipoate--protein ligase	0	0	0	1	1	1	1	1	1	0	0	0	0	0	0	6	6	0	H	0.0001998001998002
SE_LT2_04805_p.His29Gln	SE_LT2_04805	DNA repair protein RadA	0	0	0	1	1	1	1	1	1	0	0	0	0	0	0	6	6	0	O	0.0001998001998002
SE_LT2_04815_p.Lys42Asn	SE_LT2_04815	two-component system response re	0	0	0	1	1	1	1	1	1	0	0	0	0	0	0	6	6	0	K	0.0001998001998002
SE_LT2_04816_p.Pro327Ser	SE_LT2_04816	two-component system sensor histi	0	0	0	1	1	1	1	1	1	0	0	0	0	0	0	6	6	0	T	0.0001998001998002
SE_LT2_04817_p.Ala387Val	SE_LT2_04817	cell envelope integrity protein CreD	0	0	0	1	1	1	1	1	1	0	0	0	0	0	0	6	6	0	V	0.0001998001998002
SE_LT2_04819_p.Asn105His	SE_LT2_04819	fimbrial protein SthD	0	0	0	1	1	1	1	1	1	0	0	0	0	0	0	6	6	0	NU	0.0001998001998002
SE_LT2_04821_p.Ser214Asn	SE_LT2_04821	fimbrial assembly chaperone SthB	0	0	0	1	1	1	1	1	1	0	0	0	0	0	0	6	6	0	M	0.0001998001998002
SE_LT2_04824_p.Ser19Pro	SE_LT2_04824	hypothetical protein	0	0	0	1	1	1	1	1	1	0	0	0	0	0	0	6	6	0	-	0.0001998001998002

Figure S19 - MccE492 Core genome GWAS data for *S. enterica* strains. All of the SNPs significantly associated with MccE492 resistance phenotypes in the core genomes of the tested *S. enterica* strains. Represented are the SNPs, their loci and gene product as predicted in the *S. enterica* LT2 reference genome for each tested strain. The "SUM", "SUM_R", "SUM_S" are the total number of strains, the number of resistant strains, the number of susceptible strains, respectively, that have the corresponding SNP.

Gene	_C643_S55	_AAC1797_S45	_AAC1760_S53	_C683_S47	_C659_S6	_C660_S14	_C651_S54	_C650_S48	_C664_S22	_LT2	_SL1344_S5	_AAC1795_S29	_AAC1799_S21	_ATCC6962_S38	_C493_S56	_AAC1791_S37	SU	SUM	SUM	COG_category	Naive_p
36396_yfkN	0	0	0	0	1	1	1	1	1	0	0	0	0	0	0	0	5	5	0	F	0.001998001998001998 4
36397_hypothetical_protein	0	0	0	0	1	1	1	1	1	0	0	0	0	0	0	0	5	5	0	F	0.0019980019980019984
36691_dhfrI	0	0	0	0	1	1	1	1	1	0	0	0	0	0	0	0	5	5	0	H	0.0019980019980019984
36695_hypothetical_protein	0	0	0	0	1	1	1	1	1	0	0	0	0	0	0	0	5	5	0	M	0.0019980019980019984
36696_bla_1	0	0	0	1	1	1	1	1	1	0	0	0	0	0	0	0	6	6	0	V	0.0001998001998002
36697_hypothetical_protein	0	0	0	0	1	1	1	1	1	0	0	0	0	0	0	0	5	5	0	K	0.0019980019980019984
36698_hypothetical_protein	0	0	0	0	1	1	1	1	1	0	0	0	0	0	0	0	5	5	0	-	0.0019980019980019984
36699_Tn3_family_transposa..	0	0	0	0	1	1	1	1	1	0	0	0	0	0	0	0	5	5	0	L	0.0019980019980019984
36700_hypothetical_protein	0	0	0	0	1	1	1	1	1	0	0	0	0	0	0	0	5	5	0	S	0.0019980019980019984
36701_merR	0	0	0	0	1	1	1	1	1	0	0	0	0	0	0	0	5	5	0	K	0.0019980019980019984
36702_hypothetical_protein	0	0	0	0	1	1	1	1	1	0	0	0	0	0	0	0	5	5	0	-	0.0019980019980019984
36703_IS5_family_transposa..	0	0	0	0	1	1	1	1	1	0	0	0	0	0	0	0	5	5	0	L	0.0019980019980019984
36704_fieF_2	0	0	0	0	1	1	1	1	1	0	0	0	0	0	0	0	5	5	0	U	0.0019980019980019984
36705_btuB_1	0	0	0	1	1	1	1	1	1	0	0	0	0	0	0	0	6	6	0	P	0.0001998001998002
36706_cueR_2	0	0	0	1	1	1	1	1	1	0	0	0	0	0	0	0	6	6	0	K	0.0001998001998002
36707_hypothetical_protein	0	0	0	1	1	1	1	1	1	0	0	0	0	0	0	0	6	6	0	P	0.0001998001998002
36708_lspA_2	0	0	0	1	1	1	1	1	1	0	0	0	0	0	0	0	6	6	0	MU	0.0001998001998002
36709_ISL3_family_transpos..	0	0	0	1	1	1	1	1	1	0	0	0	0	0	0	0	6	6	0	-	0.0001998001998002
36710_hypothetical_protein	0	0	0	1	1	1	1	1	1	0	0	0	0	0	0	0	6	6	0	L	0.0001998001998002
36723_mcr-9.1	0	0	0	1	1	1	1	1	1	0	0	0	0	0	0	0	6	6	0	S	0.0001998001998002
36728_hypothetical_protein	0	0	0	1	1	1	1	1	1	0	0	0	0	0	0	0	6	6	0	P	0.0001998001998002
36729_rcnR_2	0	0	0	1	1	1	1	1	1	0	0	0	0	0	0	0	6	6	0	S	0.0001998001998002
36730_hypothetical_protein	0	0	0	1	1	1	1	1	1	0	0	0	0	0	0	0	6	6	0	-	0.0001998001998002
36731_hypothetical_protein	0	0	0	1	1	1	1	1	1	0	0	0	0	0	0	0	6	6	0	L	0.0001998001998002
36732_smc	0	0	0	1	1	1	1	1	1	0	0	0	0	0	0	0	6	6	0	-	0.0001998001998002
36733_hypothetical_protein	0	0	0	1	1	1	1	1	1	0	0	0	0	0	0	0	6	6	0	-	0.0001998001998002
36734_hypothetical_protein	0	0	0	1	1	1	1	1	1	0	0	0	0	0	0	0	6	6	0	S	0.0001998001998002
36735_hypothetical_protein	0	0	0	1	1	1	1	1	1	0	0	0	0	0	0	0	6	6	0	-	0.0001998001998002
36736_dcm_2	0	0	0	1	1	1	1	1	1	0	0	0	0	0	0	0	6	6	0	H	0.0001998001998002
36737_hypothetical_protein	0	0	0	1	1	1	1	1	1	0	0	0	0	0	0	0	6	6	0	L	0.0001998001998002
36738_hypothetical_protein	0	0	0	1	1	1	1	1	1	0	0	0	0	0	0	0	6	6	0	-	0.0001998001998002
36739_hypothetical_protein	0	0	0	1	1	1	1	1	1	0	0	0	0	0	0	0	6	6	0	-	0.0001998001998002
36740_hypothetical_protein	0	0	0	1	1	1	1	1	1	0	0	0	0	0	0	0	6	6	0	-	0.0001998001998002
36741_hypothetical_protein	0	0	0	1	1	1	1	1	1	0	0	0	0	0	0	0	6	6	0	-	0.0001998001998002

36742_hypothetical_protein	0	0	0	1	1	1	1	1	1	0	0	0	0	0	0	0	6	6	0	-	0.0001998001998002
36743_hypothetical_protein	0	0	0	1	1	1	1	1	1	0	0	0	0	0	0	0	6	6	0	-	0.0001998001998002
36744_hypothetical_protein	0	0	0	1	1	1	1	1	1	0	0	0	0	0	0	0	6	6	0	-	0.0001998001998002
36745_hypothetical_protein	0	0	0	1	1	1	1	1	1	0	0	0	0	0	0	0	6	6	0	P	0.0001998001998002
36746_hypothetical_protein	0	0	0	1	1	1	1	1	1	0	0	0	0	0	0	0	6	6	0	T	0.0001998001998002
36747_arsC	0	0	0	1	1	1	1	1	1	0	0	0	0	0	0	0	6	6	0	P	0.0001998001998002
36748_arsB	0	0	0	1	1	1	1	1	1	0	0	0	0	0	0	0	6	6	0	P	0.0001998001998002
36749_hypothetical_protein	0	0	0	1	1	1	1	1	1	0	0	0	0	0	0	0	6	6	0	K	0.0001998001998002
36750_arsH	0	0	0	1	1	1	1	1	1	0	0	0	0	0	0	0	6	6	0	S	0.0001998001998002
36751_ISNCY_family_transpo..	0	0	0	1	1	1	1	1	1	0	0	0	0	0	0	0	6	6	0	L	0.0001998001998002
36752_hypothetical_protein	0	0	0	0	1	1	1	1	1	0	0	0	0	0	0	0	5	5	0	-	0.0019980019980019984
36753_hypothetical_protein	0	0	0	0	1	1	1	1	1	0	0	0	0	0	0	0	5	5	0	-	0.0019980019980019984
36754_hypothetical_protein	0	0	0	0	1	1	1	1	1	0	0	0	0	0	0	0	5	5	0	S	0.0019980019980019984
36755_hypothetical_protein	0	0	0	0	1	1	1	1	1	0	0	0	0	0	0	0	5	5	0	S	0.0019980019980019984
36756_ISNCY_family_transpo..	0	0	0	0	1	1	1	1	1	0	0	0	0	0	0	0	5	5	0	L	0.0019980019980019984
36757_hypothetical_protein	0	0	0	1	1	1	1	1	1	0	0	0	0	0	0	0	6	6	0	-	0.0001998001998002
36758_hypothetical_protein	0	0	0	1	1	1	1	1	1	0	0	0	0	0	0	0	6	6	0	-	0.0001998001998002
36759_hypothetical_protein	0	0	0	1	1	1	1	1	1	0	0	0	0	0	0	0	6	6	0	-	0.0001998001998002
36760_hypothetical_protein	0	0	0	1	1	1	1	1	1	0	0	0	0	0	0	0	6	6	0	-	0.0001998001998002
36761_hypothetical_protein	0	0	0	1	1	1	1	1	1	0	0	0	0	0	0	0	6	6	0	-	0.0001998001998002
36762_hypothetical_protein	0	0	0	1	1	1	1	1	1	0	0	0	0	0	0	0	6	6	0	-	0.0001998001998002
36763_IS5_family_transposa..	0	0	0	1	1	1	1	1	1	0	0	0	0	0	0	0	6	6	0	L	0.0001998001998002
36764_hypothetical_protein	0	0	0	1	1	1	1	1	1	0	0	0	0	0	0	0	6	6	0	-	0.0001998001998002
36765_hypothetical_protein	0	0	0	1	1	1	1	1	1	0	0	0	0	0	0	0	6	6	0	-	0.0001998001998002
36766_Tn3_family_transposa..	0	0	0	1	1	1	1	1	1	0	0	0	0	0	0	0	6	6	0	L	0.0001998001998002
36767_Tn3_family_transposa..	0	0	0	1	1	1	1	1	1	0	0	0	0	0	0	0	6	6	0	L	0.0001998001998002
36770_hypothetical_protein	0	0	0	0	1	1	1	1	1	0	0	0	0	0	0	0	5	5	0	L	0.0019980019980019984
36771_hypothetical_protein	0	0	0	0	1	1	1	1	1	0	0	0	0	0	0	0	5	5	0	-	0.0019980019980019984
37135_hypothetical_protein	0	0	0	1	1	1	1	1	1	0	0	0	0	0	0	0	6	6	0	-	0.0001998001998002
37136_hypothetical_protein	0	0	0	1	1	1	1	1	1	0	0	0	0	0	0	0	6	6	0	S	0.0001998001998002
37142_yjgH	0	0	0	1	1	1	1	1	1	0	0	0	0	0	0	0	6	6	0	J	0.0001998001998002
37194_dmsB_1	0	0	0	1	1	1	1	1	1	0	0	0	0	0	0	0	6	6	0	C	0.0001998001998002
37419_hypothetical_protein	0	0	0	0	1	1	1	1	1	0	0	0	0	0	0	0	5	5	0	K	0.0019980019980019984
37420_bluR	0	0	0	0	1	1	1	1	1	0	0	0	0	0	0	0	5	5	0	K	0.0019980019980019984
37651_hypothetical_protein	0	0	0	1	1	1	1	1	1	0	0	0	0	0	0	0	6	6	0	S	0.0001998001998002
37652_hypothetical_protein	0	0	0	1	1	1	1	1	1	0	0	0	0	0	0	0	6	6	0	S	0.0001998001998002
37659_hypothetical_protein	0	0	0	1	1	1	1	1	1	0	0	0	0	0	0	0	6	6	0	-	0.0001998001998002
37660_hypothetical_protein	0	0	0	1	1	1	1	1	1	0	0	0	0	0	0	0	6	6	0	-	0.0001998001998002
37661_hypothetical_protein	0	0	0	1	1	1	1	1	1	0	0	0	0	0	0	0	6	6	0	-	0.0001998001998002
37663_hypothetical_protein	0	0	0	1	1	1	1	1	1	0	0	0	0	0	0	0	6	6	0	S	0.0001998001998002
37667_hypothetical_protein	0	0	0	1	1	1	1	1	1	0	0	0	0	0	0	0	6	6	0	-	0.0001998001998002
37668_hypothetical_protein	0	0	0	1	1	1	1	1	1	0	0	0	0	0	0	0	6	6	0	S	0.0001998001998002
37780_hypothetical_protein	0	0	0	1	1	1	1	1	1	0	0	0	0	0	0	0	6	6	0	-	0.0001998001998002
37853_ushA_1	0	0	0	1	1	1	1	1	1	0	0	0	0	0	0	0	6	6	0	F	0.0001998001998002
37854_ushA_2	0	0	0	1	1	1	1	1	1	0	0	0	0	0	0	0	6	6	0	F	0.0001998001998002
37998_hypothetical_protein	0	0	0	1	1	1	1	1	1	0	0	0	0	0	0	0	6	6	0	K	0.0001998001998002
37999_hypothetical_protein	0	0	0	1	1	1	1	1	1	0	0	0	0	0	0	0	6	6	0	K	0.0001998001998002
38001_hypothetical_protein	0	0	0	1	1	1	1	1	1	0	0	0	0	0	0	0	6	6	0	-	0.0001998001998002
38002_hypothetical_protein	0	0	0	1	1	1	1	1	1	0	0	0	0	0	0	0	6	6	0	-	0.0001998001998002
38023_hypothetical_protein	0	0	0	1	1	1	1	1	1	0	0	0	0	0	0	0	6	6	0	L	0.0001998001998002
38058_hypothetical_protein	0	0	0	1	1	1	1	1	1	0	0	0	0	0	0	0	6	6	0	-	0.0001998001998002

38059_hypothetical_protein	0	0	0	1	1	1	1	1	1	0	0	0	0	0	0	0	6	6	0	L	0.0001998001998002
38060_hypothetical_protein	0	0	0	1	1	1	1	1	1	0	0	0	0	0	0	0	6	6	0	K	0.0001998001998002
38061_hypothetical_protein	0	0	0	1	1	1	1	1	1	0	0	0	0	0	0	0	6	6	0	-	0.0001998001998002
38062_hypothetical_protein	0	0	0	1	1	1	1	1	1	0	0	0	0	0	0	0	6	6	0	-	0.0001998001998002
38063_hypothetical_protein	0	0	0	1	1	1	1	1	1	0	0	0	0	0	0	0	6	6	0	L	0.0001998001998002
38064_era_2	0	0	0	1	1	1	1	1	1	0	0	0	0	0	0	0	6	6	0	S	0.0001998001998002
38065_hypothetical_protein	0	0	0	1	1	1	1	1	1	0	0	0	0	0	0	0	6	6	0	-	0.0001998001998002
38066_hypothetical_protein	0	0	0	1	1	1	1	1	1	0	0	0	0	0	0	0	6	6	0	K	0.0001998001998002
38067_hypothetical_protein	0	0	0	1	1	1	1	1	1	0	0	0	0	0	0	0	6	6	0	-	0.0001998001998002
38068_yafY	0	0	0	1	1	1	1	1	1	0	0	0	0	0	0	0	6	6	0	K	0.0001998001998002
38069_hypothetical_protein	0	0	0	1	1	1	1	1	1	0	0	0	0	0	0	0	6	6	0	-	0.0001998001998002
38070_hypothetical_protein	0	0	0	1	1	1	1	1	1	0	0	0	0	0	0	0	6	6	0	S	0.0001998001998002
38071_hypothetical_protein	0	0	0	1	1	1	1	1	1	0	0	0	0	0	0	0	6	6	0	-	0.0001998001998002
38072_hypothetical_protein	0	0	0	1	1	1	1	1	1	0	0	0	0	0	0	0	6	6	0	S	0.0001998001998002
38073_hypothetical_protein	0	0	0	1	1	1	1	1	1	0	0	0	0	0	0	0	6	6	0	S	0.0001998001998002
38074_hypothetical_protein	0	0	0	1	1	1	1	1	1	0	0	0	0	0	0	0	6	6	0	-	0.0001998001998002
38075_klcA	0	0	0	1	0	1	1	1	1	0	0	0	0	0	0	0	5	5	0	S	0.001998001998001998 4
38076_hypothetical_protein	0	0	0	1	1	1	1	1	1	0	0	0	0	0	0	0	6	6	0	E	0.0001998001998002
38077_hypothetical_protein	0	0	0	1	1	1	1	1	1	0	0	0	0	0	0	0	6	6	0	-	0.0001998001998002
38078_hypothetical_protein	0	0	0	1	1	1	1	1	1	0	0	0	0	0	0	0	6	6	0	S	0.0001998001998002
38079_hypothetical_protein	0	0	0	1	1	1	1	1	1	0	0	0	0	0	0	0	6	6	0	S	0.0001998001998002
38080_cbeA	0	0	0	1	1	1	1	1	1	0	0	0	0	0	0	0	6	6	0	S	0.0001998001998002
38081_hypothetical_protein	0	0	0	1	1	1	1	1	1	0	0	0	0	0	0	0	6	6	0	S	0.0001998001998002
38082_hypothetical_protein	0	0	0	1	1	1	1	1	1	0	0	0	0	0	0	0	6	6	0	S	0.0001998001998002
38083_hypothetical_protein	0	0	0	1	1	1	1	1	1	0	0	0	0	0	0	0	6	6	0	-	0.0001998001998002
38084_hypothetical_protein	0	0	0	1	1	1	1	1	1	0	0	0	0	0	0	0	6	6	0	S	0.0001998001998002
38085_hypothetical_protein	0	0	0	1	1	1	1	1	1	0	0	0	0	0	0	0	6	6	0	L	0.0001998001998002
38152_hypothetical_protein	0	0	0	0	1	1	1	1	1	0	0	0	0	0	0	0	5	5	0	-	0.0019980019980019984
38203_yjcD	0	0	0	1	1	1	1	1	1	0	0	0	0	0	0	0	6	6	0	L	0.0001998001998002
38204_hypothetical_protein	0	0	0	1	1	1	1	1	1	0	0	0	0	0	0	0	6	6	0	S	0.0001998001998002
38205_hypothetical_protein	0	0	0	1	1	1	1	1	1	0	0	0	0	0	0	0	6	6	0	-	0.0001998001998002
38206_hypothetical_protein	0	0	0	1	1	1	1	1	1	0	0	0	0	0	0	0	6	6	0	-	0.0001998001998002
38207_hypothetical_protein	0	0	0	1	1	1	1	1	1	0	0	0	0	0	0	0	6	6	0	-	0.0001998001998002
38208_hypothetical_protein	0	0	0	1	1	1	1	1	1	0	0	0	0	0	0	0	6	6	0	-	0.0001998001998002
38209_hypothetical_protein	0	0	0	1	1	1	1	1	1	0	0	0	0	0	0	0	6	6	0	K	0.0001998001998002
38211_hypothetical_protein	0	0	0	1	1	1	1	1	1	0	0	0	0	0	0	0	6	6	0	-	0.0001998001998002
38212_hypothetical_protein	0	0	0	1	1	1	1	1	1	0	0	0	0	0	0	0	6	6	0	-	0.0001998001998002
38213_hypothetical_protein	0	0	0	1	1	1	1	1	1	0	0	0	0	0	0	0	6	6	0	V	0.0001998001998002
38214_hypothetical_protein	0	0	0	1	1	1	1	1	1	0	0	0	0	0	0	0	6	6	0	-	0.0001998001998002
38215_hypothetical_protein	0	0	0	1	1	1	1	1	1	0	0	0	0	0	0	0	6	6	0	-	0.0001998001998002
38216_hypothetical_protein	0	0	0	1	1	1	1	1	1	0	0	0	0	0	0	0	6	6	0	V	0.0001998001998002
38217_hypothetical_protein	0	0	0	1	1	1	1	1	1	0	0	0	0	0	0	0	6	6	0	-	0.0001998001998002
38218_hns_2	0	0	0	1	1	1	1	1	1	0	0	0	0	0	0	0	6	6	0	K	0.0001998001998002
38219_hypothetical_protein	0	0	0	1	1	1	1	1	1	0	0	0	0	0	0	0	6	6	0	-	0.0001998001998002
38220_Tn3_family_transposa..	0	0	0	0	1	1	1	1	1	0	0	0	0	0	0	0	5	5	0	L	0.0019980019980019984
38221_bla_2	0	0	0	0	1	1	1	1	1	0	0	0	0	0	0	0	5	5	0	V	0.0019980019980019984
38345_hypothetical_protein	0	0	0	1	1	1	1	1	1	0	0	0	0	0	0	0	6	6	0	-	0.0001998001998002
38346_ynfE_2	0	0	0	1	1	1	1	1	1	0	0	0	0	0	0	0	6	6	0	C	0.0001998001998002
38347_ynfE_3	0	0	0	1	1	1	1	1	1	0	0	0	0	0	0	0	6	6	0	C	0.0001998001998002
38356_hypothetical_protein	0	0	0	1	1	1	1	1	1	0	0	0	0	0	0	0	6	6	0	L	0.0001998001998002

38374_hypothetical_protein	0	0	0	0	1	1	1	1	1	0	0	0	0	0	0	0	5	5	0	S	0.0019980019980019984
38517_intS_1	0	0	0	1	1	1	1	1	1	0	0	0	0	0	0	0	6	6	0	L	0.0001998001998002
38519_hypothetical_protein	0	0	0	1	1	1	1	1	1	0	0	0	0	0	0	0	6	6	0	S	0.0001998001998002
38524_hypothetical_protein	0	0	0	1	1	1	1	1	1	0	0	0	0	0	0	0	6	6	0	S	0.0001998001998002
38525_hypothetical_protein	0	0	0	1	1	1	1	1	1	0	0	0	0	0	0	0	6	6	0	K	0.0001998001998002
38526_hypothetical_protein	0	0	0	1	1	1	1	1	1	0	0	0	0	0	0	0	6	6	0	-	0.0001998001998002
38527_hypothetical_protein	0	0	0	1	1	1	1	1	1	0	0	0	0	0	0	0	6	6	0	-	0.0001998001998002
38528_hypothetical_protein	0	0	0	1	1	1	1	1	1	0	0	0	0	0	0	0	6	6	0	-	0.0001998001998002
38529_hypothetical_protein	0	0	0	1	1	1	1	1	1	0	0	0	0	0	0	0	6	6	0	S	0.0001998001998002
38531_hypothetical_protein	0	0	0	1	1	1	1	1	1	0	0	0	0	0	0	0	6	6	0	K	0.0001998001998002
38532_hypothetical_protein	0	0	0	1	1	1	1	1	1	0	0	0	0	0	0	0	6	6	0	K	0.0001998001998002
38533_hypothetical_protein	0	0	0	1	1	1	1	1	1	0	0	0	0	0	0	0	6	6	0	J	0.0001998001998002
38534_hypothetical_protein	0	0	0	1	1	1	1	1	1	0	0	0	0	0	0	0	6	6	0	U	0.0001998001998002
38535_hypothetical_protein	0	0	0	1	1	1	1	1	1	0	0	0	0	0	0	0	6	6	0	S	0.0001998001998002
38537_hypothetical_protein	0	0	0	1	1	1	1	1	1	0	0	0	0	0	0	0	6	6	0	-	0.0001998001998002
38538_hypothetical_protein	0	0	0	1	1	1	1	1	1	0	0	0	0	0	0	0	6	6	0	S	0.0001998001998002
38539_hypothetical_protein	0	0	0	1	1	1	1	1	1	0	0	0	0	0	0	0	6	6	0	-	0.0001998001998002
38540_hypothetical_protein	0	0	0	1	1	1	1	1	1	0	0	0	0	0	0	0	6	6	0	S	0.0001998001998002
38541_hypothetical_protein	0	0	0	1	1	1	1	1	1	0	0	0	0	0	0	0	6	6	0	U	0.0001998001998002
38542_hypothetical_protein	0	0	0	1	1	1	1	1	1	0	0	0	0	0	0	0	6	6	0	M	0.0001998001998002
38543_hypothetical_protein	0	0	0	1	1	1	1	1	1	0	0	0	0	0	0	0	6	6	0	O	0.0001998001998002
38544_hypothetical_protein	0	0	0	1	1	1	1	1	1	0	0	0	0	0	0	0	6	6	0	S	0.0001998001998002
38545_hypothetical_protein	0	0	0	1	1	1	1	1	1	0	0	0	0	0	0	0	6	6	0	-	0.0001998001998002
38571_hsdS	0	0	0	1	1	1	1	1	1	0	0	0	0	0	0	0	6	6	0	V	0.0001998001998002
38652_hypothetical_protein	0	0	0	1	1	1	1	1	1	0	0	0	0	0	0	0	6	6	0	S	0.0001998001998002
38653_hypothetical_protein	0	0	0	1	1	1	1	1	1	0	0	0	0	0	0	0	6	6	0	S	0.0001998001998002
38731_hypothetical_protein	0	0	0	1	1	1	1	1	1	0	0	0	0	0	0	0	6	6	0	L	0.0001998001998002
38733_hypothetical_protein	0	0	0	1	1	1	1	1	1	0	0	0	0	0	0	0	6	6	0	S	0.0001998001998002
38734_hypothetical_protein	0	0	0	1	1	1	1	1	1	0	0	0	0	0	0	0	6	6	0	L	0.0001998001998002
38735_hypothetical_protein	0	0	0	1	1	1	1	1	1	0	0	0	0	0	0	0	6	6	0	T	0.0001998001998002
38736_hypothetical_protein	0	0	0	1	1	1	1	1	1	0	0	0	0	0	0	0	6	6	0	S	0.0001998001998002
38737_hypothetical_protein	0	0	0	1	1	1	1	1	1	0	0	0	0	0	0	0	6	6	0	K	0.0001998001998002
38738_merB	0	0	0	1	1	1	1	1	1	0	0	0	0	0	0	0	6	6	0	C	0.0001998001998002
38739_hypothetical_protein	0	0	0	1	1	1	1	1	1	0	0	0	0	0	0	0	6	6	0	-	0.0001998001998002
38740_ISL3_family_transpos..	0	0	0	1	1	1	1	1	1	0	0	0	0	0	0	0	6	6	0	K	0.0001998001998002
38741_IS110_family_transpo..	0	0	0	1	1	1	1	1	1	0	0	0	0	0	0	0	6	6	0	L	0.0001998001998002
38742_hypothetical_protein	0	0	0	1	1	1	1	1	1	0	0	0	0	0	0	0	6	6	0	S	0.0001998001998002
38743_hypothetical_protein	0	0	0	1	1	1	1	1	1	0	0	0	0	0	0	0	6	6	0	S	0.0001998001998002
38744_hypothetical_protein	0	0	0	1	1	1	1	1	1	0	0	0	0	0	0	0	6	6	0	S	0.0001998001998002
38745_hypothetical_protein	0	0	0	1	1	1	1	1	1	0	0	0	0	0	0	0	6	6	0	-	0.0001998001998002
38746_hypothetical_protein	0	0	0	1	1	1	1	1	1	0	0	0	0	0	0	0	6	6	0	-	0.0001998001998002
38747_hypothetical_protein	0	0	0	1	1	1	1	1	1	0	0	0	0	0	0	0	6	6	0	-	0.0001998001998002
38748_hin_2	0	0	0	1	1	1	1	1	1	0	0	0	0	0	0	0	6	6	0	L	0.0001998001998002
38905_bspRIM	0	0	0	1	1	1	1	1	1	0	0	0	0	0	0	0	6	6	0	H	0.0001998001998002
38906_hypothetical_protein	0	0	0	1	1	1	1	1	1	0	0	0	0	0	0	0	6	6	0	-	0.0001998001998002
38907_hypothetical_protein	0	0	0	1	1	1	1	1	1	0	0	0	0	0	0	0	6	6	0	-	0.0001998001998002
38908_hypothetical_protein	0	0	0	1	1	1	1	1	1	0	0	0	0	0	0	0	6	6	0	L	0.0001998001998002
38909_hypothetical_protein	0	0	0	1	1	1	1	1	1	0	0	0	0	0	0	0	6	6	0	L	0.0001998001998002
38910_hypothetical_protein	0	0	0	1	1	1	1	1	1	0	0	0	0	0	0	0	6	6	0	-	0.0001998001998002
38912_hypothetical_protein	0	0	0	1	1	1	1	1	1	0	0	0	0	0	0	0	6	6	0	L	0.0001998001998002

38913_hypothetical_protein	0	0	0	1	1	1	1	1	1	0	0	0	0	0	0	0	6	6	0	L	0.0001998001998002
38914_intS_2	0	0	0	1	1	1	1	1	1	0	0	0	0	0	0	0	6	6	0	L	0.0001998001998002
38916_hypothetical_protein	0	0	0	1	1	1	1	1	1	0	0	0	0	0	0	0	6	6	0	-	0.0001998001998002
39294_hypothetical_protein	0	0	0	1	1	1	1	1	1	0	0	0	0	0	0	0	6	6	0	S	0.0001998001998002
39295_hypothetical_protein	0	0	0	1	1	1	1	1	1	0	0	0	0	0	0	0	6	6	0	-	0.0001998001998002
39395_hypothetical_protein	0	0	0	0	1	1	1	1	1	0	0	0	0	0	0	0	5	5	0	S	0.001998001998001998 4
39406_hypothetical_protein	0	0	0	1	1	1	1	1	1	0	0	0	0	0	0	0	6	6	0	P	0.0001998001998002
39604_acrZ	0	0	0	1	1	1	1	1	1	0	0	0	0	0	0	0	6	6	0	U	0.0001998001998002
39664_ybiV_1	0	0	0	1	1	1	1	1	1	0	0	0	0	0	0	0	6	6	0	S	0.0001998001998002
39665_hypothetical_protein	0	0	0	1	1	1	1	1	1	0	0	0	0	0	0	0	6	6	0	-	0.0001998001998002
39911_hypothetical_protein	0	0	0	0	1	1	1	1	1	0	0	0	0	0	0	0	5	5	0	C	0.0019980019980019984
39913_xerC_1	0	0	0	0	1	1	1	1	1	0	0	0	0	0	0	0	5	5	0	L	0.0019980019980019984
39914_hypothetical_protein	0	0	0	0	1	1	1	1	1	0	0	0	0	0	0	0	5	5	0	-	0.0019980019980019984
39915_hypothetical_protein	0	0	0	0	1	1	1	1	1	0	0	0	0	0	0	0	5	5	0	L	0.0019980019980019984
39916_hypothetical_protein	0	0	0	0	1	1	1	1	1	0	0	0	0	0	0	0	5	5	0	-	0.0019980019980019984
39918_hypothetical_protein	0	0	0	0	1	1	1	1	1	0	0	0	0	0	0	0	5	5	0	K	0.0019980019980019984
39919_nemA_2	0	0	0	0	1	1	1	1	1	0	0	0	0	0	0	0	5	5	0	C	0.0019980019980019984
39920_pgrR_2	0	0	0	0	1	1	1	1	1	0	0	0	0	0	0	0	5	5	0	K	0.0019980019980019984
39921_hypothetical_protein	0	0	0	0	1	1	1	1	1	0	0	0	0	0	0	0	5	5	0	S	0.0019980019980019984
39922_hypothetical_protein	0	0	0	0	1	1	1	1	1	0	0	0	0	0	0	0	5	5	0	EG	0.0019980019980019984
39923_hypothetical_protein	0	0	0	0	1	1	1	1	1	0	0	0	0	0	0	0	5	5	0	K	0.0019980019980019984
39924_yhfK	0	0	0	0	1	1	1	1	1	0	0	0	0	0	0	0	5	5	0	GM	0.0019980019980019984
39925_hypothetical_protein	0	0	0	0	1	1	1	1	1	0	0	0	0	0	0	0	5	5	0	K	0.0019980019980019984
39926_yhjQ_2	0	0	0	0	1	1	1	1	1	0	0	0	0	0	0	0	5	5	0	C	0.0019980019980019984
39927_fdhA	0	0	0	0	1	1	1	1	1	0	0	0	0	0	0	0	5	5	0	E	0.0019980019980019984
39928_hypothetical_protein	0	0	0	0	1	1	1	1	1	0	0	0	0	0	0	0	5	5	0	S	0.0019980019980019984
39929_uvrA_2	0	0	0	0	1	1	1	1	1	0	0	0	0	0	0	0	5	5	0	L	0.0019980019980019984
39930_IS3_family_transposa..	0	0	0	0	1	1	1	1	1	0	0	0	0	0	0	0	5	5	0	L	0.0019980019980019984
39931_frmB	0	0	0	0	1	1	1	1	1	0	0	0	0	0	0	0	5	5	0	S	0.0019980019980019984
39932_hypothetical_protein	0	0	0	0	1	1	1	1	1	0	0	0	0	0	0	0	5	5	0	E	0.0019980019980019984
39934_frmR_2	0	0	0	0	1	1	1	1	1	0	0	0	0	0	0	0	5	5	0	S	0.0019980019980019984
39935_IS1_family_transposa..	0	0	0	0	1	1	1	1	1	0	0	0	0	0	0	0	5	5	0	L	0.0019980019980019984
39936_IS1_family_transposa..	0	0	0	0	1	1	1	1	1	0	0	0	0	0	0	0	5	5	0	L	0.0019980019980019984
39937_acuI_1	0	0	0	0	1	1	1	1	1	0	0	0	0	0	0	0	5	5	0	C	0.0019980019980019984
39938_acuR	0	0	0	0	1	1	1	1	1	0	0	0	0	0	0	0	5	5	0	K	0.0019980019980019984
39939_hypothetical_protein	0	0	0	0	1	1	1	1	1	0	0	0	0	0	0	0	5	5	0	K	0.0019980019980019984
39940_hypothetical_protein	0	0	0	0	1	1	1	1	1	0	0	0	0	0	0	0	5	5	0	-	0.0019980019980019984
39941_hypothetical_protein	0	0	0	0	1	1	1	1	1	0	0	0	0	0	0	0	5	5	0	S	0.0019980019980019984
39943_IS3_family_transposa..	0	0	0	0	1	1	1	1	1	0	0	0	0	0	0	0	5	5	0	L	0.0019980019980019984
39944_IS3_family_transposa..	0	0	0	0	1	1	1	1	1	0	0	0	0	0	0	0	5	5	0	L	0.0019980019980019984
39946_hypothetical_protein	0	0	0	0	1	1	1	1	1	0	0	0	0	0	0	0	5	5	0	S	0.0019980019980019984
39947_hypothetical_protein	0	0	0	0	1	1	1	1	1	0	0	0	0	0	0	0	5	5	0	-	0.0019980019980019984
39948_hypothetical_protein	0	0	0	0	1	1	1	1	1	0	0	0	0	0	0	0	5	5	0	EH	0.0019980019980019984
39949_hypothetical_protein	0	0	0	1	1	1	1	1	1	0	0	0	0	0	0	0	6	6	0	-	0.0001998001998002
39950_hypothetical_protein	0	0	0	1	1	1	1	1	1	0	0	0	0	0	0	0	6	6	0	-	0.0001998001998002
39951_hypothetical_protein	0	0	0	1	1	1	1	1	1	0	0	0	0	0	0	0	6	6	0	-	0.0001998001998002
39952_hypothetical_protein	0	0	0	1	1	1	1	1	1	0	0	0	0	0	0	0	6	6	0	L	0.0001998001998002
39953_hypothetical_protein	0	0	0	1	1	1	1	1	1	0	0	0	0	0	0	0	6	6	0	-	0.0001998001998002
39954_hypothetical_protein	0	0	0	1	1	1	1	1	1	0	0	0	0	0	0	0	6	6	0	-	0.0001998001998002
39955_hypothetical_protein	0	0	0	1	1	1	1	1	1	0	0	0	0	0	0	0	6	6	0	-	0.0001998001998002

39956_TelA-like_protein	0	0	0	1	1	1	1	1	1	0	0	0	0	0	0	0	6	6	0	P	0.0001998001998002
39957_hypothetical_protein	0	0	0	1	1	1	1	1	1	0	0	0	0	0	0	0	6	6	0	-	0.0001998001998002
39958_hypothetical_protein	0	0	0	1	1	1	1	1	1	0	0	0	0	0	0	0	6	6	0	-	0.0001998001998002
39959_hypothetical_protein	0	0	0	1	1	1	1	1	1	0	0	0	0	0	0	0	6	6	0	-	0.0001998001998002
39960_hypothetical_protein	0	0	0	1	1	1	1	1	1	0	0	0	0	0	0	0	6	6	0	S	0.0001998001998002
39961_hypothetical_protein	0	0	0	1	1	1	1	1	1	0	0	0	0	0	0	0	6	6	0	S	0.0001998001998002
39962_hypothetical_protein	0	0	0	1	1	1	1	1	1	0	0	0	0	0	0	0	6	6	0	-	0.0001998001998002
39963_hypothetical_protein	0	0	0	1	1	1	1	1	1	0	0	0	0	0	0	0	6	6	0	-	0.0001998001998002
39964_hypothetical_protein	0	0	0	1	1	1	1	1	1	0	0	0	0	0	0	0	6	6	0	-	0.0001998001998002
39965_hypothetical_protein	0	0	0	1	1	1	1	1	1	0	0	0	0	0	0	0	6	6	0	-	0.0001998001998002
39966_hypothetical_protein	0	0	0	1	1	1	1	1	1	0	0	0	0	0	0	0	6	6	0	-	0.0001998001998002
39967_hypothetical_protein	0	0	0	1	1	1	1	1	1	0	0	0	0	0	0	0	6	6	0	-	0.0001998001998002
39968_hypothetical_protein	0	0	0	1	1	1	1	1	1	0	0	0	0	0	0	0	6	6	0	-	0.0001998001998002
39969_hypothetical_protein	0	0	0	1	1	1	1	1	1	0	0	0	0	0	0	0	6	6	0	S	0.0001998001998002
39970_hypothetical_protein	0	0	0	1	1	1	1	1	1	0	0	0	0	0	0	0	6	6	0	-	0.0001998001998002
39971_hypothetical_protein	0	0	0	1	1	1	1	1	1	0	0	0	0	0	0	0	6	6	0	-	0.0001998001998002
39972_hypothetical_protein	0	0	0	1	1	1	1	1	1	0	0	0	0	0	0	0	6	6	0	-	0.0001998001998002
39973_hypothetical_protein	0	0	0	1	1	1	1	1	1	0	0	0	0	0	0	0	6	6	0	-	0.0001998001998002
39974_hypothetical_protein	0	0	0	1	1	1	1	1	1	0	0	0	0	0	0	0	6	6	0	S	0.0001998001998002
39975_hypothetical_protein	0	0	0	1	1	1	1	1	1	0	0	0	0	0	0	0	6	6	0	-	0.0001998001998002
39976_hypothetical_protein	0	0	0	1	1	1	1	1	1	0	0	0	0	0	0	0	6	6	0	-	0.0001998001998002
39977_hypothetical_protein	0	0	0	1	1	1	1	1	1	0	0	0	0	0	0	0	6	6	0	-	0.0001998001998002
39978_hypothetical_protein	0	0	0	1	1	1	1	1	1	0	0	0	0	0	0	0	6	6	0	-	0.0001998001998002
39979_hypothetical_protein	0	0	0	1	1	1	1	1	1	0	0	0	0	0	0	0	6	6	0	-	0.0001998001998002
39980_hypothetical_protein	0	0	0	1	1	1	1	1	1	0	0	0	0	0	0	0	6	6	0	-	0.0001998001998002
39981_hypothetical_protein	0	0	0	1	1	1	1	1	1	0	0	0	0	0	0	0	6	6	0	-	0.0001998001998002
39982_hypothetical_protein	0	0	0	1	1	1	1	1	1	0	0	0	0	0	0	0	6	6	0	-	0.0001998001998002
39983_hypothetical_protein	0	0	0	1	1	1	1	1	1	0	0	0	0	0	0	0	6	6	0	-	0.0001998001998002
39984_hypothetical_protein	0	0	0	1	1	1	1	1	1	0	0	0	0	0	0	0	6	6	0	-	0.0001998001998002
39985_hypothetical_protein	0	0	0	1	1	1	1	1	1	0	0	0	0	0	0	0	6	6	0	-	0.0001998001998002
39986_hypothetical_protein	0	0	0	1	1	1	1	1	1	0	0	0	0	0	0	0	6	6	0	-	0.0001998001998002
39987_hypothetical_protein	0	0	0	1	1	1	1	1	1	0	0	0	0	0	0	0	6	6	0	L	0.0001998001998002
39988_hypothetical_protein	0	0	0	1	1	1	1	1	1	0	0	0	0	0	0	0	6	6	0	OU	0.0001998001998002
39989_hypothetical_protein	0	0	0	1	1	1	1	1	1	0	0	0	0	0	0	0	6	6	0	S	0.0001998001998002
39990_hypothetical_protein	0	0	0	1	1	1	1	1	1	0	0	0	0	0	0	0	6	6	0	-	0.0001998001998002
39991_hypothetical_protein	0	0	0	1	1	1	1	1	1	0	0	0	0	0	0	0	6	6	0	S	0.0001998001998002
39992_hypothetical_protein	0	0	0	1	1	1	1	1	1	0	0	0	0	0	0	0	6	6	0	S	0.0001998001998002
39993_hypothetical_protein	0	0	0	1	1	1	1	1	1	0	0	0	0	0	0	0	6	6	0	-	0.0001998001998002
39994_hypothetical_protein	0	0	0	1	1	1	1	1	1	0	0	0	0	0	0	0	6	6	0	-	0.0001998001998002
39995_hypothetical_protein	0	0	0	1	1	1	1	1	1	0	0	0	0	0	0	0	6	6	0	-	0.0001998001998002
39996_hypothetical_protein	0	0	0	1	1	1	1	1	1	0	0	0	0	0	0	0	6	6	0	-	0.0001998001998002
39997_hypothetical_protein	0	0	0	1	1	1	1	1	1	0	0	0	0	0	0	0	6	6	0	CO	0.0001998001998002
39998_hypothetical_protein	0	0	0	1	1	1	1	1	1	0	0	0	0	0	0	0	6	6	0	S	0.0001998001998002
39999_hypothetical_protein	0	0	0	1	1	1	1	1	1	0	0	0	0	0	0	0	6	6	0	G	0.0001998001998002
40000_hypothetical_protein	0	0	0	1	1	1	1	1	1	0	0	0	0	0	0	0	6	6	0	-	0.0001998001998002
40001_hypothetical_protein	0	0	0	1	1	1	1	1	1	0	0	0	0	0	0	0	6	6	0	M	0.0001998001998002
40002_hypothetical_protein	0	0	0	1	1	1	1	1	1	0	0	0	0	0	0	0	6	6	0	-	0.0001998001998002
40003_hypothetical_protein	0	0	0	1	1	1	1	1	1	0	0	0	0	0	0	0	6	6	0	M	0.0001998001998002
40004_dsbC_2	0	0	0	1	1	1	1	1	1	0	0	0	0	0	0	0	6	6	0	O	0.0001998001998002
40005_hypothetical_protein	0	0	0	1	1	1	1	1	1	0	0	0	0	0	0	0	6	6	0	-	0.0001998001998002

40006_hypothetical_protein	0	0	0	1	1	1	1	1	1	0	0	0	0	0	0	0	6	6	0	-	0.0001998001998002
40007_hypothetical_protein	0	0	0	1	1	1	1	1	1	0	0	0	0	0	0	0	6	6	0	-	0.0001998001998002
40008_hypothetical_protein	0	0	0	1	1	1	1	1	1	0	0	0	0	0	0	0	6	6	0	O	0.0001998001998002
40009_umuC_1	0	0	0	1	1	1	1	1	1	0	0	0	0	0	0	0	6	6	0	L	0.0001998001998002
40010_umuD_1	0	0	0	1	1	1	1	1	1	0	0	0	0	0	0	0	6	6	0	KT	0.0001998001998002
40011_hypothetical_protein	0	0	0	1	1	1	1	1	1	0	0	0	0	0	0	0	6	6	0	-	0.0001998001998002
40012_hypothetical_protein	0	0	0	1	1	1	1	1	1	0	0	0	0	0	0	0	6	6	0	-	0.0001998001998002
40013_hypothetical_protein	0	0	0	1	1	1	1	1	1	0	0	0	0	0	0	0	6	6	0	-	0.0001998001998002
40014_hypothetical_protein	0	0	0	1	1	1	1	1	1	0	0	0	0	0	0	0	6	6	0	-	0.0001998001998002
40015_hypothetical_protein	0	0	0	1	1	1	1	1	1	0	0	0	0	0	0	0	6	6	0	-	0.0001998001998002
40016_hypothetical_protein	0	0	0	1	1	1	1	1	1	0	0	0	0	0	0	0	6	6	0	-	0.0001998001998002
40017_hypothetical_protein	0	0	0	1	0	1	1	1	1	0	0	0	0	0	0	0	5	5	0	-	0.0019980019980019984
40427_hypothetical_protein	0	0	0	1	1	1	1	1	1	0	0	0	0	0	0	0	6	6	0	-	0.0001998001998002
40458_sopA_1	0	0	0	1	1	1	1	1	1	0	0	0	0	0	0	0	6	6	0	O	0.0001998001998002
40459_sopA_2	0	0	0	1	1	1	1	1	1	0	0	0	0	0	0	0	6	6	0	S	0.0001998001998002
40489_hypothetical_protein	0	0	0	1	1	1	1	1	1	0	0	0	0	0	0	0	6	6	0	-	0.0001998001998002
40490_hypothetical_protein	0	0	0	1	1	1	1	1	1	0	0	0	0	0	0	0	6	6	0	V	0.0001998001998002
40491_hypothetical_protein	0	0	0	1	1	1	1	1	1	0	0	0	0	0	0	0	6	6	0	-	0.0001998001998002
40492_hypothetical_protein	0	0	0	1	1	1	1	1	1	0	0	0	0	0	0	0	6	6	0	-	0.0001998001998002
40493_hypothetical_protein	0	0	0	1	1	1	1	1	1	0	0	0	0	0	0	0	6	6	0	-	0.0001998001998002
40494_hypothetical_protein	0	0	0	1	1	1	1	1	1	0	0	0	0	0	0	0	6	6	0	T	0.0001998001998002
40495_yceD_2	0	0	0	1	1	1	1	1	1	0	0	0	0	0	0	0	6	6	0	T	0.0001998001998002
40496_yceD_3	0	0	0	1	1	1	1	1	1	0	0	0	0	0	0	0	6	6	0	T	0.0001998001998002
40497_alx_2	0	0	0	1	1	1	1	1	1	0	0	0	0	0	0	0	6	6	0	P	0.0001998001998002
40498_hypothetical_protein	0	0	0	1	1	1	1	1	1	0	0	0	0	0	0	0	6	6	0	P	0.0001998001998002
40499_hypothetical_protein	0	0	0	1	1	1	1	1	1	0	0	0	0	0	0	0	6	6	0	T	0.0001998001998002
40500_yceD_4	0	0	0	1	1	1	1	1	1	0	0	0	0	0	0	0	6	6	0	T	0.0001998001998002
40501_hypothetical_protein	0	0	0	1	1	1	1	1	1	0	0	0	0	0	0	0	6	6	0	HJ	0.0001998001998002
40502_hypothetical_protein	0	0	0	1	1	1	1	1	1	0	0	0	0	0	0	0	6	6	0	F	0.0001998001998002
40503_hypothetical_protein	0	0	0	1	1	1	1	1	1	0	0	0	0	0	0	0	6	6	0	S	0.0001998001998002
40504_stiP	0	0	0	1	1	1	1	1	1	0	0	0	0	0	0	0	6	6	0	J	0.0001998001998002
40505_hypothetical_protein	0	0	0	1	1	1	1	1	1	0	0	0	0	0	0	0	6	6	0	G	0.0001998001998002
40506_hypothetical_protein	0	0	0	1	1	1	1	1	1	0	0	0	0	0	0	0	6	6	0	S	0.0001998001998002
40507_hypothetical_protein	0	0	0	1	1	1	1	1	1	0	0	0	0	0	0	0	6	6	0	-	0.0001998001998002
40508_hypothetical_protein	0	0	0	1	1	1	1	1	1	0	0	0	0	0	0	0	6	6	0	-	0.0001998001998002
40509_hypothetical_protein	0	0	0	1	1	1	1	1	1	0	0	0	0	0	0	0	6	6	0	S	0.0001998001998002
40510_yceD_5	0	0	0	1	1	1	1	1	1	0	0	0	0	0	0	0	6	6	0	T	0.0001998001998002
40511_hypothetical_protein	0	0	0	1	1	1	1	1	1	0	0	0	0	0	0	0	6	6	0	S	0.0001998001998002
40512_hypothetical_protein	0	0	0	1	1	1	1	1	1	0	0	0	0	0	0	0	6	6	0	S	0.0001998001998002
40513_hypothetical_protein	0	0	0	1	1	1	1	1	1	0	0	0	0	0	0	0	6	6	0	T	0.0001998001998002
40514_hypothetical_protein	0	0	0	1	1	1	1	1	1	0	0	0	0	0	0	0	6	6	0	S	0.0001998001998002
40515_hypothetical_protein	0	0	0	1	1	1	1	1	1	0	0	0	0	0	0	0	6	6	0	S	0.0001998001998002
40516_hha_2	0	0	0	1	1	1	1	1	1	0	0	0	0	0	0	0	6	6	0	S	0.0001998001998002
40517_hypothetical_protein	0	0	0	1	1	1	1	1	1	0	0	0	0	0	0	0	6	6	0	L	0.0001998001998002
40518_hypothetical_protein	0	0	0	1	1	1	1	1	1	0	0	0	0	0	0	0	6	6	0	-	0.0001998001998002
40519_hypothetical_protein	0	0	0	1	1	1	1	1	1	0	0	0	0	0	0	0	6	6	0	-	0.0001998001998002
40520_hypothetical_protein	0	0	0	1	1	1	1	1	1	0	0	0	0	0	0	0	6	6	0	S	0.0001998001998002
40521_hypothetical_protein	0	0	0	1	1	1	1	1	1	0	0	0	0	0	0	0	6	6	0	-	0.0001998001998002
40522_hypothetical_protein	0	0	0	1	1	1	1	1	1	0	0	0	0	0	0	0	6	6	0	-	0.0001998001998002
40523_hypothetical_protein	0	0	0	1	1	1	1	1	1	0	0	0	0	0	0	0	6	6	0	-	0.0001998001998002

40524_hypothetical_protein	0	0	0	1	1	1	1	1	1	0	0	0	0	0	0	0	6	6	0	-	0.0001998001998002
40525_hypothetical_protein	0	0	0	1	1	1	1	1	1	0	0	0	0	0	0	0	6	6	0	-	0.0001998001998002
40526_hypothetical_protein	0	0	0	1	1	1	1	1	1	0	0	0	0	0	0	0	6	6	0	-	0.0001998001998002
40527_hypothetical_protein	0	0	0	0	1	1	1	1	1	0	0	0	0	0	0	0	5	5	0	-	0.0019980019980019984
40528_hypothetical_protein	0	0	0	1	1	1	1	1	1	0	0	0	0	0	0	0	6	6	0	-	0.0001998001998002
40529_hypothetical_protein	0	0	0	1	1	1	1	1	1	0	0	0	0	0	0	0	6	6	0	-	0.0001998001998002
40530_hypothetical_protein	0	0	0	1	1	1	1	1	1	0	0	0	0	0	0	0	6	6	0	-	0.0001998001998002
40531_hypothetical_protein	0	0	0	1	1	1	1	1	1	0	0	0	0	0	0	0	6	6	0	-	0.0001998001998002
40532_hypothetical_protein	0	0	0	1	1	1	1	1	1	0	0	0	0	0	0	0	6	6	0	S	0.0001998001998002
40533_hypothetical_protein	0	0	0	1	1	1	1	1	1	0	0	0	0	0	0	0	6	6	0	-	0.0001998001998002
40534_hypothetical_protein	0	0	0	1	1	1	1	1	1	0	0	0	0	0	0	0	6	6	0	P	0.0001998001998002
40535_hypothetical_protein	0	0	0	1	1	1	1	1	1	0	0	0	0	0	0	0	6	6	0	S	0.0001998001998002
40536_hypothetical_protein	0	0	0	1	1	1	1	1	1	0	0	0	0	0	0	0	6	6	0	-	0.0001998001998002
40537_hypothetical_protein	0	0	0	1	1	1	1	1	1	0	0	0	0	0	0	0	6	6	0	S	0.0001998001998002
40538_hypothetical_protein	0	0	0	1	1	1	1	1	1	0	0	0	0	0	0	0	6	6	0	-	0.0001998001998002
40539_hypothetical_protein	0	0	0	1	1	1	1	1	1	0	0	0	0	0	0	0	6	6	0	-	0.0001998001998002
40540_hypothetical_protein	0	0	0	1	1	1	1	1	1	0	0	0	0	0	0	0	6	6	0	-	0.0001998001998002
40541_uvrD_2	0	0	0	1	1	1	1	1	1	0	0	0	0	0	0	0	6	6	0	L	0.0001998001998002
40542_hypothetical_protein	0	0	0	1	1	1	1	1	1	0	0	0	0	0	0	0	6	6	0	-	0.0001998001998002
40543_hypothetical_protein	0	0	0	1	1	1	1	1	1	0	0	0	0	0	0	0	6	6	0	S	0.0001998001998002
40544_hypothetical_protein	0	0	0	1	1	1	1	1	1	0	0	0	0	0	0	0	6	6	0	S	0.0001998001998002
40545_hypothetical_protein	0	0	0	1	1	1	1	1	1	0	0	0	0	0	0	0	6	6	0	-	0.0001998001998002
40546_hypothetical_protein	0	0	0	1	1	1	1	1	1	0	0	0	0	0	0	0	6	6	0	S	0.0001998001998002
40547_hypothetical_protein	0	0	0	1	1	1	1	1	1	0	0	0	0	0	0	0	6	6	0	U	0.0001998001998002
40548_hypothetical_protein	0	0	0	1	1	1	1	1	1	0	0	0	0	0	0	0	6	6	0	-	0.0001998001998002
40549_hypothetical_protein	0	0	0	1	1	1	1	1	1	0	0	0	0	0	0	0	6	6	0	-	0.0001998001998002
40550_hypothetical_protein	0	0	0	1	1	1	1	1	1	0	0	0	0	0	0	0	6	6	0	-	0.0001998001998002
40551_hypothetical_protein	0	0	0	1	1	1	1	1	1	0	0	0	0	0	0	0	6	6	0	-	0.0001998001998002
40552_hypothetical_protein	0	0	0	1	1	1	1	1	1	0	0	0	0	0	0	0	6	6	0	-	0.0001998001998002
40757_hypothetical_protein	0	0	0	1	1	1	1	1	1	0	0	0	0	0	0	0	6	6	0	V	0.0001998001998002
40758_hypothetical_protein	0	0	0	1	1	1	1	1	1	0	0	0	0	0	0	0	6	6	0	S	0.0001998001998002
40759_hypothetical_protein	0	0	0	1	1	1	1	1	1	0	0	0	0	0	0	0	6	6	0	L	0.0001998001998002
40790_yibH	0	0	0	1	1	1	1	1	1	0	0	0	0	0	0	0	6	6	0	V	0.0001998001998002
40791_yiaW	0	0	0	1	1	1	1	1	1	0	0	0	0	0	0	0	6	6	0	S	0.0001998001998002
40797_sadB_1	0	0	0	1	1	1	1	1	1	0	0	0	0	0	0	0	6	6	0	S	0.0001998001998002
40798_sadB_2	0	0	0	1	1	1	1	1	1	0	0	0	0	0	0	0	6	6	0	S	0.0001998001998002
40858_hypothetical_protein	0	0	0	1	1	1	1	1	1	0	0	0	0	0	0	0	6	6	0	L	0.0001998001998002
40899_ilvB_1	0	0	0	0	1	1	1	1	1	0	0	0	0	0	0	0	5	5	0	H	0.0019980019980019984
40900_ilvB_2	0	0	0	0	1	1	1	1	1	0	0	0	0	0	0	0	5	5	0	H	0.0019980019980019984
40918_hypothetical_protein	0	0	0	0	1	1	1	1	1	0	0	0	0	0	0	0	5	5	0	-	0.0019980019980019984
40919_hypothetical_protein	0	0	0	0	1	1	1	1	1	0	0	0	0	0	0	0	5	5	0	EH	0.0019980019980019984
40920_hypothetical_protein	0	0	0	1	1	1	1	1	1	0	0	0	0	0	0	0	6	6	0	-	0.0001998001998002
40921_hypothetical_protein	0	0	0	1	1	1	1	1	1	0	0	0	0	0	0	0	6	6	0	-	0.0001998001998002
40922_hypothetical_protein	0	0	0	1	1	1	1	1	1	0	0	0	0	0	0	0	6	6	0	-	0.0001998001998002
40923_hypothetical_protein	0	0	0	1	1	1	1	1	1	0	0	0	0	0	0	0	6	6	0	-	0.0001998001998002
40924_hypothetical_protein	0	0	0	1	1	1	1	1	1	0	0	0	0	0	0	0	6	6	0	-	0.0001998001998002
40925_hypothetical_protein	0	0	0	1	1	1	1	1	1	0	0	0	0	0	0	0	6	6	0	L	0.0001998001998002
40926_hypothetical_protein	0	0	0	1	1	1	1	1	1	0	0	0	0	0	0	0	6	6	0	S	0.0001998001998002
40927_hypothetical_protein	0	0	0	1	1	1	1	1	1	0	0	0	0	0	0	0	6	6	0	-	0.0001998001998002
40928_hypothetical_protein	0	0	0	1	1	1	1	1	1	0	0	0	0	0	0	0	6	6	0	-	0.0001998001998002

40929_hypothetical_protein	0	0	0	1	1	1	1	1	1	0	0	0	0	0	0	0	6	6	0	-	0.0001998001998002
40930_hypothetical_protein	0	0	0	0	1	1	1	1	1	0	0	0	0	0	0	0	5	5	0	-	0.0019980019980019984
40931_higB-1	0	0	0	1	1	1	1	1	1	0	0	0	0	0	0	0	6	6	0	S	0.0001998001998002
40932_hypothetical_protein	0	0	0	1	1	1	1	1	1	0	0	0	0	0	0	0	6	6	0	-	0.0001998001998002
40933_hypothetical_protein	0	0	0	1	1	1	1	1	1	0	0	0	0	0	0	0	6	6	0	S	0.0001998001998002
40934_hypothetical_protein	0	0	0	1	1	1	1	1	1	0	0	0	0	0	0	0	6	6	0	-	0.0001998001998002
40935_hypothetical_protein	0	0	0	1	1	1	1	1	1	0	0	0	0	0	0	0	6	6	0	-	0.0001998001998002
40936_hypothetical_protein	0	0	0	1	1	1	1	1	1	0	0	0	0	0	0	0	6	6	0	-	0.0001998001998002
40937_hypothetical_protein	0	0	0	1	1	1	1	1	1	0	0	0	0	0	0	0	6	6	0	-	0.0001998001998002
40938_hypothetical_protein	0	0	0	1	1	1	1	1	1	0	0	0	0	0	0	0	6	6	0	-	0.0001998001998002
40939_hypothetical_protein	0	0	0	1	1	1	1	1	1	0	0	0	0	0	0	0	6	6	0	-	0.0001998001998002
40940_hypothetical_protein	0	0	0	1	1	1	1	1	1	0	0	0	0	0	0	0	6	6	0	V	0.0001998001998002
40941_hypothetical_protein	0	0	0	1	1	1	1	1	1	0	0	0	0	0	0	0	6	6	0	-	0.0001998001998002
40942_hypothetical_protein	0	0	0	1	1	1	1	1	1	0	0	0	0	0	0	0	6	6	0	-	0.0001998001998002
40943_hypothetical_protein	0	0	0	1	1	1	1	1	1	0	0	0	0	0	0	0	6	6	0	S	0.0001998001998002
40944_hypothetical_protein	0	0	0	1	1	1	1	1	1	0	0	0	0	0	0	0	6	6	0	-	0.0001998001998002
40945_hypothetical_protein	0	0	0	1	1	1	1	1	1	0	0	0	0	0	0	0	6	6	0	S	0.0001998001998002
40946_hypothetical_protein	0	0	0	1	1	1	1	1	1	0	0	0	0	0	0	0	6	6	0	S	0.0001998001998002
40947_hypothetical_protein	0	0	0	1	1	1	1	1	1	0	0	0	0	0	0	0	6	6	0	-	0.0001998001998002
40948_hypothetical_protein	0	0	0	1	1	1	1	1	1	0	0	0	0	0	0	0	6	6	0	-	0.0001998001998002
40949_hypothetical_protein	0	0	0	1	1	1	1	1	1	0	0	0	0	0	0	0	6	6	0	-	0.0001998001998002
40950_hypothetical_protein	0	0	0	1	1	1	1	1	1	0	0	0	0	0	0	0	6	6	0	-	0.0001998001998002
40951_hypothetical_protein	0	0	0	1	1	1	1	1	1	0	0	0	0	0	0	0	6	6	0	L	0.0001998001998002
40952_hypothetical_protein	0	0	0	1	1	1	1	1	1	0	0	0	0	0	0	0	6	6	0	-	0.0001998001998002
40953_hypothetical_protein	0	0	0	1	1	1	1	1	1	0	0	0	0	0	0	0	6	6	0	K	0.0001998001998002
40954_dam_2	0	0	0	1	1	1	1	1	1	0	0	0	0	0	0	0	6	6	0	L	0.0001998001998002
40955_hypothetical_protein	0	0	0	1	1	1	1	1	1	0	0	0	0	0	0	0	6	6	0	-	0.0001998001998002
40956_hypothetical_protein	0	0	0	1	1	1	1	1	1	0	0	0	0	0	0	0	6	6	0	-	0.0001998001998002
40957_hypothetical_protein	0	0	0	1	1	1	1	1	1	0	0	0	0	0	0	0	6	6	0	-	0.0001998001998002
40958_hypothetical_protein	0	0	0	1	1	1	1	1	1	0	0	0	0	0	0	0	6	6	0	K	0.0001998001998002
40959_repB	0	0	0	1	1	1	1	1	1	0	0	0	0	0	0	0	6	6	0	S	0.0001998001998002
40960_hypothetical_protein	0	0	0	1	1	1	1	1	1	0	0	0	0	0	0	0	6	6	0	-	0.0001998001998002
40961_hypothetical_protein	0	0	0	1	1	1	1	1	1	0	0	0	0	0	0	0	6	6	0	-	0.0001998001998002
40962_hypothetical_protein	0	0	0	1	1	1	1	1	1	0	0	0	0	0	0	0	6	6	0	S	0.0001998001998002
40963_hypothetical_protein	0	0	0	1	1	1	1	1	1	0	0	0	0	0	0	0	6	6	0	S	0.0001998001998002
40964_hypothetical_protein	0	0	0	1	1	1	1	1	1	0	0	0	0	0	0	0	6	6	0	S	0.0001998001998002
40965_hypothetical_protein	0	0	0	1	1	1	1	1	1	0	0	0	0	0	0	0	6	6	0	-	0.0001998001998002
40966_hypothetical_protein	0	0	0	1	1	1	1	1	1	0	0	0	0	0	0	0	6	6	0	U	0.0001998001998002
40967_hypothetical_protein	0	0	0	1	1	1	1	1	1	0	0	0	0	0	0	0	6	6	0	-	0.0001998001998002
40968_hypothetical_protein	0	0	0	1	1	1	1	1	1	0	0	0	0	0	0	0	6	6	0	O	0.0001998001998002
40969_hypothetical_protein	0	0	0	1	1	1	1	1	1	0	0	0	0	0	0	0	6	6	0	S	0.0001998001998002
40970_traC	0	0	0	1	1	1	1	1	1	0	0	0	0	0	0	0	6	6	0	U	0.0001998001998002
40971_hypothetical_protein	0	0	0	1	1	1	1	1	1	0	0	0	0	0	0	0	6	6	0	D	0.0001998001998002
40972_virB	0	0	0	1	1	1	1	1	1	0	0	0	0	0	0	0	6	6	0	K	0.0001998001998002
40973_hypothetical_protein	0	0	0	1	1	1	1	1	1	0	0	0	0	0	0	0	6	6	0	-	0.0001998001998002
40974_hypothetical_protein	0	0	0	1	1	1	1	1	1	0	0	0	0	0	0	0	6	6	0	-	0.0001998001998002
40975_hypothetical_protein	0	0	0	1	1	1	1	1	1	0	0	0	0	0	0	0	6	6	0	-	0.0001998001998002
40976_hypothetical_protein	0	0	0	1	1	1	1	1	1	0	0	0	0	0	0	0	6	6	0	-	0.0001998001998002
40977_parM	0	0	0	1	1	1	1	1	1	0	0	0	0	0	0	0	6	6	0	O	0.0001998001998002
40978_hypothetical_protein	0	0	0	1	1	1	1	1	1	0	0	0	0	0	0	0	6	6	0	-	0.0001998001998002
45427_hypothetical_protein	0	0	0	1	1	1	1	0	1	0	0	0	0	0	0	0	5	5	0	-	0.0019980019980019984

Figure S20 - MccE492 Pan genome GWAS data for *S. enterica* strains. All the genes significantly associated with MccE492 resistance phenotypes in the pan or accessory genomes of the tested *S. enterica* strains. Represented are the SNPs, their loci and gene product as predicted in the *S. enterica* LT2 reference genome for each tested strain. The "SUM", "SUM_R", "SUM_S" are the total number of strains, the number of resistant strains, the number of susceptible strains, respectively, that have the corresponding SNP.

Bibliography

Delgado MA, Rintoul MR, Farias RN, Salomon RA. 2001. *Escherichia coli* RNA polymerase is the target of the cyclopeptide antibiotic microcin J25. *Journal of bacteriology* **183**: 4543-4550.

Guijarro JI, González-Pastor JE, Baleux F, Millán JLS, Castilla MA, Rico M, Moreno F, Delepierre M. 1995. Chemical structure and translation inhibition studies of the antibiotic microcin C7 (∗). *J Biol Chem* **270**: 23520-23532.

Huang K, Zeng J, Liu X, Jiang T, Wang J. 2021. Structure of the mannose phosphotransferase system (man-PTS) complexed with microcin E492, a pore-forming bacteriocin. *Cell Discov* **7**: 20.

Metlitskaya A, Kazakov T, Kommer A, Pavlova O, Praetorius-Ibba M, Ibba M, Krasheninnikov I, Kolb V, Khmel I, Severinov K. 2006. Aspartyl-tRNA synthetase is the target of peptide nucleotide antibiotic Microcin C. *J Biol Chem* **281**: 18033-18042.

Parks WM, Bottrill AR, Pierrat OA, Durrant MC, Maxwell A. 2007. The action of the bacterial toxin, microcin B17, on DNA gyrase. *Biochimie* **89**: 500-507.
